# A single-molecule spin–photon interface

**DOI:** 10.1038/s41586-026-11147-6

**Published:** 2026-10-28

**Authors:** Simon Roggors, Thomas Unden, Anna Aubele, Paul Mentzel, Gregor Bayer, Alon Salhov, Jochen Scharpf, Martin B. Plenio, Alex Retzker, Fedor Jelezko, Tim R. Eichhorn, Tobias A. Schaub, Matthias Pfender, Philipp Neumann, Ilai Schwartz

**Affiliations:** 1 NVision Quantum Technologies, Ulm, Germany; 2 Institute for Quantum Optics (IQO), Ulm University, Ulm, Germany; 3 Racah Institute of Physics, The Hebrew University of Jerusalem, Jerusalem, Israel; 4 Institute of Theoretical Physics, Ulm University, Ulm, Germany; 5 Center for Integrated Quantum Science and Technology (IQST), Ulm University, Ulm, Germany

**Keywords:** Qubits, Electronic structure of atoms and molecules, Magnetic materials, Optical materials

## Abstract

Optical interfaces that connect long-lived spin qubits to photons are a central requirement for quantum networking and distributed quantum information processing. At present, solid-state atomic defects are leading candidates because of their inherent spin and optical coherence^1,2^. Building on these advancements, synthetically tailored molecular systems represent a fundamental change in the field, using precise atomic control and consistent bottom-up assembly^3,4^. However, a molecular platform that concurrently satisfies the demands of a robust spin–photon interface—namely, the combination of bright fluorescence, high spectral stability and long ground-state spin lifetimes—has yet to be realized at the single-molecule level. Here we show that a triplet ground-state carbene molecule, embedded within a structurally matched host crystal, functions as a robust spin–photon interface with single-molecule addressability. The system exhibits narrow ZPLs, spectral stability over more than an hour, spin-selective optical transitions and single-molecule optically detected magnetic resonance. Coherent control yields millisecond-scale dynamical-decoupling coherence and tens-of-milliseconds spin relaxation at a temperature of 4.5 K. These results establish molecular qubits as a viable platform for single-emitter quantum optics^5,6^ while preserving the advantages of bottom-up chemical design^7^ and processable materials^8–10^.

## Abstract (web summary)

A triplet carbene molecule in a tailored crystal provides a stable single-molecule spin–photon interface with millisecond coherence, advancing chemically engineered molecular qubits for quantum networking.

## Main

At present, colour centres in wide-bandgap semiconductors, spearheaded by the nitrogen-vacancy centre in diamond, have driven landmark achievements from loophole-free Bell inequality tests^11^ and multi-node quantum networks^12^ to fault-tolerant quantum error correction^13^. For practical applications at room temperature, the nitrogen-vacancy centre remains an exceptional benchmark for solid-state spin lifetimes. However, semiconductor-based solid-state systems fundamentally suffer from extraneous electron defects and charge traps inherent to the host crystal, such as diamond^14,15^ or silicon carbide^16,17^. These fluctuating charge environments cause substantial optical spectral diffusion, degrading the spectral stability of the emitter. Although inversion-symmetric defects, such as the silicon-vacancy centre in diamond, offer better optical stability by mitigating these charge noise effects^18,19^, they instead face phonon-limited spin coherence times. By contrast, molecular crystals are assembled by weak intermolecular interactions rather than continuous covalent networks^4^ and are known to lack the extraneous electron defects and charge traps of semiconductor materials^20^ (for a quantitative comparison of different spin–photon interfaces; see Supplementary Information section VIII). Leveraging this pristine environment, alongside precise atomic control and consistent bottom-up assembly^3^, optical spin readout has been successfully demonstrated across a variety of molecular platforms. Originally, the first demonstration of optically detected magnetic resonance (ODMR) in single molecules was performed on pentacene, which possesses an excited-state triplet electron spin^21^. Progressing towards ground-state electron spin systems, in more recent works, organometallic chromium(IV) complexes^22^ have proven to be a versatile platform, with the ability to tune spin properties by chemically changing the qubit or the matrix^7^. Moreover, molecular rare-earth complexes leverage the inherently narrow optical linewidths of rare-earth ions within molecular crystal environments^23^, facilitating the optical readout of both electron^24^ and nuclear spin states^25^. Meanwhile, purely organic qubits such as diradicals^26–28^ and carbenes^29^ offer a distinct advantage; the absence of heavy atoms minimizes spin–orbit coupling, leading to significantly extended spin lifetimes and cyclical, coherent optical transitions. To date, however, experimental progress on molecular qubits with optical readout has been confined to bulk ensembles. To transcend the constraints of ensemble-averaged platforms and enable isolated single-molecule addressability, we used a robust molecular qubit, namely, di([1,1′-biphenyl]-4-yl)carbene, hosted within an isosteric, crystalline di([1,1′-biphenyl]-4-yl)methanone matrix, providing high fluorescence emission, spectral stability and favourable inter-system crossing (ISC) dynamics.

### Qubit design

**Fig. 1:**
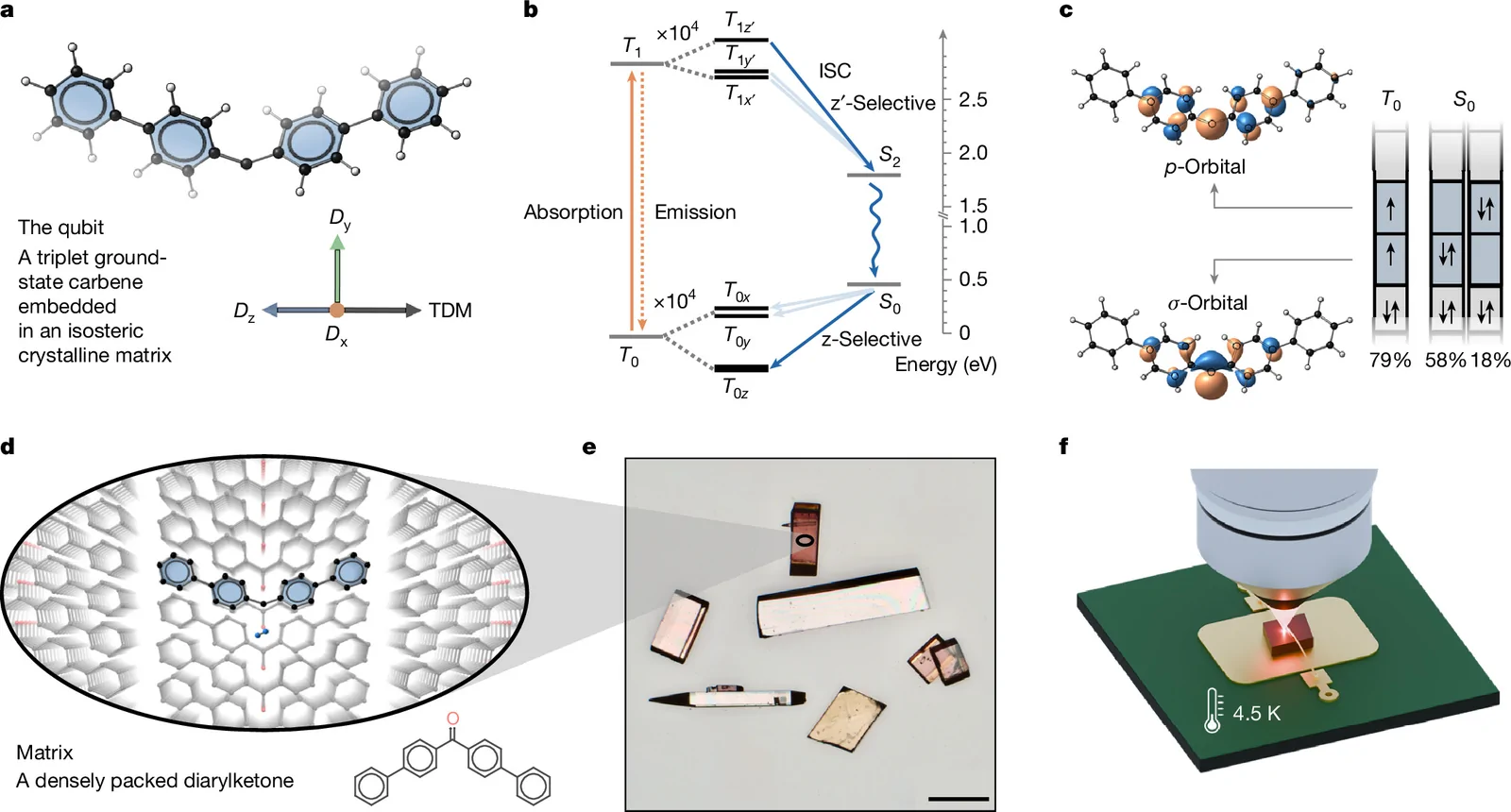
Molecular colour centre with a triplet ground state doped into a crystalline matrix. **a**, Molecular structure of the qubit molecule and nearby nitrogen molecule together with the ZFS tensor and the transition dipole moment (TDM) from SA8-CASSCF(10,10)/QD-SC-NEVPT2/def2-TZVPP calculations (Methods). **b**, Scaled energy levels (ZFS enlarged for clarity) showing the ZFS change between ground and photoexcited triplet states. Note the sign change of the ZFS parameter in *T*_0_ and *T*_1_ indicated by reordered *x*,* y*, *z* substates. The blue arrows illustrate optical spin pumping by ISC through the singlet manifold to the *T*_0*z*_ ground state (see Supplementary Information sections IV.F.8., VII.H. and VII. L.). **c**, Spin-bearing *σ*- and *p-*orbitals (isosurface = 0.04) and major spin configurations of the *T*_0_ and *S*_0_ states. **d**, The carbene and the nitrogen molecule embedded in the crystalline lattice of the ketone matrix. **e**, Representative crystals with a uniform 2.5 mol% doping rate shown in multiple orientations. Different colours stem from variations of the effective optical density along the viewing axis, scaling with crystal thickness and orientation. **f**, Schematic of the low-temperature confocal microscopy system. An MW wire is shown positioned over the doped crystal sample for local spin manipulation. Scale bar, 500 μm (**e**).

Our qubit molecule is a bent, pseudo-*C*_2_-symmetric diarylcarbene (carbene angle approximately 142°) with two *para*-connected biphenyls and derives its unique characteristics from the ferromagnetic exchange of two strongly correlated, unpaired electrons, which are co-localized on the divalent carbon centre and the proximal aryl units (Fig. 1a). To gain a deeper understanding of the compound in situ, we first relax its geometry while embedded in the molecular matrix, and subsequently compute the electronic structure of the isolated compound. Quantum chemical calculations predict that the *T*_0_ → *T*_1_ electronic transition possesses a locally excited state character, which is a key requirement for strong emission into the zero-phonon line (ZPL; see Supplementary Information section IV.E). During this transition, spin density formally redistributes from the central carbene *p*-orbital to an adjacent phenyl ring (Supplementary Information section IV.F.6 and Supplementary Fig. 27). Consequently, this spatial shift induces a substantial change in the magnitude and sign of the zero-field splitting (ZFS) parameters between the ground and excited triplet states (Methods). The resulting large frequency separation of spin-conserving transitions facilitates the optical addressability of individual spin states. The predictions also indicate that the lowest-lying singlet state *S*_0_ is significantly destabilized relative to the triplet ground state by Δ*E*_ST_ = 0.47 eV, ensuring a robust triplet configuration. In line with previous theoretical studies^30,31^ on the spin-optical interfaces of bent carbenes, the electronic structure renders ISC between the singlet and the triplet ground state allowed in ref. ^32^, promoting a highly spin-selective population of the *T*_0*z*_ state due to symmetry (for a discussion on the excited-state *T*_1*z*′_ selectivity, see Supplementary Information section IV.F.8).

Motivated by early reports on paradigmatic diphenylcarbene in diphenylketone^33,34^ and similar studies on carbene molecules^35–38^, we mitigate the inherent reactivity of carbenes^39,40^ using a precursor strategy in which a photoactivatable diaryldiazomethane precursor is embedded in an isosteric, rigid, crystalline matrix, namely, di([1,1′-biphenyl]-4-yl)methanone (Fig. 1d; see the Supplementary Information). Two key advantages emerge from this specific crystalline matrix that merit emphasis: (1) it imposes a rigid constraint that planarizes the biphenyl units (Ph–Ph dihedral angle about 6° compared with 36° in the gas phase). This minimizes structural changes when the molecule is optically excited to the *T*_1_ state, effectively funnelling more emission directly into the ZPL (Supplementary Information section IV.D); and (2) the specific perpendicular (edge-to-face) arrangement electronically separates the qubit and the surrounding matrix molecules by minimizing the overlap of their electron clouds^41^.

Homogeneously doped crystals with different morphologies and doping concentrations were prepared using standard solution-phase methods. For instance, vapour diffusion crystallization within a few days yielded tabular or prismatic millimetre-sized bulk crystals (Fig. 1e), whereas drop-casting produced parallelogramic sub-μm thin plates, all of which exhibited high optical quality (Methods and Supplementary Information). Beyond its aforementioned primary function, the photoactivation method offers a powerful feature in that it enables the activation of carbenes with sub-μm precision (Supplementary Information and Supplementary Fig. 36). This level of control, achieved through confocal microscopy, allows the qubit molecule density to be regulated independently of the overall doping level.

### Optical and electron spin spectroscopy

The precursor-doped crystal (25 ppm doping ratio) was oriented within a cryo-confocal microscope (Fig. 1f and Methods) to maximize coupling efficiency for both incident laser light and emitted photons. After cool-down to 4.5 K, the diffraction-limited laser focus was used to photoeliminate nitrogen, effectively converting the precursor molecules into dense ensembles of qubit molecules^40,42^ (for more information, see Supplementary Information section VII.B). Subsequent thermal annealing relieves local matrix strain and stabilizes the crystalline environment, as further corroborated by electron paramagnetic resonance (EPR) measurements (see Methods and Supplementary Information section VI.C). Although the photoeliminated nitrogen molecule remains trapped in close vicinity to the carbene, our data yield no indication of detrimental electrostatic interactions or correlated lattice strain at the used sparse doping concentrations (Supplementary Information section IV.F.9).

**Fig. 2:**
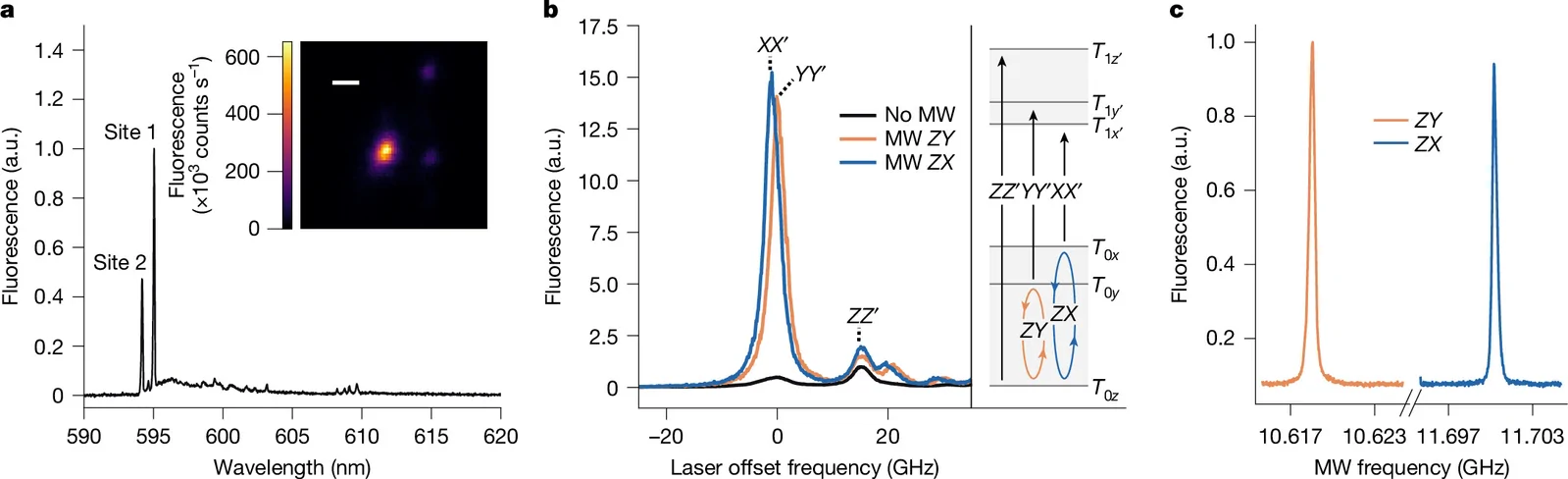
Ensemble optical spectroscopy and ODMR. **a**, The fluorescence spectrum of a carbene ensemble under 580 nm excitation exhibits two dominant ZPLs. The confocal microscopy scan in the inset shows several locally created ensembles. **b**, The excitation spectra show accumulated fluorescence photons (detection wavelength >605 nm) when scanning the laser frequency across the longer-wavelength ZPL (black). Additional microwave (MW) radiation resonant with spin transitions *T*_0*z*_ ↔ *T*_0*y*_ (orange) or *T*_0*z*_ ↔ *T*_0*x*_ (blue) significantly alters the fluorescence response. The inset shows a simplified triplet level scheme highlighting the actively manipulated transitions. **c**, The ODMR spectrum revealing the spin resonances exploited in **b** with the same colour coding. Scale bar, 2 μm (**a**). Source data

On continued laser excitation, the qubit molecule fluoresces and is thus observable in a confocal microscopy image (Fig. 2a, inset). The ensemble fluorescence spectrum exhibits two sharp ZPLs (at 594.18 nm and 595.04 nm in Fig. 2a, limited by the resolution of the spectrometer to about 0.1 nm full-width at half-maximum (FWHM), amounting to about 19% of total fluorescence), accompanied by a lower-energy vibronic and phononic sideband. We attribute the two ZPLs to the presence of two nearly identical, yet structurally distinguishable sites for qubit molecules within the host crystal (comparable, for example, to terrylene molecules in *p*-terphenyl crystals^43^). This assignment is further corroborated by detailed ODMR and EPR measurements (Supplementary Information sections VI.A and VII.D). Subsequent resonant optical excitation experiments focused on the higher-wavelength molecular site (595.04 nm) to prevent unwanted off-resonant excitation of the lower-wavelength species. Scanning the laser frequency across the ZPL and detecting the phonon sideband emission (>605 nm) reveals two inhomogeneously broadened excitation lines with a narrow FWHM of about 3 GHz (Fig. 2b, black curve). Comparably narrow lines are known, for example, for pentacene or terrylene in *p*-terphenyl^43,44^. The spectral splitting between the lines in Fig. 2b matches the expected energy difference for spin-conserving optical transitions in related carbene molecules^29^. To unambiguously assign these optical transitions to specific spin states, apart from laser excitation, we applied resonant microwave fields corresponding to either the *ZY *or *ZX* transition (see Fig. 2b, inset, for the nomenclature of the transitions). As shown in Fig. 2b, these microwave fields greatly increase the intensity of the lower-frequency excitation line and, moreover, reveal a sub-structure. The lower-energy peak is a composite of two overlapping optical transitions, *XX*′ and *YY*′. Optical excitation of the latter transitions induces parasitic ISC to the closest singlet state (*T*_1_ → *S*_2_), followed by an ISC decay mainly into the triplet ground state *T*_0*z*_, eventually removing the molecules from the optical excitation loop (compare also the calculated ISC rates in Fig. 1b). The application of resonant microwaves replenishes *T*_0*x*_ or *T*_0*y*_ populations from the *T*_0*z*_ state. This selectively enhances the fluorescence intensity of the corresponding optical excitation line, allowing us to directly map specific optical transitions to their distinct triplet spin states. The excitation line corresponding to the *ZZ*′ transition is low in amplitude because of a higher ISC rate for ${T}_{1{z}^{{\prime} }}\to {S}_{2}$ compared to ${T}_{1{x}^{{\prime} }}\to {S}_{2}$ and ${T}_{1{y}^{{\prime} }}\to {S}_{2}$, in accordance with the calculated values and excited state lifetime measurements (Fig. 1b and Supplementary Information section III.C). For the sake of consistency, all laser excitation spectra are plotted against the laser frequency offset relative to the centre of the lower frequency optical transition, which includes both the *XX*′ and *YY*′ transitions. Note that the excitation spectrum in Fig. 2b contains additional features beyond about 20 GHz that are also modulated when applying microwaves. These spectral features are consistent with isotope effects because of natural abundance ^13^C shifting the ZPL of the qubit molecule^45^ (Supplementary Information section VII.H). The ODMR spectra on ensembles of qubit molecules reveals two sharp and strong fluorescence maxima when the swept microwave frequency corresponds to transition *ZY* at 10.618 GHz or *ZX *at 11.700 GHz, while the laser is kept at laser offset frequency around 0 GHz (see Fig. 2c). From these values, the ground-state ZFS parameters are deduced to be $|{D}_{{T}_{0}}|=11.1597(2)\,\mathrm{GHz}$ and $|{E}_{{T}_{0}}|=540.9(2)\,\mathrm{MHz}$ (the sign of the ZFS parameters is obtained by quantum chemical simulations, as well as by EPR measurements in Supplementary Information section VI.A). Notably, the ensemble ODMR resonances show a FWHM of about 400 kHz, providing an upper bound on the distribution of ZFS parameters ${D}_{{T}_{0}}$ and ${E}_{{T}_{0}}$ in the probed ensemble.

### Single-molecule spectroscopy

**Fig. 3:**
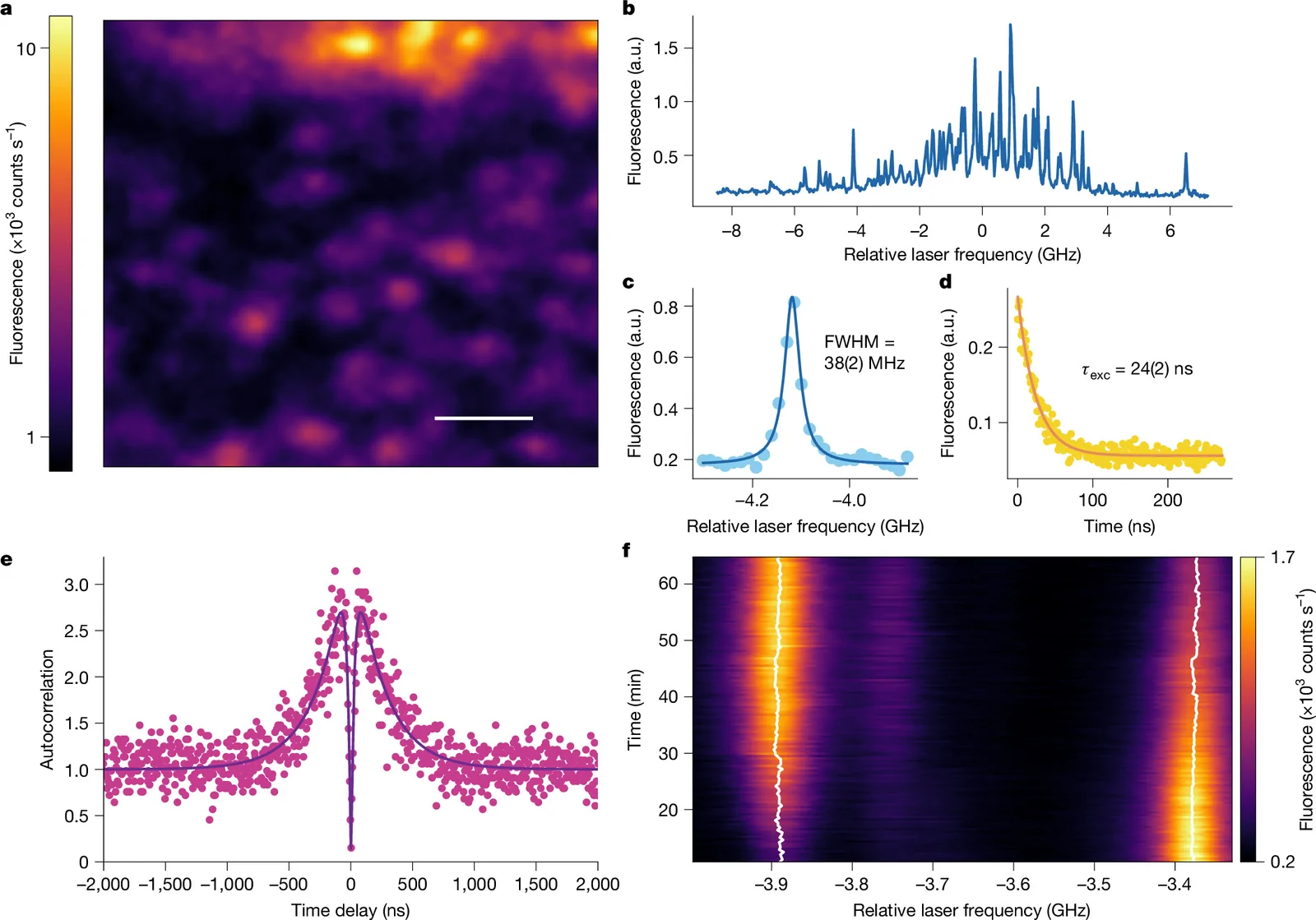
Single-molecule microscopy, spectroscopy and spectral stability. **a**, Confocal fluorescence microscopy showing a multitude of diffraction-limited bright spots in a sparsely activated region with 3 nW of resonant laser excitation. Fluorescence was enhanced by simultaneous microwave excitation (Methods). A Gaussian filter with *σ* ≈ 100 nm was applied to smooth the image. **b**, The laser excitation spectrum (with microwave as in **a**) reveals multiple sharp resonances (compare the previous inhomogeneously broadened spectrum of Fig. 2b). **c**, Excerpt from a spectral region of **b** showing an isolated resonance with a linewidth of 38(2) MHz. **d**, Pulsed laser excitation on this resonance revealing a fluorescence lifetime of 24(2) ns. **e**, Fluorescence autocorrelation measurement confirms the detection of a single emitter (${g}^{(2)}(0)=0.14{2}_{-0.023}^{+0.169}$). **f**, Long-term, repeated acquisition of fluorescence excitation spectra demonstrates spectral diffusion of the individual emitters with a standard deviation of about 2.6 MHz during 1 h. Scale bar, 2 μm (**a**). Source data

**Fig. 4:**
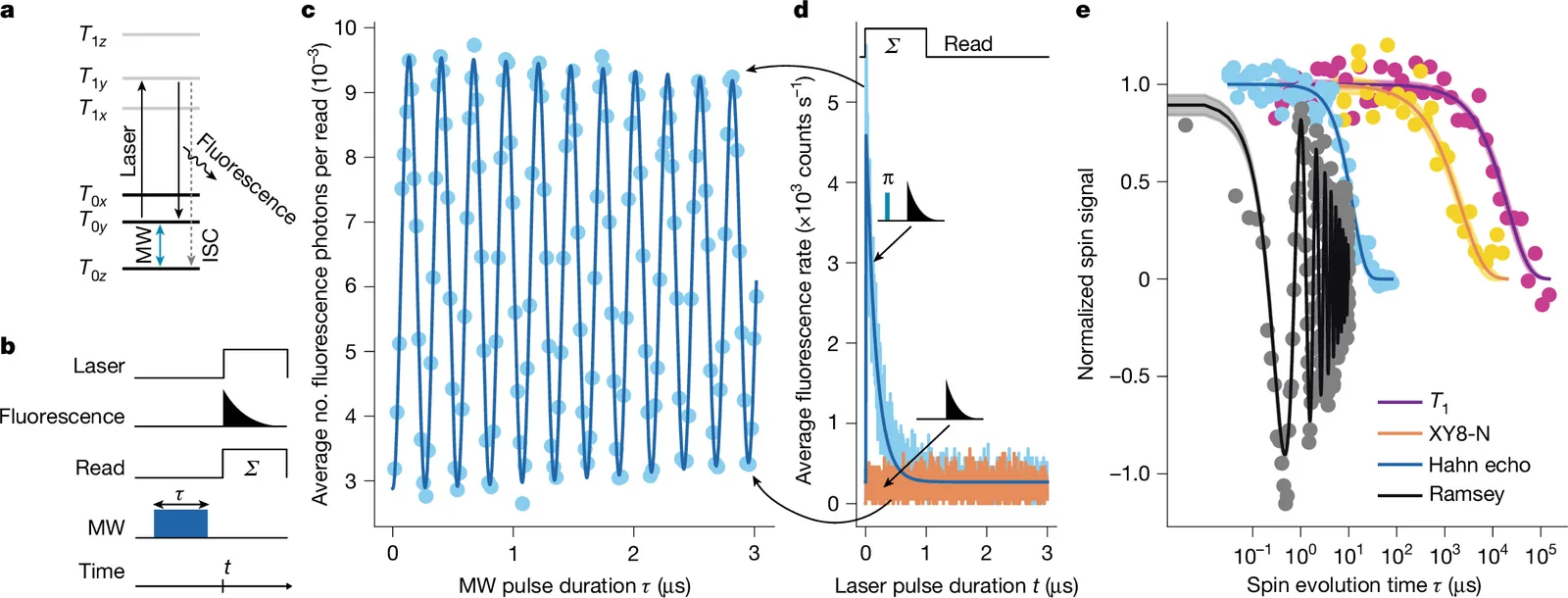
Single-spin initialization, manipulation and readout. **a**, Energy-level diagram with the most important transitions: resonant laser excitation *YY*′, excited-state decay through fluorescence to *T*_0*y*_ or through ISC to *T*_0*z*_ and microwave (MW) field resonant to transition *ZY*. **b**, Pulse sequence for spin Rabi oscillations: A MW pulse of duration *τ* coherently drives the initialized spin from *T*_0*z*_ to *T*_0*y*_ and back. The laser pulse invokes fluorescence only if the spin is in state *T*_0*y*_, and after prolonged exposure of a few μs initializes the system in *T*_0*z*_ through ISC. The amount of fluorescence photons constitutes the readout signal. The sequence is repeated for different *τ*. **c**, Resulting fluorescence signal showing Rabi oscillations of a single carbene spin with a frequency of 3.7 MHz. **d**, Exemplary fluorescence responses on laser excitation for the electron spin in state *T*_0*z*_ (orange, no MW pulse after initialization) or *T*_0*y*_ (blue, MW π-pulse after initialization). Prolonged laser excitation leads to spin initialization to *T*_0*z*_. **e**, Lifetimes of the single spin for various MW pulses sequences. During a Ramsey sequence (π/2 − *τ* − π/2) the spin signal decays after 5.3(4) μs to 1/*e*, during a Hahn-echo sequence (π/2 − *τ*/2 − π − *τ*/2 − π/2) after 12.2(6) μs, during an XY8-*N* sequence (${\rm{\pi }}/2-{[\mathrm{XY8}]}^{N}-{\rm{\pi }}/2$, with 1 μs π-pulse separation and *τ* = *N* × 8 × 1 μs for *N* = 1, …, 2,000) after 2.2(3) ms and during a *T*_1_-sequence (π − *τ* or *τ*) after 21(2) ms. Source data

Using a gradual photoactivation procedure, we obtain in situ spatial control over the qubit molecule density (Methods). Within a weakly activated region, the inhomogeneously broadened ensemble ZPL resolves into distinct, potentially single-molecule excitation lines (see Fig. 3b; microwave radiation targeting the *ZX* and *ZY* spin transitions was applied to enhance the fluorescence). Resonant optical excitation further isolates a subset of these activated molecules, yielding a sparse spatial distribution characteristic of individual emitters^44,46,47^ (Fig. 3a). To confirm the observation of a single molecule, we focused the laser onto an isolated spot and directed the fluorescence into a Hanbury Brown and Twiss setup. The resulting photon autocorrelation function exhibits a zero-delay coincidence dip to ${g}^{(2)}(0)=0.14{2}_{-0.023}^{+0.169}$, well below the threshold of 0.5, indicative of a single emitter^48^ (Fig. 3e and Methods). Scanning the excitation laser across a single-molecule resonance revealed a Lorentzian lineshape with a FWHM of 38(2) MHz (Fig. 3c). We determined the corresponding excited-state lifetime via rapid modulation of the excitation light. Given the measured decay time of 24(2) ns (Fig. 3d), the lifetime-limited linewidth of the optical transition is calculated to be 6.6(5) MHz. The measured linewidth of 38(2) MHz therefore indicates an additional pure-dephasing contribution. Temperature-dependent spectroscopy (Supplementary Information section VII.J) reveals that this broadening is largely driven by thermally activated coupling to low-energy lattice or intramolecular vibrational modes. Despite this phonon-assisted dephasing, repeated acquisition of excitation spectra over more than an hour (Fig. 3f) demonstrates remarkable spectral stability, with the fitted centre frequencies of two distinct molecular lines exhibiting standard deviations of just 2.4 MHz and 2.6 MHz. The slow changes in intensity of both lines are due to a relative drift between the focus of the microscope and the molecules. Representative photon count rates are on the order of 2 × 10^3^ counts s^−1^, limited mostly by a photoluminescence quantum yield (PLQY) of 10.8%, as well as a strong dependence of the count rate on the strength of simultaneously applied microwave driving (Supplementary Information sections III.B and VII.F). By removing one of the two applied microwave signals, and thus modulating the excitation spectrum as in Fig. 2b, the spectral separation of the *XX*′ and *YY*′ transitions was determined to be 1.555(1) GHz (Supplementary Information section VII.I). The latter frequency separation is the smallest of the spin-selective optical transitions, while still allowing for highly spin-selective optical excitation due to the narrow homogeneous linewidth of 38 MHz. Although the photoactivated di([1,1′-biphenyl]-4-yl)carbene molecules are chemically stable at cryogenic temperatures (Supplementary Information section VI.C), they exhibit photobleaching through a two photon-process that limits lifetimes under continuous laser and microwave radiation (similar to Figs. 3 and 4) of about 119 h (see Supplementary Information section VII.G).

### Single-spin readout

Optically detected single-spin manipulations were performed in a similar fashion as the ensemble measurements shown in Fig. 2c. A laser pulse resonant to the optical single-molecule transition *XX*′ or *YY*′ was used to measure the spin state, yielding a high fluorescence count rate when the spin of that molecule is in state *T*_0*x*_ or *T*_0*y*_, respectively, and only background photons otherwise (Fig. 4a,d). The relative fluorescence contrast of the readout is ≲95%, limited mostly by background fluorescence (Supplementary Information section VII.M). Furthermore, during laser illumination, the ISC eventually leads to the initialization of the electron spin in *T*_0*z*_ (Fig. 4d). In between laser pulses, a microwave pulse sequence coherently manipulates the electron spin in the triplet ground state and thus affects the fluorescence response to the subsequent laser pulse (Fig. 4b). As a prime example, Fig. 4c presents the Rabi oscillation of a single-qubit molecule due to a microwave pulse resonant to spin transition *ZY*. More complex microwave sequences were applied to measure spin properties such as the Hahn-echo spin coherence time *T*_2_ = 12.2(6) μs, the spin coherence time ${T}_{2}^{\mathrm{(XY8)}}=2.2(3)\,\mathrm{ms}$ during an XY8 dynamical decoupling sequence and the spin lattice relaxation time *T*_1_ = 21(2) ms (Fig. 4e and Methods). Given the abundance of proton nuclear spins in the environment, these long spin lifetimes are maintained because of the suppressive effect of the large ZFS in *T*_0_ compared with common hyperfine couplings^7,29^. Although Fig. 4d demonstrates rapid spin depolarization due to optical excitation, the high selectivity of the ISC can be leveraged for repetitive readout of a subspace of the triplet spin (Supplementary Information section VII.M).

### Conclusion and outlook

Although bulk ensembles of molecular defects remain highly valuable for applications such as absorption-based quantum memories^49^, the ability to isolate and address a single molecule unlocks a distinct class of quantum protocols by establishing a direct, one-to-one mapping between the optical transition and the individually controlled ground-state electron spin. By substitutionally embedding photoactivated triplet ground-state carbenes within an isosteric, structurally matched crystalline matrix, we achieved single-emitter addressability, characterized by high fluorescence, narrow optical linewidths and exceptional long-term spectral stability. This pristine interface not only facilitated highly efficient spin-selective excitation but also enabled the coherent control and measurement of the magnetic sub-structure of the molecule, yielding a ${T}_{2}^{{\rm{(XY8)}}}$ time of 2.2(3) ms and *T*_1_ time of 21(2) ms at 4.5 K. Considering an exponential decay of the coherence, the storage fidelity can be approximated as $F\simeq \frac{1+\exp [-t/{T}_{2}]}{2}$. Thus, given teabove values for ${T}_{2}^{{\rm{(XY8)}}}$, a quantum state can be stored for 40 μs with a fidelity of 99%. These single-molecule electron spin values represent a substantial leap for molecular qubits, extending previously reported values on ensembles by more than one order of magnitude^7,29^.

Although the focus of this work was the demonstration of an individually addressable molecular qubit, we believe it is worth placing its properties in the broader context of the requirements for a quantum-network node. A widely used set of benchmarks for such a spin–photon interface includes a spin coherence time sufficient to store quantum information between entanglement attempts, a high rate of useful photons, combining a large Debye–Waller factor, high PLQY and sufficient photon emission rate, near-lifetime-limited and spectrally stable optical transitions to enable photon indistinguishability between remote nodes, and spin-selective optical transitions for high-fidelity spin–photon entanglement^1^. Measured against these criteria, the qubit molecule reported here already combines many of the required properties in a single system: hour-scale optical spectral stability of 2.4–2.6 MHz, a narrow optical linewidth of 38 MHz, spin-selective optical transitions separated by more than 1.5 GHz, and millisecond-scale spin coherence under dynamical decoupling, all at a comparatively high operating temperature of 4.5 K. The net emission efficiency into the ZPL, combining a 10.8% PLQY and 19% DWF, is about 2.1%, already comparable to that of solid-state defects such as the nitrogen-vacancy centre (see Supplementary Information section VIII for a quantitative comparison to other colour centres). This combination of properties demonstrates the promise of molecular qubits as spin–photon interfaces, with clear, and in several cases already demonstrated, routes to further improvement.

Rational, bottom-up molecular design offers a concrete route to further optimize photophysical parameters such as PLQY, excited-state lifetime and ISC dynamics, as already demonstrated by targeted donor-strength tuning in related molecular radical qubits^50^ and by deuteration-induced quantum-yield enhancement^29,51^. This, as well as lowering the operating temperature to 2–4 K, has the potential to prolong *T*_1_ and *T*_2_ (ref. ^29^), and coherent memory times can be extended significantly further by using nuclear spins^25,52^. Furthermore, the bottom-up nature of chemical synthesis allows for the deterministic creation of localized nuclear spin registers through site-selective isotopic labelling, offering a degree of uniformity unattainable at present in solid-state systems.

Beyond fundamental research, this platform offers immediate pathways for advanced technological applications. The intrinsic processability of these molecular systems, including their application in ultrathin films, facilitates their integration with mature photonic integrated circuits based on materials such as silicon nitride or lithium niobate^8–10^.

This integration provides a viable route for the on-chip routing of photons entangled with single-qubit molecules, serving as a fundamental building block for quantum repeater nodes^53^ and distributed quantum computing^54^. This also highlights an important advantage over semiconductor solid-state defects: integrating semiconductor defects into photonic architectures typically requires complex top-down nanofabrication or etching of the host substrate, which is known to introduce local lattice strain and degrade the spectral stability of the emitter^14,15^. Molecular systems, however, can be processed into thin films and directly deposited onto pre-fabricated photonic circuits^8–10^, minimizing substrate-induced degradation.

This hybrid integration architecture is supported by the robust electronic structure of the carbenes: the substantial energy separation between spin-selective transitions of at least 1.555(1) GHz is more than two orders of magnitude greater than the lifetime-limited linewidth of 6.6 MHz. This ensures that high-fidelity spin addressability will persist even when excited-state lifetimes are deliberately shortened through coupling to high-finesse optical cavities, enabling an increased PLQY, Debye–Waller factor and photon collection efficiency.

Apart from the photonic manipulation unlocked by PIC integration, recent studies have shown that periodically poled lithium niobate can effectively transfer the photon quantum state from the visible band to the telecom band, unlocking accessibility to low-loss optical fibres and long-range quantum communication applications^55^.

Ultimately, this work introduces a structurally precise and chemically tunable interface that promises a scalable framework for the next generation of quantum technologies.

## Methods

### Computational methods

Multiscale ONIOM (QM/QM2) optimization^56,57^ as implemented in ORCA 6.1.0 (refs. ^58,59^) was used to simulate the carbene with a nearby nitrogen in situ, that is, specifically taking into account the matrix environment. ONIOM calculations were performed by dividing an 837-atom system derived from the experimental single-crystal structure of the matrix (CCDC 2542406) into a 309-atom high-level QM subsystem (carbene + N_2_ and first shell of six surrounding matrix molecules) and a fixed-coordinate, 528-atom, low-level QM2 subsystem (second shell of 12 surrounding matrix molecules), using a subtractive coupling scheme with electrostatic embedding.

The central, high-level QM model part was treated by unrestricted Kohn–Sham calculations with density functional theory (DFT) using the B97-3c composite approach^60^. This method natively incorporates the modified triple-zeta split-valence basis set of Ahlrichs (def2-mTZVP)^61^ alongside the atom-pairwise dispersion correction with the Becke–Johnson damping scheme (D3BJ)^62,63^. The Coulomb integrals were evaluated using the Split-RI-J approximation with the def2-mTZVP/J auxiliary basis set^64^. Matrix molecules in the QM2 region were computed using the semiempirical extended tight-binding method GFN2-xTB (XTB2)^65^. The relaxed cluster was used to extract the final coordinates of the carbene qubit in the triplet ground state (Fig. 1).

Time-dependent density functional theory (TD-DFT) calculations used the B3LYP functional^66–68^ in combination with the def2-TZVPP basis set^61^ and the D4 dispersion correction^69^. In this calculation, the Coulomb and exchange contributions were evaluated using the resolution-of-identity approximation^70^ in conjunction with the RIJCOSX scheme^71^.

For prediction of spin dynamics, we use the state-averaged complete active space self-consistent field (SA-CASSCF) method^72,73^ corrected by the van Vleck quasi-degenerate extension to the strongly contracted second-order *n*-electron valence state perturbation theory (SC-NEVPT2) to account for dynamic correlation (spin–orbit and spin–spin coupling)^74–76^. The active space included 10 electrons in 10 orbitals. The wavefunction is state-averaged over all states that are close in energy to the region of interest; in our case, the first three triplet and five singlet states (SA8). The def2-TZVPP basis set^61^ was used for all atoms, alongside the def2-TZVPP/C^77^ auxiliary basis set to accelerate the evaluation of Coulomb and exchange integrals through the RIJCOSX approximation^78^. These calculations predict an oblate ZFS tensor in the *T*_0_ ground state and a prolate tensor in the *T*_1_ excited state. The calculated spin-conserving transition frequencies and the vertical singlet–triplet splitting (Δ*E*_ST_) are summarized in Extended Data Table 1.

### Sample preparation

All crystals of the qubit molecule doped into the matrix were grown by solution-based crystallization methods and under exclusion of daylight, that is, under red light ≥600 nm to avoid slow decomposition of the diazomethane precursor. In a typical procedure, di([1,1′-biphenyl]-4-yl)methanone is dissolved in hot THF (stabilizer-free, HPLC grade, 0.014 M) and cooled to room temperature before the diazomethane precursor dopant is added as a solid and dissolved using an ultrasonication bath for a few seconds. The corresponding solution is filtered through a syringe filter (PTFE 0.2 μm) and added to vials (2–5 ml) standing in a screw-cap jar. The jar is filled with *n*-hexane (fill height about 2–3 cm) and sealed. After standing in the dark at room temperature for several days, doped crystals form at the bottom of the vials. The supernatant is removed using a Pasteur pipette to avoid mechanical stress on the crystals. The residue is then washed twice with small volumes of THF/*n*-hexane (1:2) without allowing the crystals to dry out. This approach preserves the surface quality and prevents secondary nucleation or island-like growth. The resulting, air-dried crystals remain stable in a freezer (*T* = −22 °C) for at least 6 months. A doped crystal of about 1 mm^3^ volume (25 ppm doping ratio) with an optically flawless appearance was selected and mounted onto a cryogenic cold finger using vacuum grease to ensure optimal thermal contact. The crystal was oriented such that its transition dipole moment vector of the *T*_0_ → *T*_1_ transition was within the plane of polarization of the laser beam. A 25 μm diameter copper wire was positioned across the sample to facilitate microwave delivery for spin manipulation.

### Cryogenic microscopy and spectroscopy experiment

Optical excitation and fluorescence experiments were performed using a custom-built cryogenic confocal microscope operating at a temperature of approximately 4.5 K at the sample mount (for a detailed sketch of the experiment, see Supplementary Information section VII.A). The molecular crystal sample was attached to a sample platform (4.5 K) of a closed-cycle optical cryostat. The microscope objective (NA = 0.9, WD = 1 mm) is within the vacuum shroud of the cryostat but at room temperature and separated from the cold sample by a thin metal plate thermalized at 50 K with a small aperture (about 4 mm). The sample platform can be coarsely moved with respect to the objective by slip-stick positioners, and a galvo-scanner outside the cryostat allows fine scanning of the laser beam across the sample through a telecentric lens system. Optical excitation is performed using a tunable (570–620 nm) dye laser using rhodamine-6G as the laser medium. Initial photoactivation and off-resonant optical excitation of the qubit molecule is performed at a wavelength of 580 nm. For full activation 5 μW of laser light was focused on the crystal surface for about 2 min; and for mild activation about 1 μW of focused laser light was scanned across the crystal surface. For annealing, the crystal with as-activated carbenes was heated up to 200 K for about 1 h. Fluorescence spectra were recorded under off-resonant laser illumination (580 nm, 5 μW, 545/70 nm band-pass filter for laser clean-up) using a 590-nm long-pass filter in front of the spectrometer. Resonant laser excitation for excitation spectroscopy, microscopy or spin-state initialization and readout typically requires powers of approximately 3 nW, a 600-nm short-pass filter for clean-up and a 605-nm long-pass for fluorescence detection. The angle of linear laser polarization was adjusted to maximize qubit molecule fluorescence. For some experiments, continuous-wave (CW) resonant optical excitation was accompanied by CW resonant spin excitation in the ground-state spin triplet to increase fluorescence intensity. In detail, when resonant optical excitation on the optical transitions *XX*′ or *YY*′ shelves the molecule into the dark state *T*_0*z*_, resonant microwave spin excitation on transitions *ZX* or *ZY*, respectively, retrieves this molecule from *T*_0*z*_ and thus enables continuous optical excitation. The shelving process is the effect causing the fluorescence decay shown in Fig. 4d. This technique was used for experiments with results shown in Figs. 2b,c and 3. Notably, this microwave recovery strongly influences the achievable saturation count rate, which can be seen in saturation measurements for different applied microwave Rabi frequencies in Supplementary Information section VII.F The laser intensity can be switched on and off within a few tens of nanoseconds using an acousto-optical modulator that yields an on–off ratio of the laser intensity of more than 10^4^. An electro-optical modulator with a bandwidth of 5 GHz is used to provide faster switching times required for excited-state lifetime measurements. The strength of the applied microwave field was varied between experiments. It was strongest for the Rabi measurement in Fig. 4c. For microwave-assisted fluorescence excitation spectra as shown in Fig. 2b, the microwave field was reduced by more than a factor of 10, corresponding to a Rabi frequency of about 250 kHz. For CW-ODMR measurements as shown in Fig. 2c, the microwave driving was reduced further to a Rabi frequency of about 40 kHz.

### Single-spin initialization, coherent control and readout

The procedure for conducting basic single-spin manipulation and readout measurements is described in the main text for the example of Rabi oscillations (Fig. 4b–d). In the latter case, the electron spin was manipulated during laser-off periods, and the readout signal comprised the sum of all fluorescence photons during laser-on periods (Fig. 4d) divided by the number of laser pulse repetitions (typically, about 2 × 10^6^ repetitions are performed). For the pulsed spin experiments depicted in Fig. 4e, namely, Hahn-echo, XY8-*N* and *T*_1_ measurements, the spin signal is derived slightly differently. In the latter cases, each data point is the difference of two complementary measurement results. For example, in the *T*_1_ measurement, two complementary measurements for a certain data point are (1) a bare waiting time *τ* before the next readout laser pulse and (2) a microwave π-pulse followed by the same waiting time before the next readout laser pulse. Here, (1) yields a low and (2) a high number of fluorescence photons. When the spin states of the complementary measurements have decayed to the equilibrium state, the number of fluorescence photons is supposed to be equal and thus the difference is zero. In the case of the Hahn-echo and XY8-*N* measurements, two complementary measurements differ only by the phase of the last π/2-pulse within the microwave sequence; it is either 0 or π. Finally, a stretched exponential decay, $$f(\tau )=A\times \exp \{-{(\tau /T)}^{\beta }\}\,+$$$${y}_{0}$$, was fitted to the data; for the *T*_1_ measurement $$\beta \,\mathop{=}\limits^{!}\,1$$. The displayed data are then normalized to the respective value of *A*. The stretch-factor *β* for Hahn-echo and XY8-*N* measurements are 1.7(2) and 0.9(2), respectively. Deviations from *β* = 1 for Hahn-echo and XY8-*N* measurements are known for decoherence of single spins and hint towards non-Markovian processes, as expected for proximal electron and nuclear spins such as protons in the present case^79–81^.

### Fit of the second-order photon autocorrelation

The measured second-order autocorrelation function *g*^(2)^(*τ*) is analysed using a three-level system model to capture both the single-photon anti-bunching, as well as the bunching dynamics of the singlet state. The ideal correlation function is parameterized as

$${g}_{\mathrm{ideal}}^{(2)}(\tau )=1-(1-{g}_{0}+A){{\rm{e}}}^{-| \tau -{\tau }_{0}| /{\tau }_{\mathrm{anti}}}+A\times {{\rm{e}}}^{-| \tau -{\tau }_{0}| /{\tau }_{\mathrm{bunch}}}$$

with the relative amplitude *A* of the bunching behaviour, the depth of the anti-bunching *g*_0_, the timescales of the anti-bunching and bunching *τ*_anti_ and *τ*_bunch_, respectively, and the electronic coincidence shift *τ*_0_. To account for finite timing resolution of the measurement, ${g}_{{\rm{ideal}}}^{2}(\tau )$ is convolved with a Gaussian instrument response function. The total standard deviation of the temporal jitter *σ*_total_ = 1.56 ns was calculated by taking the root mean square of two times the variance of the detector jitter (*σ*_SPAD_ = 0.425 ns) and the uniform discretization error introduced by the 5 ns temporal binning of the correlation data (${\sigma }_{\mathrm{bin}}=5/\sqrt{12}\,\mathrm{ns}$). Finally, the convolved model is fitted to the raw correlation data using a non-linear least-squares algorithm, incorporating a baseline scaling parameter to ensure the fitted *g*^(2)^(*τ*) correctly normalizes to 1 as *τ* → *∞*. The fitted values in Fig. 3e are *τ*_bunch_ = 245(9) ns, *τ*_anti_ = 28(2) ns and ${g}^{(2)}(0)=0.14{2}_{-0.023}^{+0.169}$, with the *g*^(2)^(0) value denoting the lowest point of the convoluted autocorrelation function, while the underlying value is lower. To estimate the uncertainty of the *g*^(2)^(0) value, a Monte Carlo simulation is performed by generating synthetic data sets from the covariance matrix of the fit. The fitted anti-bunching timescale of *τ*_anti_ = 28(2) ns corresponds well with the lifetime of the excited state from Fig. 3d, hinting towards optical excitation below saturation^82,83^. The strong bunching behaviour is caused by resonant excitation of the *YY*′ transition and the resulting spin polarization into the *T*_0*z*_ state. The decay rate of the bunching *τ*_bunch_ does allow insight into the dynamics of spin polarization, however, because of the complex landscape of transition rates and microwave drives, it does not give direct access to the ${T}_{1{y}^{{\prime} }}$ ISC rate.

## Online content

Any methods, additional references, Nature Portfolio reporting summaries, source data, extended data, supplementary information, acknowledgements, peer review information; details of author contributions and competing interests; and statements of data and code availability are available at https://doi.org/10.1038/s41586-026-11147-6.

## Supplementary information

Supplementary Information This file contains Supplementary Information sections 1–8, including Supplementary Tables 1–13 and Supplementary Figs. 1–55.

Peer Review File

## Source data

Source Data Figs. 2, 3 and 4

## Figures and Tables

**Extended Data Table 1:**
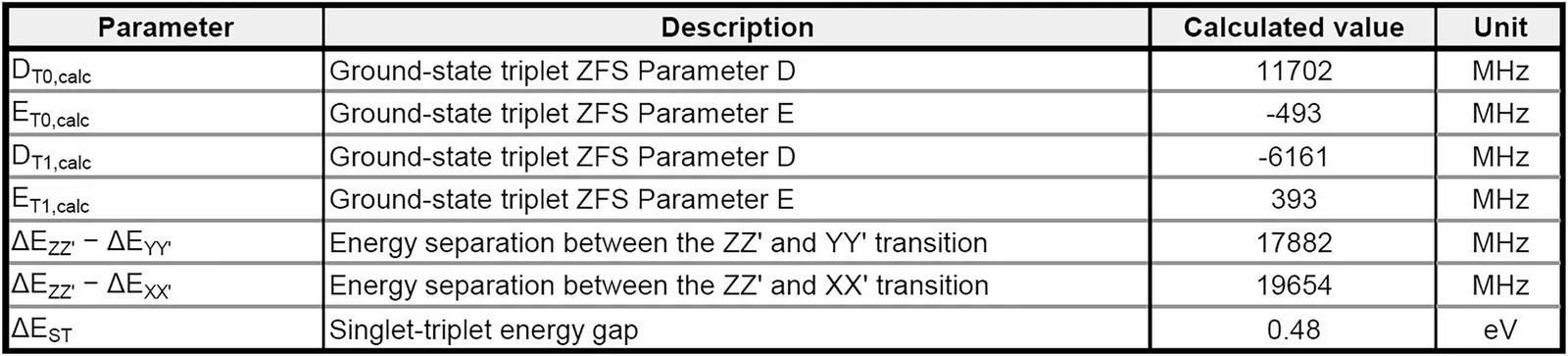
Properties of the carbene qubit predicted from multiconfigurational SA8-CASSCF(10,10)/QD-SC-NEVPT2/def2-TZVPP calculations

## Data Availability

Crystallographic X-ray data that support the findings of this study have been deposited in the Cambridge Structural Database with the CCDC accession number 2542406. ORCA output files for multiscale ONIOM geometry optimization as well as TDDFT and CASSCF/NEVPT2 single-point calculations of the final qubit can be found at https://doi.org/10.6084/m9.figshare.31963335. The data that support the findings of this study are available from the corresponding authors upon request. Source data are provided with this paper.

## Code availability

All analysis codes related to the current study are available from the corresponding authors upon request.
