# Supplementary material for: A single-molecule spin–photon interface
Source: Nature. 2026 Oct 28;658(8138):1153–9. doi: 10.1038/s41586-026-11147-6 (PMC13716814; doi:10.1038/s41586-026-11147-6)
Supplement: Supplementary file 1 [file 41586_2026_11147_MOESM1_ESM.pdf]

nature portfolio

https://doi.org/10.1038/s41586-026-11147-6

Supplementary information

A single-molecule spin–photon interface

In the format provided by the
authors and unedited

Supplementary Information: "A Single-Molecule Spin-Photon Interface"

CONTENTS

I. Chemistry and material preparation 2
A. Synthesis and characterization 2
1. Experimental methods 2
2. Synthesis of di([1,1'-biphenyl]-4-yl)diazomethane 2
B. NMR spectra 5

II. Material fabrication and sample preparation 11
A. Growth of doped molecular crystals 11
B. X-ray crystallographic analysis 11

III. Optical spectroscopy of bulk materials and frozen solutions 15
A. Steady-state spectroscopy 15
B. Photoluminescence quantum yields 16
C. Time-correlated single photon counting 16

IV. Computational chemistry 19
A. Computational methods 19
B. Precursor ground state geometry 20
C. Triplet ground state geometry (T_0) 21
D. Excited state geometry in vacuum (T_1) 22
E. Property prediction via TD-DFT 23
F. Property prediction via CASSCF/NEVPT2 23
1. Active space deduction 23
2. Complete active space 23
3. Singlet triplet gap 25
4. State structure 25
5. Energy level structure 26
6. Spin densities 27
7. Zero-field splitting: Tensor parameters and spin state overlap 29
8. ISC pathways due to SOC 29
9. The role of the photocleaved nitrogen 31

V. Microscopy and refractive index tensor of undoped crystals 32

VI. EPR Spectroscopy: Ensemble GS ZFS tensor 33
A. Rotation-dispersion spectra: ground state (GS) ZFS frame 33
B. Comparison: Crystal, ground state ZFS and refractive index frame 34
C. Thermal Stability 35

VII. Cryo-confocal measurements 35
A. Experimental setup and estimated collection efficiency 35
1. Excitation path 36
2. Sample Environment 36
3. Collection Path and Detection 37
4. Estimated Collection Efficiency 37
B. Activation mechanism and yield 38
C. Comparison of annealed and nascent carbenes 39
D. Off-resonant ODMR on both annealed molecular sites 41
E. Determination of the Debye-Waller factor 42

F. Fluorescence saturation measurements 43
G. Photo-bleaching in ensembles of di([1,1'-biphenyl]-4-yl)carbene 44
H. Ensemble excitation spectra and ^{13}C isotope effects 45
I. Single molecule spectra and spin state assignment 47
J. Temperature dependence of position and width of optical resonances 48
K. Optically detected magnetic resonance of the excited state 49
L. Fluorescence lifetime measurements 49
M. On the prospect of single-shot readout 51
1. Repetitive readout of a subspace of the triplet spin 52

VIII. Comparison of spin-photon interfaces 53

References 54

I. CHEMISTRY AND MATERIAL PREPARATION

A. Synthesis and characterization

1. Experimental methods

All commercially available chemicals were purchased from Sigma Aldrich, TCI Germany, VWR International, Fischer Scientific, Acros Organics, ABCR, ChemPur, Strem, BLDpharm, and Alfa Aesar. Unless otherwise noted, commercially available materials were used without purification. Moisture and oxygen sensitive reactions were carried out in flame-dried glassware and under an inert atmosphere of purified argon using syringe/septa technique. Commercially available anhydrous tetrahydrofuran (≥99.9%, THF) was stored over molecular sieve (3 Å) and used without purification.

Thin-layer chromatography (TLC) was performed on the aluminium plates coated with 0.20 mm thickness of Silica gel 60 F_{254} (Macherey-Nagel). Developing plates were visualized using UV light at wavelength of 254 and 365 nm. ^1H and ^{13}C NMR spectra were recorded on a Bruker Avance III HD 400 (^1H: 400.1 MHz, ^{13}C: 100.6 MHz) spectrometer at 25 °C. The chemical shifts (δ) are reported in parts per million (ppm) and are referenced to solvent signal: $\delta = 5.32$ ppm (^1H) and $\delta = 53.8$ ppm (^{13}C) for CD_2Cl_2 and $\delta = 3.58$ ppm (^1H) and $\delta = 67.2$ ppm (^{13}C) for THF-d_8. Coupling constants (J) are given in Hz and the apparent resonance multiplicity is reported as singlet (s), doublet (d), triplet (t), or multiplet (m). 2D Correlation NMR spectra were recorded for signal assignment of all target compounds.

FT-IR spectra were recorded on pure powders using a Spectrum Two FT-IR spectrometer (PerkinElmer, USA) equipped with a GladiATR™ ATR accessory (Pike Technologies, USA).

UV/Vis absorption spectra were recorded on a Thermo Scientific™ GENESYS™ 150 spectrophotometer. All measurements were performed in methylcyclohexane using quartz cuvettes with a path length of 1 cm at room temperature. The extinction coefficient ϵ was determined by measuring the absorption at different concentrations.

High resolution MALDI mass spectra were recorded on a Bruker SolariX using Dithranol as matrix. Chemical ionisation (CI) mass spectra were recorded on a Thermo Scientific ISQ LT single quadrupole mass spectrometer by direct vaporization of samples into the MS ionization chamber (DIP) using methane as reagent gas. For sample **S3** the molecular mass was not observed which we ascribe to the delicate combination of thermal, light, and acid sensitivity of the diaryldiazomethanes. Instead, the MALDI mass spectrum of **S3** shows fragment ions at m/z 319.14902 (100%) resulting from N_2-cleavage and protonation together with a larger cluster at 665.29577 (74%) $[2M–N_2+H]^+$.

Automated flash chromatography was performed on a Biotage® Selekt system using Biotage® Sfär silica gel cartridges (20 µm). Product purification was monitored by UV absorbance at 250 nm and 300 nm. Matrix compound **S1** was purified by vacuum sublimation in the last step to afford sufficiently pure material. Elemental analysis was carried out on a Elementar vario Micro cube at the Core Facility Elemental, Molecular and Materials Analysis (University Ulm). Melting points were determined with a Büchi M-565 using 2 °C/min heating rate.

2. Synthesis of di([1,1'-biphenyl]-4-yl)diazomethane

Di([1,1'-biphenyl]-4-yl)methanone (S1): The compound was prepared according to a modified literature procedure[1] (see Supplementary Figure 1). 4,4'-Dibromobenzophenone (5.00 g, 14.7 mmol) and phenylboronic acid (4.12 g, 33.8 mmol) were dissolved in a degassed mixture of toluene (123 mL, 0.12 M), ethanol (63 mL, 0.23 M) and water (63 mL, 0.23 M). K_2CO_3 (12.2 g, 88.2 mmol) and $Pd(PPh_3)_4$ (0.34 g, 0.29 mmol) were added, and the reaction

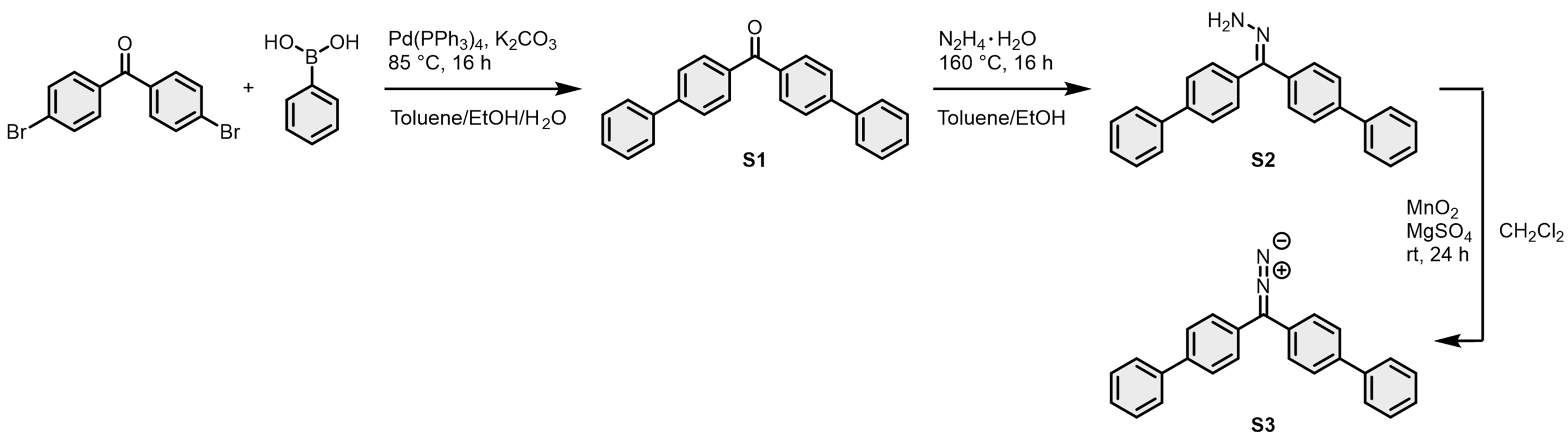

Supplementary Figure 1. Synthetic route to matrix S1 and dopant **S3**.

mixture was stirred for 16 h at 85 °C. After cooling to room temperature, water was added and the layers were separated. The aqueous phase was extracted with ethyl acetate (2 × 200 mL). The combined organic phases were washed with water (300 mL) and brine (200 mL), dried over anhydrous Na_2SO_4, filtered, and concentrated under reduced pressure. The crude product was purified by column chromatography (flash-silica gel; CH_2Cl_2/cyclohexane 1:1) to obtain di([1,1'-biphenyl]-4-yl)methanone (**S1**) as a colourless solid (4.5 g, 13.5 mmol, 92% yield). Analytically pure material was obtained by vacuum sublimation (3×10^{-2} mbar, 195 °C). R_f (SiO_2, CH_2Cl_2/n-hexane 3:2) = 0.33. Mp. 238 – 240 °C (lit.:[1] 238 – 240 °C); ^{1}H NMR (400 MHz, CD_2Cl_2) δ 7.92 (d, J = 8.7 Hz, 4H, H1/H3), 7.76 (d, J = 8.7 Hz, 4H, H4/H12), 7.70 (d, J = 8.2 Hz, 4H, H7/H11), 7.50 (m, 4H, H8/H10), 7.42 (t, J = 7.3 Hz, 2H, H9) ppm; ^{13}C NMR (101 MHz, CD_2Cl_2) δ 195.9 (C13), 145.4 (C5), 140.3 (C6), 136.9 (C2), 131.0 (C1/3), 129.4 (C8/10), 128.6 (C9), 127.7 (C7/11), 127.3 (C4/12) ppm; IR (ATR): $\tilde{\nu}$ = 1639 (C=O) cm^{-1}; UV/Vis (methylcyclohexane): λ_{max} = 295 nm (ϵ = 30 400 M^{-1} cm^{-1}); HRMS (APCI, positive mode) m/z calcd for $C_{25}H_{18}O$: 335.14304 [M+H]$^+$, found 335.14300; Elemental analysis (%calcd, %found for $C_{25}H_{18}O$): C (89.79, 90.12), H (5.43, 5.67). The analytical data is in accordance with literature data.[1]

(Di([1,1'-biphenyl]-4-yl)methylene)hydrazine (S2): The compound was prepared according to a modified literature procedure.[2] A mixture of di([1,1'-biphenyl]-4-yl)methanone (300 mg, 0.90 mmol), hydrazine monohydrate (N_2H_4 64-65% 0.14 mL, 2.70 mmol), toluene (anhydrous, 4.50 mL, 0.20 M) and EtOH (anhydrous, 2.25 mL, 0.40 M) was heated at 160 °C for 16 h in a high-pressure vessel sealed with a PTFE-lined screw cap. The reaction mixture was allowed to cool to room temperature over 1 h, and the solvents were removed under reduced pressure. Purification by flash column chromatography on silica gel (CH_2Cl_2) afforded di([1,1'-biphenyl]-4-yl)methylene)hydrazine (**S2**) as a colourless solid (250 mg, 0.72 mmol, 80% yield). R_f (SiO_2, CH_2Cl_2) = 0.16; Mp. 178 – 181 °C; ^{1}H NMR (400 MHz, CD_2Cl_2) δ 7.80 (d, J = 8.60 Hz, 2H), 7.69 (d, J = 7.0 Hz, 2H), 7.62 (d, J = 7.1 Hz, 2H), 7.59 – 7.54 (m, 4H), 7.50 (t, J = 7.5 Hz, 2H), 7.45 – 7.39 (m, 5H), 7.36 – 7.32 (m, 1H), 5.55 (s, 2H) ppm; ^{13}C NMR (101 MHz, CD_2Cl_2) δ 148.2, 142.1, 141.0, 140.9, 140.8, 138.1, 132.3, 129.8, 129.3, 129.2, 128.4, 128.1, 127.8, 127.5, 127.3, 127.2, 127.1 ppm; IR (ATR): $\tilde{\nu}$ = 3200, 3363 (N–H), 1486 (C=N) cm^{-1}; HRMS (APCI, positive mode) m/z calcd for $C_{25}H_{20}N_2$: 349.16993 [M+H]$^+$, found 349.16974; Elemental analysis (%calcd, %found for $C_{25}H_{20}N_2$): C (86.17, 86.23), H (5.79, 6.01), N (8.04, 7.97).

Di([1,1'-biphenyl]-4-yl)diazomethane (S3): The compound was handled under exclusion of daylight; all manipulations were carried out under red light ≥600 nm. A mixture of (di([1,1'-biphenyl]-4-yl)methylene)hydrazine (160 mg, 0.46 mmol), anhydrous $MgSO_4$ (66 mg, 0.55 mmol), and CH_2Cl_2 (anhydrous, 9.2 mL, 0.05 M) was cooled to 0 °C under vigorous stirring. Activated MnO_2 (150 mg, 1.72 mmol) was added in one portion, resulting in the immediate formation of a deep purple suspension. The suspension was allowed to warm to room temperature and stirred for 24 h. The resulting mixture was filtered through a plug of activated basic alumina (Brockmann activity I) and the solids were rinsed with a mixture of n-hexane/CH_2Cl_2 1:1 (80 mL) until the filtrate was colorless. The filtrate was concentrated at room temperature by rotary evaporation and stored at 7 °C for a few hours. The formed dark purple needles were filtered off and dried under vacuum to give the analytically pure product (140 mg, 0.40 mmol, 88%). R_f (Al_2O_3 basic, CH_2Cl_2/n-hexane 3:2) = 0.88; Mp. 238 – 241 °C; ^{1}H NMR (400 MHz, THF-d_8) δ 7.71 (d, J = 8.7 Hz, 4H, H4/12), 7.65 (d, J = 8.3 Hz, 4H, H7/11), 7.43 – 7.40 (m, 8H, H1/3/8/10), 7.30 (t, J = 7.3 Hz, 2H, H9) ppm; ^{13}C NMR (101 MHz, THF-d_8) δ 141.1 (C2), 139.3 (C6), 129.4 (C1/3), 129.1 (C5), 128.3 (C4/12), 127.8 (C9), 127.2 (C7/11), 126.1 (C8/10) ppm (consistent with previous reports, the diazomethane carbon gives only a weak signal and was not observed[3]); IR (ATR): $\tilde{\nu}$ = 2024 (N≡N) cm^{-1}; UV/Vis (methylcyclohexane): λ_{max} = 537 nm

(ϵ = 130 $M^{-1}\,cm^{-1}$); MS (MALDI, positive mode) m/z 319.14902 ($[M–N_2+H]^+$, 100%), 665.29577 ($[2M–N_2+H]^+$, 74%); Elemental analysis (%calcd, %found for $C_{25}H_{18}N_2$): C (86.68, 86.79), H (5.24, 5.29), N (8.09, 8.03).

B. NMR spectra

The NMR spectra of compounds S1–S3 are shown in Supplementary Figures 2–13.

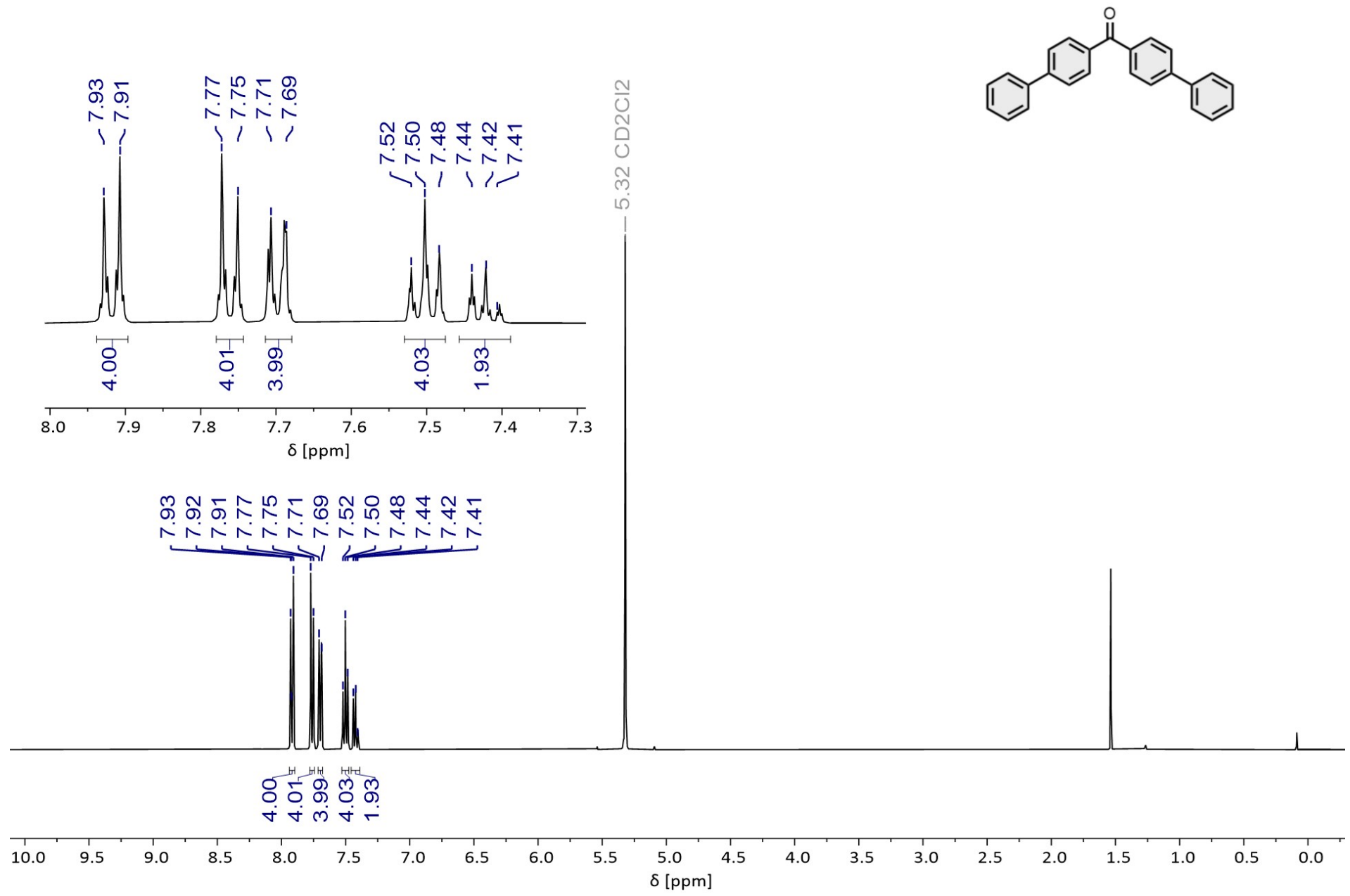

Supplementary Figure 2. ^{1}H NMR spectrum (400.1 MHz, CD_2Cl_2) of compound **S1** recorded at rt.

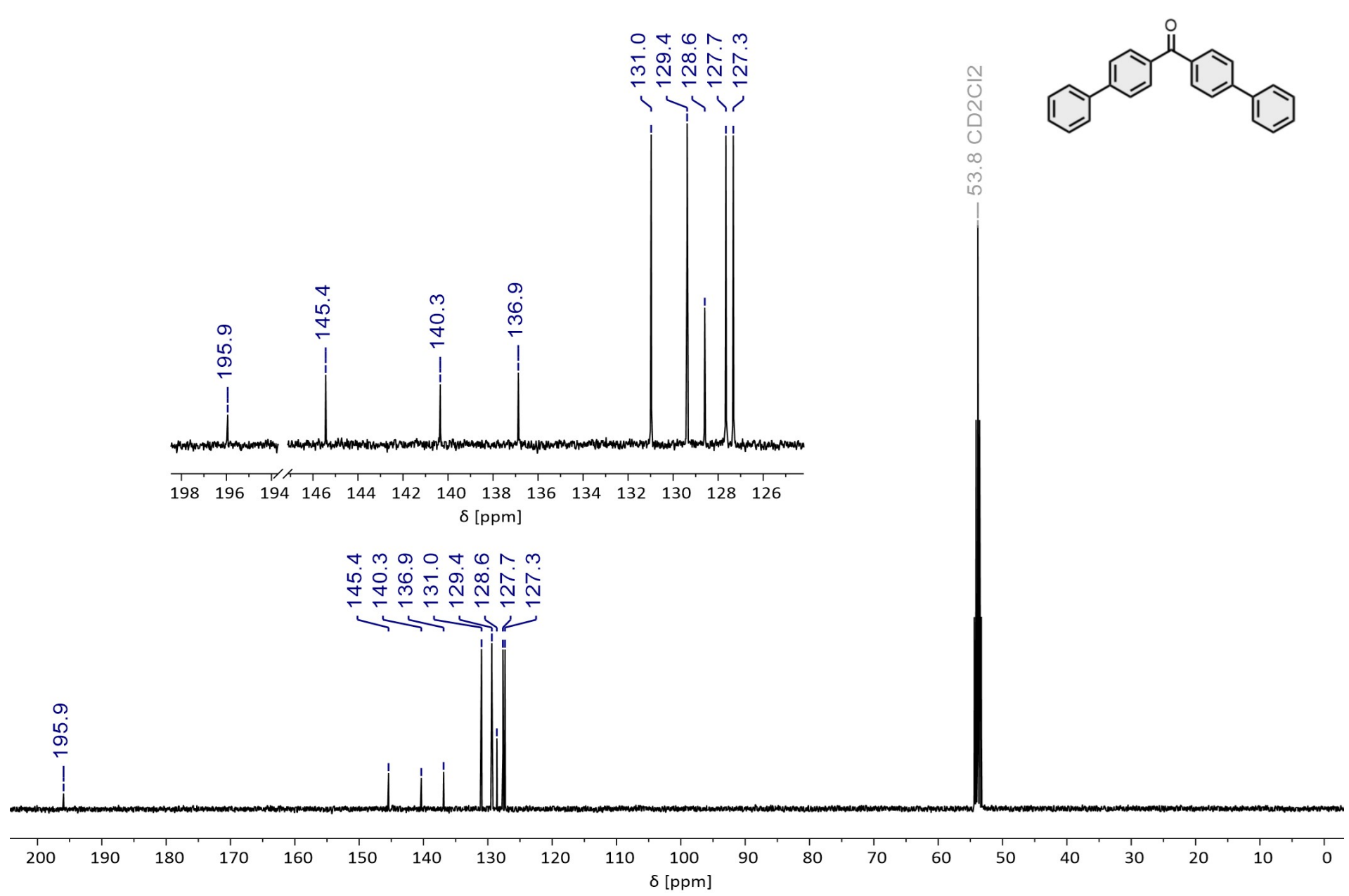

Supplementary Figure 3. ^{13}C NMR spectrum (100.6 MHz, CD_2Cl_2) of compound **S1** recorded at rt.

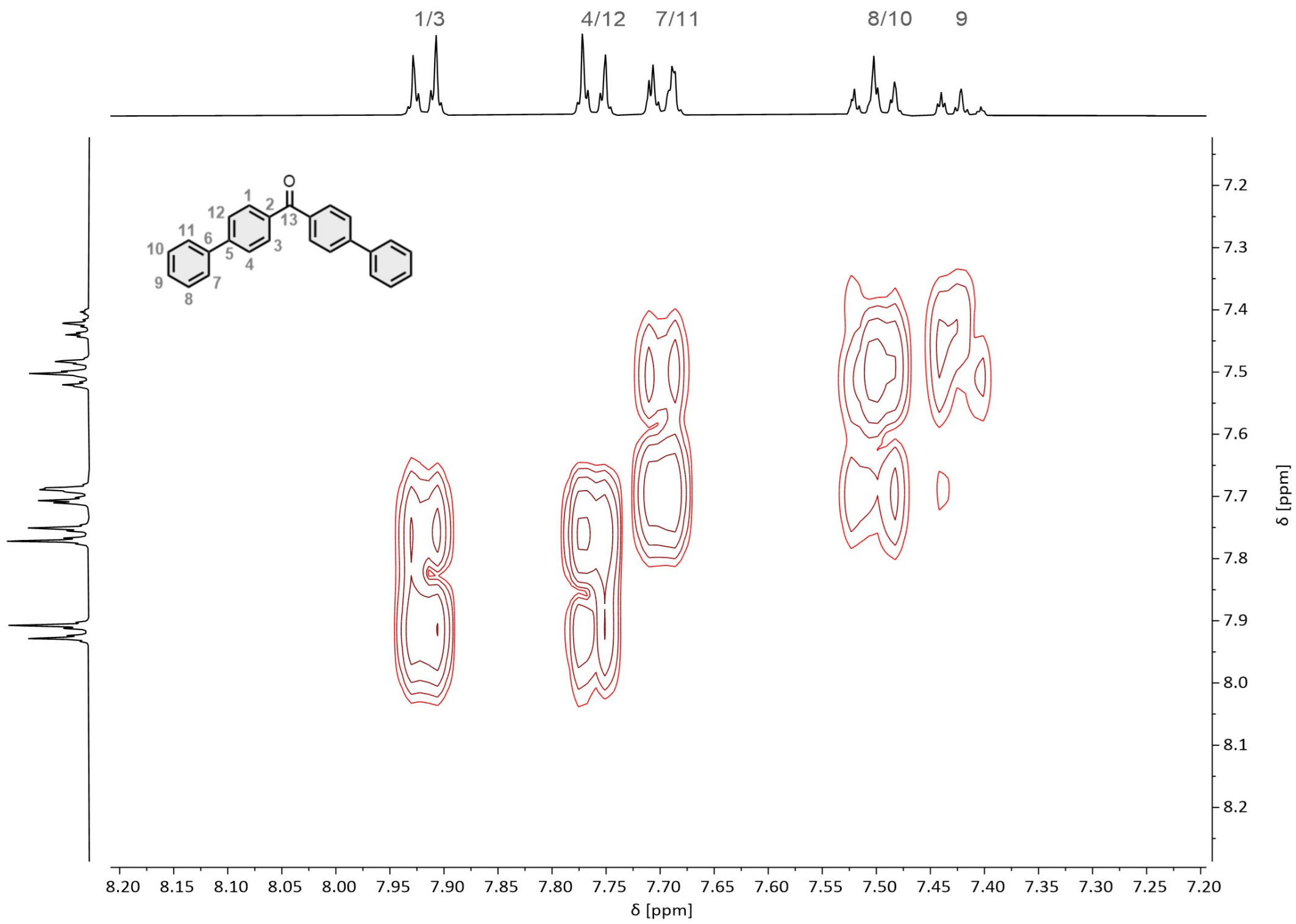

Supplementary Figure 4. Two-dimensional $^{1}H,^{1}H$-COSY NMR spectrum (400.1 MHz, CD_2Cl_2) of **S1** at rt.

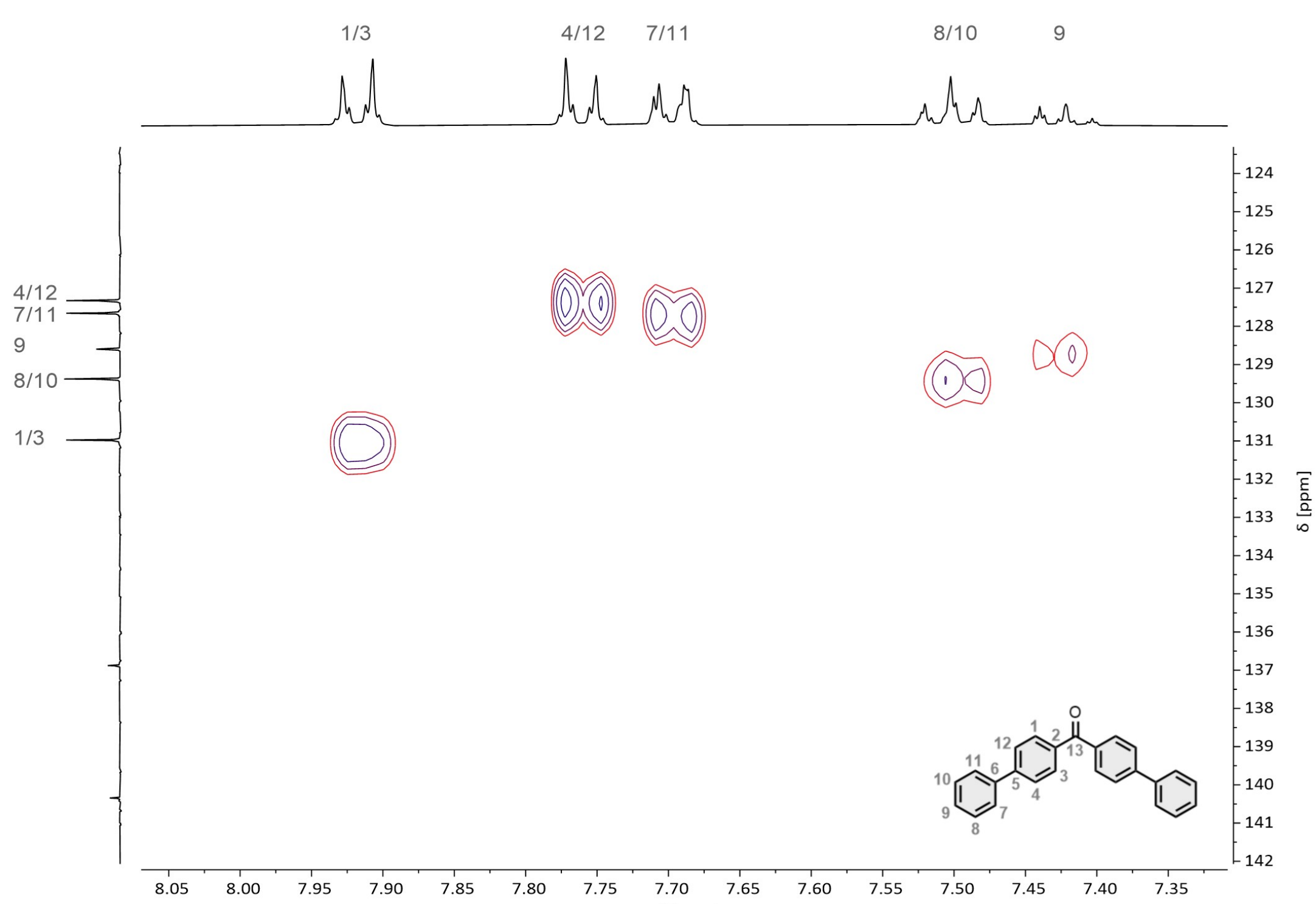

Supplementary Figure 5. 2D $^{1}H,^{13}C$-HSQC NMR spectrum (^{1}H: 400.1 MHz, ^{13}C: 100.6 MHz, CD_2Cl_2) of **S1** rt.

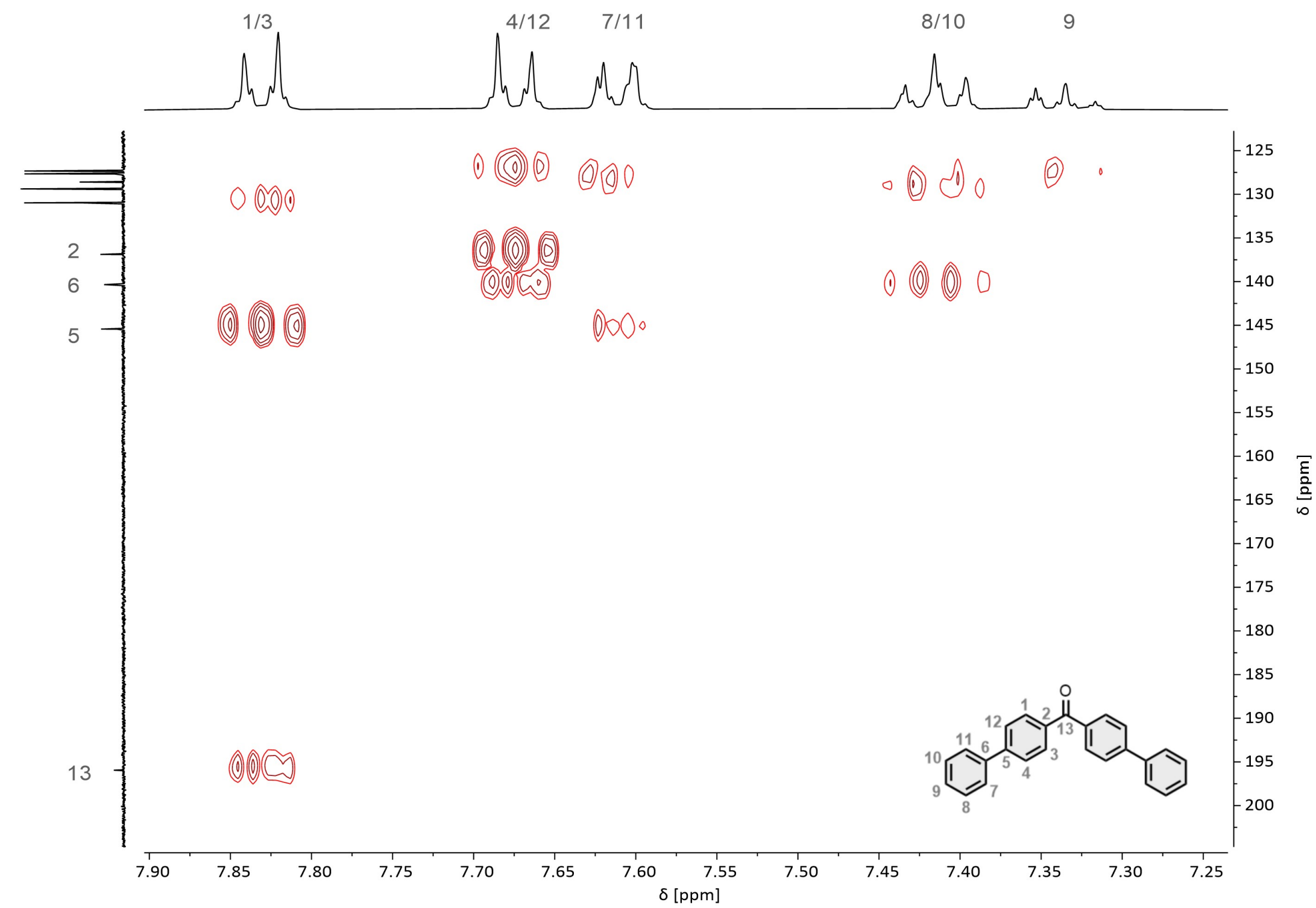

Supplementary Figure 6. 2D $^{1}H,^{13}C$-HMBC NMR spectrum (^{1}H: 400.1 MHz, ^{13}C: 100.6 MHz, CD_2Cl_2) of **S1** at rt.

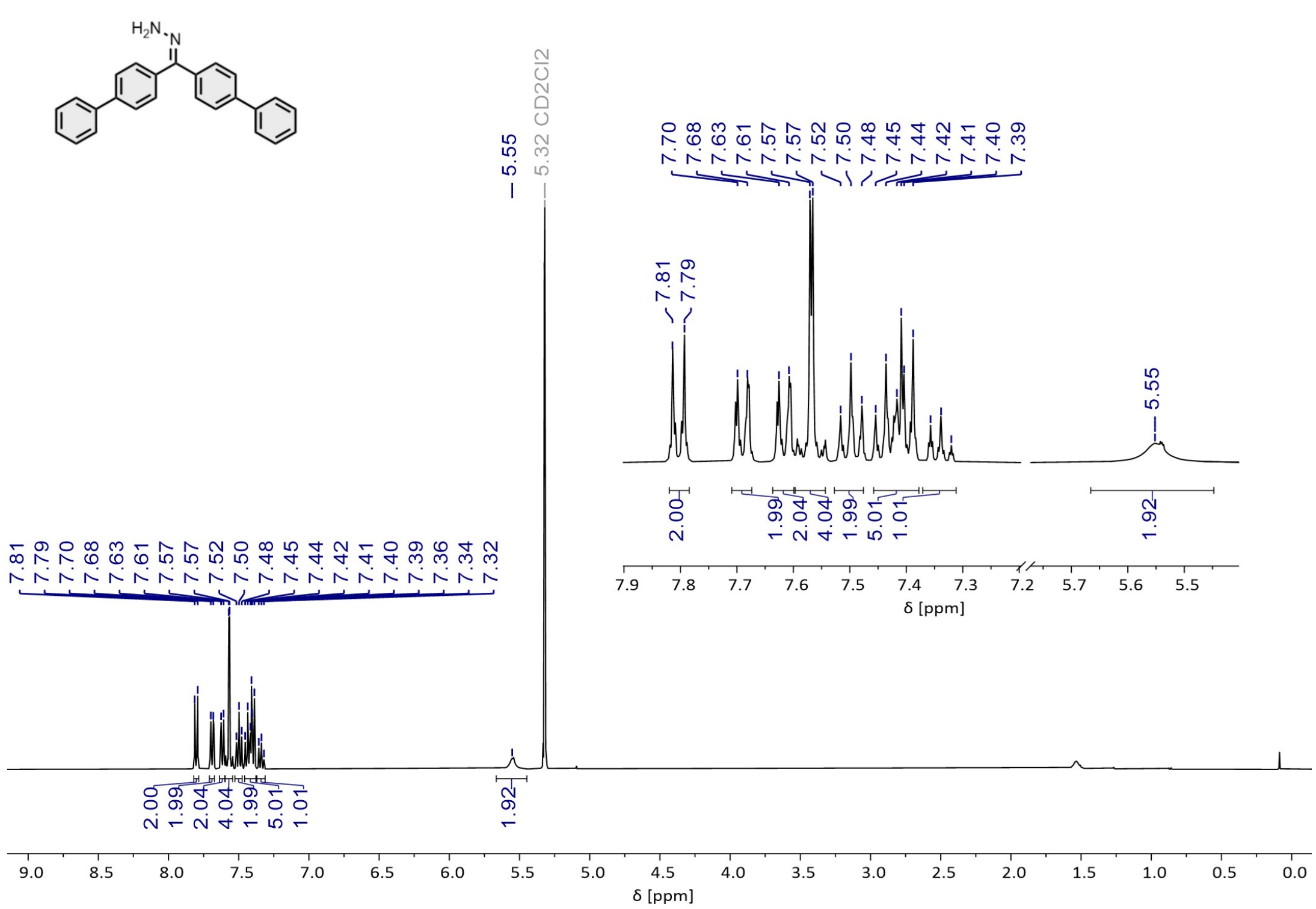

Supplementary Figure 7. ^{1}H NMR spectrum (400.1 MHz, CD_2Cl_2) of compound **S2** recorded at rt.

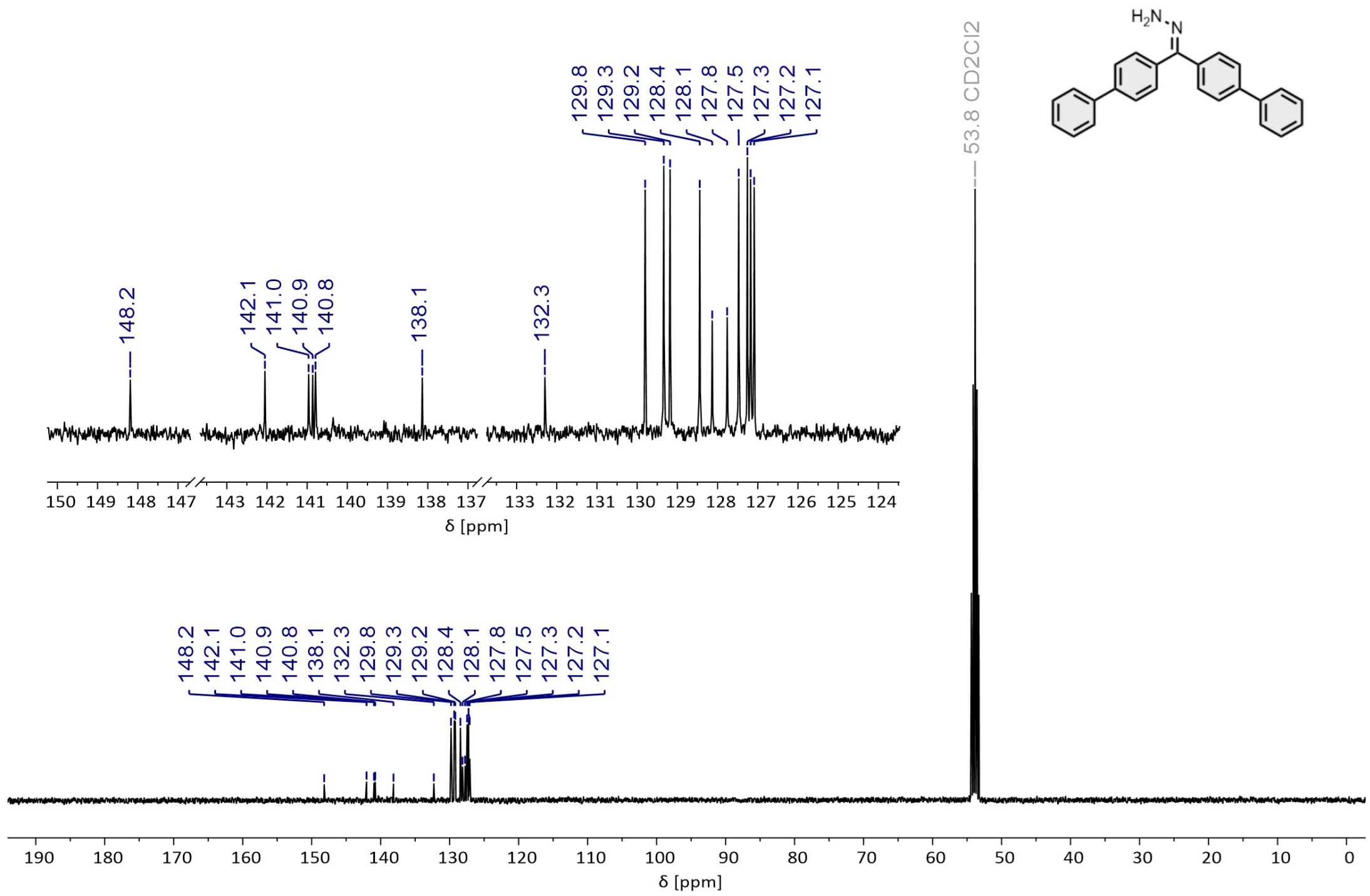

Supplementary Figure 8. ^{13}C NMR spectrum (100.6 MHz, CD_2Cl_2) of compound **S2** recorded at rt.

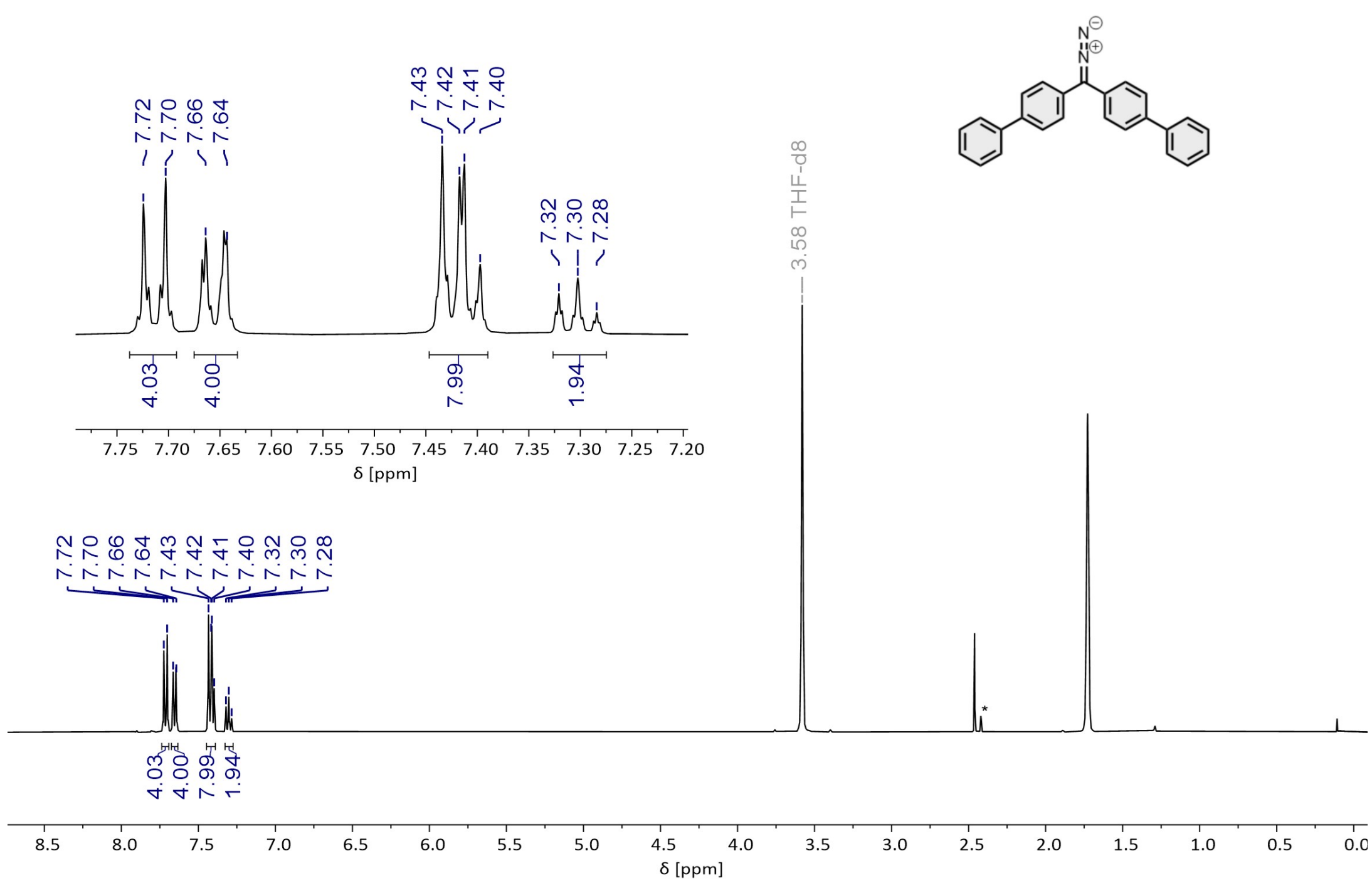

Supplementary Figure 9. ^{1}H NMR spectrum (400.1 MHz, THF-d_8) of compound **S3** recorded at rt; *denotes impurity from solvent.

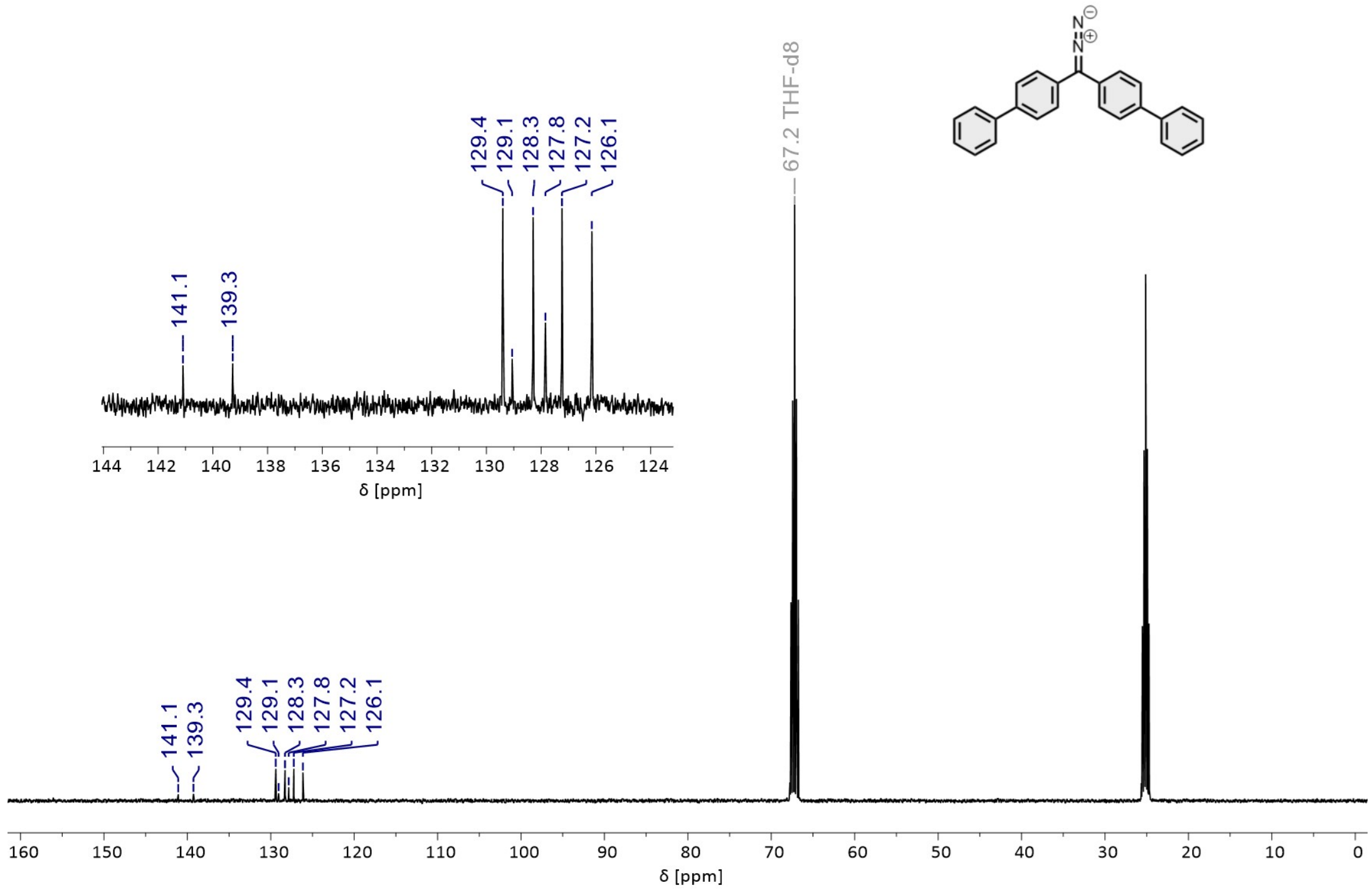

Supplementary Figure 10. ^{1}3C NMR spectrum (100.6 MHz, THF-d_8) of compound **S3** recorded at rt.

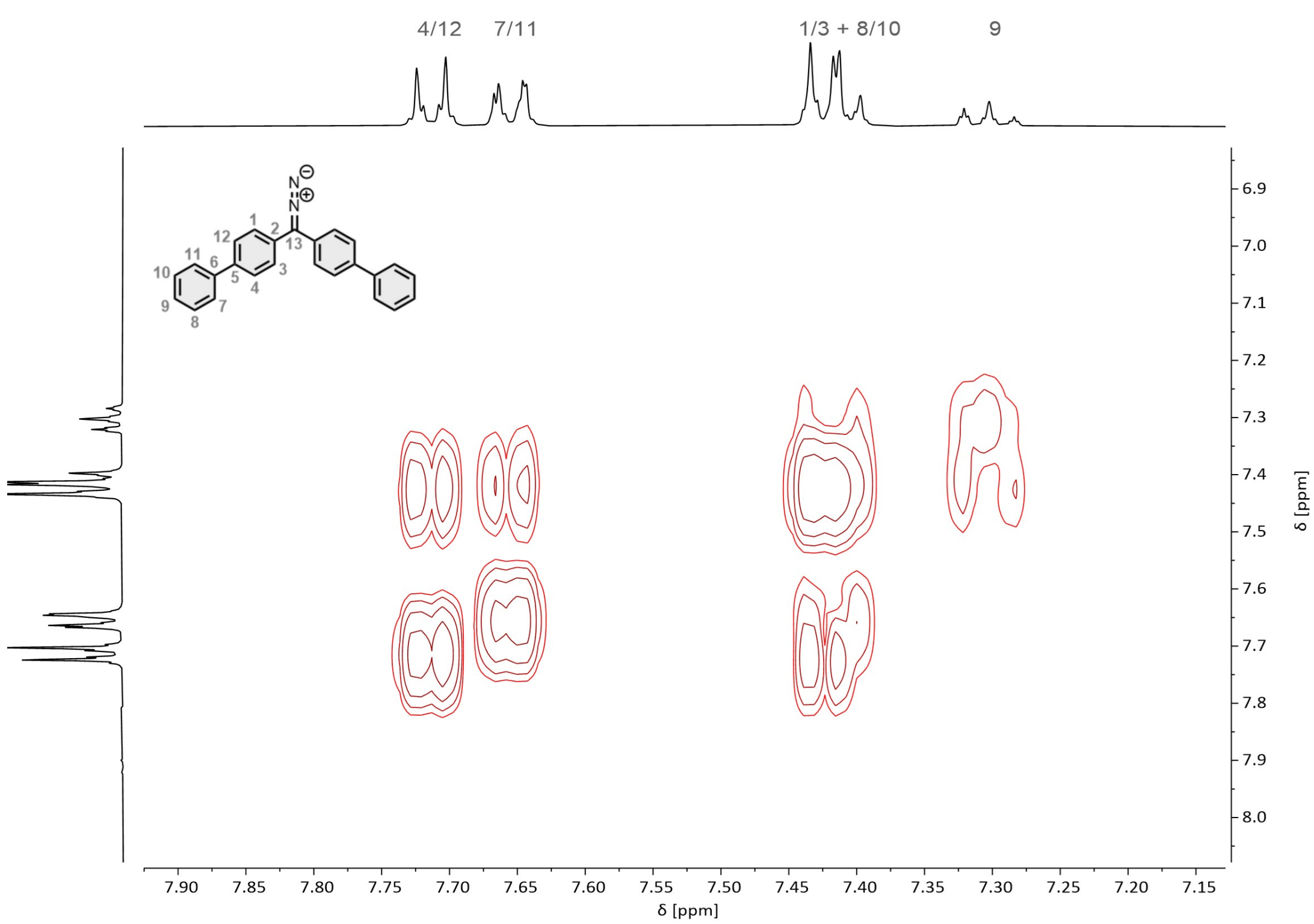

Supplementary Figure 11. Two-dimensional ^{1}H,^{1}H-COSY NMR spectrum (400.1 MHz, THF-d_8) of **S3** at rt.

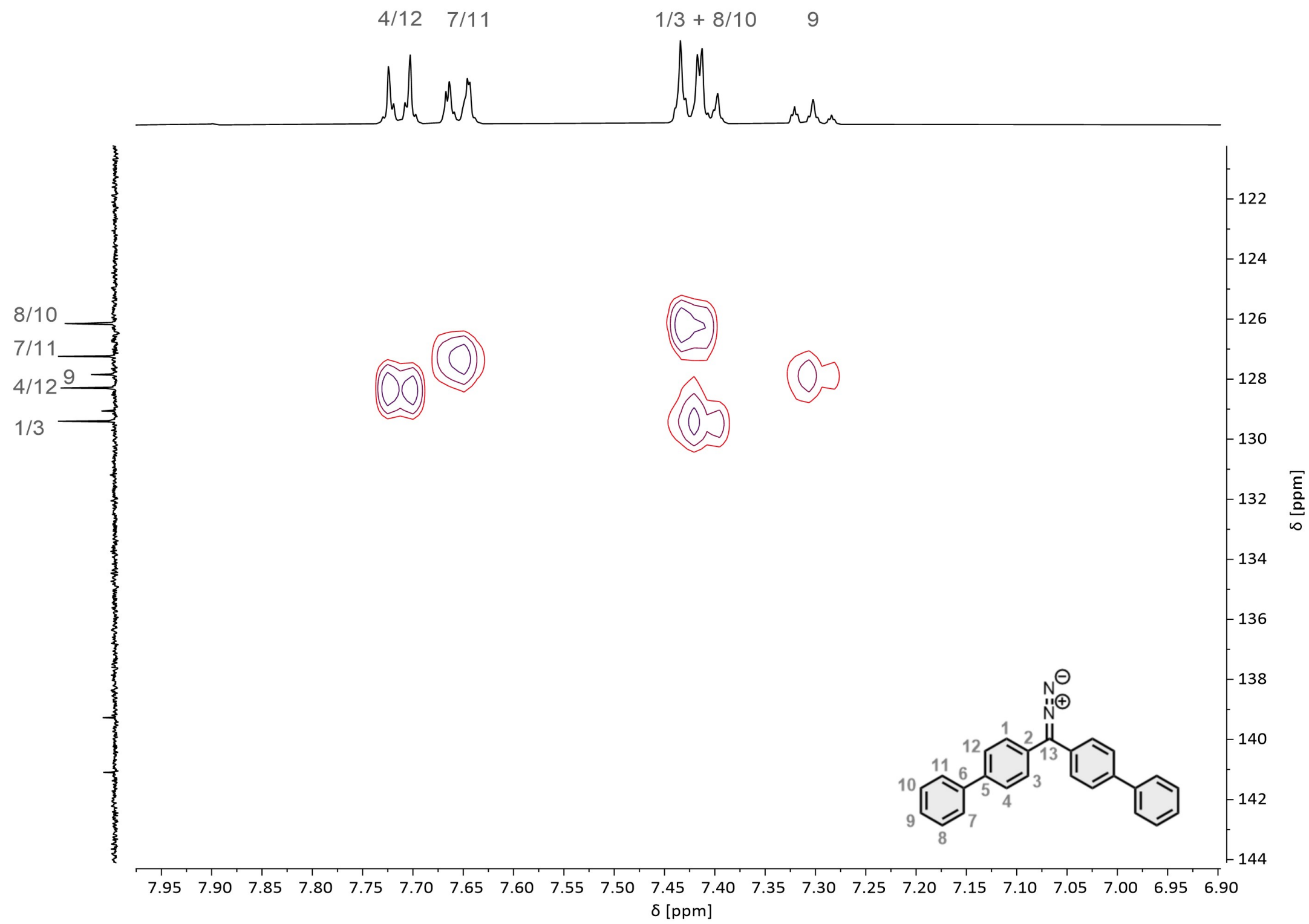

Supplementary Figure 12. 2D ^{1}H,^{13}C-HSQC NMR spectrum (^{1}H: 400.1 MHz, ^{13}C: 100.6 MHz, THF-d_8) of **S3** rt.

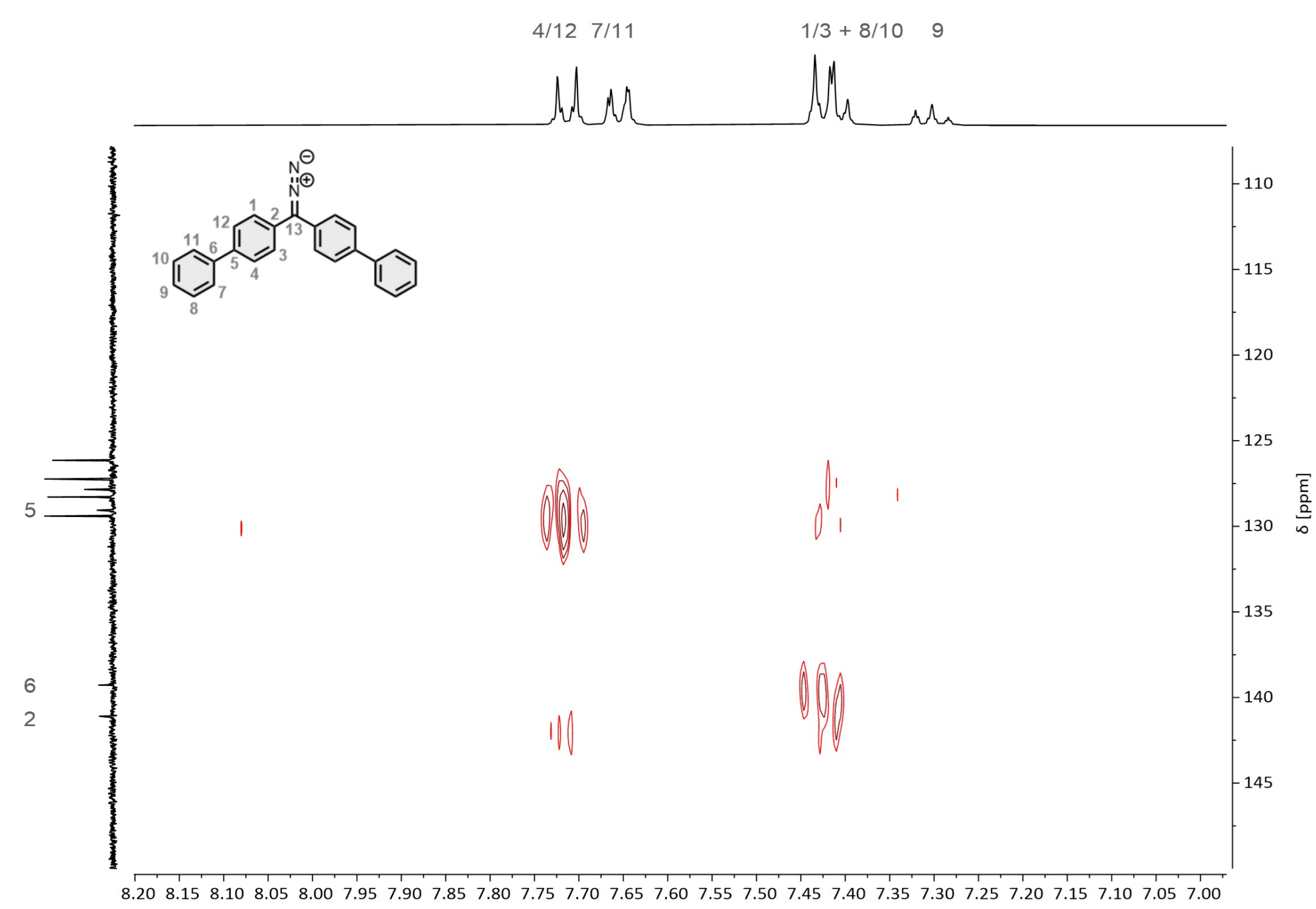

Supplementary Figure 13. 2D ^{1}H,^{13}C-HMBC NMR spectrum (^{1}H: 400.1 MHz, ^{13}C: 100.6 MHz, THF-d_8) of **S3** at rt.

II. MATERIAL FABRICATION AND SAMPLE PREPARATION

A. Growth of doped molecular crystals

Details on the crystal growth can be found in the *Methods* section. The doping ratio ξ, defined as the molar ratio of dopant/matrix was estimated based on the single crystal structure data of the matrix (see section: X-ray crystallographic analysis). Using the cell volume of 1670 Å^3, the molecular volume calculates to 417.5 Å^3. The average distance d (nm) was then estimated as $\xi = 0.4175/d^3$ with ξ being the doping ratio. Supplementary Table 1 shows exemplary average distances.

Supplementary Table 1. Exemplary doping ratios and the resulting average distance.

Ratio ξ (dopant/matrix)	Avg. distance (nm)
0.025	2.6
0.0025	5.5
0.00025	12
0.000025	25

B. X-ray crystallographic analysis

Crystallographic data for the structure reported in this paper have been deposited with the Cambridge Crystallographic Data Centre.[4] CCDC 2542406 contains the supplementary crystallographic data for this paper. These data can be obtained free of charge from The Cambridge Crystallographic Data Centre via https://www.ccdc.cam.ac.uk/structures.

Supplementary Table 2. Crystallographic data for CCDC 2542406 (**S1**).

**Parameter**	**Value**
CCDC number	2542406
Empirical formula	$C_{25}H_{18}O$
Formula weight	334.42
Temperature (K)	150.00(10)
Crystal system	Orthorhombic
Space group (No.)	P b c n (60)
a (Å)	7.3846(1)
b (Å)	6.1526(1)
c (Å)	36.7595(4)
α (°)	90
β (°)	90
γ (°)	90
Volume (Å^3)	1670.15(4)
Z	4
ρ_{calc} (g cm^{-3})	1.330
μ (mm^{-1})	0.614
$F(000)$	706
Crystal size (mm^3)	0.10 × 0.19 × 0.89
Crystal colour	clear light colourless
Crystal shape	needle
Radiation	CuKα (λ = 1.54184 Å)
h,k,lmax	9, 7, 46
Reflections collected	29204
Independent reflections	1740 [$R_{int} = 0.0616$]
Completeness to $\theta = 76.67°$	99.4 %
Abs. corr. T_{min}/T_{max}	0.695, 1.000
Goodness-of-fit on F^2	1.0536
Final R indexes [$I \geq 2\sigma(I)$]	$R_1 = 0.0222$, $wR_2 = 0.0535$
Final R indexes [all data]	$R_1 = 0.0247$, $wR_2 = 0.0546$
Largest peak/hole (e Å^{-3})	0.1179 / -0.1701

This report and the CIF file were generated using FinalCif.[5]

Clear, colorless single crystals suitable for crystallographic analysis were grown by slow gas-phase diffusion of *n*-hexane into a solution of **S1** in THF (0.014 M) at room temperature over several days. A rod-like prismatic crystal was prepared in perfluoroalkylether (viscosity 1800 cSt, CAS: 69991-67-9, ABCR) and mounted on a nylon loop. Data were collected on a Rigaku/Oxford Diffraction SuperNova diffractometer, utilizing Cu K(α radiation (α) = 1.54184 Å) from a microfocus source and an Atlas CCD detector under a stream of nitrogen at 150 K. All data were integrated with CrysAlisPro and a multi-scan absorption correction using SCALE3 ABSPACK was applied.[6] The structure was solved by direct methods with SHELXT 2018/2 and refined by full-matrix least-squares methods against F^2 using SHELXL-2018/3.[7,8] The crystal structure was refined against F^2 using the NoSpherA2[9] implementation within the Olex2 software suite.[10] Unlike conventional independent atom models, in this approach nonspherical atomic form factors were calculated via tailored aspherical electron densities. These densities were generated using a Hirshfeld Atom Refinement (HAR) approach, with the underlying wavefunction calculated at the B3LYP/cc-pVTZ level of theory using the ORCA 5.0 interface. Hydrogen atom positions and anisotropic displacement parameters were refined freely without the use of a riding model, providing a more physically accurate representation of the nuclear positions and bonding density.

Compound **S1** crystallizes in the orthorhombic space group Pbcn (No. 60). The unit cell parameters at 150.0(1) K are a = 7.3846(1) Å, b = 6.1526(1) Å, c = 36.7595(4) Å, with $\alpha = \beta = \gamma = 90°$ and a total cell volume of V = 1670.15(4) Å^3 (Supplementary Table 2). The unit cell contains Z = 4 molecules, while the asymmetric unit contains half of a molecule (Z' = 0.5). This reduction in the asymmetric unit arises because the molecule sits on a special position, specifically a twofold rotation axis (Wyckoff position 4c) passing through the carbonyl group (O001 and C005), as indicated by their site symmetry order of 2.

The unit cell of **S1** shows a tightly packed arrangement in which no π-π interactions between aryl units are present (Supplementary Fig. 14a-c). Instead, we find a herringbone-type arrangement of biphenyls of adjacent molecules

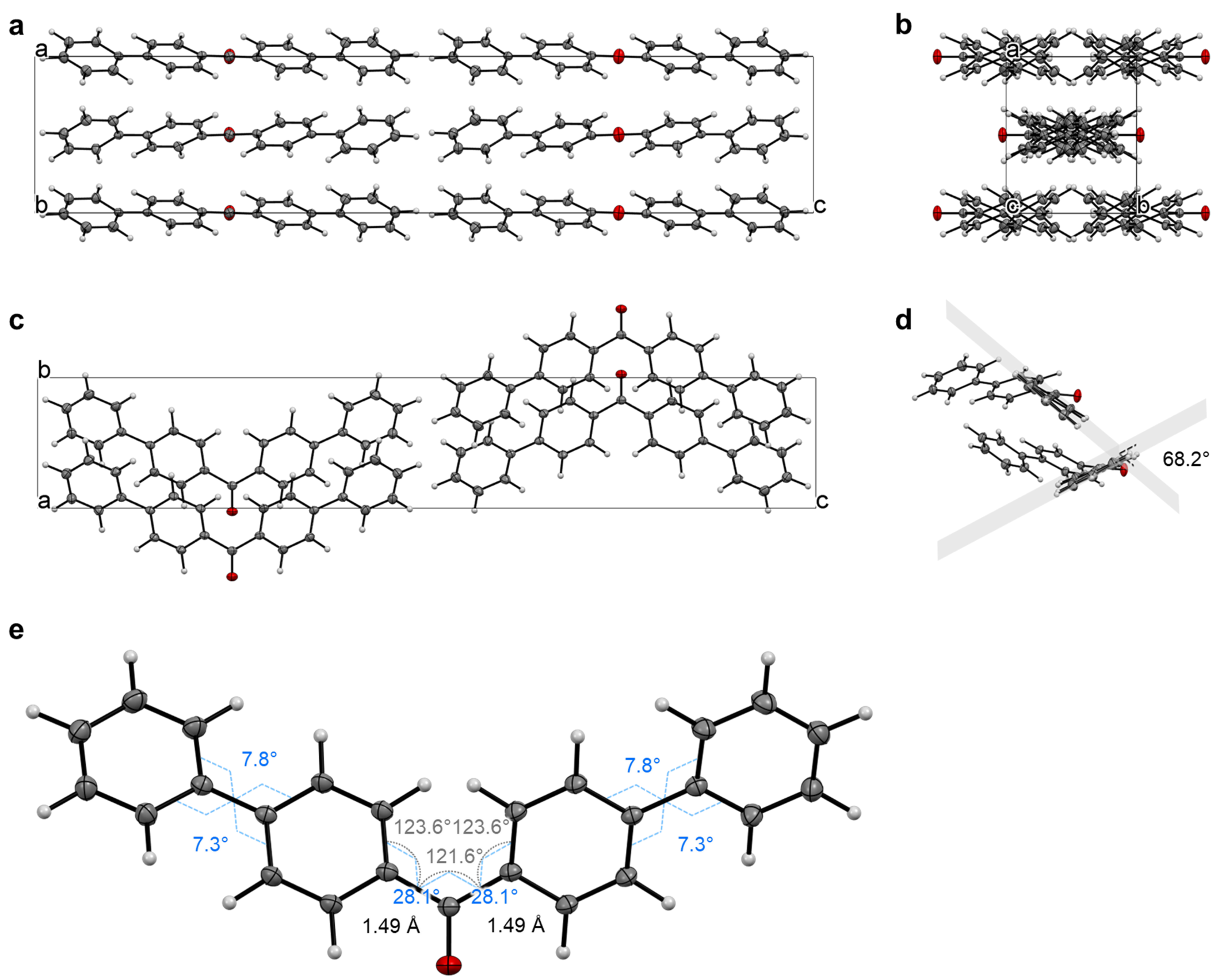

Supplementary Figure 14. Unit cell of **S1** along the b- (a), c- (b), and a-axis (c) at 50% probability level; A pair of neighboring molecules with view along the biphenyl long axis illustrating the dihedral tilt angle of herringbone stacked biphenyl units (d); Molecular structure with selected dihedral angles and the central ketone angle (e).

with a dihedral tilt angle ϕ of 68.2°(Supplementary Fig. 14d) The biphenyl side arms were significantly co-planarized (dihedral angle of 7.3°/7.8°), which we ascribe to crystal packing forces - a feature well known for biphenyl (see e.g. CCDC 1111363,[11] Supplementary Fig. 14e).

The unit cell comprises two pairs of enantiomers (displaying *P* and *M* chirality, Supplementary Fig. 15 denoted by dark and light gray colored atoms, respectively). The molecules C–C(=O)–C plane is slightly tilted relative to the unit cell c-axis by and angle of approximately ±1.73° (blue and red colored rings). Within the crystal lattice, these represent two unique orientations, resulting in an overall difference in orientation of approximately 3.5°.

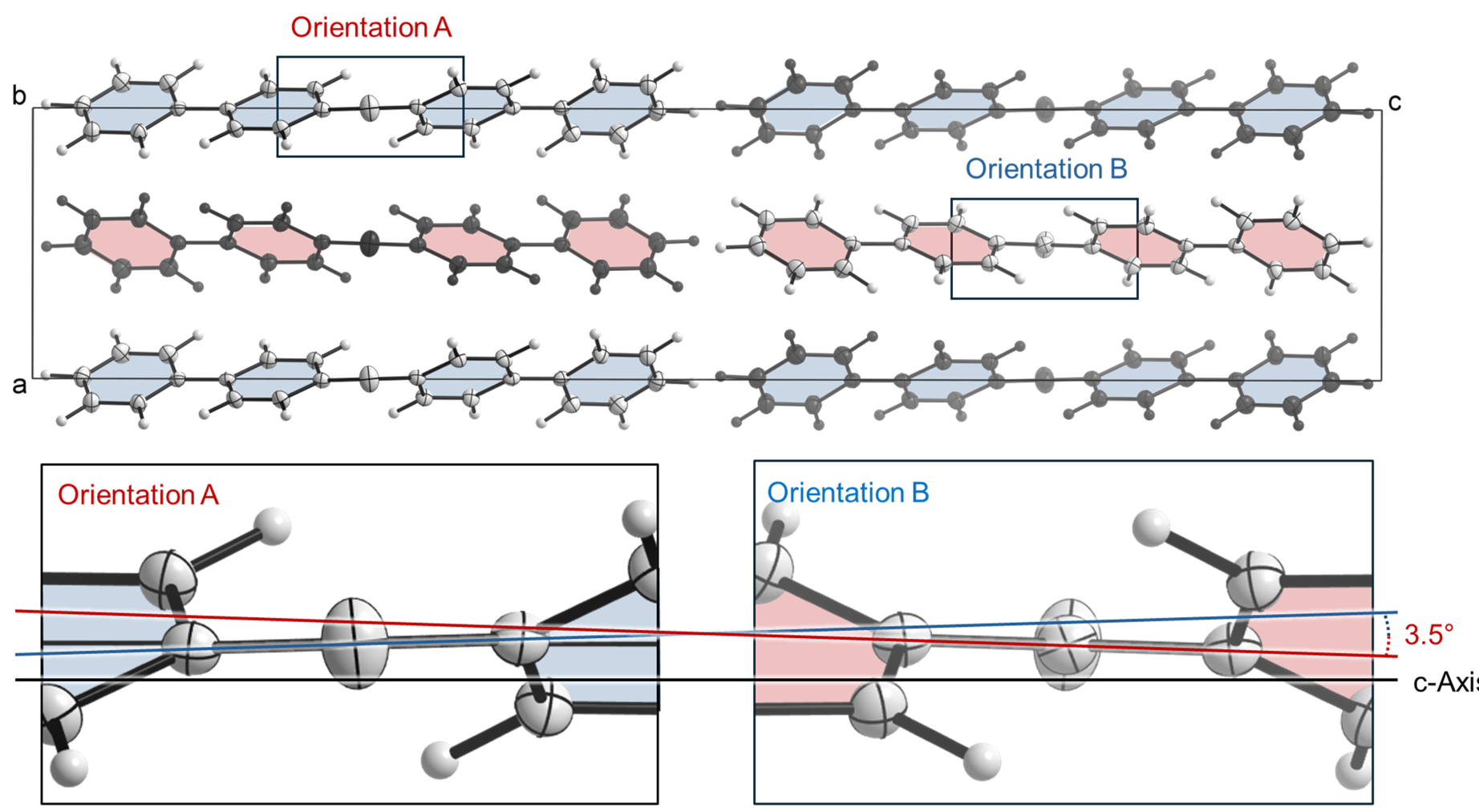

Supplementary Figure 15. Illustration of the structural and orientational relationship of the four matrix molecules in the unit cell; Two pairs of enantiomers, *P*- and *M*-chirality with respect to the ketone aryls (dark and light gray atoms, respectively) and two unique orientations of the molecules (blue and red rings) deviating by an angle of 3.5° with respect to the C=O axis (b-axis of the unit cell, see the insets for a zoom-in).

We analyzed the energy networks of intermolecular interactions using the *CrystalExplorer* package[12] at the B3LYP/6-31(d,p) level of theory to illustrate the cohesion of molecules in different growth directions of the crystal (Supplementary Fig. 16). In agreement with our observations when mechanically manipulating crystals we find strong interactions along the crystallographic a and b directions originating from strong attractive C=O$\cdots$H–C interactions as well as multiple $\pi\cdots$H–C interactions, which in total lead to the edge-to-face arrangement of the biphenyl units as mentioned above. The individual layers of tightly packed matrix molecules in the *ab* plane are held together by multiple weak dispersive van der Waals interaction, which denotes the cleavage plane.

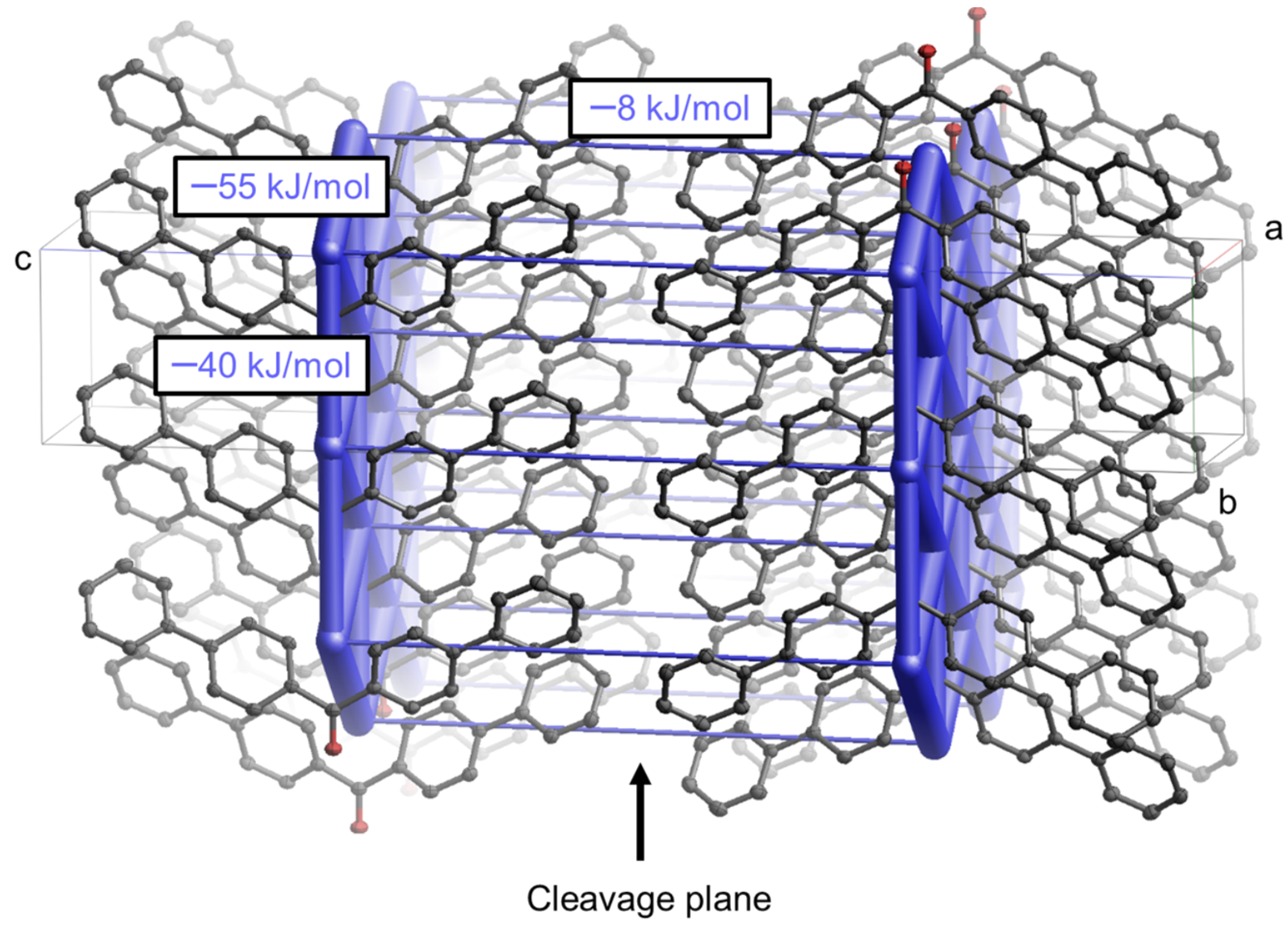

Supplementary Figure 16. Depiction of the energy networks obtained via *CrystalExplorer* program[12] to illustrate the laminar arrangement of the crystal structure of **S1**.

III. OPTICAL SPECTROSCOPY OF BULK MATERIALS AND FROZEN SOLUTIONS

A. Steady-state spectroscopy

Fluorescence spectroscopy was performed using an inhouse-built setup with a LP450-SF25 laserdiode (Thorlabs, 452 nm) and a QEPro spectrometer (OceanOptics) with a 475 nm long pass filter to remove the excitation light from the emission. For that, the corresponding diaryldiazomethane precursor **S3** was dissolved in the respective solvent (optical density OD < 0.1) in a 5 mm NMR tube, containing ca. 0.2 mL solution and was rapidly frozen by plunging

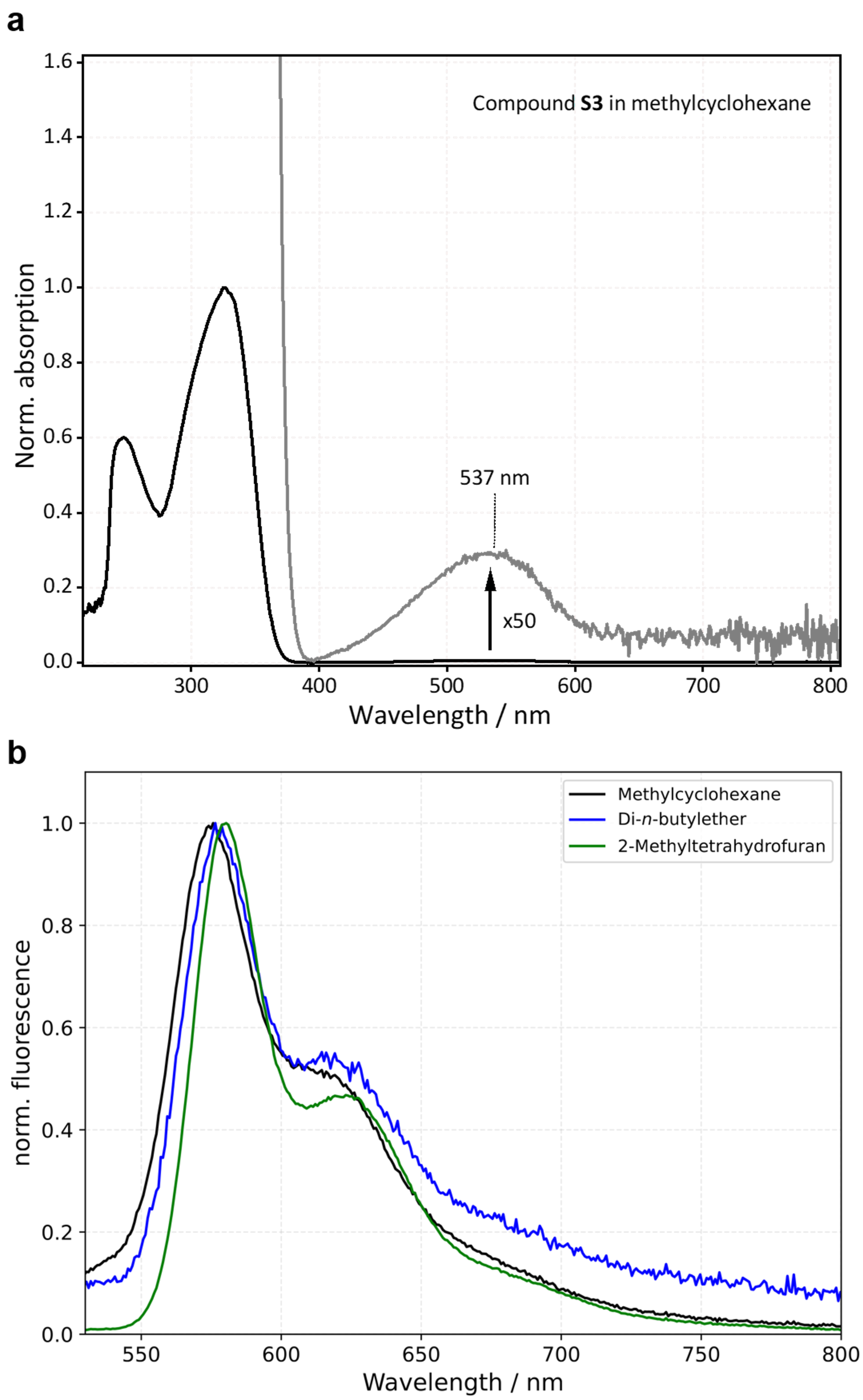

Supplementary Figure 17. Absorption spectroscopy of the diazomethane precursor **S3** in methylcyclohexane at room temperature with the S0 $\rightarrow$ S1 absorption increased (a) and fluorescence spectra of the carbene in frozen methylcyclohexane, di-*n*-butylether and 2-methyltetrahydrofuran at 77 K demonstrating emission from a locally excited state (b).

into liquid nitrogen (77 K). After temperature equilibration (ca. 1 min), photoactivation was carried out using the laserdiode while recording the fluorescence signal.

To investigate solvatochromic effects, three solvents with varying permittivities were selected (methylcyclohexane, di-n-butylether, and 2-methyltetrahydrofuran). Experimental results revealed only a negligible bathochromic shift across the series (575–580 nm, ca. 0.02 eV; see Supplementary Fig. 17). Qualitatively, the use of a frozen solvent medium constrains the ability of the solvent shell to adapt to charge transfer in the excited state, thereby reducing the solvatochromic effect. For the sake of comparison, we refer to the values reported by Nowacki and coworkers,[13] who studied the electron-transfer characteristics of a series of donor-acceptor type chromophores in similar frozen solvents (including methylcyclohexane and 2-methyltetrahydrofuran) and found charge-transfer band shifts of approximately 0.5 eV. This finding allows us to rule out an excited charge-transfer state, which is consistent with our theoretical findings.

B. Photoluminescence quantum yields

Absolute fluorescence quantum yield ϕ were determined in triplicate using a Quantaurus-QY (C11347, Hamamatsu) equipped with a cryo module after excitation at 460 nm. For that, the corresponding diaryldiazomethane precursor **S3** was dissolved in 2-methyltetrahydrofuran (optical density OD < 0.1), the tube containing ca. 0.2 mL solution rapidly frozen by plunging into liquid nitrogen (77 K). After temperature equilibration (ca. 1 min), quantitative photoactivation was carried out using a UV LED curing lamp (Jupitertech, (12.0 W/cm^2, distance ca. 3 cm) with a central wavelength of 395 nm within 1 min. The carbene molecules show a photoluminescent quantum yield of 0.09(1). The PLQY of our doped molecular crystals was also assessed at 4.5 K to better reflect actual operating conditions utilizing excitation saturation measurements (see Sec. VII F).

C. Time-correlated single photon counting

Time correlated single photon counting (TCSPC) experiments were performed using an inhouse-built setup with a pulsed DLnsec laser (LABS electronics) with 520 nm or 450 nm wavelength excitation wavelength. For filtering the excitation light from the emission, either a 550 nm or a 475 nm long pass filter was used in front of an avalanche photodiode SPCM-AQRH (Excelitas). The pulse trigger and photon time tagging was done by using an OPX1000 (Quantum Machines). The samples were placed in a 5 mm NMR tube, cooled down to 77 K and then photoactivated using a UV LED curing lamp (Jupitertech, (12.0 W/cm^2, distance ca. 3 cm) with a central wavelength of 395 nm within 30 seconds. Measurements were performed on photoactivated di([1,1'-biphenyl]-4-yl)carbene in frozen 2-methyltetrahydrofuran, as well as on carbenes generated within di([1,1'-biphenyl]-4-yl)methanone host crystals by photoactivating diazomethane precursors at varying dopant densities. The 2-methyltetrahydrofuran sample was degassed by gently bubbling argon through the solution for 10 min prior freezing. The results from the 2-methyltetrahydrofuran measurements show an overlap of two exponential decays with timescale 4.4 ns and 27.3 ns, and respective relative amplitudes of 0.43 and 0.57 (see Supplementary Fig. 18). The fluorescence lifetime measurements on crystal samples is shown in Supplementary Fig. 19, and the fit results in Supplementary Table 3.

The TCSPC data of the doped molecular crystals reveals a bi-exponential fluorescence decay. The long-lifetime component ($\tau_2 \approx 22.3 - 26.2$ ns) observed in the ensemble measurements aligns well with the excited-state lifetime of the $T_{1y'}$ state measured at the single-molecule level via cryo-confocal microscopy (24 ± 2 ns, see Sec. VII L). Based on this, we tentatively assign the short-lifetime component ($\tau_1 \approx 3.9 - 5.1$ ns) to the $T_{1z'}$ state with a dominant decay via inter-system crossing (ISC). However, this assignment could not be definitively verified through single-molecule fluorescence lifetime measurements since spin-selective optical excitation for this state ($T_{0z} \rightarrow T_{1z'}$) could not be observed for single molecules.

In systems where short-range intermolecular interactions are prevalent, elevated doping concentrations typically induce a pronounced shortening of the excited-state lifetime.[14,15] In contrast, the fitted lifetimes (τ_1 and τ_2) in our system remain constant across a broad range of dopant concentrations, spanning from 0.0025% to 0.25%. Only at the highest doping level investigated we observe a marginal decrease in lifetime, which likely signifies the onset of concentration quenching driven by reduced intermolecular distances. This constant lifetimes at lower doping rates indicate that the carbene molecules are well-isolated within the ketone host matrix, and the observed emission kinetics are driven purely by intrinsic monomolecular photophysics rather than intermolecular crosstalk.

Interestingly, while the lifetimes remain largely constant, the pre-exponential amplitudes exhibit a concentration dependence. As the doping ratio decreases to 0.0025%, the relative amplitude of the short-lived component (A_1) increases from 0.43 to 0.62, while the long-lived component (A_2) correspondingly decreases from 0.57 to 0.37 (see

Supplementary Table 3). While we cannot explain this trend conclusively at this point, it may hint at a concentration-dependent inner filter effect (radiation trapping) in which the short timescale component is suppressed by repeated absorption/re-emission processes eventually funneling more photons into the more emissive long lived component at high doping rates.

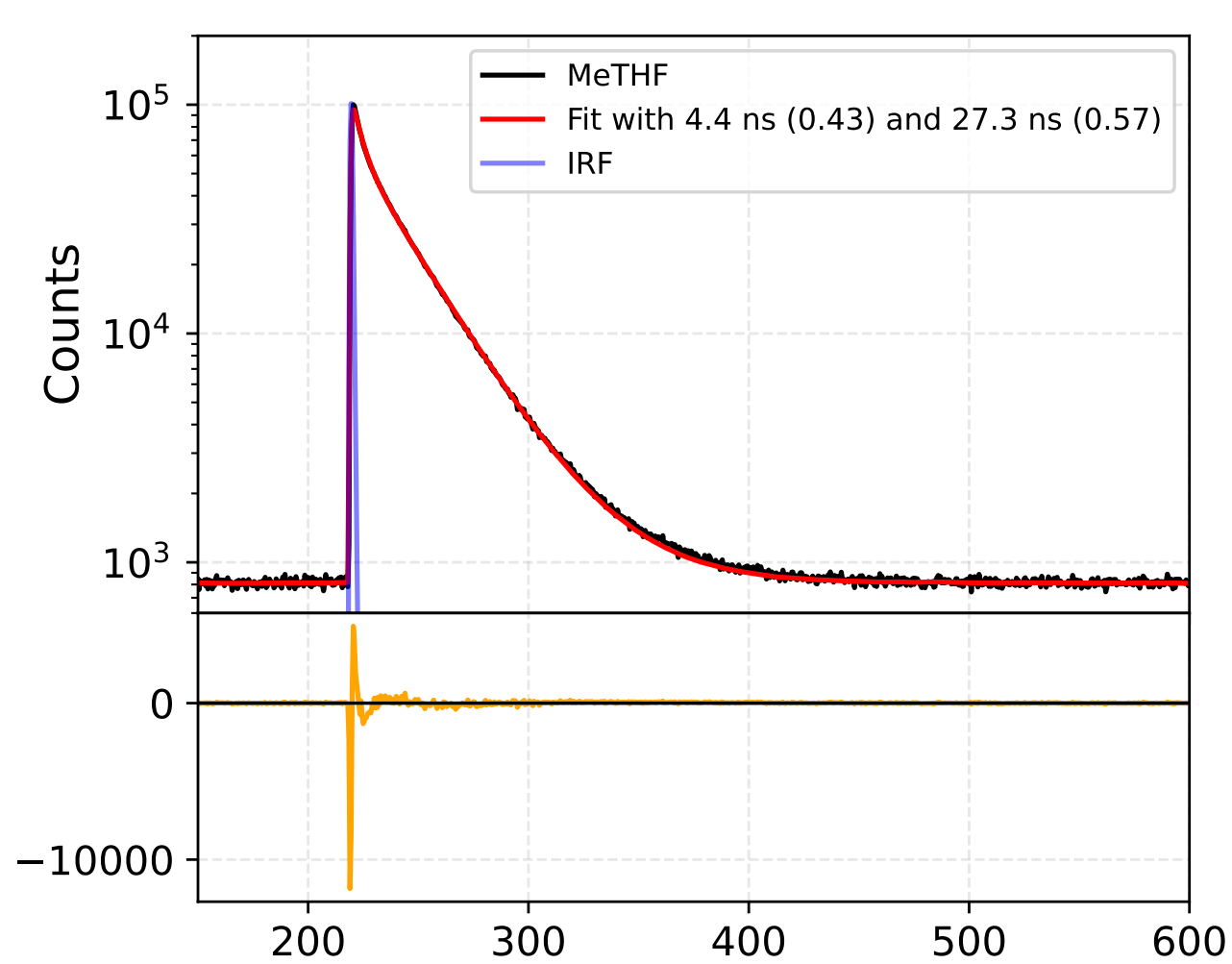

Supplementary Figure 18. TCSPC measurement with reconvolution fit of activated carbene in 2-methyltetrahydrofuran at 77 K. The orange graph denotes the residuals of the fit.

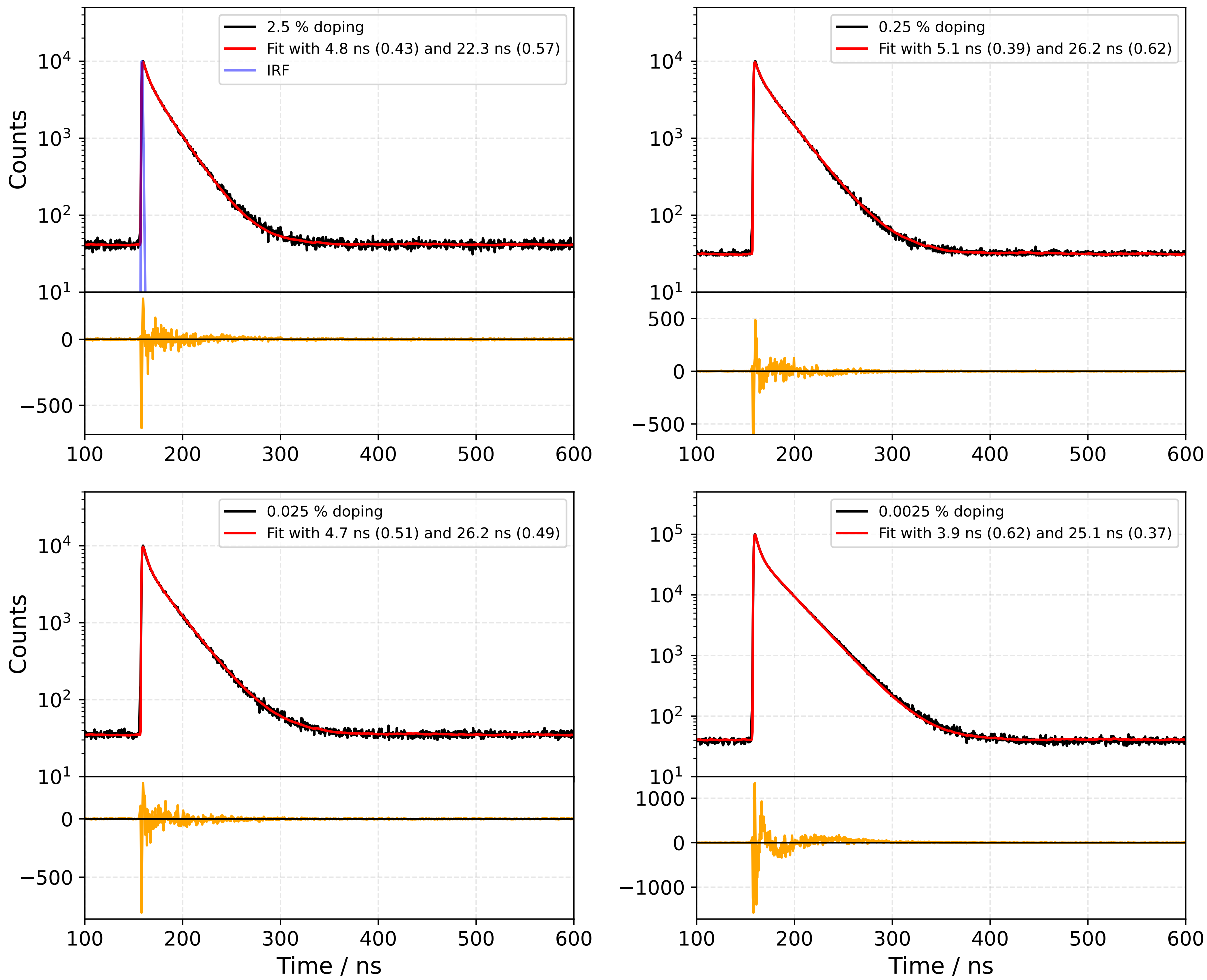

Supplementary Figure 19. TCSPC measurements with reconvolution fits of activated doped crystals with different doping rates at 77 K. The orange graph denotes the residuals of the fit.

Supplementary Table 3. Fluorescence lifetimes (τ) and relative amplitudes (A) for different doping concentrations from reconvolution fits of TCSPC data at 77 K.

**Concentration**	$\boldsymbol{\tau_1}$ **(ns)**	$\boldsymbol{A_1}$	$\boldsymbol{\tau_2}$ **(ns)**	$\boldsymbol{A_2}$
2.5 %	4.8	0.43	22.3	0.57
0.25 %	5.1	0.39	26.2	0.62
0.025 %	4.7	0.51	26.2	0.49
0.0025 %	3.9	0.62	25.1	0.37

IV. COMPUTATIONAL CHEMISTRY

A. Computational methods

ORCA output files for multiscale ONIOM geometry optimization as well as TD-DFT and CASSCF/NEVPT2 single point calculations of the final qubit can be found under `https://doi.org/10.6084/m9.figshare.31963335`.

To validate the suitability of the B97-3c functional for geometry prediction within the ONIOM framework, we first optimized the structure of di([1,1'-biphenyl]-4-yl)carbene in vacuum in the triplet ground state and compared it to results obtained at the B3LYP/def2-TZVPP level. The latter has been shown, both in our experience and by others,[16] to provide reliable geometries (Supplementary Fig. 20). The predicted structures are nearly identical, with negligible deviations in overall shape or key geometric parameters, including bond lengths, dihedral angles, and the carbene angle.

Following the experimental sequence of doping and subsequent photoactivation to generate the activated carbene, the ONIOM calculations were analogously performed in two successive steps. First, the geometry of the closed-shell singlet diazo precursor was optimized (Supplementary Fig. 21). We note that it is important to use nonspherical atomic form factors within the NoSpherA2 extension[9] of Olex2 during structure refinement to ensure the crystallographic data is suitable for generating model clusters without computational post-treatment of hydrogens.[10] In light of recent *in-situ* X-ray diffraction studies on halogenated diarylcarbenes[17] we then proceeded on the assumption that the nitrogen is trapped in the crystal lattice in close vicinity to the carbene center after photoactivation. Hence, the relaxed structure of the diazomethane precursor in the matrix was used to model the system after photoactivation, *i.e.* the resulting carbene in the triplet ground state and the closeby nitrogen molecule embedded in the matrix (T_0, Supplementary Fig. 22a-d). For that, the nitrogen was cleaved off manually by mimicking the out-of plane distorted conical intersection for the photochemical nitrogen dissociation upon excitation to S_2 as reported recently, and geometry optimization was performed.[18]

The resulting relaxed cluster was used to extract the coordinates of the final qubit in the triplet ground state (see Supplementary Fig. 22d). We note that this specific model setup limits geometric relaxation strictly to the qubit molecule and the first matrix shell and does not account for longe-range rearrangements within the matrix.

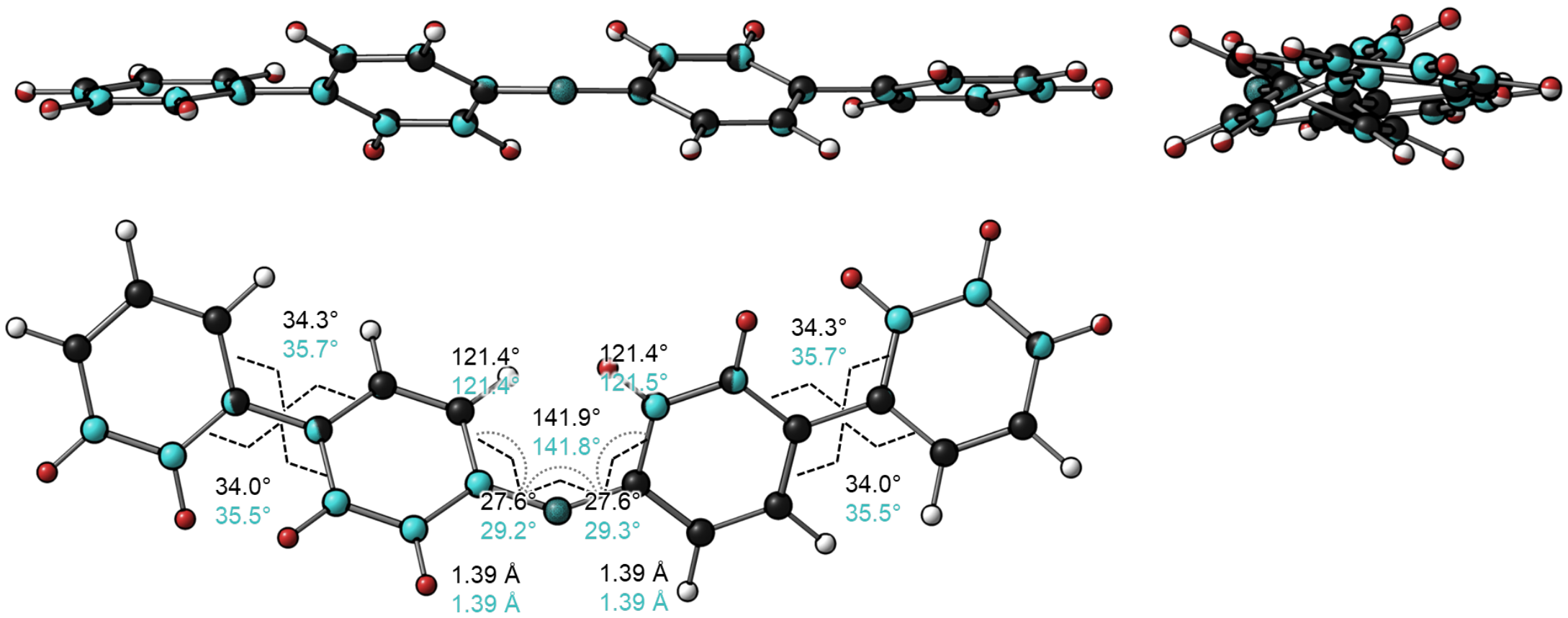

Supplementary Figure 20. Merged geometries of DFT-optimized structures of di([1,1'-biphenyl]-4-yl)carbene for the triplet ground state configuration at the B3LYP/def2-TZVPP (black carbons, white hydrogens) and B97-3c/def2-mTZVP (turquoise carbons, red hydrogens) level of theory calculated in vacuum; root-mean-square-deviation of the atomic positions is 0.0321 Å.

B. Precursor ground state geometry

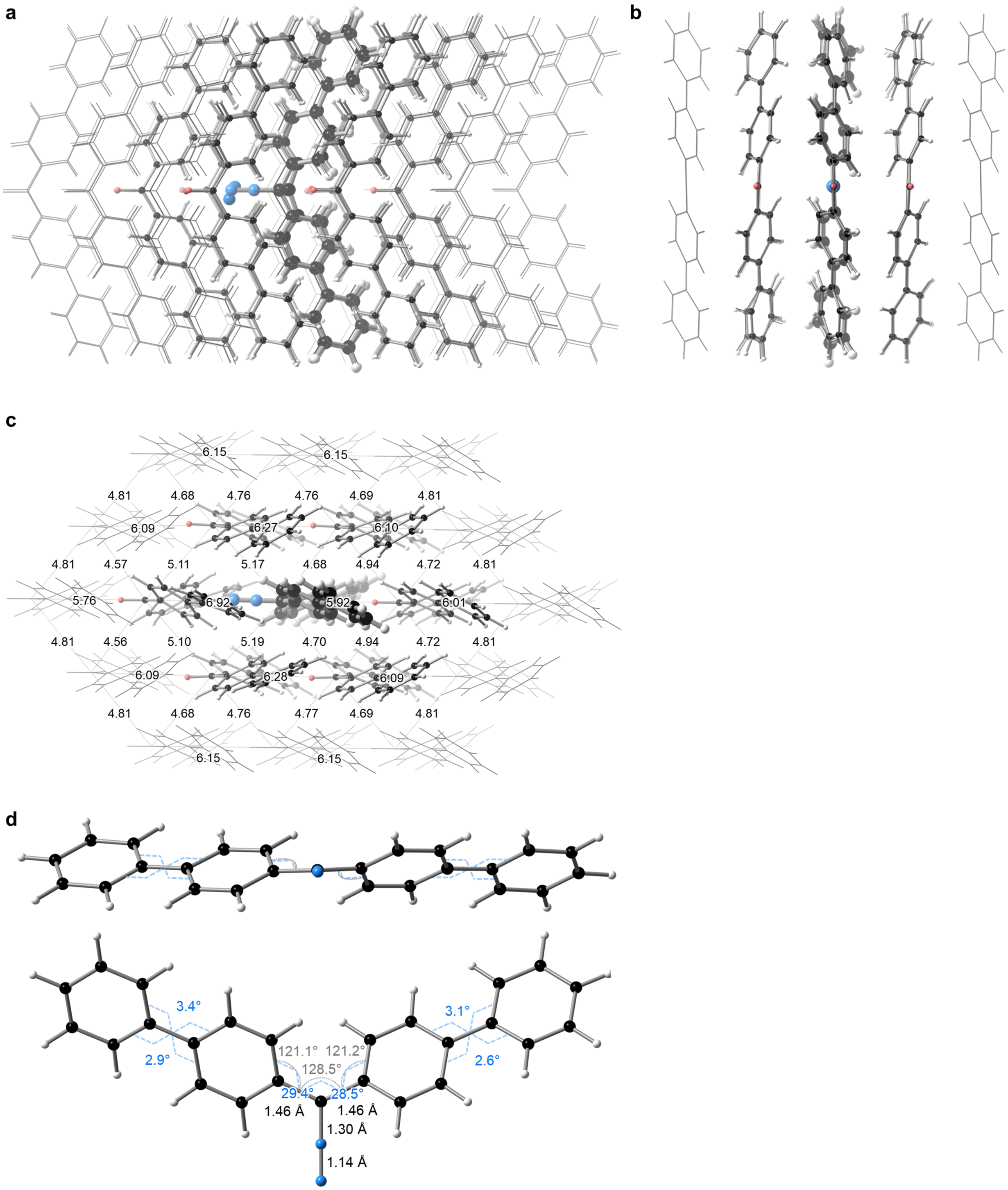

Supplementary Figure 21. Illustration of the multiscale model for the carbene precursor consisting of 837 atoms; The high-level QM region including a diazomethane derivative (bold) and the surrounding shell of six matrix molecules treated at the B97-3c level of theory, and an outer shell of matrix molecules with fixed coordinates (12 molecules, wireframe) treated using semiempirical extended tight-binding method, GFN2-xTB (denoted as XTB2;[19] Top (a), front (b), and side view with distances (in Å) of the ketone and diazomethane carbons to illustrate the structural rearrangement in the first molecular shell around the carbene precursor (c); Molecular structure of diazomethane derivative isolated from the multiscale calculations together with relevant structural data (d).

C. Triplet ground state geometry (T_0)

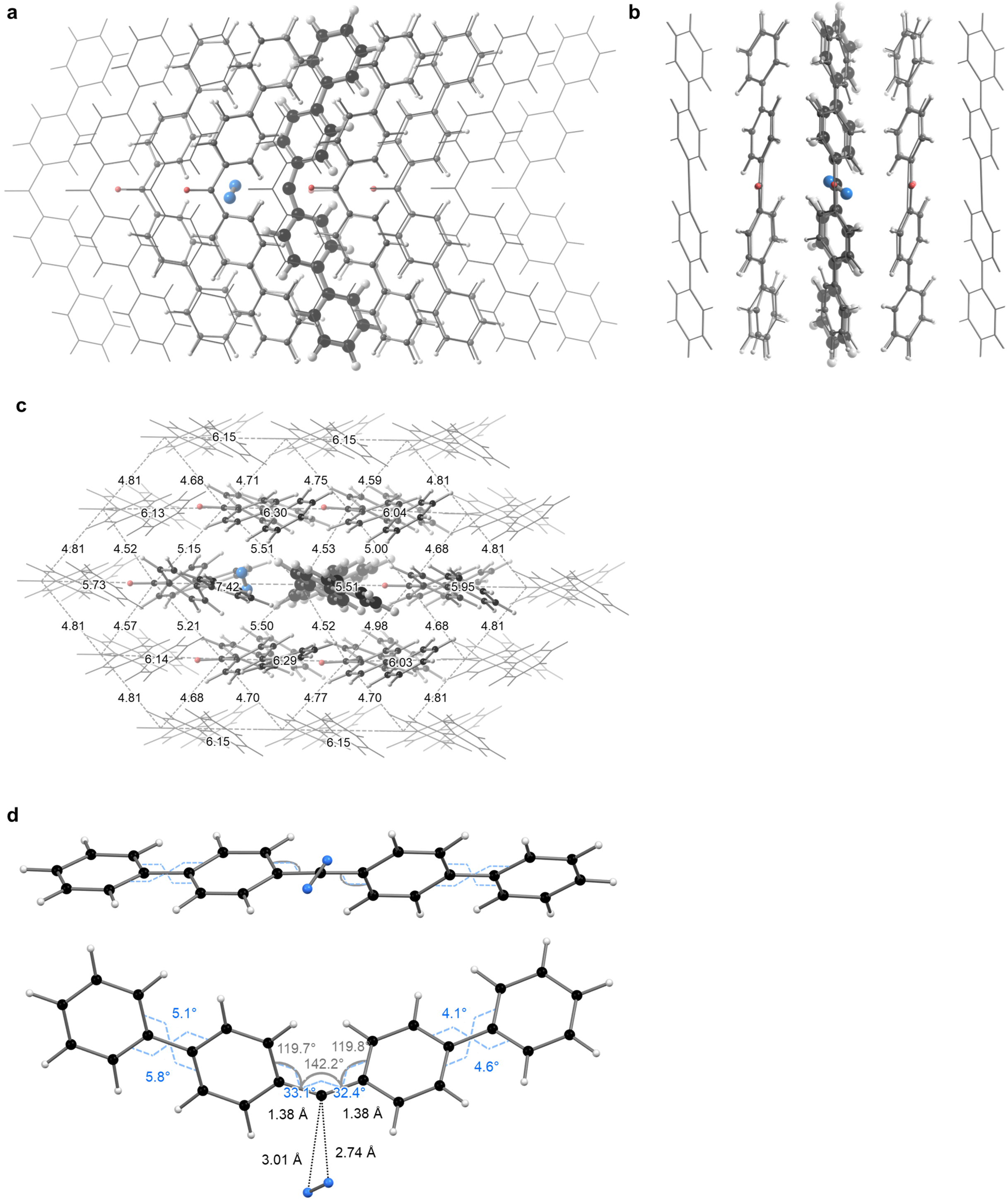

Supplementary Figure 22. Illustration of the multiscale model of the qubit in T_0 geometry; The high-level QM region (carbene and N_2, bold) and surrounding shell of six matrix molecules (ball-and-stick) treated at the B97-3c level of theory and an outer shell of 12 matrix molecules with fixed coordinates (wireframe) computed using semiempirical extended tight-binding method, GFN2-xTB (denoted as XTB2;[19] Top (a), front (b), and side view with distances (in Å) of the central ketone and carbene carbons to illustrate the structural rearrangement in the first molecular shell around the qubit (c); Molecular structure of the carbene + N_2 in T_0 geometry isolated from the multiscale calculations together with relevant structural data (d).

D. Excited state geometry in vacuum (T_1)

When optimized in vacuum, the carbene becomes considerably more coplanar with respect to both the central carbene and biphenyl dihedral angles (see Supplementary Fig. 23). Interestingly, the specific arrangement of our molecular matrix in the crystalline state pre-orients the biphenyl groups into a conformation closely resembling the excited-state geometry (see the dihedral angles in Supplementary Fig. 22d). We assume that this preorientation is important for the pronounced ZPL observed in the cryo confocal measurements and that the intrinsic tendency of the central carbene unit to co-planarize in the excited state is effectively counteracted by the rigid environment of the crystal lattice.

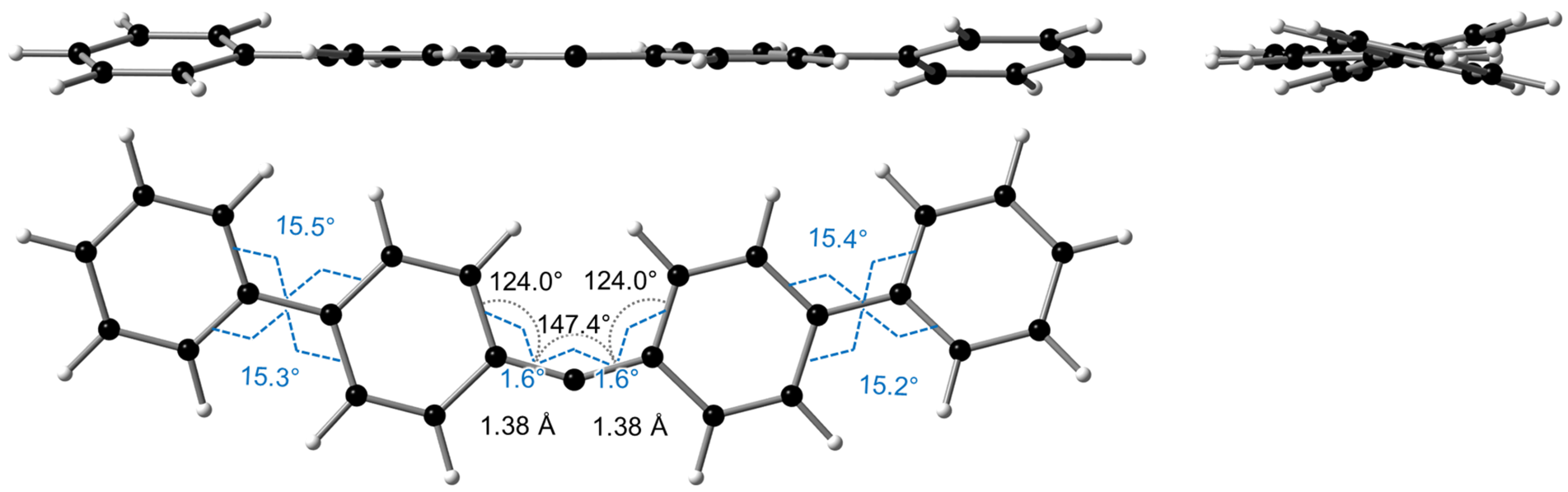

Supplementary Figure 23. Optimized geometry for the first excited triplet state in vacuum obtained via TD-DFT calculations at the B3LYP/def2-TZVPP level of theory (unrestricted Kohns-Sham calculations).

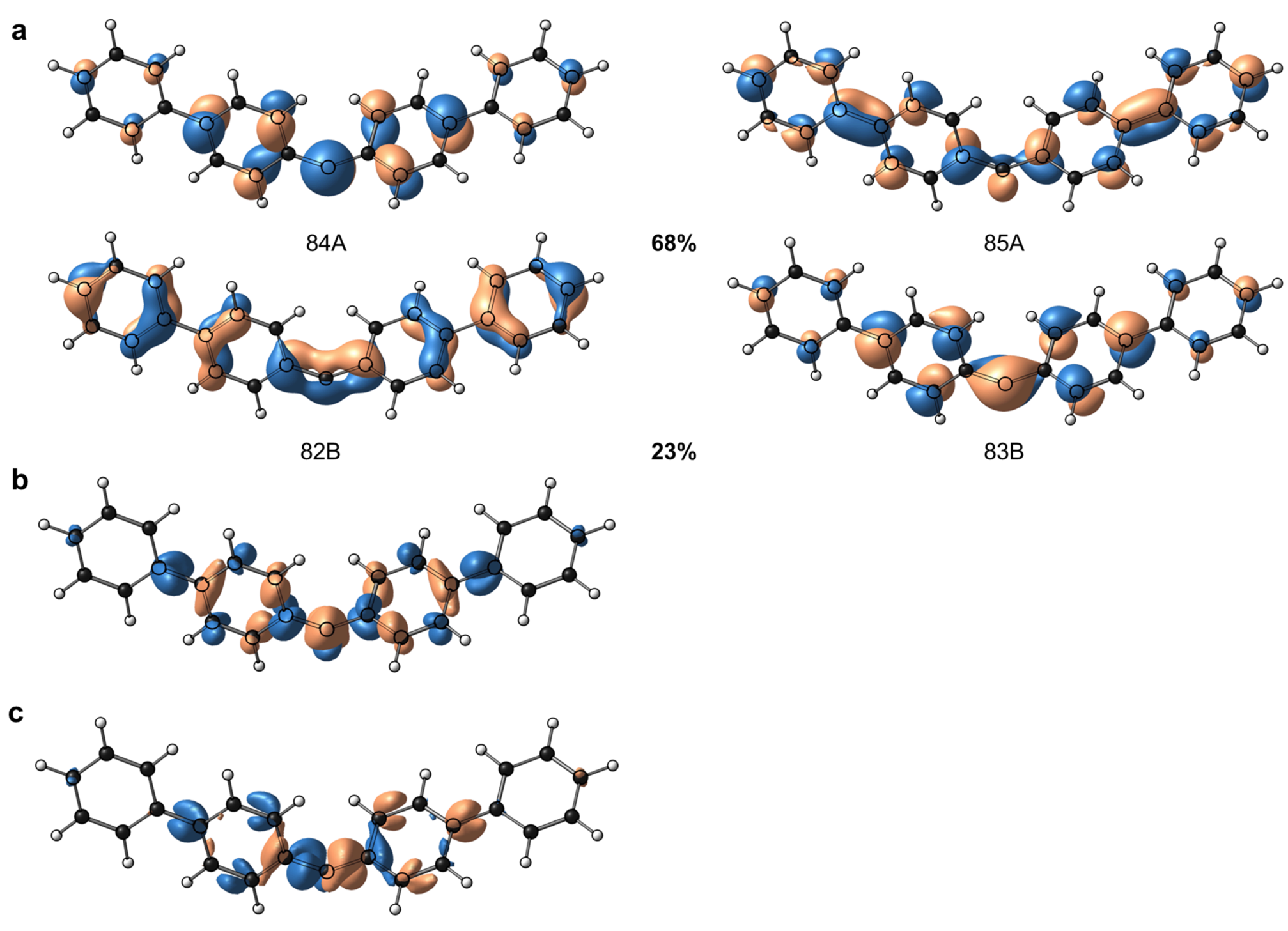

Supplementary Figure 24. TD-DFT calculations at the B3LYP/def2-TZVPP level of theory (unrestricted Kohn-Sham calculations); Representation of natural transition orbitals (NTO) of the "particle" (right) and "hole" pairs (left) that is dominated by SOMO→LUMO character (A-manifold, 68%) and to a smaller extent by HOMO→SOMO character (B-manifold, 23%, a); Difference density with depletion (orange) and accumulation (blue) zones showing a locally excited state (B3LYP/def2-TZVPP, isosurface=0.0015, b) and transition density plot showing regions that contribute to the transition dipole moment (TDM, isosurface=0.0015, b, compare to Fig. 1a in the manuscript).

E. Property prediction via TD-DFT

The TD-DFT calculation (B3LYP/def2-TZVPP, D4) of the carbene in its triplet ground state (pseudo-C_2 geometry from ONIOM calculations) predicts the lowest energy excitation ($T_0 \rightarrow T_1$ transition) at 2.55 eV (487 nm) with an oscillator strength of $f = 0.084$ (Supplementary Fig. 24). This lowest energy transition is multiconfigurational in character and is dominated by a SOMO→LUMO (A-manifold, 68%) and, to a smaller extent, by a HOMO→SOMO (B-manifold, 23%) transition. The difference density exhibits electron depletion and accumulation zone in which the contributions from the outer phenyl rings in the A- and B-manifold are mutually cancelled out and the density shift upon electronic excitation is predominantly localized on the central carbene carbon and the next-neighbor phenyl rings. This spatial confinement indicates that the excitation can be characterized as a locally excited (LE) state within the central core of the molecule, with no relevant charge-transfer contribution, which is in line with the spectroscopic observation of a sharp and pronounced ZPL and negligible solvatochromic shift of the emission band. Furthermore, the transition density plot, which represents the mathematical product of the ground state and excited state wavefunctions also shows that the overlap region predominantly contributing to the transition dipole moment is mainly localized of the central carbene and the adjacent phenyls.

F. Property prediction via CASSCF/NEVPT2

1. Active space deduction

The ONIOM-derived structure of di([1,1'-biphenyl]-4-yl)carbene exhibits a slight structural deviation from ideal C_2-symmetry compared to the fully C_2-symmetrized geometry, with a root-mean-square deviation (RMSD) of 0.009 Å (Supplementary Fig. 25). Noteworthy in this regard is the slightly more co-planarized biphenyl on the right hand side (avg. difference to the other biphenyl ca 1.1°). Further we note that the final geometry obtained in the ONIOM calculations is largely independent of the starting orientation of the cleaved-off nitrogen, yielding virtually identical structures if e.g. the nitrogen was cleaved off in an in-plane fashion. This leads us to the conclusion that the observed pseudo-C_2 symmetry is not an artefact from ONIOM calculations but results from the asymmetric relaxation of the expelled nitrogen within the crystal lattice (c.f. Supplementary Fig. 22). For property predictions we therefore use the C_2-symmetry obtained from ONIOM calculations.

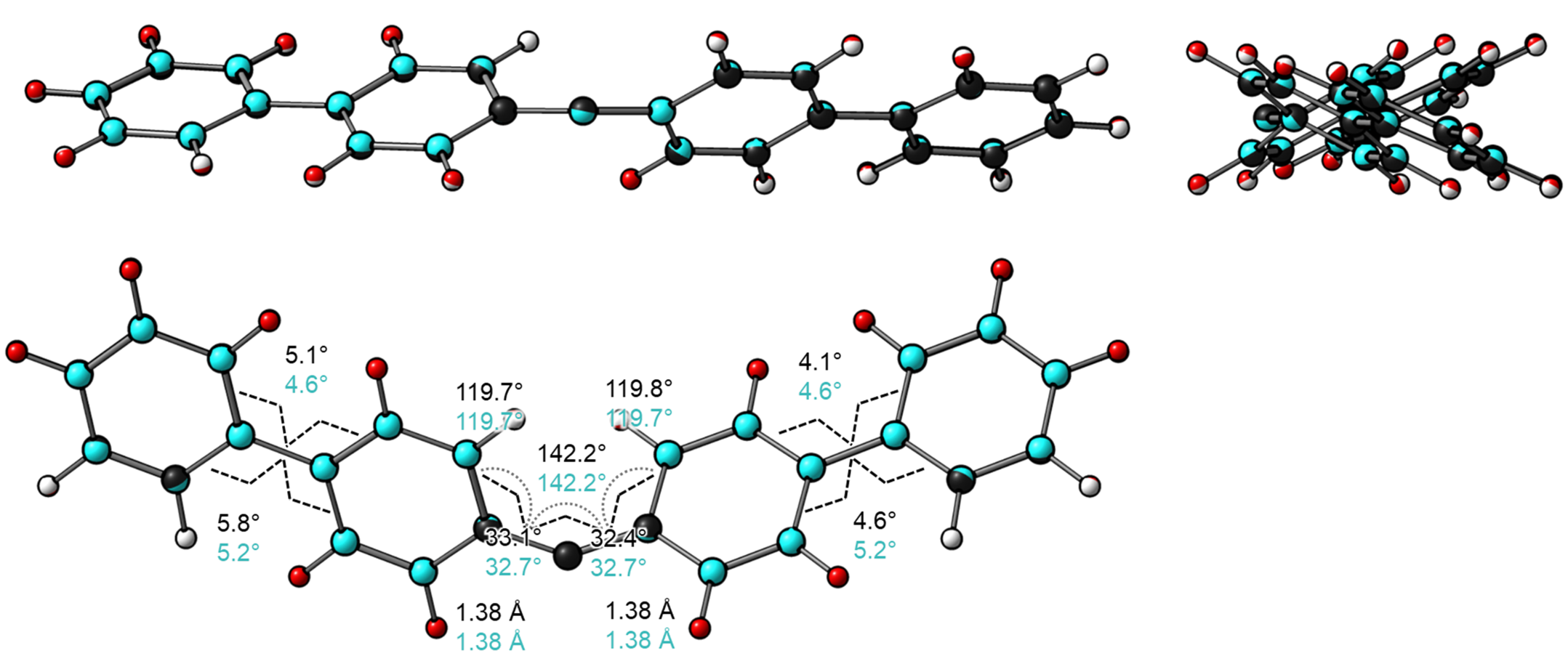

Supplementary Figure 25. Merged geometries of pseudo-C_2 symmetric di([1,1'-biphenyl]-4-yl)carbene isolated from ONIOM calculations (black carbons, white hydrogens) and the perfect C_2 symmetric geometry (turquoise carbons, red hydrogens; root-mean-square deviation (RMSD) of 0.009 *Å*).

2. Complete active space

The pairing theorem associated with the alternancy symmetry of our carbenes dictates that the active space must comprise $4n + 2$ electrons and an equal number of orbitals (where n is a non-negative integer) to prevent artificial

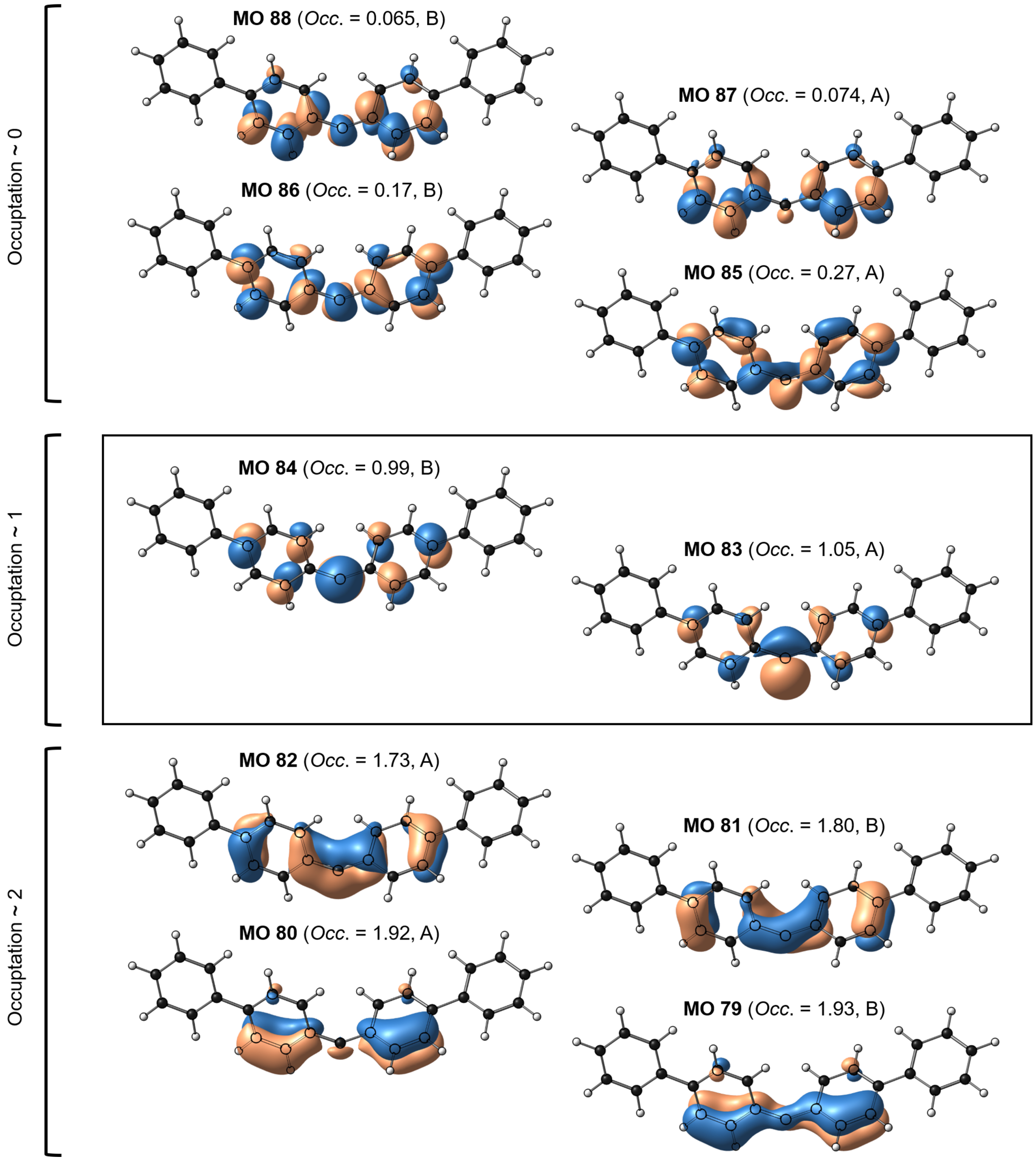

Supplementary Figure 26. Molecular orbitals of the qubit within the chosen (10,10) active space together with the energies and occupancies for the ground state triplet (isovalue= 0.04) and the tentativley assigned irreducible representations.

symmetry breaking.[20] Using the ONIOM pseudo-C_2 symmetric geometry of our qubit, a minimum active space of 10 electrons in 10 orbitals was necessary to arrive at the a sufficiently complete and balanced active space. Attempts to use a larger (14,14) active space were ultimately abandoned due to excessive computational demands required for strongly contracted N-electron valence state perturbation theory. When employing a (10,10) active space the occupation numbers at the outermost were 1.93 and 0.07 (Supplementary Table 4).

Exploratory SA8-CASSCF(10,10)/QD-SC-NEVPT2/def2-TZVPP calculations were also performed on the carbene+N_2 isolated from the ONIOM calculations to evaluate the influence of the neighboring nitrogen on the electronic structure of the carbene. While absolute numbers for the properties predicted below varied by up to $\pm10\%$ (mostly due to a minor overlap of the σ-orbital with the closeby nitrogen), qualitatively we find no changes in the overall picture presented and, hence, omitted the nitrogen from further calculations for the sake of clarity.

The active orbitals obtained from above mentioned SA8-CASSCF(10,10)/QD-SC-NEVPT2/def2-TZVPP calculations are plotted in Supplementary Fig. 26 using the Chemcraft software.[21] In total, six triplet and six singlet roots were computed, and a state-averaged CASSCF (SA-CASSCF) optimization was performed over the lowest three triplet and five singlet states (SA8), as these correspond to the optical range of interest (see Supplementary Table 4). Visual inspection of the active orbitals reveals that the CAS is predominantly delocalized over the central carbene carbon and the adjacent phenyl rings. Based on the pseudo-C_2 symmetry we tentatively assigned irreducible representation

A and B according to their symmetry/antisymmetry to both the identity operation E as well as the 180° rotation around the C_2 axis.

3. *Singlet triplet gap*

The vertical singlet-triplet energy gap (ΔE_{ST}) was estimated from the abovementioned SA8-CASSCF(10,10)/QD-SC-NEVPT2/def2-TZVPP calculations. In this framework the vertical singlet-triplet energy gap (ΔE_{ST}) is then provided by the Eigenvalue of state 3 with ~0.48 eV, which can best be described as an closed shell singlet state with minor open shell singlet admixture (see Supplementary Table 4). This value is slightly smaller than the previously reported ΔE_{ST} ~0.63 eV of a familiar carbene doped into a molecular matrix.[3]

4. *State structure*

**CASSCF triplet states**		
ROOT 0: E = -957.5312064575 Eh		
0.79021	[0]	222221100000
0.03214	[21]	222211110000
0.02226	[147]	222121101000
ROOT 1: E = -957.4225960089 Eh (2.955 eV, 23837.2 cm^{-1})		
0.31957	[1]	222221010000
0.25699	[20]	222211200000

**CASSCF singlet states**		
ROOT 0: E = -957.5048293774Eh		
0.57503	[0]	222222000000
0.18202	[6]	222220200000
ROOT 1: E = -957.4796538119 Eh (0.685 eV, 5525.4 cm^{-1})		
0.68414	[1]	222221100000
ROOT 2: E = -957.4297097250 Eh (2.044 eV, 16486.9 cm^{-1})		
0.34488	[6]	222220200000
0.22454	[0]	222222000000

Supplementary Table 4. CASSCF-calculated multiconfigurational electronic wavefunctions of ground (root 0) and first excited states (root 1,2,...) of the triplet and singlet manifold. Shown here are the energies and relative contributions of single configurations (weights and electron occupations of the CAS, using 10 orbitals). Except for root 0 of the triplet states, configurations that contribute with ≤ 10% are not shown but can be found in the Orca output file.

**State**	**Energy ($\mathbf{cm^{-1}}$)**	**Weight**	**Real**	**Imaginary**	**Block**	**Root**	**Spin**	M_s
0	0.0000	0.999876	-0.000004	-0.999938	0	0	1	0
1	0.3739	0.499995	0.707086	0.004922	0	0	1	1
		0.499992	0.707084	-0.004922	0	0	1	-1
2	0.4068	0.499945	-0.004858	0.707051	0	0	1	1
		0.000107	-0.000001	-0.010357	0	0	1	0
		0.499948	-0.004933	-0.707053	0	0	1	-1
3	3834.5438	0.999997	-0.999999	0.000001	1	0	0	0
4	6943.4878	1.000000	-1.000000	0.000000	1	1	0	0
5	14505.1925	0.999998	-0.999999	0.000008	1	2	0	0
6	23219.6353	0.499609	0.008439	-0.706780	0	1	1	1
		0.000773	-0.000001	-0.027804	0	1	1	0
		0.499618	0.008432	0.706786	0	1	1	-1
7	23219.6615	0.499985	-0.707046	-0.008469	0	1	1	1
		0.499976	-0.707043	0.008164	0	1	1	-1
8	23219.8841	0.000406	0.004159	0.019711	0	1	1	1
		0.999188	0.000001	-0.999594	0	1	1	0
		0.000406	0.004162	-0.019711	0	1	1	-1
9	24873.7791	0.130933	0.257444	-0.254274	0	2	1	1
		0.738196	0.000036	-0.859183	0	2	1	0
		0.130870	0.257327	0.254270	0	2	1	-1
10	24873.7797	0.443379	0.658577	0.098259	0	2	1	1
		0.113188	-0.000020	0.336434	0	2	1	0
		0.443433	0.658619	-0.098254	0	2	1	-1
11	24873.7908	0.425688	0.002037	0.652445	0	2	1	1
		0.148615	-0.000610	-0.385505	0	2	1	0
		0.425697	0.000214	-0.652454	0	2	1	-1
12	26049.4515	1.000000	1.000000	-0.000000	1	3	0	0
13	26241.2140	1.000000	-1.000000	-0.000003	1	4	0	0

Supplementary Table 5. Spinstates in T_0 geometry (block 0 = triplet states, block 1 = singlet states) included into state averaging, based on the multiconfigurational CASSCF-calculated wavefunctions (root 0, 1,...), employing QDPT with SOC and SSC contributions.

6. Spin densities

Changes in the spin distribution were analyzed based on state specific CASSCF(10,10)/QD-SC-NEVPT2/def2-TZVPP calculations for the T_0 and T_1 electronic configuration. The transition from the T_0 to the T_1 state is accompanied by a stark change in the magnitude and sign of the axial and the transversal ZFS component D and E, respectively.

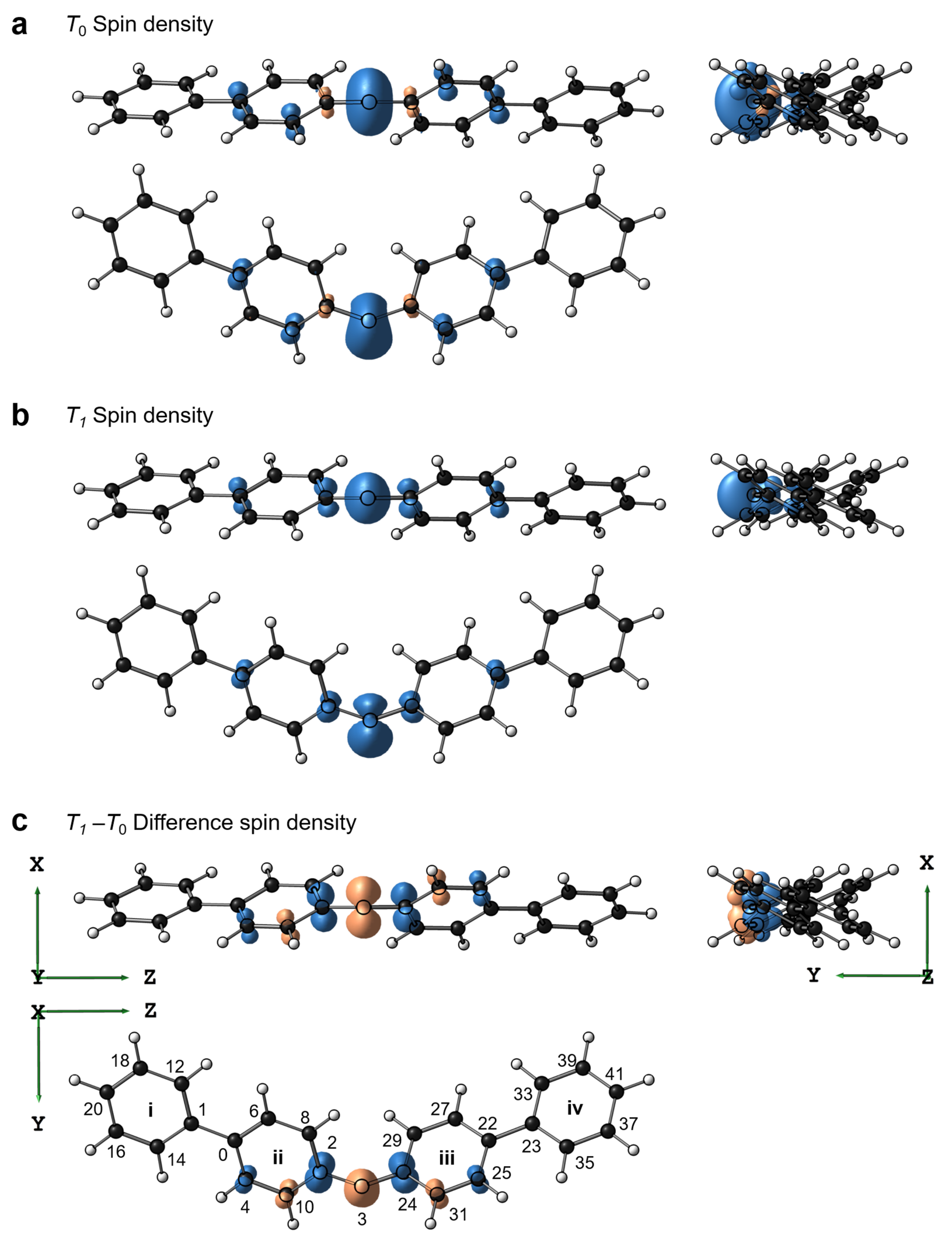

Supplementary Figure 27. Spin density plots from state specific CASSCF/NEVPT2 calculation (CAS(10,10)), in the (a) T_0 and (b) T_1 electronic configuration and (c) difference spin density obtained by mathematically subtracting T_1 from T_0 density together with atom numbering (orange denotes spin depletion and blue accumulation zones when going from T_0 to T_1, isosurface=0.015, see also Supplementary Table 6.

Supplementary Table 6. Loewdin spin densities and orbital breakdowns for selected carbon atoms in T_0 and T_1 electronic configuration (see Supplementary Fig. 27 for atom numbering).

**Atom**	**Spin density (T_0)**	**Spin density (T_1)**	**Orbital spin (s, p_x, p_y, p_z)**
C0	0.1283	0.1330	T_0: s=0.0000, p_x=0.0963, p_y=0.0339, p_z=0.0048<br>T_1: s=0.0000, p_x=0.0893, p_y=0.0314, p_z=0.0044
C1	0.0185	0.0209	–
C2	0.0811	0.2246	T_0: s=0.0035, p_x=-0.0608, p_y=-0.0145, p_z=0.0081<br>T_1: s=0.0035, p_x=0.0988, p_y=0.0431, p_z=0.0112
C3	1.0941	0.6931	T_0: s=0.0636, p_x=0.4657, p_y=0.5578, p_z=0.0001<br>T_1: s=0.0540, p_x=0.0746, p_y=0.5027, p_z=0.0002
C4	-0.0220	0.0783	T_0: s=0.0001, p_x=-0.0396, p_y=-0.0146, p_z=-0.0015<br>T_1: s=0.0001, p_x=0.0426, p_y=0.0160, p_z=0.0022
C6	0.0161	0.0429	–
C8	0.0697	0.0637	–
C10	0.1182	0.0376	T_0: s=0.0018, p_x=0.0856, p_y=0.0346, p_z=0.0109<br>T_1: s=0.0013, p_x=0.0040, p_y=0.0012, p_z=0.0050
C12	0.0082	0.0108	–
C14	0.0078	0.0106	–
C16	0.0022	0.0031	–
C18	0.0023	0.0031	–
C20	0.0062	0.0087	–
C22	0.1294	0.1326	T_0: s=0.0000, p_x=0.0984, p_y=0.0330, p_z=0.0048<br>T_1: s=0.0000, p_x=0.0902, p_y=0.0302, p_z=0.0043
C23	0.0187	0.0209	–
C24	0.0811	0.2230	T_0: s=0.0035, p_x=-0.0624, p_y=-0.0132, p_z=0.0081<br>T_1: s=0.0036, p_x=0.0993, p_y=0.0411, p_z=0.0110
C25	-0.0221	0.0782	T_0: s=0.0001, p_x=-0.0406, p_y=-0.0140, p_z=-0.0016<br>T_1: s=0.0001, p_x=0.0434, p_y=0.0152, p_z=0.0023
C27	0.0163	0.0425	–
C29	0.0701	0.0635	–
C31	0.1187	0.0378	T_0: s=0.0018, p_x=0.0883, p_y=0.0324, p_z=0.0110<br>T_1: s=0.0014, p_x=0.0043, p_y=0.0012, p_z=0.0050
C33	0.0083	0.0108	–
C35	0.0079	0.0106	–
C37	0.0023	0.0031	–
C39	0.0023	0.0032	–
C41	0.0063	0.0087	–

In the T_0 state, radical character is highly localized at the C3 center (ρ=1.09), dominated by the p_y (0.56, mainly σ-orbital) and p_x (0.47, mainly p-orbital) orbitals and a weak contribution from the s-orbital (0.06) due to hybridization (Supplementary Table 6, for spin density plots, atom numbering and coordinate system see Supplementary Fig. 27a). This spatial confinement is characteristic for bent carbenes and results in a strong dipolar spin-spin interaction and an oblate ZFS tensor with a large positive magnitude (D_{T_0} = 11702 MHz, E_{T_0}=-493 MHz, see Sec. IV F 7). Upon excitation to T_1, the central carbene carbon C3 donates significant spin density to the adjacent π-system; its total spin decreases to 0.69 as the localized p_x contribution drops to 0.07, while the p_y contribution remains substantial at 0.50 (Supplementary Fig. 27b). Notably, this transition drives a pronounced shift in the spin distribution. While the spin density on C3 (C31, C10) decreases substantially from 1.09 (0.12) to 0.69 (0.04) respectively, there is a corresponding accumulation of spin density on C24 and C2 (C25, C4), which increase from 0.08 (-0.02) to 0.22 (0.08) in T_1 (see Supplementary Fig. 27c). The resulting more diffuse spin distribution elongated along the z-axis in the T_1 state fundamentally alters the dipolar spin-spin interaction, thereby inverting the ZFS tensor to a prolate geometry

with a reduced, negative magnitude in the excited state (D_{T_1}=-7066 MHz, E_{T_1}=393 MHz).

7. *Zero-field splitting: Tensor parameters and spin state overlap*

The zero-field splitting (ZFS) parameters for the lowest-energy triplet state and the first excited triplet state were computed incorporating both spin-orbit coupling (SOC) and spin-spin coupling (SSC) contributions. The resulting ZFS tensors are then given by a coordinate frame (x,y,z) for the ground-state triplet and (x',y',z') for the excited-state triplet (see IV F 7) which are nearly identical (see Supplementary Table 8).

From Supplementary Table 5 we calculate the zero-field splitting (ZFS) parameters D and E (see Supplementary Table 7) with contribution from both spin-spin and spin-orbit interactions (here, the ground state T_0 is given by states 0, 1 and 2 while the first excited triplet state T_1 consists of states 6, 7 and 8) using the Hamiltonian of the form

$$H_{\mathrm{ZFS}} = D_x S_x^2 + D_y S_y^2 + D_z S_z^2 = D(S_z^2 - 1/3 \cdot S^2) + E(S_x^2 - S_y^2)$$

assuming a traceless tensor $\mathbf{D}$ and the convention[22] $|D_z| > |D_x| \geq |D_y|$, where the ZFS energies are $E(T_x) = 1/3 \cdot D - E$, $E(T_y) = 1/3 \cdot D + E$ and $E(T_z) = -2/3 \cdot D$.[23]

**ZFS Parameter**	**GS (MHz)**	**ES (MHz)**
D	11702	-7066
E	-493	393

Supplementary Table 7. ZFS parameters of di([1,1'-biphenyl]-4-yl)carbene in the triplet ground state (GS) and first excited state (ES) obtained from SA8-CASSCF(10,10)/QD-SC-NEVPT2/def2-TZVPP.

In order to estimate to which degree the optical transitions are spin-conserving between the ground and excited triplet states, we further calculate the overlap of the spin state as the vectorial product (see Supplementary Table 8). As a result, the spin state conservation is greater than 0.998 for each state between T_0 and T_1. For T_0 and T_2, however, we observe a reduced degree of spin state overlap, accompanied by an apparent rotation of the principal ZFS axis from the z-axis toward the x-axis (z overlaps mostly with x''). In the subsequent section, we evaluate the ISC pathways governed by direct spin-orbit coupling and demonstrate that T_2 likely participates as a mediating state. We note that the T_2 spin eigenfunctions should ideally be accounted for when explicitly computing the spin-orbit coupling matrix elements. However, as our current aim is to establish a qualitative picture, we assume that the spin state overlap remains sufficiently high across the relevant triplet manifold. This approximation allows us to keep the ground state triplet T_0 ZFS spin states x, y and z as basis for the spin selectivity.

T_0	T_1 **overlap**	T_2 **overlap**
x	$\|\langle x\|x'\rangle\|^2 > 0.998$	$\|\langle x\|z''\rangle\|^2 > 0.85$
y	$\|\langle y\|y'\rangle\|^2 > 0.999$	$\|\langle y\|y''\rangle\|^2 > 0.86$
z	$\|\langle z\|z'\rangle\|^2 > 0.999$	$\|\langle z\|x''\rangle\|^2 > 0.73$

Supplementary Table 8. Spin state overlap between ground state T_0 and excited states T_1 and T_2. The spin states are named x, y and z according to their energy (see above convention) with prime and doubleprime for the excited states to indicate that they do not necessarily need to be conserved.

8. *ISC pathways due to SOC*

In our previous work,[3] we analyzed the ISC pathways within the Condon approximation. By relying on the energy gap law, we restricted our analysis to the direct transitions between the photoexcited T_1 state and the closest-lying singlet state S_2, as well as the ground-state transition between T_0 and S_0. While this simplified approach yielded satisfactory agreement with experimental results at the time, the present theoretical study reveals two observations that necessitate a more nuanced treatment (see Supplementary Fig. 28). First, while a large, z-selective spin-orbit coupling matrix element (SOCME) exists exclusively for the ground-state ISC ($S_0 \leftrightarrow T_0$), the excited T_1 state exhibits comparatively weak direct SOCMEs to S_2 across all spin sublevels. Second, we identify a T_2 state in close energetic

proximity to T_1 that possesses a significantly larger SOCME with the S_2 state. Given our experimental findings of strong ISC and high spin-selectivity in the photoexcited state via the z-transition, these theoretical observations strongly point to a breakdown of the Condon approximation. The ISC rate is therefore likely dominated by a second-order spin-vibronic mechanism,[24] wherein the $T_1 \leftrightarrow S_2$ transition borrows intensity via non-adiabatic vibrational mixing between the closely spaced T_1 and T_2 states. While a fully quantitative evaluation of this pathway would require explicitly calculating the non-adiabatic coupling and the promoting vibrational modes, such an analysis exceeds the scope of the present theoretical study and remains a subject for future work.

The ISC selectivity of the ground state, on the other hand, is consistent with an insightful theoretical study on bent carbenes by Yuen-Zhou and co-workers,[25] in that we find that the pseudo-C_2 symmetry suppresses xy-selective ground-state ISC while opening the z-selective, El-Sayed-allowed channel by favoring population of the non-bonding σ orbital (see the state structure in Supplementary Table 4). From the SOCMEs in Supplementary Figure 28 we estimate normalized ground-state ISC rates $(0.000165, 0.000165, 0.999670)$ into states $T_{0\text{x}}$, $T_{0\text{y}}$ and $T_{0\text{z}}$, respectively.

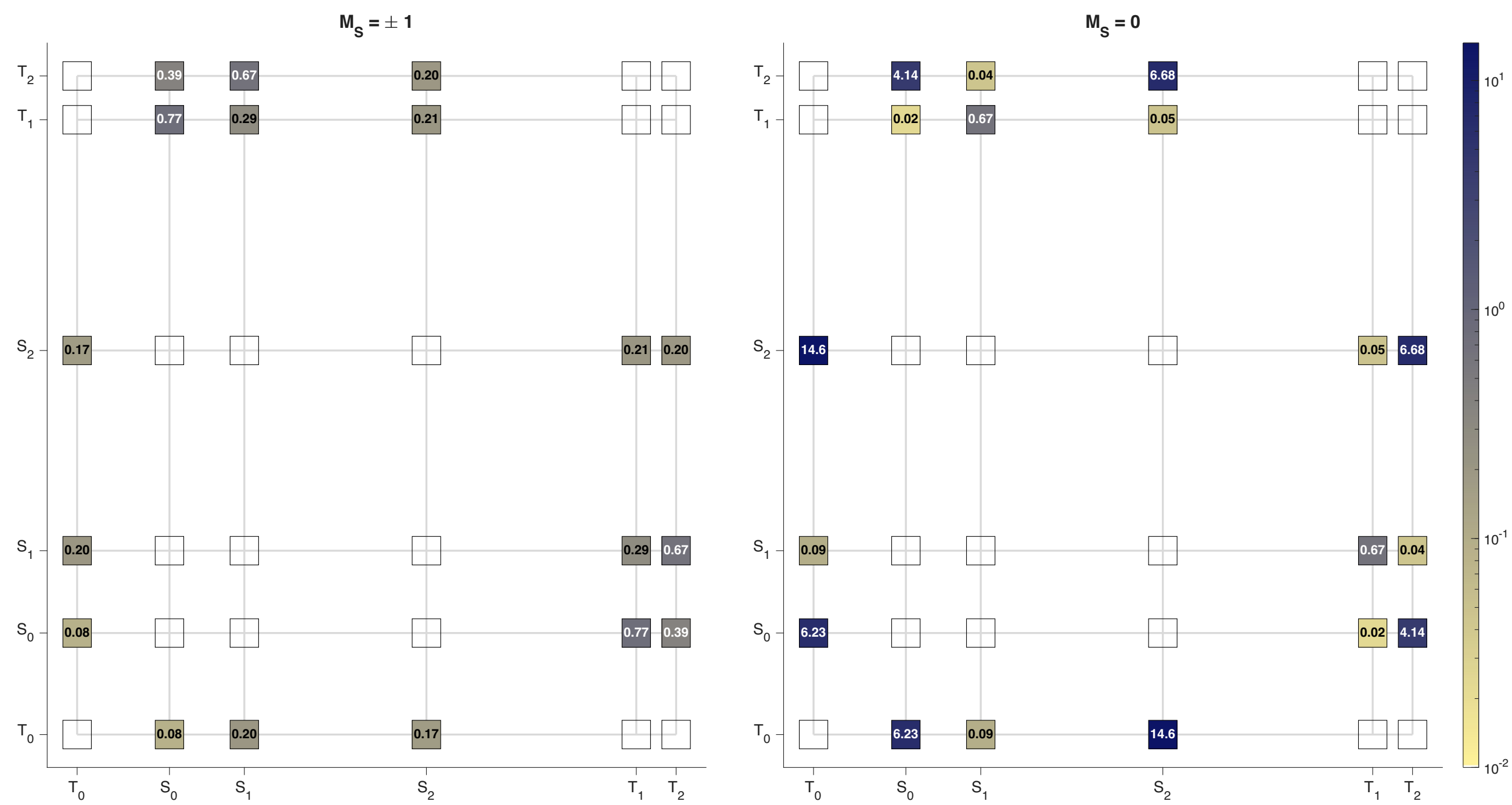

Supplementary Figure 28. Matrix of SOCMEs (in cm^{-1}) between the lowest three triplet and singlet states. The spin-dependent couplings are presented in the basis $M_S = \pm 1\,(0)$, which here correspond to a mixture of x/y (z) zero field triplet states. Values below 0.01 (in cm^{-1}) are omitted for clarity, and the spacing of the states along both axes is proportional to their relative energy differences. Notably, the direct coupling between the lowest photoexcited state T_1 and its closest-lying singlet S_2 is low across all spin sublevels. This indicates that strictly following the Condon approximation is insufficient for this transition, especially given the presence of the energetically close T_2 state, which exhibits strong, $M_S = 0$-selective ($= z$-selective) coupling to S_2 and likely acts as an intermediate perturbative state (see main text).

On the other hand, the experimentally z'-selective ISC observed in the excited state aligns with our experimental findings and warrants closer inspection. We further analyze this phenomenon by applying the theoretical framework established by Yuen-Zhou and co-workers.[25]

As has been discussed in a previous report,[26] in bent carbenes such as our pseudo-C_2-symmetric qubit, the departure from a linear D_2 geometry breaks the strict spatial symmetry that typically restricts excited-state ISC to the xy-plane ($M_S = \pm 1$). This structural bending unlocks a z-selective ($M_S = 0$) ISC channel mediated by B-irrep triplet states (see Supplementary Fig. 26 for irrep assignment). It is important to note that this channel can still be suppressed at specific bending angles due to destructive interference between HOMO-to-SOMO transition pathways. However, as previously argued, accurately modeling this system requires a precise evaluation of the T_1 and T_2 state mixing. As a comprehensive theoretical treatment of this mixing lies beyond the scope of the current work, we rely on our experimental findings to confirm the dominant z'-selectivity of the excited-state ISC.

9. The role of the photocleaved nitrogen

In the current view, in our system the nitrogen acts merely as a benign, sterically constrained neighbor rather than an active source of decoherence. To shine light on this aspect we consider long range and local effects in the following. As far a long range effects are concerned, we perform thermal annealing to 200 K following photoactivation, which actively relieves local matrix strain induced by the photocleavage of nitrogen and stabilizes the crystalline environment. This is monitored via in-situ EPR spectroscopy at around 120 K to 130 K, demonstrating that the structural relaxation reaches a stable equilibrium with constant observables, e.g. the resonance signal in the EPR and the emission wavelength of the ZPL in the cryo confocal measurements. Importantly, these observables do not shift over the wide range of doping ratios studied so far (0.5%-2.5 ppm), which suggests local matrix rearrangements rather than macroscopic crystal lattice restructuring to relieve strain. To the same effect, for the sub part-per-billion (ppb) levels intended for future quantum network applications, the average distance between individual qubit molecules is on the scale of hundreds of nanometers to micrometer. At these sparse concentrations, we do not expect macroscopic effects or correlated disorder arising from overlapping lattice strain fields between multiple carbene defect centers.
Regarding the local environment, our calculations indicate i) restructuring of the matrix upon nitrogen cleavage mostly along the y-direction (pointing along the C=O bond), and ii) that the nitrogen molecule remains trapped in the crystal lattice in close vicinity to the carbene center after photoelimination in accordance with previous experimental studies on congested carbenes.[17] While the precise molecular dynamics of this nearby nitrogen warrant further investigation, our current data does not allow for exclusion of a remaining detrimental effect on the qubit's properties.

V. MICROSCOPY AND REFRACTIVE INDEX TENSOR OF UNDOPED CRYSTALS

Thin-film crystals of the undoped matrix with hundreds of nanometer to few micrometer thickness were slowly grown with several hours by partially sealing a drop casted, several 100 nL of a 15 mM solution of di([1,1'-biphenyl]-4-yl)methanone (**S1**) in 1:1 tetrahydrofuran:mesitylene between a microscopy glass slide and a cover glass. Under these growth conditions, crystals typically exhibit top / bottom surfaces of parallelogramic outline (see Supplementary Figures 29 and 30) that have an acute angle of $(79.0 \pm 1)^{\circ}$ and coincide with the crystal cleavage plane. Crystals were selected by their homogeneous appearance exhibiting uniformity in thickness, and studied with a Zeiss Axio Imager microscope equipped with a conoscopy unit.

Using polarized light microscopy, the refractive index tensor axes of the biaxial crystal can be identified to be the normal to the plane of the parallelogram and the two bisectors, as depicted in (c) of Supplementary Fig. 29. The correct orientation of the refractive index axes in respect to the crystal outline can be identified by its conoscopy pattern (see Supplementary Fig. 30). Identifying the refractive index along the acute bisector with the immersion method [27] under polarized light to be ~ 1.55, one can thus deduce the full tensor by simulating the conoscopy pattern [28] [29]. In the example shown, the crystal thickness was measured to be 2.7 μm. A nominal maximum numerical aperture of 0.9 for both the condenser and objective was employed as a simulation input, allowing the crystal's refractive index tensor to be estimated as $(n_x \sim 1.55, n_y \sim 1.74, n_z \gtrsim 1.85)$.

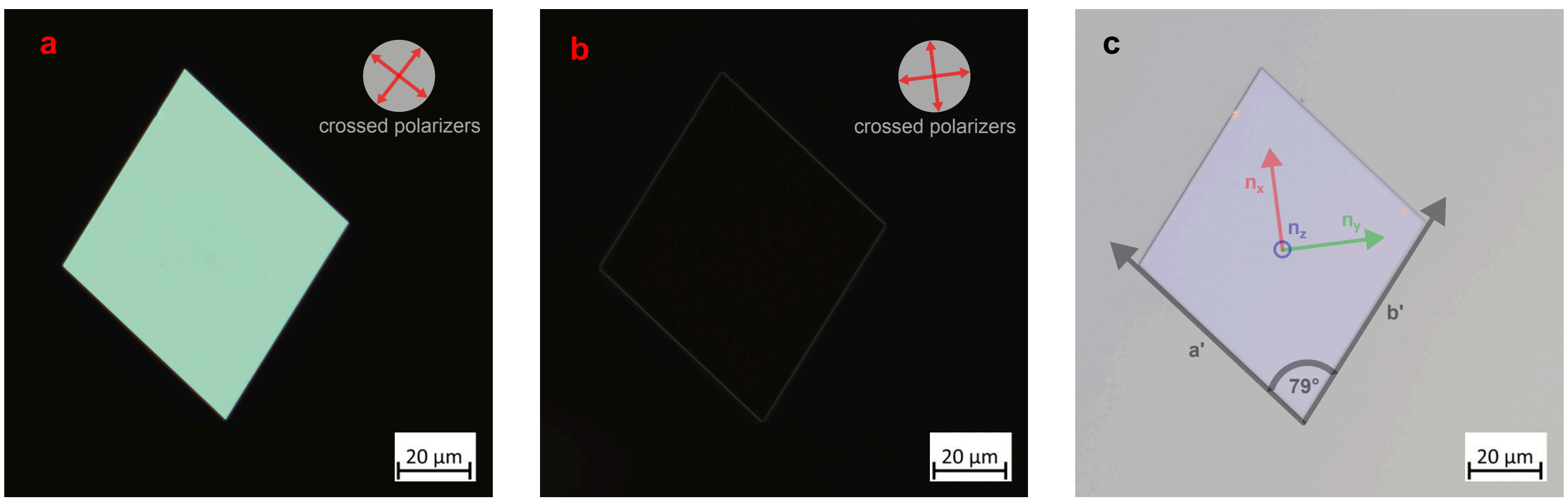

Supplementary Figure 29. Microscopy images of a submicron thick crystal. From left to right: Image under crossed linear polarizers (indicated by insets) with maximum brightness of crystal appearance (a). Polarizers turned by 45° in respect to bright appearance, matching the in-plane refractive index axes (b). As a result, the crystal is rendered completely dark except for scattered light on the edges. A brightfield image of the crystal (c) with refractive index axes (n_x, n_y, n_z) and axes (a', b') drawn onto the parallelogramic outline.

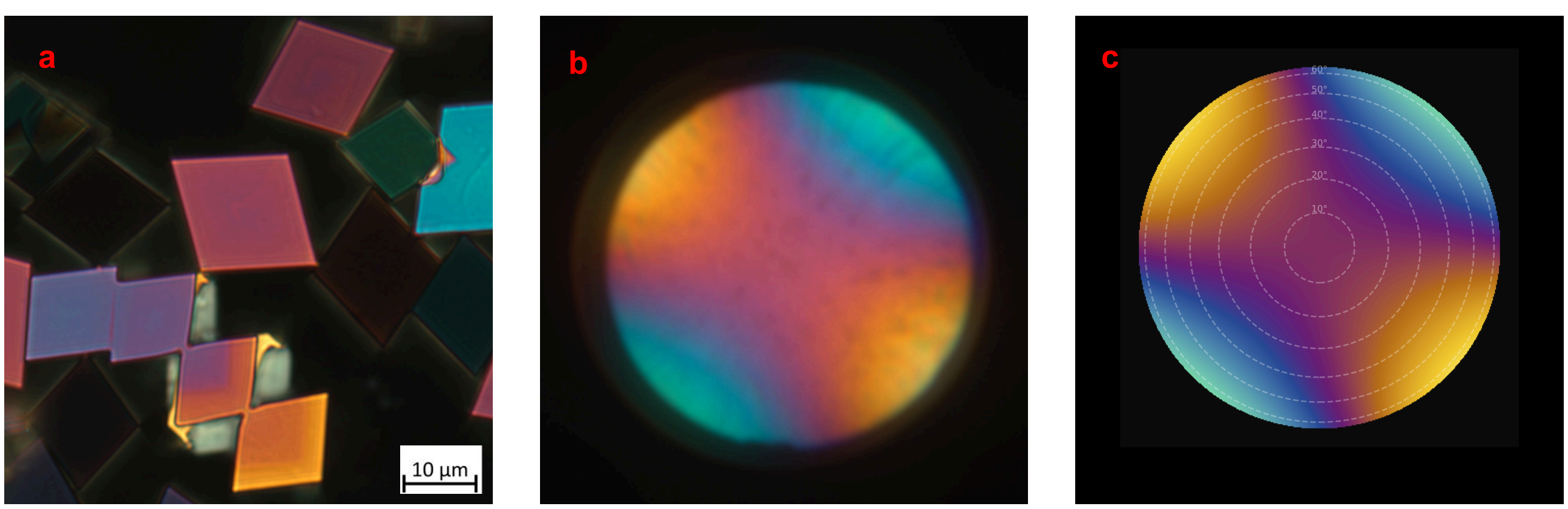

Supplementary Figure 30. To evaluate the refractive index tensor via conoscopy, the central 2.7 μm thick crystal in the polarized image of (a) was chosen. (b) Experimental conoscopy pattern and (c) corresponding simulation, with an overlaid polar grid indicating the inclination angle collected by the high NA of the microscope. Simulation parameters and results are detailed in the main text.

VI. EPR SPECTROSCOPY: ENSEMBLE GS ZFS TENSOR

The ground state zero-field splitting (GS ZFS) tensor of the carbene spin ensemble and its eigenframe compared to the crystal frame (and hence refractive index tensor frame, see Sec. V) were characterized using EPR spectroscopy on a Bruker Elexsys II X-band spectrometer, operated with a dielectric ring resonator (Bruker ER 4118X-MD5) and equipped with an Helium-flow cryostat (Oxford Instruments CF935) to allow temperature-dependent measurements and in-situ annealing. A small, selected crystal of di([1,1'-biphenyl]-4-yl)carbene doped into di([1,1'-biphenyl]-4-yl)methanone (0.25 mol %) with flawless optical appearance and $\sim 0.1\,\mathrm{mm}^3$ volume (2500 ppm doping ratio) is inserted into a quartz glass tube and fixed with a PVA coating. Crystal samples are rotated by a goniometer along the axis of the sample stick, perpendicular to B_0. For photoactivation of the carbene ensemble we illuminate the sample with a 395 nm UV LED lamp (Jupitertech NSP1, maximum intensity of $12\,\mathrm{W/cm}^2$) held in front of the cryostat optical side-port window at cryogenic temperature. The photoactivation was followed by reading the EPR signal amplitude and was found to be completed after a duration of typically few ten seconds of illumination.

A. Rotation-dispersion spectra: ground state (GS) ZFS frame

After photoactivation at temperatures of $\sim 50\,\mathrm{K}$, the continuous-wave field-swept EPR spectrum typically contains two close-lying resonances of the same order of magnitude, which can be identified by a few degree tilt between otherwise magnetically identical sites of spin 1. To achieve a complete characterization of the zero-field splitting (ZFS) tensor, rotation-dispersion spectra were recorded for two orthogonally oriented crystals. In the first configuration, the rotation axis was aligned with the normal to the cleavage plane; in the second, it coincided with the crystallographic a'-axis (see Supplementary Fig. 29c). In both cases, the spectra are recorded for the nascent and annealed state of the carbene after photoactivation (see Supplementary Fig. 33 for illustration of the effect of annealing on the signals). We used the following experimental and spectrometer settings: Recordings of nascent and annealed spectra were all done at 50 K, annealing was performed between 120 and 130 K, further, the carbene spins where excited with $15\,\mu\mathrm{W}$ microwave power before the cavity with a quality factor Q $\sim$ 10'000, magnetic field sweeps were done with 20 ms conversion time and 200 mT (800 mT) sweep width with 4000 (8000) data points and 2.5 G (5 G) amplitude modulation at 100 kHz for Supplementary Fig. 31 (Supplementary Fig. 32).

The spectral positions of the rotation-dispersion spectra are then simulated with EasySpin.[30] For the nascent case, we find a good agreement using $(D, E)_{\mathrm{nascent}}$ = (10900, -520) MHz. For the annealed case we use the values of $(D, E)_{\mathrm{annealed}}$ = (11160, -541) MHz found from the cryo-confocal measurements. Further, we introduce a tilt of 2.5° between the paramagnetic sites along the D_y axis. In both cases, we assume an isotropic g-factor of 2.0023. It is important to note the lack of significant deviation in ZFS values between the nascent and annealed states. This may

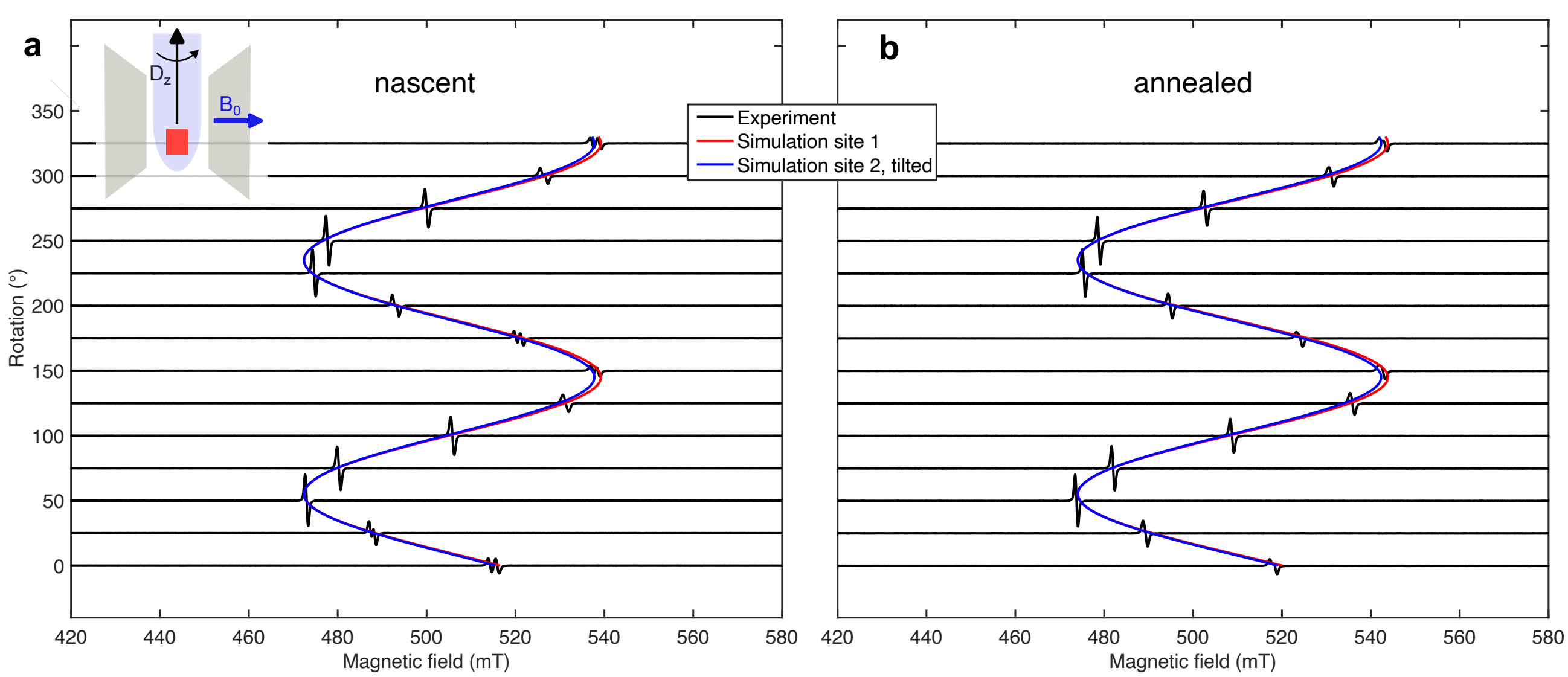

Supplementary Figure 31. Rotation-dispersion spectra with simulation of the resonance line positions, of a nascent (a) and annealed (b) crystal with its cleavage plane normal (corresponding to the ZFS D_z axis by analysis) orientated along the rotation axis as depicted in the inset of (a).

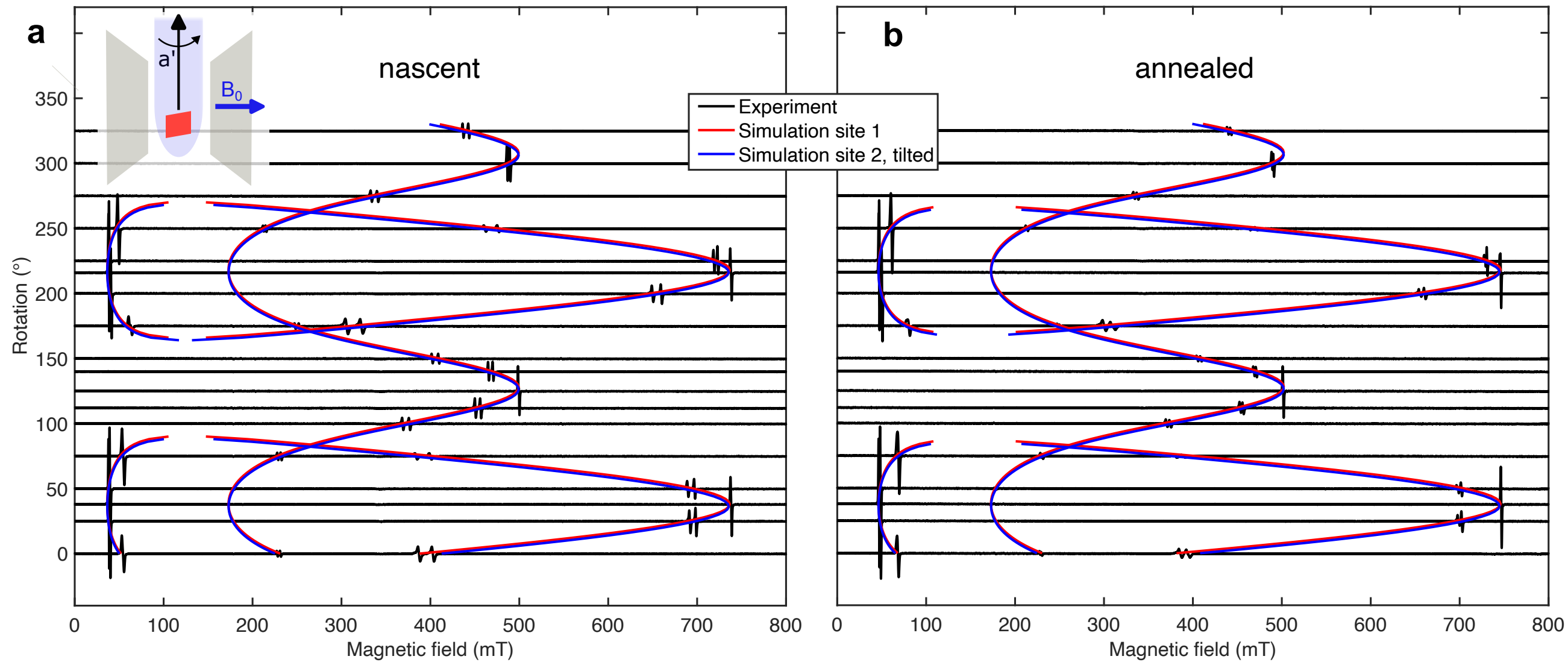

Supplementary Figure 32. Rotation-dispersion spectra with simulation of the resonance line positions, of a nascent (a) and annealed (b) crystal with its crystal a' axis (refer to Supplementary Fig. 29 c) orientated along the rotation axis as depicted in the inset of (a).

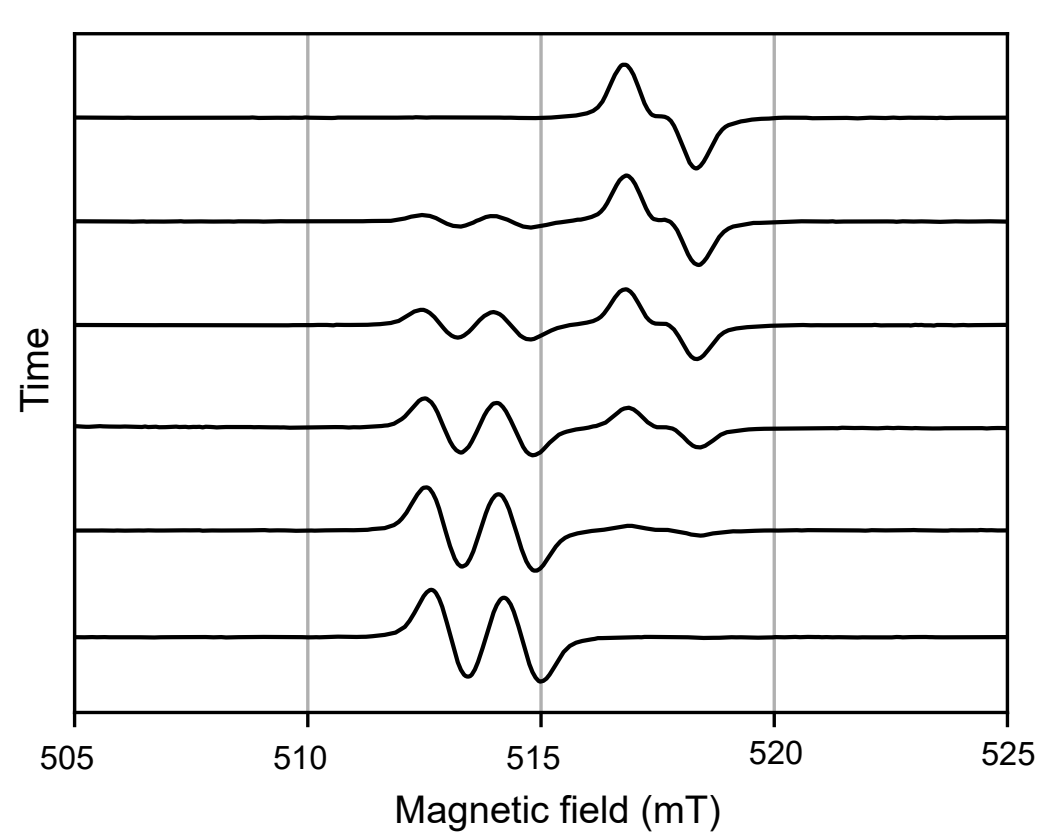

Supplementary Figure 33. Annealing at $\sim 120\,\mathrm{K}$ of the crystal of Supplementary Fig. 31 using the rotation position of the goniometer at 0°, and recording the field-swept EPR spectrum about every 10 minutes.

indicate that the crystalline matrix is rigid to an extent that it precludes significant structural changes of the carbenes.

B. Comparison: Crystal, ground state ZFS and refractive index frame

Combining the polarization microscopy results on undoped thin-film crystals and the measurement of the ground state ZFS tensor with EPR on doped bulk samples (0.25 mol %), we arrive at the conclusion that ZFS and refractive index tensor axes are co-aligned (up to a few degree of alignment, measurement and simulation uncertainty) and can be referred to characteristic crystal axes (see Supplementary Table 9). This result matches very well the predicted orientations from the X-ray structure together with theoretically derived ZFS tensor axes (see Supplementary Fig. 34).

C. Thermal Stability

When doped into the matrix and photoactivated the carbenes are thermally stable at least up to 250 K with no signal loss within the measurement error over several hours. At around 300 K the carbenes become unstable with slow signal decrease over hours.

Crystal frame	GS ZFS Frame	Refractive index frame
Acute bisector of CP	D_x	n_x
Obtuse bisector of CP	D_y	n_y
Normal to CP	D_z	n_z

Supplementary Table 9. Orientation of the ground state ZFS frame in respect to the crystal frame and refractive index frame (see Supplementary Fig. 29). CP: cleavage plane.

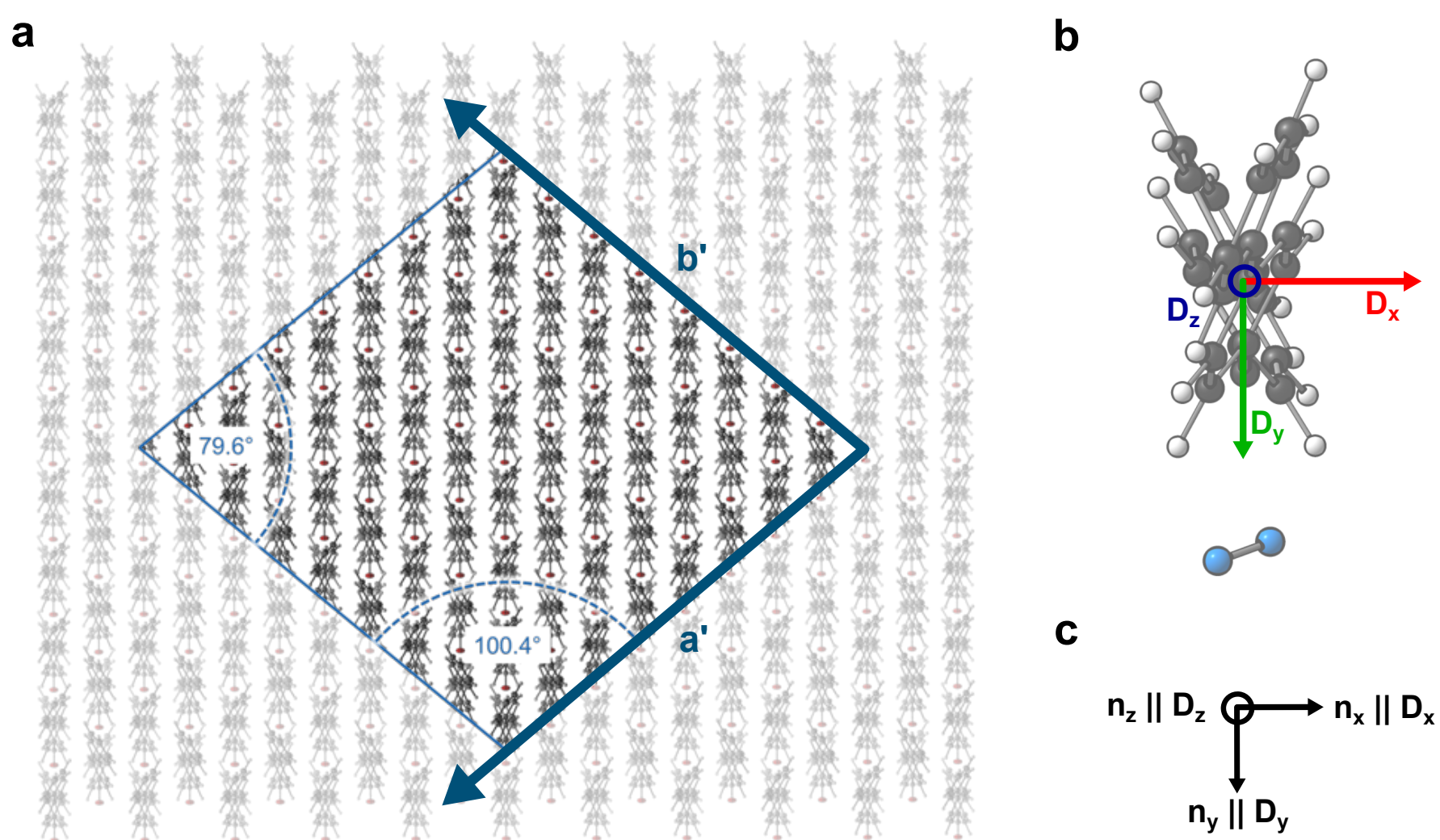

Supplementary Figure 34. Crystal image from X-ray structure data of the matrix (see section II B, a), view is normal to the cleavage plane, highlighting a monoclinic cell which matches well to the parallelogramic outline of grown crystals (see Supplementary Fig. 29, therein the axes definition of a' and b' which are repeated here). The predicted ZFS axes from quantum chemistry calculations of the carbene (see section Computational chemistry), adapted to the crystal frame by assuming that a doped carbene molecule substitutionally replaces a ketone molecule of the matrix, fits well to the ZFS axes referenced to the crystal frame from EPR measurements (b). All refractive index and ZFS axes are summarized in (c), compare to Supplementary Table 9.

VII. CRYO-CONFOCAL MEASUREMENTS

A. Experimental setup and estimated collection efficiency

The experimental setup is shown in Supplementary Fig. 35.

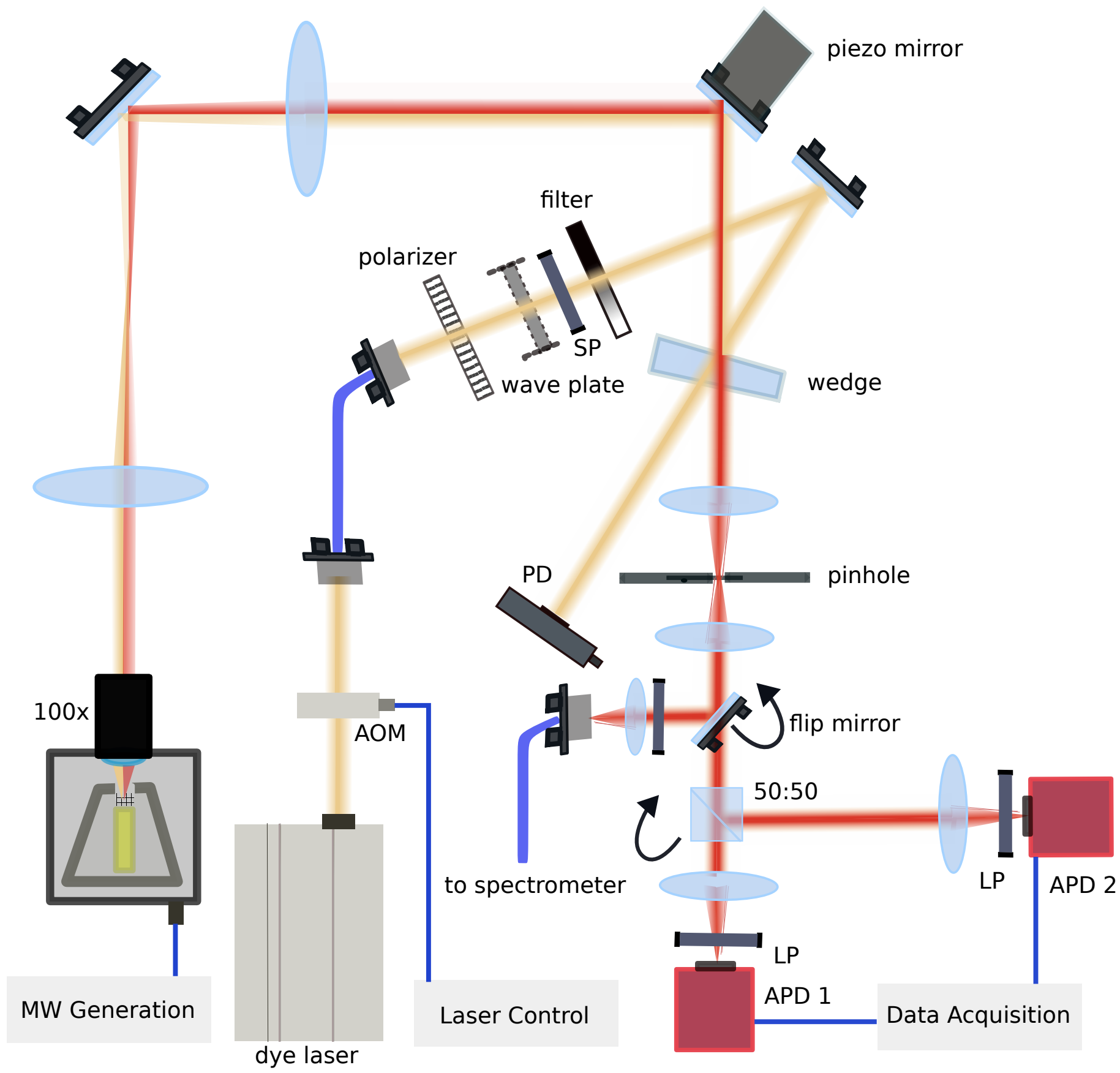

Supplementary Figure 35. Schematic drawing of the experimental confocal setup.

1. *Excitation path*

The excitation light is generated by a tunable dye laser, the wavelength of which is actively locked to a temperature-stabilized reference cavity and monitored by a wavemeter to ensure high frequency stability. The beam intensity is modulated, and pulses are defined using an acousto-optic modulator (AOM), which is driven by a dedicated laser control system. After spatial filtering via a single-mode optical fiber, an electro-optic intensity modulator (EOM) can be additionally integrated into the setup to enable ultra-fast laser switching for excited-state lifetime measurements. The polarization state of the excitation beam is adjusted using a linear polarizer and a subsequent half wave plate. The beam is spectrally cleaned using a shortpass (SP600) filter. The overall laser power can be adjusted using a neutral density (ND) filter wheel. A glass wedge transmits the largest fraction of the excitation beam onto a photodiode (PD) for power monitoring. The reflection of the excitation beam from the glass wedge is directed via a piezo mirror, serving as a fast steering mirror for lateral scanning. A 4f optical system relays the beam from the piezo mirror to the back aperture of the objective (NA= 0.9), which then focuses the light onto the sample.

2. *Sample Environment*

The sample is housed inside a cryostat to maintain cryogenic temperatures during the measurements. To ensure optimal thermal anchoring, the sample was mounted in direct contact with the cold finger using vacuum grease. For experiments involving spin manipulation, microwave magnetic fields were generated by a AC current-carrying wire (approximately 25 µm in diameter) positioned in close proximity to the sample. This wire was soldered onto a printed circuit board (PCB) that surrounds the cold finger and is connected to the external microwave (MW) generation module.

3. Collection Path and Detection

Single-molecule fluorescence is collected in an epi-illumination geometry through the same objective. The red-shifted emission is de-scanned by the piezo mirror. To ensure confocal detection and to reject out-of-focus background, the collected light is focused through a pinhole (25 µm). Under these optical conditions, the diffraction-limited Airy disk diameter at the pinhole plane is calculated to be approximately 22.55 μm. This configuration yields a transmission efficiency of 84.0%.
Following the pinhole, the signal can be routed into two distinct detection paths:
Spectroscopy Mode: A flip mirror can be introduced into the beam path to direct the fluorescence into a multimode fiber connected to a spectrometer (LP590).
Photon Correlation Mode: With the flip mirror removed, the beam enters a Hanbury Brown and Twiss (HBT) configuration. A 50:50 beamsplitter divides the signal into two detection arms. Each arm is equipped with a longpass (LP) filter to fully suppress any residual laser scattering. The transmitted photons are detected by two independent single-photon avalanche diodes, APD 1 (LP605) and APD 2 (LP615). Furthermore, to eliminate optical crosstalk caused by infrared breakdown flashes originating from APD 1, an additional 700 nm shortpass filter (SP700) is placed in the detection path of APD 2. The photon arrival times are recorded and processed by a data acquisition system.

4. Estimated Collection Efficiency

Geometric Collection Efficiency: We consider an oscillating electric dipole emitter embedded within a crystal matrix with a refractive index of $n_\mathrm{c} = 1.85$. The dipole is oriented horizontally (in-plane) and located at a depth close to the crystal surface ($\approx 1\,\mu$m). Emitted light is collected by a microscope objective positioned in vacuum ($n_\mathrm{vac} = 1.0$) with a numerical aperture of NA = 0.9. Here, we evaluate the ideal geometric collection efficiency η assuming infinity-space imaging prior to any confocal pinhole filtering. The maximum half-angle of collection in vacuum, θ_vac, is given by the definition of the numerical aperture:

$$\mathrm{NA} = n_\mathrm{vac} \sin\theta_\mathrm{vac} = 0.9 \tag{1}$$

According to Snell's law, the refracted rays originating from the crystal obey the boundary condition:

$$n_\mathrm{c} \sin\theta_\mathrm{max} = n_\mathrm{vac} \sin\theta_\mathrm{vac} = \mathrm{NA} \tag{2}$$

where θ_max represents the maximum acceptance half-angle inside the organic crystal. Substituting the given values yields:

$$\sin\theta_\mathrm{max} = \frac{\mathrm{NA}}{n_\mathrm{c}} = \frac{0.9}{1.85} = 0.5 \implies \theta_\mathrm{max} = 29^\circ \tag{3}$$

An oscillating electric dipole aligned along the x-axis does not emit isotropically. Furthermore, depending on the direction of emission the linear polarization has a different decomposition into s- and p-polarization with respect to the plane of incidence when hitting the crystal-vacuum interface. Taken all this into account when employing the Fresnel equations for transmission at interfaces, we integrate the total transmission of light emitted into cone θ_max and further into vacuum to the geometric collection efficiency

$$\eta_\mathrm{geo} \approx 7.9\% \tag{4}$$

of the total amount of emitted light.
Optical Losses: To quantitatively evaluate the expected photon count rates from the embedded dipole, we compiled an optical loss budget of the entire confocal microscope setup. The overall system detection efficiency η_sys is the product of the geometric collection efficiency η_geo, the cumulative transmission of all optical elements in the beam path T_opt and the photon detection efficiency η_APD of the avalanche photo detector. For example, for the long-pass filter (LP605), the effective transmission is derived from the product of its base transmission (97%) and the specific spectral overlap/cut-off ratio for our emission (40%), yielding an effective transmission of 39%. Supplementary Table 10 details the step-by-step transmission efficiencies of all components in the detection path.

Multiplying the transmission and reflection coefficients of all components yields an instrumentation and detection efficiency of approximately $T_\mathrm{opt} \approx 25\,\%$. Finally, the end-to-end probability of detecting an emitted photon is calculated to be $\eta_\mathrm{sys} = \eta_\mathrm{geo} \cdot T_\mathrm{opt} \cdot \eta_\mathrm{APD} \approx 1.3\,\%$.

Supplementary Table 10. Optical Loss Budget. The cumulative efficiency reflects the probability of a radiated photon surviving up to and including the respective component.

Component / Interface	Component Efficiency (%)	Cumulative Efficiency (%)
Dipole Geometric Collection	7.9	7.9
Optical Cryostat Window	99	7.8
Objective	88	6.9
Lenses	96	6.6
Mirrors	96	6.4
Beam Sampler	95.5	6.1
Pinhole	84	5.1
Long-Pass Filter (LP605)	39	2.0
η_{APD}	65	**1.3**

B. Activation mechanism and yield

The vast majority of previous photochemical studies on generation of related diphenylcarbenes have utilized light sources that include UV or blue light.[31–38] Similarly, for our diazomethane precursor we find that activation with UV or blue light to be much more efficient than with 580 nm used herein. The mechanism governing this wavelength-dependent efficiency has only recently been elucidated.[18] In this study the authors could successfully generate diphenylcarbene at 500 nm, but the quantum yields for the conversion were exceptionally low ($\Phi = 0.020$) compared to UV irradiation at 300 nm ($\Phi = 0.36$ to 0.46 depending on the solvent used). According to this and a follow-up study,[39] excitation to higher-lying singlet states (S_n, such as S_2 via UV/blue light) drives the system toward a non-planar S_1/S_0 conical intersection where outward forces effectively separate the carbene and N_2 fragments, resulting in highly efficient photoelimination. Conversely, excitation to the S_1 state (via lower-energy visible light, e.g., > 500 nm) leads to an in-plane deactivation pathway where the molecular forces actually hinder fragment separation. As a result, direct S_1 excitation is 10 to 50 times less efficient than higher S_n excitation.

Based on this mechanistic picture, we hypothesize that the previous usage of UV and blue light was widespread because the activation from higher excited states is so highly efficient. Considering that the direct reaction via the S_1 state is substantially slower, the possibility of using longer wavelengths (like 580 nm in our case, see Supplementary Fig. 17a for the absorption spectrum of precursor **S3**) was mostly overlooked in the literature. However, for our single-molecule microscopy experiments utilizing 580 nm is advantageous in that the longer wavelength allows us to more selectively address the target molecules, minimize background autofluorescence, and avoid photodamage to the host matrix and the qubits themselves. The reduced photoelimination efficiency is thereby easily compensated by a higher photon flux in our confocal laser setup.

A coarse estimate of the conversion yield from precursors to activated and annealed carbenes in the cryo-confocal is performed as follows:

- We measure the amount of fluorescence from a fully activated spot and compare it to the amount of fluorescence from a single carbene molecule to estimate the total amount of detected carbenes N_{carbene}.
- We measure the extent of the confocal PSF and calculate the amount of expected precursors $N_{\mathrm{precursor}}$ in that volume.
- We estimate the creation yield to be

$$\eta = N_{\mathrm{carbene}}/N_{\mathrm{precursor}} \tag{5}$$

The fluorescence microscopy image in Figure 2a shows one fully activated spot that exhibits a fluorescence signal of 600 kcounts/s compared to 2 kcounts/s for a single carbene for simlar excitation. That is 4.5 nW resonant laser excitation on the maximum of the inhomogeneously broadened ZPL for the ensemble (FWHM ~ 3 GHz) and resonant excitation on the homogeneously broadened ZPL of a single carbene (FWHM ~ 40 MHz). In addition a microwave field resonant to the $T_{0\mathrm{z}} \leftrightarrow T_{0\mathrm{x}}$ transition is applied. Then we integrate the total excitation spectrum of Figure 2 (labeled XX') and estimate around 26 000 detected carbene molecules inside the confocal spot for site 1. We assume that additionally half of that amount is in site 2 (see Figure 2a) and therefore we end up with a total number of

detected carbenes of $N_{\text{carbene}} \approx 40\,000$.

The confocal volume of the microscopy setup is roughly $V = 1.44 \cdot \pi \cdot (0.3\,\mu\text{m})^2 \cdot 1.2\,\mu\text{m}$, where $0.3\,\mu\text{m}$ is the half width at half maximum of the lateral intensity distribution (PSF^2) of a single carbene, $1.2\,\mu\text{m}$ is the axial full width at half maximum and 1.44 is a conversion factor to have $\int \text{PSF}^2 dV = V$. The molecular density of matrix molecules is $\rho = 4/1.67\,\text{nm}^3$ and the nominal doping rate is $C = 25\,\text{ppm}$. We therefore estimate the amount of precursors inside a confocal volume to $N_{\text{precursor}} \approx \rho \cdot V \cdot C \approx 29\,000$. Finally, we arrive at an estimate for creation yield $\eta \sim 1.4(6)$, which is above the physical limit of $\eta = 1$. This estimate is intended only as an order-of-magnitude consistency check. The given uncertainties arise from the effective confocal volume, excitation and detection efficiencies, the amount of emitters in site 2 and the comparison between ensemble and single-molecule fluorescence. We have not incorporated any uncertainty for the local dopant concentration. Consequently, the estimate is not suitable for determining an absolute conversion yield. The estimate nevertheless suggests that the conversion efficiency is likely high. Activation of carbenes is also possible for wavelength at the ZPL of annealed carbenes. However, this creation cannot be directly monitored, because the laser light cannot excite nascent carbenes. To reveal their creation nonetheless, we have detected the creation afterwards by scanning the region where activation was performed with 580 nm laser light.

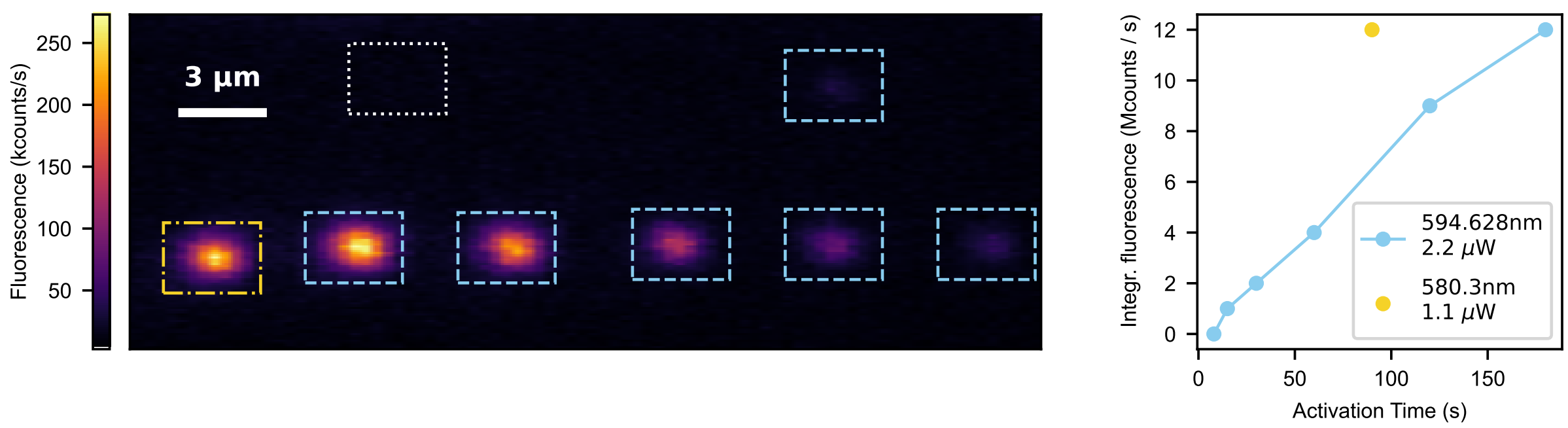

Supplementary Figure 36. Left, Confocal image recorded with 580 nm laser light. In regions marked with cyan dashed boxes activation has been performed with $2.2\,\mu$W of 594.628 nm laser light for different durations. In the orange dash-dotted box activation has been performed with $1.1\,\mu$W of 580.3 nm laser light for about 100 s. The white dotted box marks a region without activation (i.e. background level). Right, the fluorescence in each box is accumulated, background-corrected and displayed vs. the activation duration. Prolonged illumination leads to an increased amount of activated carbene molecules. The activation with 580 nm is significantly more efficient than activation with 594.628 nm laser light.

Supplementary Figure 36 shows the latter confocal image exhibiting multiple fluorescent spots where activation was performed for several durations. The fluorescence of the individual spots is integrated and displayed vs. the activation time. For comparison, activation is also shown for 580 nm laser light, where similar activation is achieved for half the laser power and half the activation time. The maximum degree of activation displayed in Supplementary Figure 36 is about half of the value for saturated activation. In[3] hints for a one-photon activation process were presented. Under these assumptions, continuous resonant illumination with about 2 nW of resonant laser illumination (as in the presented experiments of the main text) would lead to the activation of half the precursors in about 200 000 s (i.e. 2 days).

C. Comparison of annealed and nascent carbenes

When photo-activated at temperatures below 120 K, di([1,1'-biphenyl]-4-yl)carbene embedded in di([1,1'-biphenyl]-4-yl)methanone appears spectrally blue shifted as compared to the annealed qubit molecules investigated in-depth in the main text (heated up to 200 K for about one hour, see Supplementary Fig. 37). In addition, the nascent carbenes incorporate a much more dominant phonon side-band. Similarly, laser excitation spectra of nascent carbenes clearly show an increased inhomogeneous broadening in which, however, the fine structure is still visible (Supplementary Fig. 38). By taking into account the transition frequencies in Supplementary Fig. 39, the ground-state ZFS parameters for the nascent molecules of both molecular site 1 and 2 were deduced to be $|D_{T_0,\text{site 1}}| = 10.9300(2)$ GHz, $|E_{T_0,\text{site 1}}| = 516.0(2)$ MHz, $|D_{T_0,\text{site 2}}| = 10.9380(2)$ GHz and $|E_{T_0,\text{site 2}}| = 519.0(2)$ MHz.

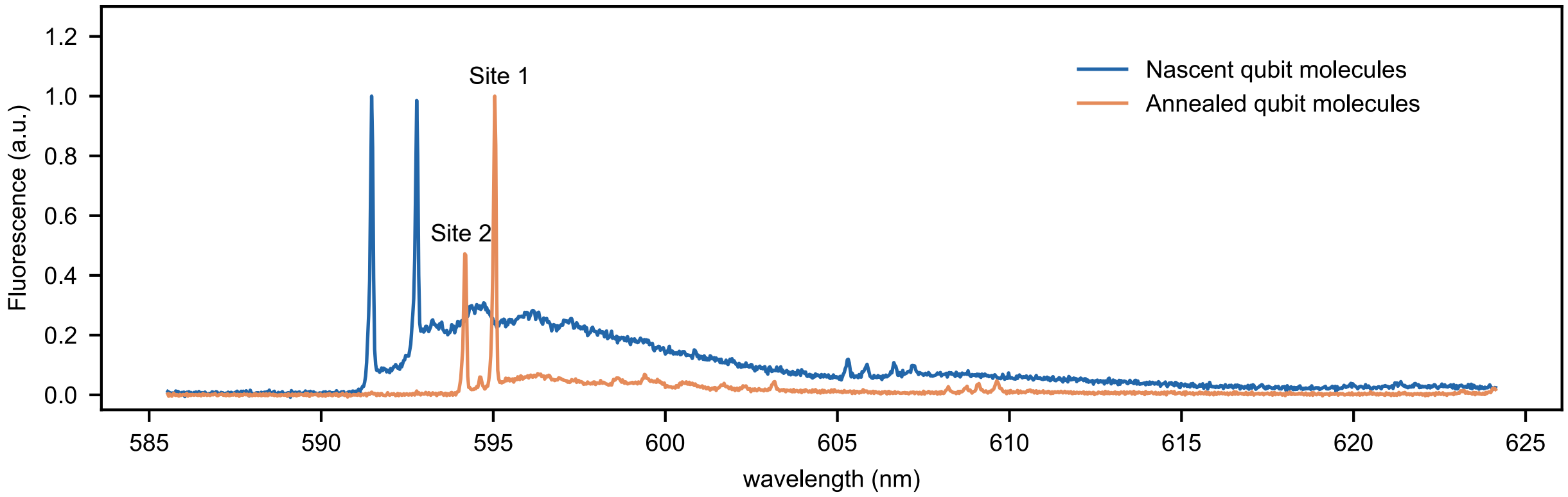

Supplementary Figure 37. Fluorescence spectra for both nascent and annealed qubit molecules in the matrix. Both spectra are normalized to their respective maximum, thus, total fluorescence levels cannot be compared.

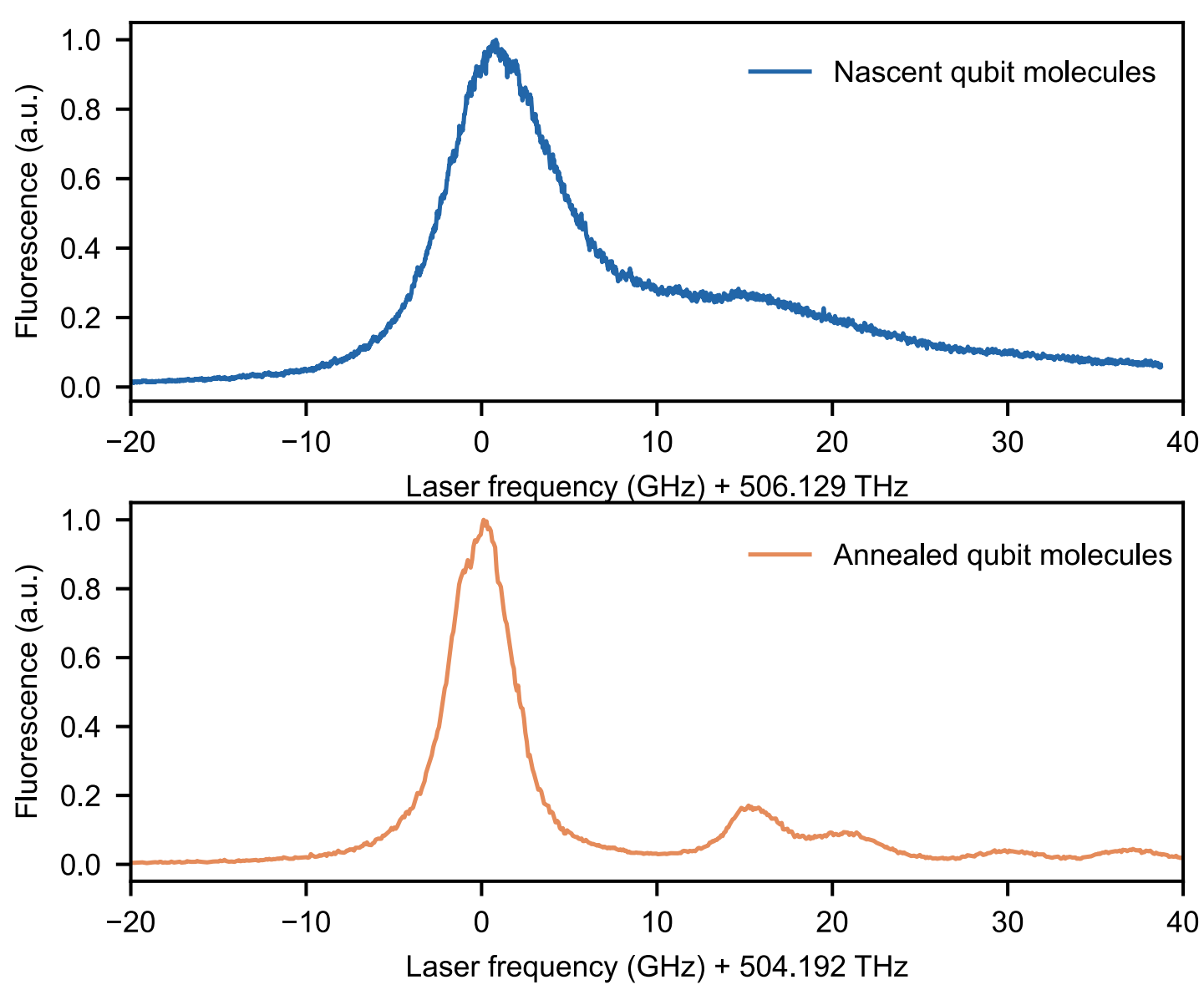

Supplementary Figure 38. Laser excitation spectra for the higher wavelength molecular site for both nascent and annealed di([1,1'-biphenyl]-4-yl)carbene embedded in di([1,1'-biphenyl]-4-yl)methanone. For both measurements, microwave driving has been applied on the $T_{0\mathrm{z}}$ to $T_{0\mathrm{x}}$ and $T_{0\mathrm{z}}$ to $T_{0\mathrm{y}}$ transition. In both cases, the laser frequency was offset to the respective overlap of the $T_{0\mathrm{x}}$ to $T_{1\mathrm{x}'}$ and $T_{0\mathrm{y}}$ and $T_{1\mathrm{y}'}$ transition (as in the main text).

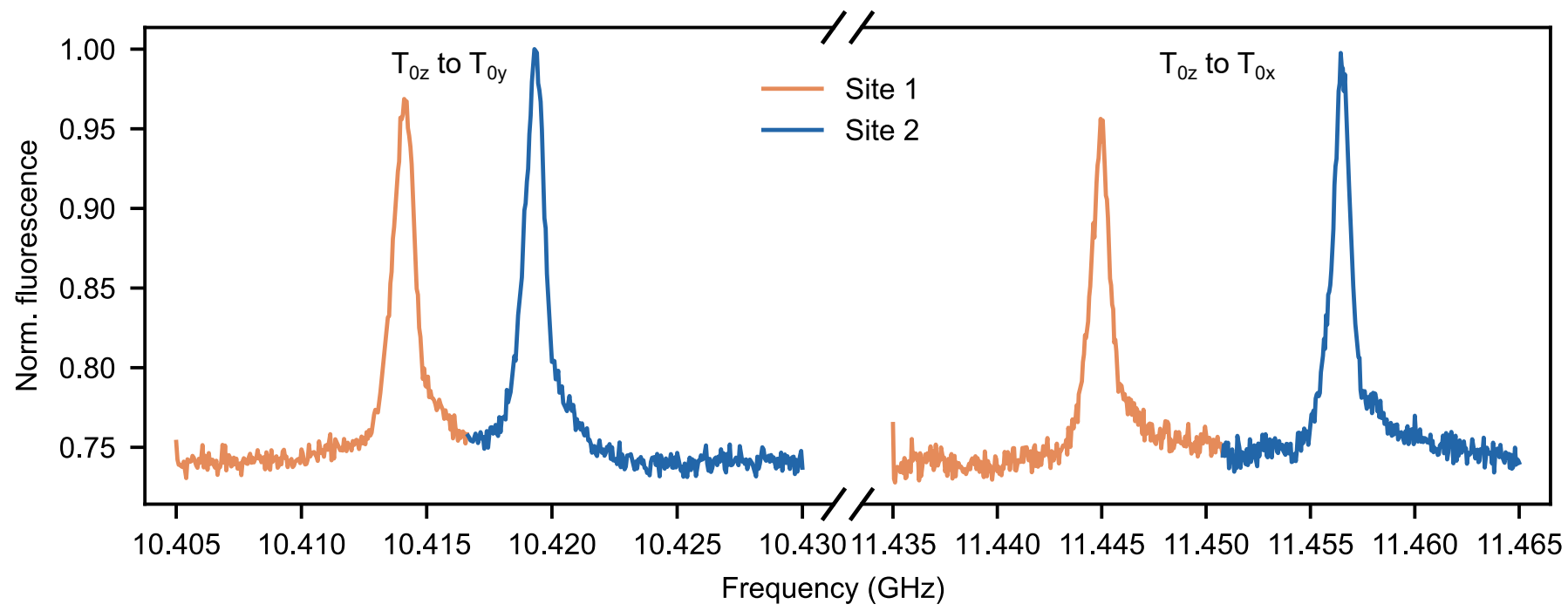

Supplementary Figure 39. Full ODMR spectrum, recorded using off-resonant laser excitation at 580 nm and laser power of 270 nW. The signal stems from both molecular sites of **nascent** di([1,1'-biphenyl]-4-yl)carbene molecules, and shows two pairs of ODMR lines corresponding to the T_{0z} to T_{0x}/T_{0y} transitions of both molecule classes.

D. Off-resonant ODMR on both annealed molecular sites

In order to gain more insight into the nature of site 2 (shorter wavelength emission, see Fig. 2a), ODMR spectra were recorded using off-resonant laser excitation at a wavelength of 580 nm (see Supplementary Fig. 40).

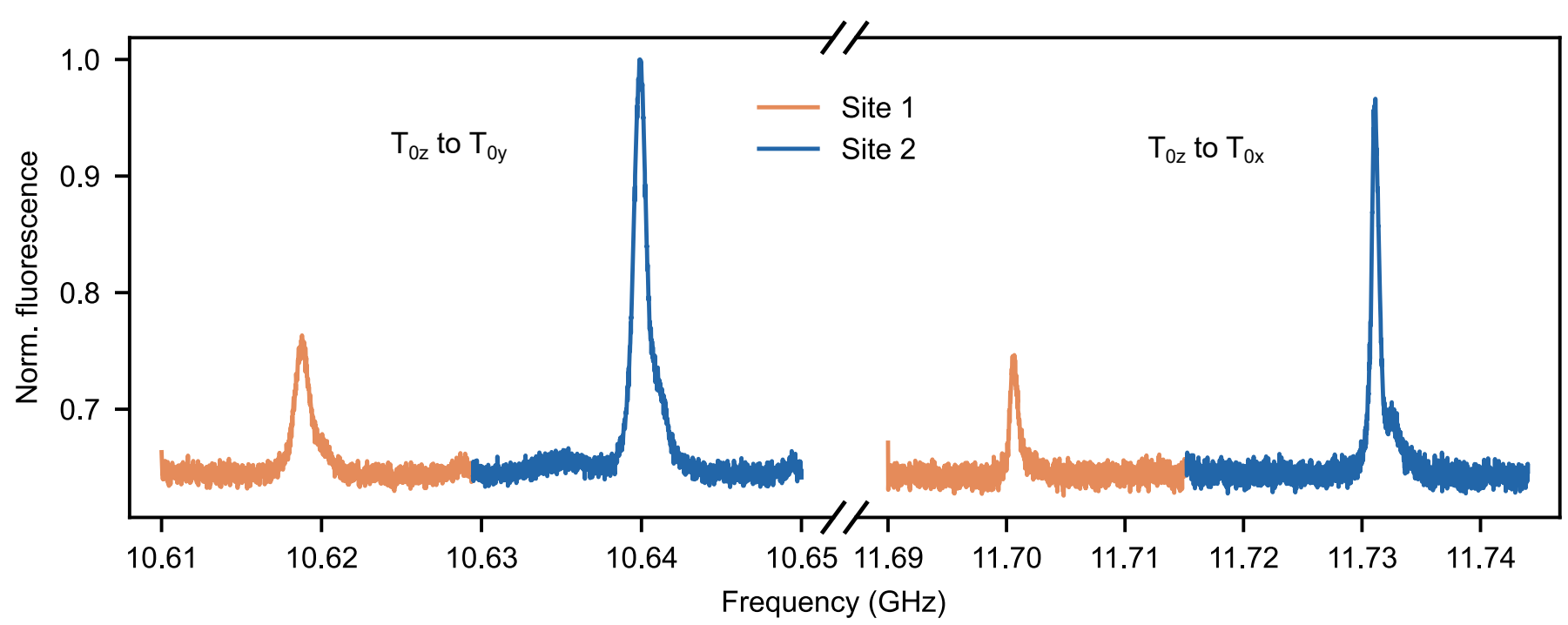

Supplementary Figure 40. Full ODMR spectrum, recorded using off-resonant laser excitation at 580 nm and laser power of 270 nW. The signal stems from both **annealed** molecular sites (compare Fig. 2a in the main text), and shows two pairs of ODMR lines corresponding to the T_{0z} to T_{0x}/T_{0y} transitions of both molecule classes.

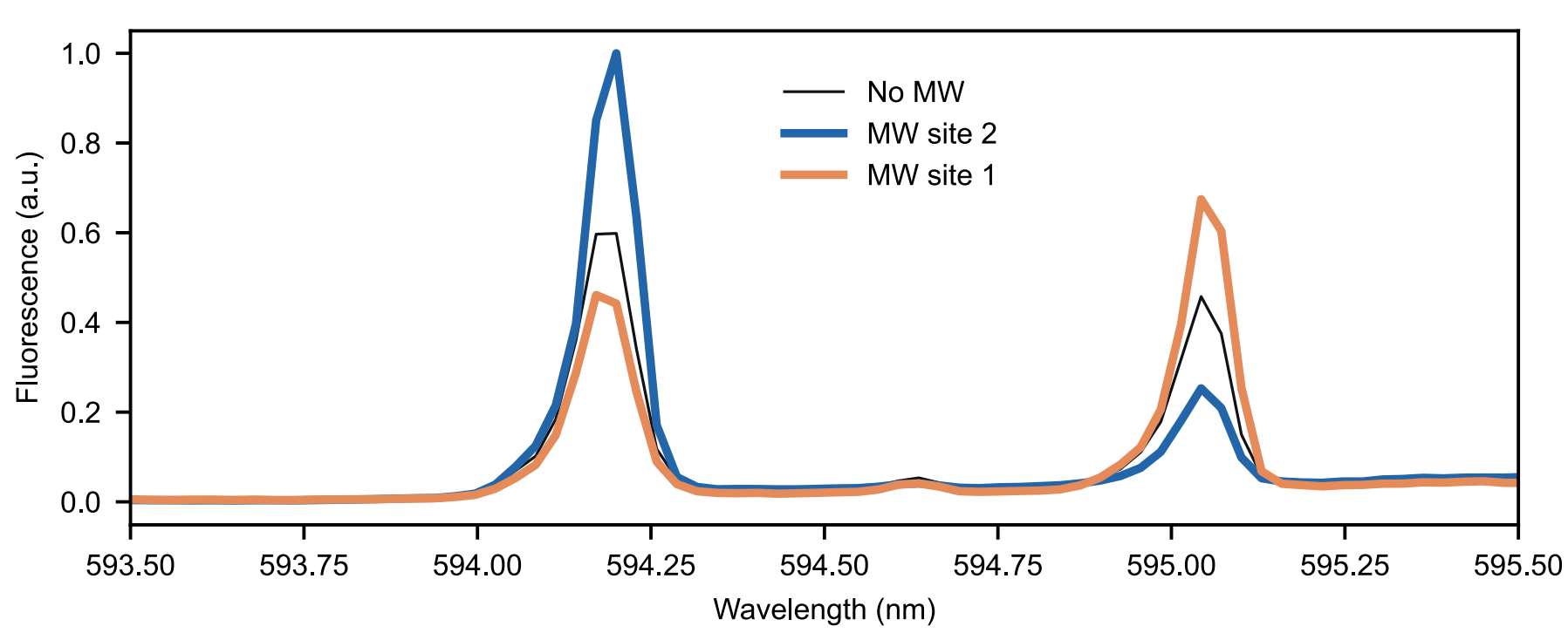

Supplementary Figure 41. Fluorescence Spectrum, zoomed-in to the ZPLs of the two molecular sites. Excitation was performed using laser wavelength of 580 nm and laser power of 270 nW. In addition, microwave radiation was applied to manipulate the fluorescence intensity from both sites separately.

To further strengthen the claim, that the two ZPLs in the emission spectrum correspond to the two respective pairs of ODMR lines and assign the individual lines, fluorescence spectra are recorded while applying microwave signals on one or the other pair of ODMR resonances. Since the applied microwaves modulate the fluorescence intensity of the corresponding site, assignment of ODMR frequency to molecular site can be performed. By taking into account the transition frequencies $T_{0z} \leftrightarrow T_{0y}$ at 10.640 GHz or $T_{0z} \leftrightarrow T_{0x}$ at 11.731 GHz for molecular site 2 (Supplementary Fig. 40), the ground-state ZFS parameters for molecular site 2 were deduced to be $|D_{T_0,\text{site 2}}| = 11.1860(2)$ GHz and $|E_{T_0,\text{site 2}}| = 546.0(2)$ MHz.

E. Determination of the Debye-Waller factor

Supplementary Fig. 42 shows a full emission spectrum of annealed di([1,1'-biphenyl]-4-yl)carbene in di([1,1'-biphenyl]-4-yl)methanone. In order to derive the Debye-Waller factor, especially for site 1, the modulating property of microwave driving site-selective spin transitions is used to create a difference spectrum, that only contains contributions from molecular site 1 (see Supplementary Fig. 41 for the underlying effect, and Supplementary Fig. 43 for the resulting single site spectrum). Fluorescence background is mostly removed by offsetting the spectral region ≤590 nm to an average of 0. From this, the Debye-Waller Factor can be determined as the ratio of intensity within the ZPL divided by the intensity in the spectral window between ZPL and 720 nm, resulting in 19.47%.

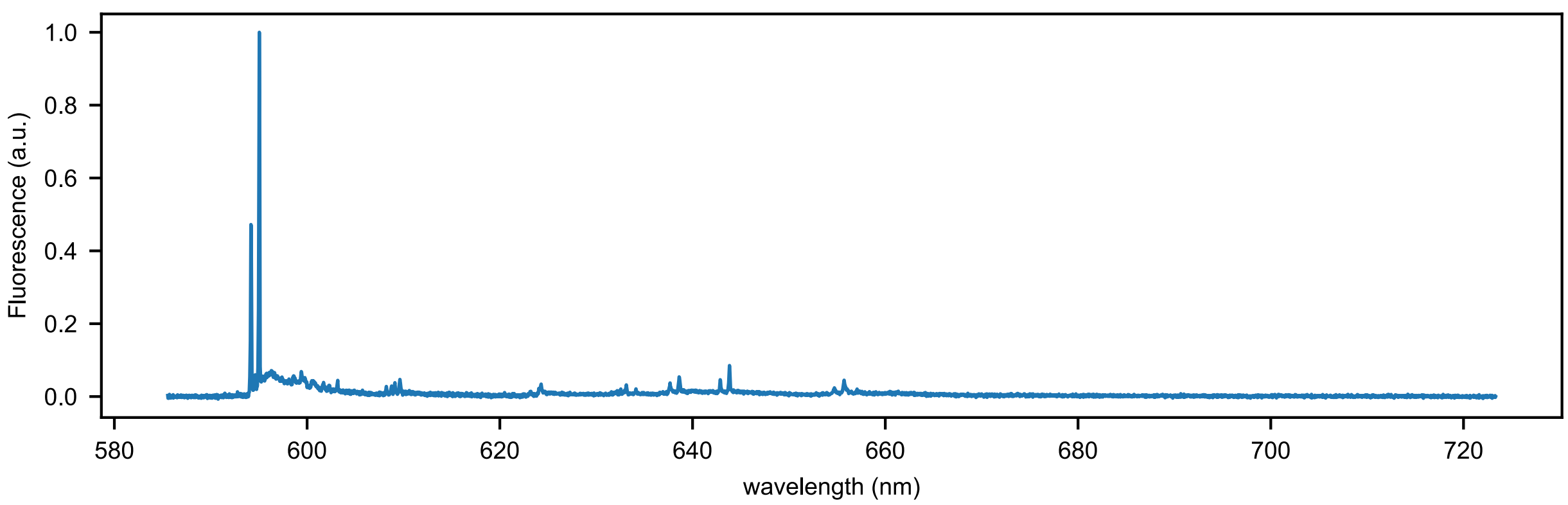

Supplementary Figure 42. Full fluorescence emission spectrum, showing two molecular sites. Excitation was performed using laser wavelength of 580 nm and laser power of 270 nW.

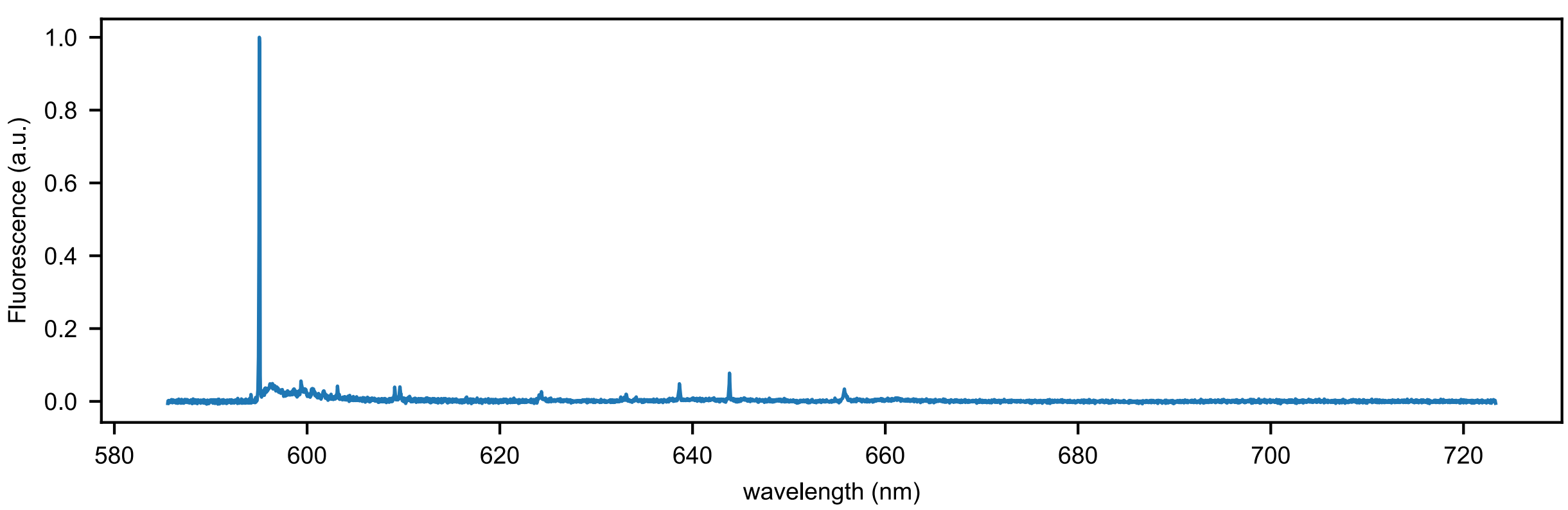

Supplementary Figure 43. Full fluorescence emission spectrum, showing only molecular site 1. Excitation was performed using laser wavelength of 580 nm and laser power of 270 nW. In order to remove site 2, the full emission spectrum was modulated using site selective microwave driving, and subtraction.

F. Fluorescence saturation measurements

In order to assess the brightness of the molecule, one would usually perform the measurement of the fluorescence count rate under saturating excitation power. In the prevalent case, however, some further considerations need to be taken into account. First, single molecule experiments are performed spectrally selective, both for the respective molecule and the addressed optical transition. Due to the highly spin polarizing ISC pathway to the T_{0z} state, this causes a high transient but low steady-state fluorescence intensity (see Methods "Cryogenic Microscopy and Spectroscopy Experiment"). By applying MW radiation on the ZX and/or the ZY transition, fluorescence intensity is strongly increased, which ultimately enables the detection of single molecules, as performed in Fig. 3 a-f in the main text (for the effect of enhancing the fluroescence intensity, also compare the blue/orange excitation spectrum (with MW) to the black excitation spectrum (no MW) in Fig. 2b). We perform saturation measurements on the YY' transition for different Rabi frequencies applied to the ZY transition, while simultaneously applying a weak Rabi drive of 69 kHz on the ZX transition. The resulting saturation curve is shown in Supplementary Fig. 44). In order

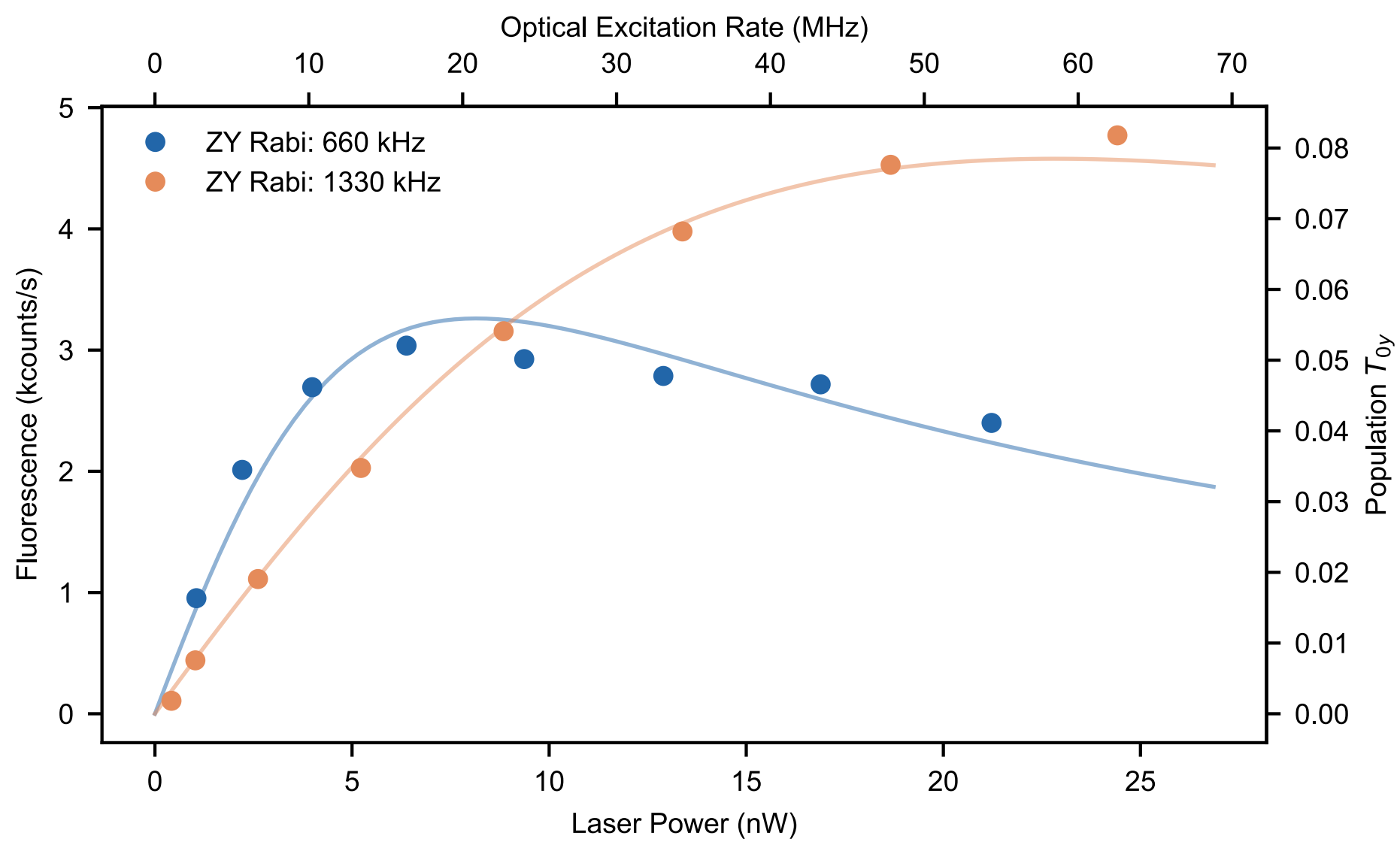

Supplementary Figure 44. Saturation fluorescence measurement for a single molecule excited on its XX' transition. The right y axis shows the simulated population probability of $T_{1y'}$ in the steady state, the upper x axis shows the optical excitation rate used as a parameter in the simulation.

to verify the observed behavior (especially the decrease of fluorescence intensity with increasing laser power in the blue curve), we fit a parameterized simulation in QuTIP[40] to the measurement data. The modeled system consists of seven energy states (three triplet ground states, three triplet excites states and one singlet state). The behavior shown in Supplementary Fig. 44 is obtained by setting the following parameters

Parameter	Description	Value
c_{laser}	conversion factor laser power to excitation rate	2.56 MHz/nW
$r_{\text{Y'Y}}$	optical decay rate	37.74 MHz
$r_{\text{Y'S}}$	ISC rate $T_{1y'}$ to S	3.997 MHz
r_{SY}	ISC rate S to T_{0y}	2.035 MHz
r_{SX}	ISC rate S to T_{0x}	2.035 MHz
r_{SZ}	ISC rate S to T_{0z}	10 MHz
T_1	T_1 spin relaxation time	21 ms
$f_{\text{Rabi,ZX}}$	Rabi frequency ZX	69 kHz

Supplementary Table 11. Model parameters used to create the behavior as seen in the solid line in Supplementary Fig. 44

From the model, the maximum count rate for the case of a Rabi frequency of 1.33 MHz can be determined to be $I_{\max} = 4.58$ kcounts/s at a laser power of $P_{\max} = 22.79$ nW. Interestingly, the model fit also allows for determination of the PLQY. Looking at the point of highest fluorescence (4.58 kcounts/s at 22.79 nW), and using the conversion factors in Supplementary Table 11, one obtains an optical excitation rate of $r_{\text{YY'}} = 58.4$ MHz and an population

probability of the T_{0y} state of $P_{T_{0y}} = 0.0785$. While $r_{Y'Y}$ denotes the rate between the states $T_{1y'}$ and T_{0y}, the number of de-excitations on this channel can be calculated to be

$$r_{\text{photons,max}} = P_{T_{1y'}} \cdot r_{Y'Y} = 2.96\,\text{MHz}. \tag{6}$$

This represents the maximum number of photons that are emitted, assuming a steady state of the system as reached in Supplementary Fig. 44. For the performed experiment, we expect a photon detection efficiency of 1.3% (see SI Sec. VII A 4), which would result in a maximum rate of photon detections of

$$r_{\text{photons,max,detected}} = 0.013 \cdot r_{\text{photons,max}} = 38.48\,\text{kHz}. \tag{7}$$

Taking the actually detected photon rate of $I_{\text{max}} = 4.58\,\text{kHz}$ into account, it allows for the deduction of a quantum efficiency of the Y'Y transition of QE =11.9%. Additionally, the rate of photoexcitations can be deduced to be

$$r_{\text{photoexcitations}} = P_{T_{0y}} \cdot r_{YY'} = 3.27\,\text{MHz}, \tag{8}$$

and allows the determination of a

$$\text{PLQY} = \frac{r_{\text{photons,max}}}{r_{\text{photoexcitations}}} \cdot \text{QE} = 10.8\%. \tag{9}$$

It should be noted, that this value incorporates quite a few assumptions, and should not be considered an accurate determination of the PLQY. Nevertheless, considering that in this experiment we are using resonant excitation of one of the two bright triplet subspaces, there is a striking resemblance of this value with the PLQY measured in a frozen solution of molecules (see SI Sec. III B).

G. Photo-bleaching in ensembles of di([1,1'-biphenyl]-4-yl)carbene

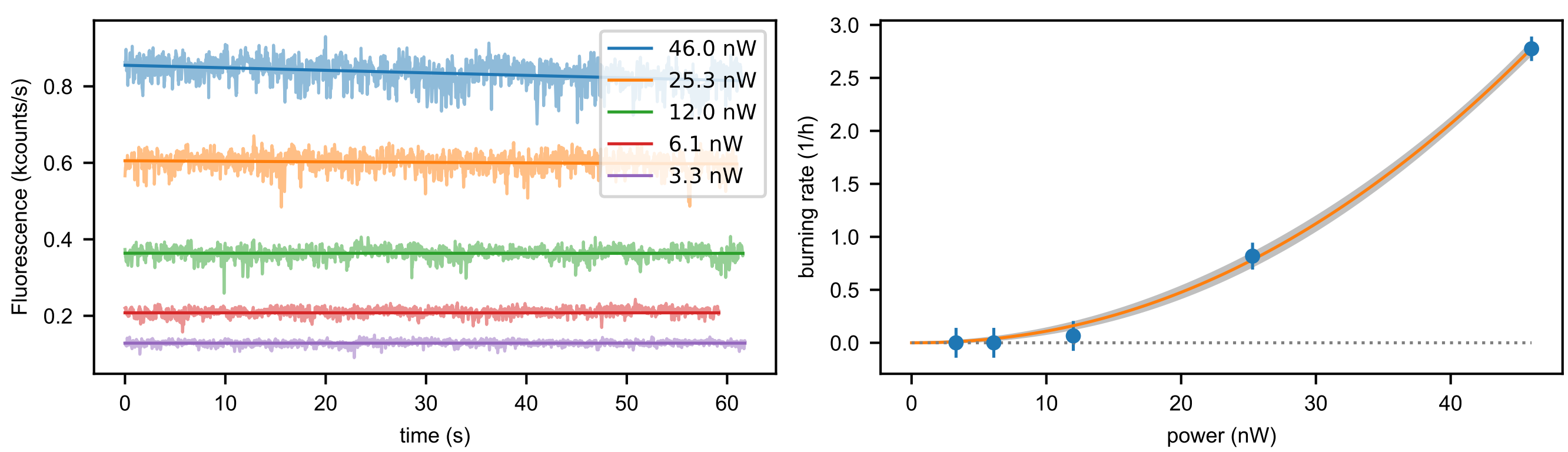

Supplementary Figure 45. Left, Fluorescence time traces of an ensemble of annealed di([1,1'-biphenyl]-4-yl)carbene molecules for laser excitation resonant to the YY' ZPL of the ensemble and different powers. In addition, microwave fields are applied on spin transitions ZX and ZY. A decay is fit to the traces. Right, The decay rates extracted from the left panel are plotted vs. the laser power. A power law is fit to the data.

During the course of the experiments presented in the main text some single di([1,1'-biphenyl]-4-yl)carbene molecules stop fluorescing once in a while. In the following we refer to these events as burning, though, we cannot exclude a mere spectral jump of their optical transition frequency. Such a jump in our case is hardly detectable due to the dense spectrum full of individual emitters. Typically, emitters are stable within a few hours of a working day. Starting new experimental runs on a next day includes setting up confocal microscopy, laser and microwave fields. Slight deviations of parameters from day to day prevent continuation of experiments on the same emitter next day, but choosing an emitter suitable for the newly set parameters.
To assess the stability of di([1,1'-biphenyl]-4-yl)carbene molecules during optical excitation we, therefore, investigate ensembles of molecules. In addition, we investigate stability also for higher laser powers than those used for the experiments described in the main manuscript. Ramping up the laser power we need to take care that we set higher Rabi frequencies on the spin transitions of the ground state triplet to not shelve the molecules into a dark state and

prevent optical excitation (see SI Sec. VII F).

We set spin Rabi frequencies of ≈ 0.07 MHz and ≈ 1 MHz on the ZX and ZY transitions, respectively, and we apply the laser resonant to YY' ZPL of the ensemble of molecules. Doing so we slightly populate $T_{1y'}$ and thus invoke fluorescence but we also pump the system into dark states T_{0z}, T_{0x} and the metastable singlet state. The singlet state seems to decay relatively fast ($\gtrsim 2$ MHz, see SI Sec. VII F) back into the ground state (mainly into T_{0z}), but the other spin states are much more stable and are therefore equilibrated by the microwave drives. We record the fluorescence time traces for different laser powers and observe a decrease over time (see Supplementary Figure 45). From the relative decrease of fluorescence over time we estimate an average decay rate γ_{burn} for the different laser powers and fit a power law $\gamma_{\mathrm{burn}} = \gamma_{\mathrm{nW}} \cdot P^m$ to the laser power (P) dependence (see Supplementary Figure 45, $m = 2.1 \pm 0.1$ and $\gamma_{\mathrm{nW}} = (8.4 \pm 3.4) \cdot 10^{-4}\,\mathrm{h}^{-1} \cdot \mathrm{nW}^{-m}$). The latter fit is in agreement with a two-photon-process for burning. From the ensemble measurements we estimate an average lifetime for continuous illumination with 3 nW and microwave application of ≈ 119 hours. Note, that under these conditions the temperature of the sample has increased by about 1 K.

H. Ensemble excitation spectra and ^{13}C isotope effects

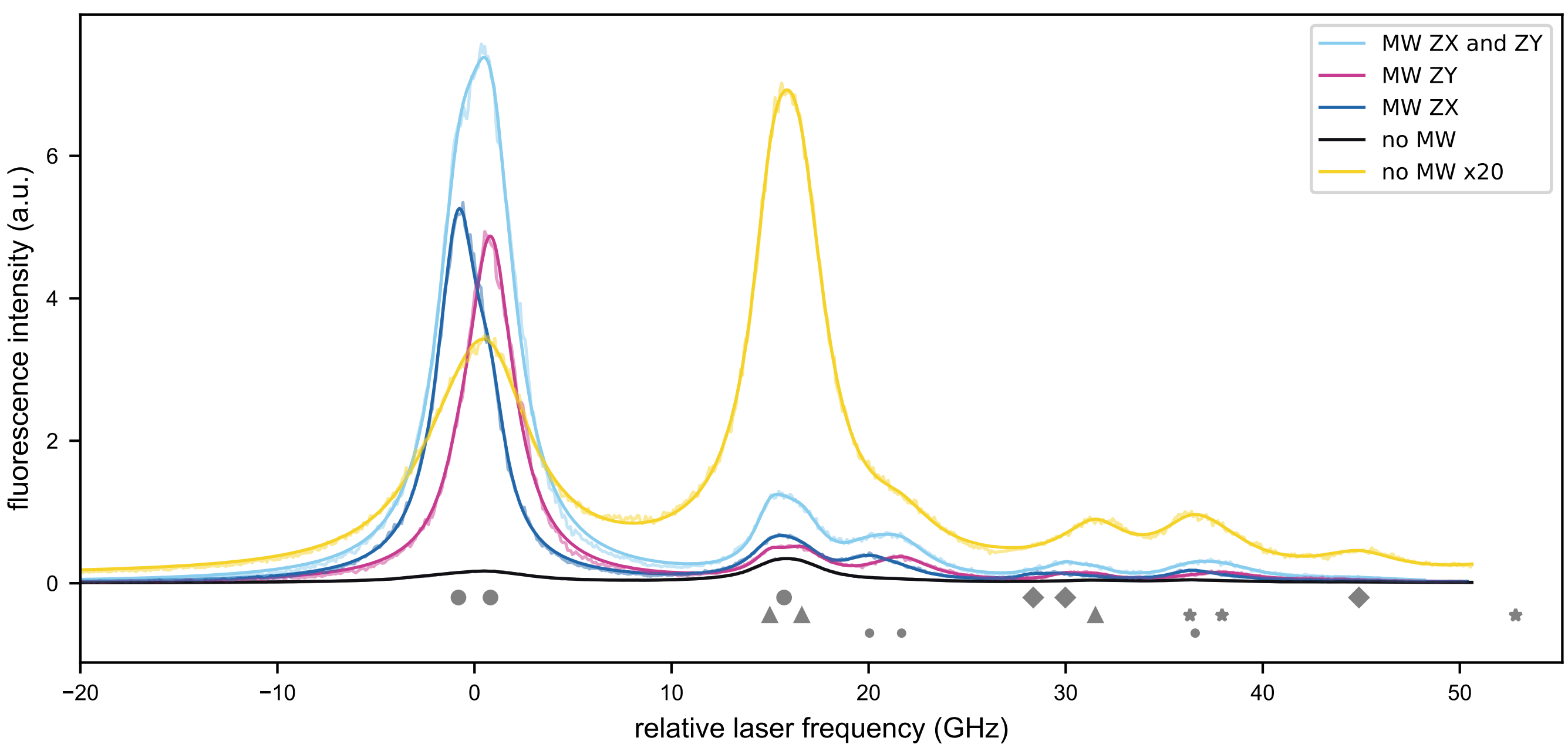

Supplementary Figure 46. Fluorescence excitation spectra of an ensemble of qubit molecules in crystal site 1 for four cases of microwave fields: MW resonant to $T_{0z} \leftrightarrow T_{0x}$, MW resonant to $T_{0z} \leftrightarrow T_{0y}$, MW resonant to both transitions, all MW fields off (see legend). Five sets of each three Lorentzians are fit to the data. The spectral positions of the three Lorentzians of each set are marked by a symbol underneath; and each of the sets has a different symbol (circle, triangle, dot, diamond and star). The frequency separation within each triplet are identical.

The excitation spectra of ensembles of qubit molecules depicted in Supplementary Fig. 46 show multiple resonances with varying amplitudes depending on the type of MW application. We attribute the strongest peak of each of the curves labeled "mw ZX", "mw ZY" and "no mw" (marked by a circle below) to optical transitions $T_{0x} \rightarrow T_{1x'}$, $T_{0y} \rightarrow T_{1y'}$ and $T_{0z} \rightarrow T_{1z'}$, respectively. As described in the text, MW radiation that is resonant to spin transitions $T_{0z} \leftrightarrow T_{0x}$ and $T_{0z} \leftrightarrow T_{0y}$ recycles molecules into optical excitation and emission that have previously been shelved into the dark spin state T_{0z}. The latter process mainly describes the behavior of the two strongest optical resonances around 0 GHz. The optical transition $T_{0z} \rightarrow T_{1z'}$ around 15 GHz is almost unaffected. When looking at the expected relative fluorescence intensities of the three excited triplet states (see eq. (18), $p_{\mathrm{F,x'}}, p_{\mathrm{F,y'}} : p_{\mathrm{F,z'}} \approx 5:1$), their relative ISC rates (see eq. (17), $\gamma_{\mathrm{ISC,z'}} : \gamma_{\mathrm{ISC,x'}}, \gamma_{\mathrm{ISC,y'}} \gtrsim 5:1$) and the ratios of the ISC from the metastable to the ground state triplets ($\gamma_{\mathrm{ISC,z}} : \gamma_{\mathrm{ISC,x}}, \gamma_{\mathrm{ISC,y}} \sim 6000:1$, see SI Sec. IV F 8 and Supplementary Figure 28) favoring polarization into T_{0z}, we can tentatively explain this behavior. Although, $T_{1x'}$ and $T_{1y'}$ are much more fluorescent than $T_{1z'}$, eventually, they are pumped into the dark spin state T_{0z} via ISC and fluorescence seizes. $T_{1z'}$, however, despite being

dimmer is very likely to end up in T_{0z}, either via fluorescence, ISC or non-radiative decay, from where it can be excited again, finally yielding more fluorescence when microwaves are off. When population in the ground state triplet is efficiently redistributed via microwaves $T_{1x'}$ and $T_{1y'}$ exhibit much more fluorescence than $T_{1z'}$. All weaker than the previously mentioned optical resonances in Supplementary Fig. 46 that are marked with triangles, dots, diamonds and stars cannot be explained as optical transitions of the same molecules that lead to the strongest resonances; they have to belong to molecules that differ to some extent. Our hypothesis is the existence of at least five optically distinguishable classes of molecules (five different symbols in the figure), each with triples of optical resonances that have the same intra-triple separation but each triple is shifted with respect to each other. Therefore, we have fit 5×3 Lorentzian lines to each of the four spectra in Supplementary Fig. 46 with same frequencies across spectra but varying widths and amplitudes. Furthermore, three Lorentzians of a triple always have the same mutual separation.

The main triple of resonances (marked by a circle) in the gray curve (i.e. "mw ZX and ZY") has a relative area of 0.789 compared to the total area. Interestingly, the probability for all 25 carbon atoms of our qubit molecule to be ^{12}C is 0.758 for a natural abundance of 0.989. This similarity motivated the consideration of isotope effects to slightly shift optical resonances of otherwise identically packed molecules inside the ketone crystal. Indeed, such isotope effects have been investigated before for other optically narrow band molecular emitters like pentacene and terrylene in p-terphenyl crystals and for singlet to triplet excitation spectra in anthracene and naphthalene crystals.[41–44] In the latter reference targeted isotopic enrichment was performed to correlate different strengths of isotopic shifts with specific ^{13}C locations on the molecules.

Summarizing, our hypothesis for interpreting the spectra in Supplementary Fig. 46 is the existence of several subensembles of di([1,1'-biphenyl]-4-yl)carbene molecules that belong to the same crystal site (i.e. site 1, see Sec. VII D) but differ in isotopic composition. Most of the molecules are supposed to have a pure ^{12}C backbone while all other molecules might have one or more ^{13}C isotopes spread over the molecule. Depending on the number and position of the ^{13}C isotopes on the molecules, the influence on the ZPL shift might be different and thus leads to several additional resonances in the fluorescence excitation spectra. Although, we have fit 5 molecular subensembles to the spectra, there might be more than that. Some might be hard to resolve, others might be too weak to be identified due to probability, and others might just not be sufficiently bright because the necessary microwave transition frequency might be different than for the molecules comprising the main resonances. Supplementary Figure 47 shows ODMR resonances for three different optical excitation frequencies potentially belonging to at least three different isotopic compositions. The resonances are slightly shifted with respect to each other and are split in one case. The addition of a single ^{13}C nuclear spin to a molecule that consists of ^{12}C isotopes otherwise doubles the amount of energy levels within the electron spin triplet ground state. However, at zero magnetic field the ^{13}C spin shifts the energy depending on hyperfine interaction but does not lift the degeneracy of the new spin level pairs. Therefore, we do not expect to necessarily find more ODMR resonances than without any ^{13}C spin. One of the spectra in Supplementary Fig. 47 exhibits two resonances instead of one. Reasons might be for example more than one ^{13}C spin per molecule or the

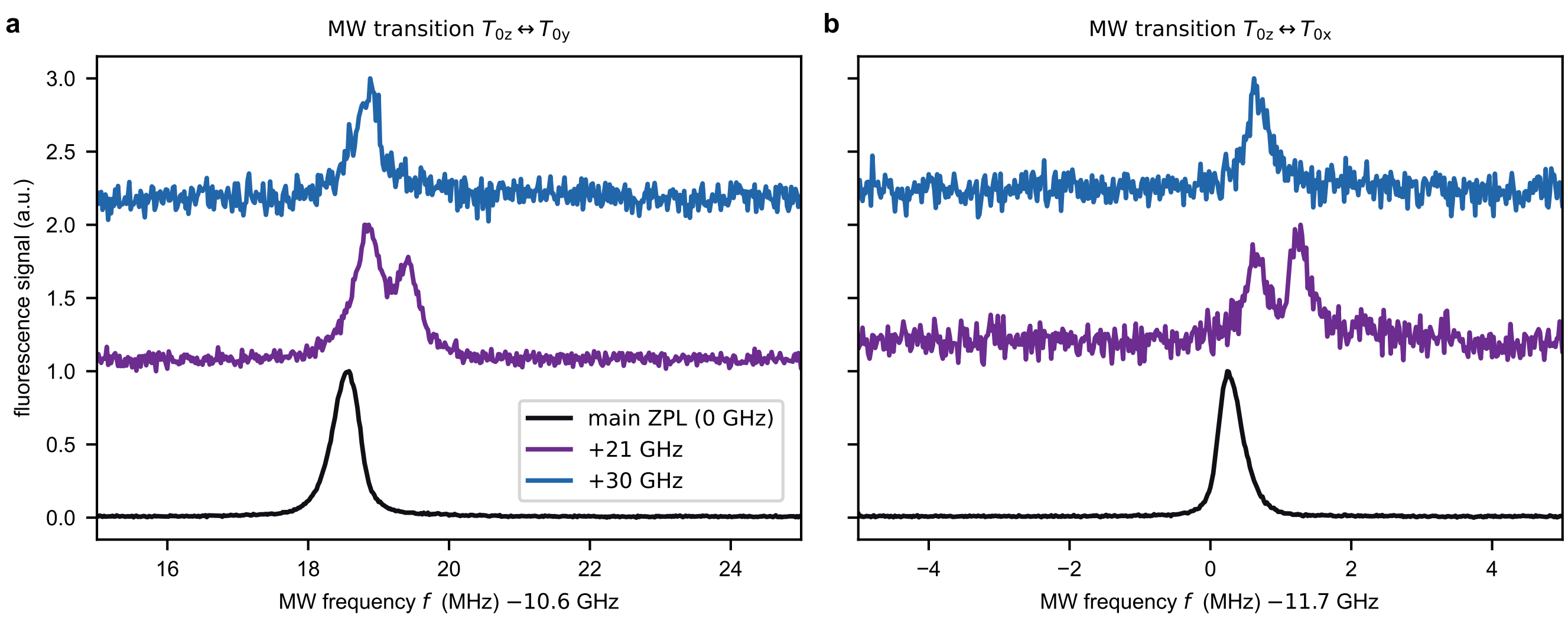

Supplementary Figure 47. ODMR spectra on spin transitions (a), $T_{0z} \leftrightarrow T_{0y}$ and (b), $T_{0z} \leftrightarrow T_{0x}$, each for different laser excitation frequencies. Different laser excitation frequencies might excite molecular sub-ensembles that differ in isotopic composition of their carbon backbone (see text).

optical excitation of two sub-ensembles of molecules with similar optical frequency but different location of a single ^{13}C spin on the molecule.

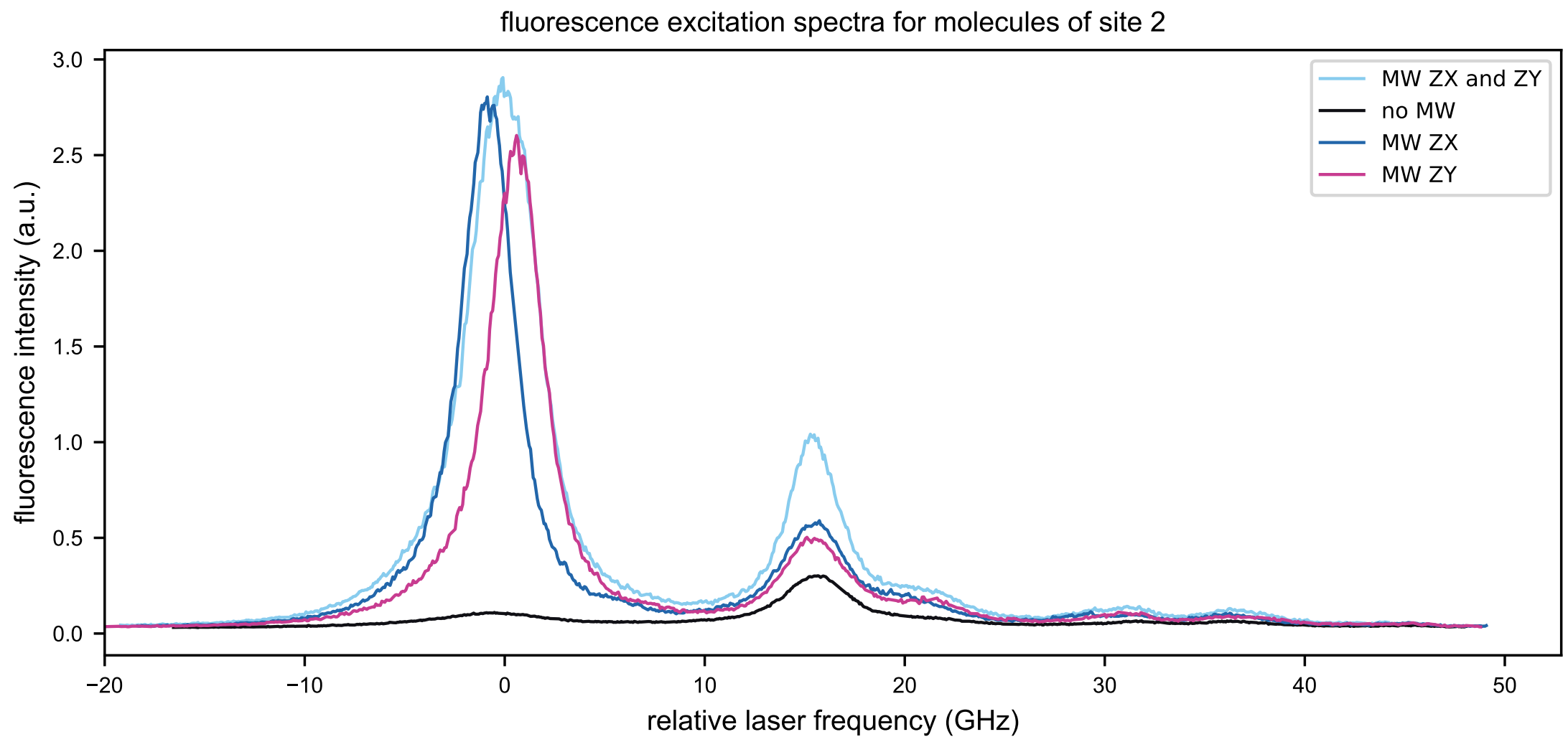

Supplementary Figure 48. Fluorescence excitation spectra of an ensemble of qubit molecules in crystal site 2 for four cases of microwave fields: MW resonant to $T_{0\mathrm{z}} \leftrightarrow T_{0\mathrm{x}}$, MW resonant to $T_{0\mathrm{z}} \leftrightarrow T_{0\mathrm{y}}$, MW resonant to both transitions, all MW fields off (see legend).

Finally, we show the excitation spectra of molecule ensembles in crystal site 2 (see Supplementary Fig. 48). The absolute position of the ZPL of molecules in site 1 and site 2 can be taken from Supplementary Fig. 43. The relative laser frequency given in Supplementary Figs. 46,48 helps comparing both spectra. Both exhibit similar separations of spin selective transitions and isotope shifts.

I. Single molecule spectra and spin state assignment

The inhomogeneously broadened ensemble excitation spectra in Supplementary Fig. 46 exhibit separated spin-selective transitions. For optical transitions $T_{0\mathrm{x}} \rightarrow T_{1\mathrm{x}'}$ and $T_{0\mathrm{y}} \rightarrow T_{1\mathrm{y}'}$, however, the separation is smaller than the inhomogeneous linewidth. The excitation spectra of several individual qubit molecules in Supplementary Fig. 49 shows the same shift but for much narrower optical lines. The depicted cross-correlation of spectra of qubits excited on their $T_{0\mathrm{x}} \rightarrow T_{1\mathrm{x}'}$ transition ($f(XX')$, Supplementary Fig. 49a) or on their $T_{0\mathrm{y}} \rightarrow T_{1\mathrm{y}'}$ transition ($f(YY')$, Supplementary Fig. 49b) reveals an average splitting Δf_{XY} of these optical transition frequencies f of

$$\begin{aligned} \Delta f_{XY} &= f\left(YY'\right) - f\left(XX'\right) = 1.555(1)\,\mathrm{GHz} \\ &= 2E_{\mathrm{es}} - 2E_{\mathrm{gs}} \\ &= 2E_{\mathrm{es}} + 1.0818(2)\,\mathrm{GHz} \end{aligned} \tag{10}$$

with a width of the correlation peak of 143(4) MHz, which is about three times larger than the single emitter linewidth (Supplementary Fig. 49d).

From spin-selective optical line separation Δf_{XY} and the ground state ZFS parameter E_{gs}, the excited state ZFS parameter E_{es} can be estimated to be $E_{\mathrm{es}} \approx 237(1)\,\mathrm{MHz}$. The last piece of information to fully estimate the energy levels of the ground and excited triplet states is the excited state ZFS parameter D_{es}. This requires information, for example, from the excited state ODMR frequencies $f(Z'Y') = 3.958(1)\,\mathrm{GHz}$ and $f(Z'X') = 4.423(1)\,\mathrm{GHz}$ of spin transition $T_{1\mathrm{z}'} \leftrightarrow T_{1\mathrm{y}'}$ and $T_{1\mathrm{z}'} \leftrightarrow T_{1\mathrm{x}'}$, respectively (see Supplementary Fig. 51). The latter measurements also give another estimate for E_{es}, which differs by 5 MHz from the previous estimate.

$$\begin{aligned} D_{\mathrm{es}} &= -\left[f\left(Z'X'\right) + f\left(Z'Y'\right)\right]/2 \\ &= -4.1905(10)\,\mathrm{GHz} \end{aligned} \tag{11}$$

$$\begin{aligned} E_{\mathrm{es}} &= \left[f\left(Z'X'\right) - f\left(Z'Y'\right)\right]/2 \\ &= 0.2325(10)\,\mathrm{GHz} \end{aligned} \tag{12}$$

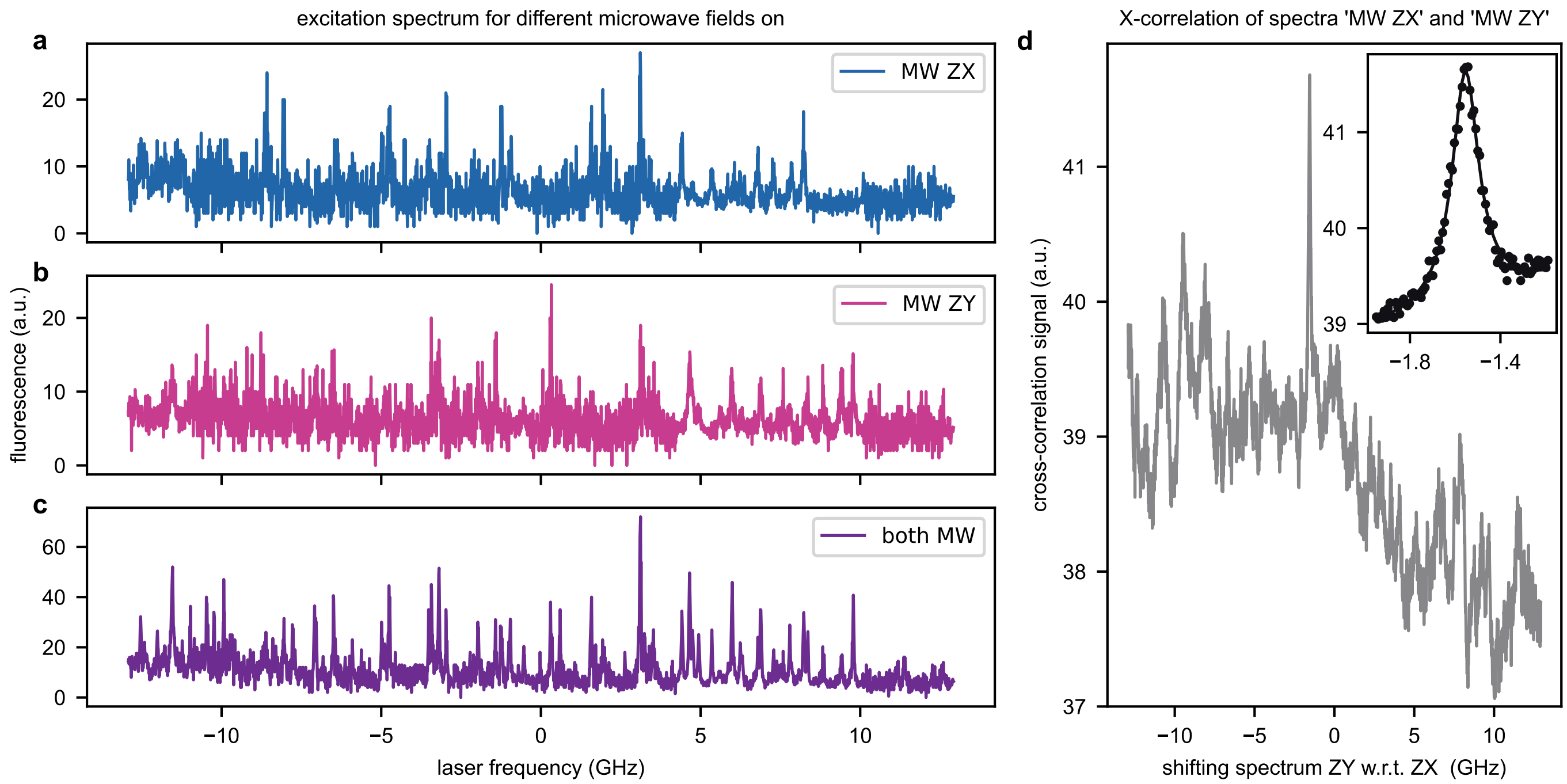

Supplementary Figure 49. Fluorescence excitation spectra of few qubit molecules. (a), An additional MW field is applied resonant to spin transition $T_{0z} \leftrightarrow T_{0x}$, ((b), to $T_{0z} \leftrightarrow T_{0y}$ and (c) to both transitions. (d) Cross-correlation of data in **a** and **b** according to $d_k = \sum_{n=N}^{M-1} a_{n+k} \cdot b_n/(M-N)$. The inset zooms into the region of the maximum correlation value with a local fit of a Lorentzian on a linear slope to the apparent peak. According to the fit, the optical transition $T_{0x} \rightarrow T_{1x'}$ is shifted with respect to $T_{0y} \rightarrow T_{1y'}$ by 1.555(1) GHz to lower frequencies.

The spin-selective optical transition $T_{0z} \rightarrow T_{1z'}$ has not been observed for single molecules, yet. This might be due to the high probability of ISC in the excited state compared to a fluorescent decay into the ground state. From Supplementary Fig. 46 we estimate that the expected fluorescence intensity is more than ten times smaller for excitation on transition $T_{0z} \rightarrow T_{1z'}$ than for transitions $T_{0x} \rightarrow T_{1x'}$ or $T_{0y} \rightarrow T_{1y'}$.

The separation of energy levels with the qubit molecules are summarized in Supplementary Table 12.

	Ground state triplet	Excited state triplet
ZFS D	11.1597(2) GHz	−4.1905(10) GHz
ZFS E	−0.5409(2) GHz	0.2325(10) GHz
	separation of optical transitions	
Δf_{XY}	1.55 GHz	
Δf_{XZ}	16.12 GHz	
Δf_{ZY}	14.58 GHz	

Supplementary Table 12. Summary of experimentally obtained energy level separations of di([1,1'-biphenyl]-4-yl)carbene molecules in a di([1,1'-biphenyl]-4-yl)methanone matrix at around 4.5 K.

J. Temperature dependence of position and width of optical resonances

The investigated carbene molecules do not exhibit transform limited optical transitions in excitation spectra under present conditions (∼ 4.5 K). It is known for example from Terrylene molecules in p-terphenyl, that transitions shift and narrow down upon further reducing temperature.[41] Following previous observations in molecular emitters, we employ an empirical Arrhenius description to parameterize thermally activated broadening and line shifts. The current experimental setup does not allow for further cooling but we have instead increased temperature slightly and investigated the behavior of optical transitions of single carbenes. Supplementary Figure 50a shows the excitation spectra of a single carbene molecule for different temperatures between 4.67 K and 9.5 K. As the temperature increases

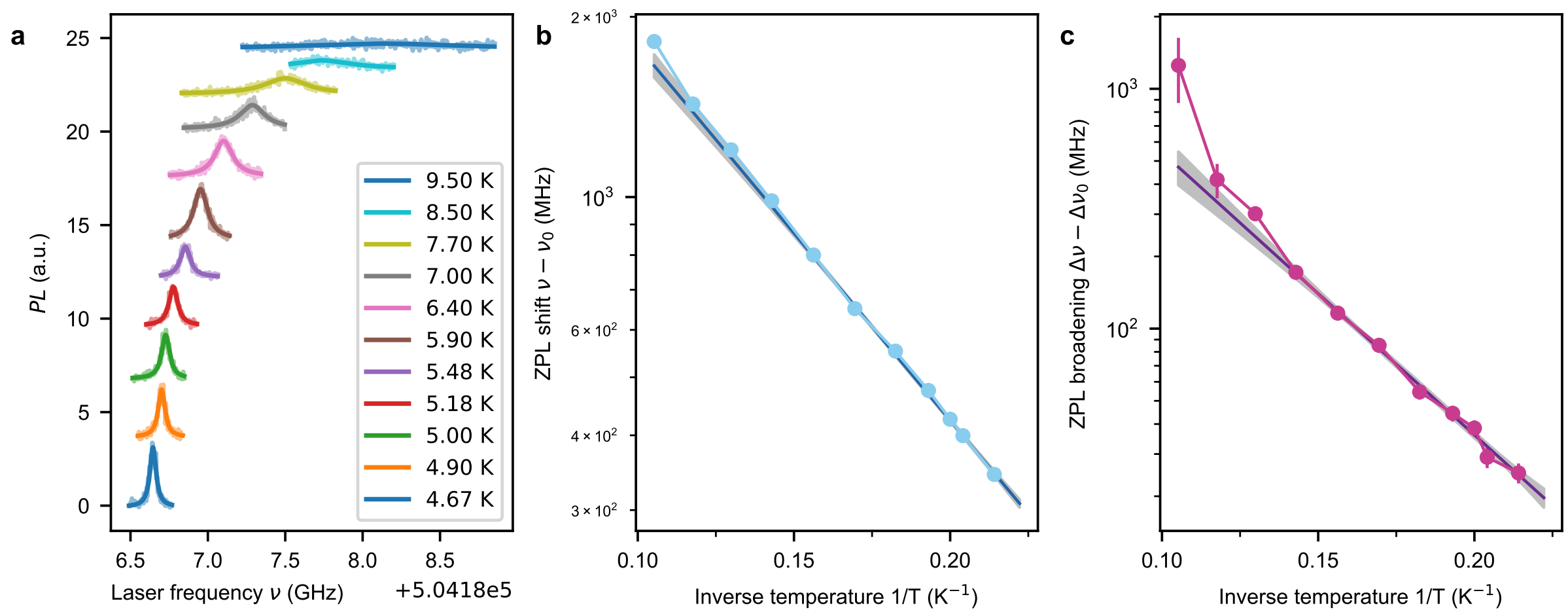

Supplementary Figure 50. Temperature dependence of single carbene optical transitions. **a**, fluorescence excitation spectra for different temperatures. Offsets are used for better discrimination. **b**, Arrhenius plot and fit of the shift of the ZPL with temperature. **c**, Arrhenius plot and fit of the additional line broadening of the ZPL with temperature.

the resonance shifts towards higher frequencies, broadens and reduces in amplitude significantly up to a point where the resonance is hardly detectable. A Lorentzian line is fit to all spectra and the extracted resonance position and linewidth are plotted against temperature in Supplementary Figures 50b and c respectively. As in[41] an Arrhenius law can be fit to both curves.

$$\nu - \nu_0 = \delta_\infty \exp\left(-T_E/T\right) \tag{13}$$

$$\Delta\nu - \Delta\nu_0 = \Delta_\infty \exp\left(-T_E/T\right) \tag{14}$$

The fit parameters yield for the line position $\nu_0 = 504.18630(5)$ THz, for the shift at infinite temperatures $\delta_\infty = 8(1)$ GHz and an activation energy of $k_B T_E = k_B \cdot 14(1)$ K. For the linewidth the parameters are $\Delta\nu_0 = 23(7)$ MHz for the width limit at zero temperature, a width at infinite temperature of $\Delta_\infty = 8(4)$ GHz and an activation energy of $k_B T_E = k_B \cdot 27(4)$ K. The activation energies correspond to frequencies of 292(20) GHz and 563(80) GHz respectively and might be associated with resonances in the phonon sideband (see Supplementary Fig. 43). The estimated linewidth limit $\Delta\nu_0$ is about half the value of the emitter at 4.67 K, however, still not lifetime limited. Further experiments at lower temperature will be required to get a better estimate.

K. Optically detected magnetic resonance of the excited state

For efficient ODMR in the excited state a sufficiently strong resonant microwave field is required to flip the spin with the excited state lifetime. Here, such fields should correspond to Rabi frequencies of a few 10 MHz. We have reduced the maximum MW field because of too strong heating and therefore sacrificed contrast in these measurements. Supplementary Fig. 51 shows the excited state ODMR spectrum and describes the pulse sequence utilized for ensembles of molecules.

L. Fluorescence lifetime measurements

In the cryo-confocal setup excited state lifetime measurements were performed on single molecules and for resonant optical excitation compared to off-resonant excitation of molecules ensembles as described in III C. To this end, the dye laser light was set on resonance with transition $T_{0y} \leftrightarrow T_{1y'}$ and was chopped by an AOM and an EOM. While the AOM achieves a higher on/off contrast ($\approx 10^4$), switching it is slower than the EOM. Therefore we cascaded AOM and EOM laser switching. The total pulse sequence starts with a MW π-pulse on the $T_{0z} \leftrightarrow T_{0y}$ transition, preparing the ground state spin in its T_{0y} state. Then the AOM turns on and shortly after the EOM. After 500 ns the EOM turns off and the AOM remains on for another 450 ns. The excited state lifetime is extracted from the fluorescence decay after the EOM is switched off (see Supplementary Fig. 52). To estimate the instrument response function (IRF)

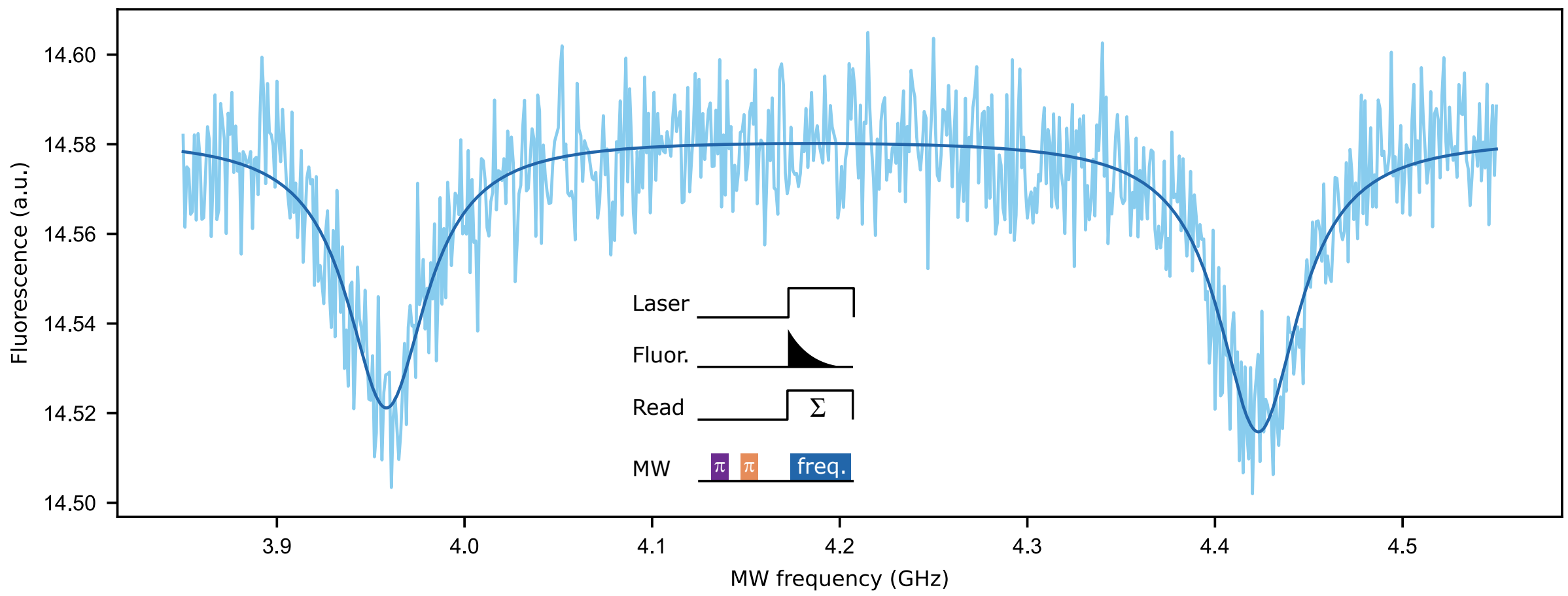

Supplementary Figure 51. Optically detected magnetic resonance spectra of the excited state. To this end we work with ensembles of molecules and apply MW π-pulses on the $T_{0z} \leftrightarrow T_{0x}$ and $T_{0z} \leftrightarrow T_{0y}$ transitions in the ground state (i.e. without laser illumination, see inset). Hence, the molecules are prepared in their bright fluorescent state for optical excitation with a laser around 0 GHz (c.f. Supplementary Fig. 46). During this laser pulse the molecules transition either $T_{0x} \rightarrow T_{1x'}$ or $T_{0y} \rightarrow T_{1y'}$. In addition an MW pulse with variable frequency is applied during the laser pulse. The latter pulse affects the electron spin in the excited triplet state. If on resonance the MW pulse flips the excited state spin from either $T_{1x'}$ or $T_{1y'}$ into $T_{1z'}$, which then more quickly decays to the ground state T_{0z} yielding less fluorescence. The spectrum shows the two resonances $T_{1y'} \leftrightarrow T_{1z'}$ (3.958(1) GHz) and $T_{1x'} \leftrightarrow T_{1z'}$ (4.423(1) GHz) as exactly such dips in fluorescence. The latter assignment is performed by taking into account the optical transition frequencies corresponding to T_{0y} to $T_{1y'}$ and T_{0x} to $T_{1x'}$.

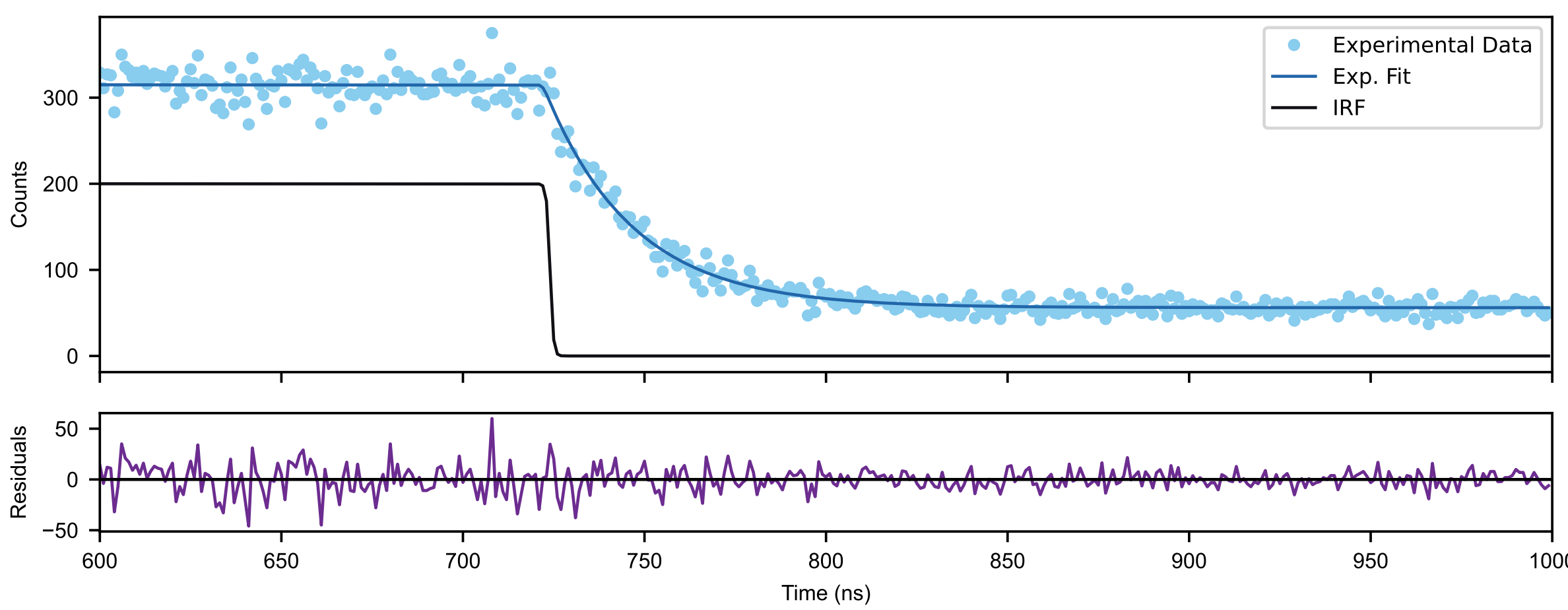

Supplementary Figure 52. Single molecule excited state lifetime measurement. (Upper graph) The fluorescence response of a single molecule excited on its $T_{0y} \leftrightarrow T_{1y'}$ is shown for when the laser is switched off (around 720 ns). The instrument response function (IRF) to the laser pulse's falling edge is vertically scaled and plotted for comparison. A single exponential decay convolved with IRF is fit to the data. (Lower graph) Residuals of fit to data.

to the falling edge of the laser pulse we have recorded the bare laser light on the single photon detector (see IRF in Supplementary Fig. 52). The fit to the molecule fluorescence response is a convolution of a single exponential decay with the IRF. It yields an excited state lifetime of

$$\tau_{\mathrm{exc}} = 24(2)\,\mathrm{ns}. \tag{15}$$

The lifetime of the $T_{1z'}$ of a single molecule could not be determined because we did not find a single molecule $T_{0z} \leftrightarrow T_{1z'}$ transition in excitation spectroscopy. The lifetime of the $T_{1y'}$ state, however, is comparable to the long lifetime component in the ensemble measurement results presented in Supplementary Fig. 19. In addition, the state $T_{1x'}$ is similarily longlived as $T_{1y'}$. We, therefore, expect that the lifetime of $T_{1z'}$ is similar to the short lifetime presented in Supplementary Figure 19, i.e. ≈ 5 ns.

The excited state lifetimes give some insight to the ISC rates for different spin states. If we separate the excited state decay rates $\gamma_{x'/y'/z'}$ into pure ISC $\gamma_{ISC,x'/y'/z'}$, radiative decay γ_r and non-radiative decay γ_{nr} we get

$$\gamma_{x'/y'/z'} = \gamma_r + \gamma_{nr} + \gamma_{ISC,x'/y'/z'} \,. \tag{16}$$

And if we assume $\gamma_{x'} \approx \gamma_{y'}$, we arrive at

$$\begin{aligned}\gamma_r + \gamma_{nr} + \gamma_{ISC,z'} &\approx \frac{24\,\text{ns}}{5\,\text{ns}} \left(\gamma_r + \gamma_{nr} + \gamma_{ISC,x'/y'}\right) \\ &\approx 5\left(\gamma_r + \gamma_{nr} + \gamma_{ISC,x'/y'}\right) \\ \gamma_{ISC,z'} &\approx 4\gamma_r + 4\gamma_{nr} + 5\gamma_{ISC,x'/y'} \\ \gamma_{ISC,z'} &\gtrsim 5\gamma_{ISC,x'/y'} \,.\end{aligned} \tag{17}$$

Hence, the ISC rate $\gamma_{ISC,z'}$ from $T_{1z'}$ into the singlet manifold is likely at least five times larger than those ($\gamma_{ISC,x'/y'}$) from $T_{1x'}$ and $T_{1y'}$ into the singlet manifold. Depending on the unknown values for γ_r and γ_{nr} this ratio might be much higher. Conversely, the probability to emit fluorescence from the three triplet excited states relate to each other like

$$\begin{aligned}p_{F,x'/y'} &= \frac{\gamma_r}{\gamma_r + \gamma_{nr} + \gamma_{ISC,x'/y'}} \approx 24\,\text{ns} \cdot \gamma_r \\ p_{F,z'} &= \frac{\gamma_r}{\gamma_r + \gamma_{nr} + \gamma_{ISC,z'}} \approx 5\,\text{ns} \cdot \gamma_r \\ p_{F,x'/y'} &\approx 5 \cdot p_{F,z'} \,.\end{aligned} \tag{18}$$

Hence, $T_{1z'}$ is expected to be five times dimmer than the other two states.
While excited state lifetimes give some insight into ISC from excited to metastable singlet states, the pulsed measurements in Figure 4 of the main paper give some hint about the total ISC rate from the singlet manifold to the ground state triplet states. Namely, coherent spin manipulation between two laser pulses can be performed for short gaps down to $1\,\mu$s (also for short laser pulses of $\sim 1\,\mu$s). Therefore, we suppose a singlet lifetime below $1\,\mu$s. The simulation in SI Sec. VII F yield values down to 100 ns.

M. On the prospect of single-shot readout

A benchmark experiments for any qubits is its capability to be read out in a single shot. This experiment is usually more nuanced. For example, depending on the readout mechanism and the qubit type, the measurement might yield a high-fidelity readout result, but not preserve the qubit in the measured state afterwards. In our case, the possibility for single-shot readout is currently limited mostly by two factors. First, a high branching ration of the excited triplet to the singlet state (and thus rather low cyclicity of the optical transition) limits the number of optical excitations until the original spin state is lost. For an estimation of the ISC rates, see SI Sec. VII F. This effect is amplified by a quantum efficiency of the triplet to triplet deexcitation of 11.9%. Second, the experimental setup currently provides an end-to-end photon detection efficiency of 1.3% (see Sec. VII A 4). Taken together, the resulting measurements of the spin state can be seen in Fig. 4d in the main text.
Further analysis of the measurement can be seen in Supplementary Fig. 53. Here, the cumulated number of photons for the readout of both spin states is plotted over the length of the readout pulse, as well as the resulting signal-to-noise ratio (SNR) assuming photon shot noise

$$N = \sqrt{n_{T_{0y}} + n_{T_{0z}}}, \tag{19}$$

with the number of cumulatively detected photons $n_{T_{0y}}$ and $n_{T_{0z}}$ for both spin states. The highest SNR of $\text{SNR}_{max} = 0.0747$ can be reached using a readout window of 5.43μs. When assessing the upper physical boundaries of the molecule with respect to single-shot spin readout, one might assume a photon detection efficiency of 100%, in contrast to the estimation of 1.3% (see Sec. VII A 4) for our experimental setup. This would increase the detected fluorescence by a factor of 76.9, and the SNR by a factor of $\sqrt{76.9} \cong 8.8$. This means that, even for perfect photon detection efficiency, the SNR of the readout is $\text{SNR}_{\text{max, perfect}} = 0.66$, and thus not yet in the regime for direct single-shot readout.
The evaluated relative contrast of the spin readout reaches $\sim 95\%$ for short durations of the readout. The smallest separation between spin-selective optical transitions is the single-molecule XX'–YY' splitting of $\Delta f_{XY} \approx 1.56\,\text{GHz}$,

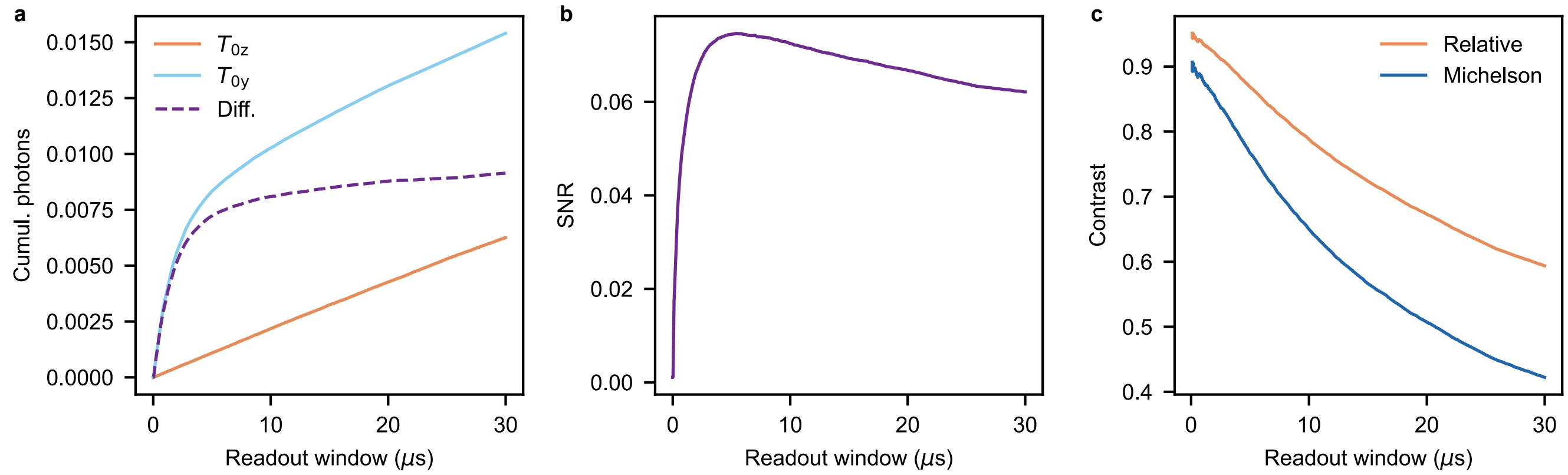

Supplementary Figure 53. Further analysis of the optical spin state readout from Fig. 4d in the main text. **a** shows the cumulative number of detected photons per readout pulse, and the absolute contrast. **b** shows the signal-to-noise ratio of the readout, assuming photon shot noise as the dominant noise source. **c** shows the relative and the Michelson contrast for different readout windows.

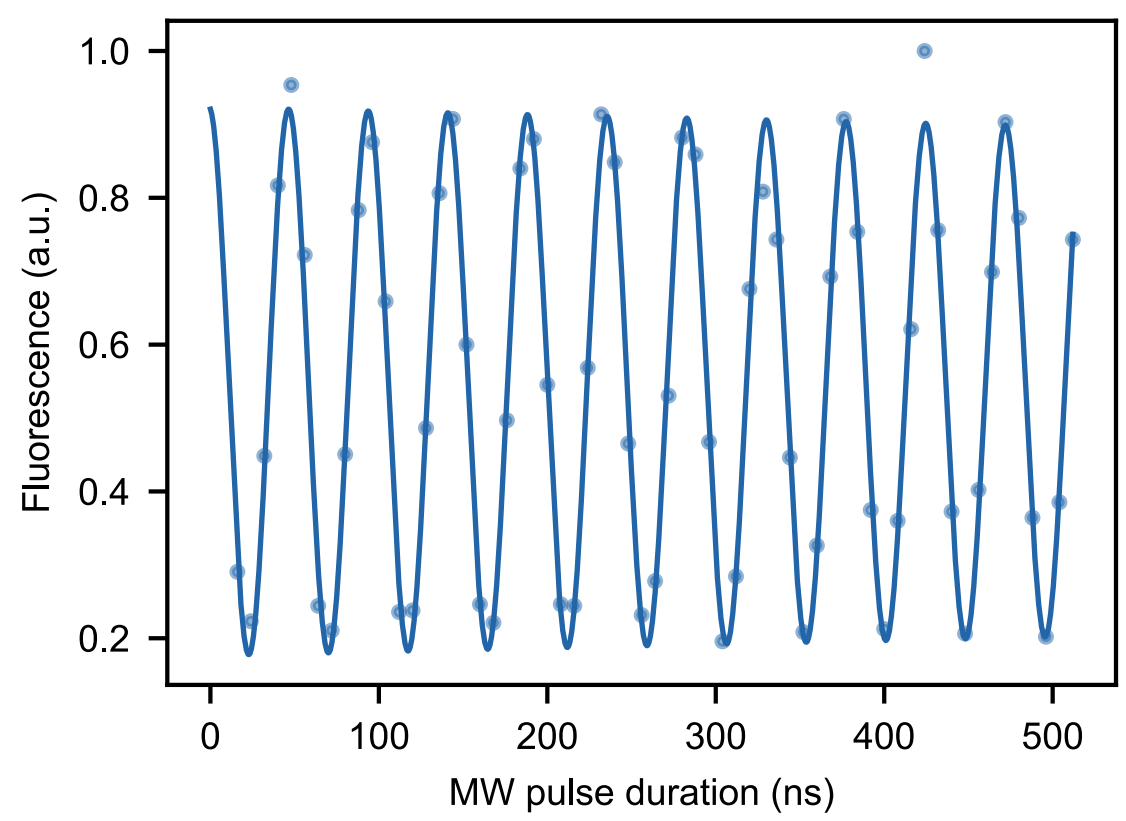

Supplementary Figure 54. Rabi oscillations between the T_{0x} and T_{0y} state at a transition frequency of 1.08 GHz. The fitted Rabi frequency is 21.2 MHz. The measurement is performed by optical initialization of the spin state to T_{0z}, a subsequent π-pulse on the ZY transition, a MW pulse with varying duration, and subsequent laser excitation of the YY' transition.

while the measured single-molecule optical linewidth is 38(2) MHz. Assuming a Lorentzian line shape, this corresponds to an off-resonant excitation probability of approximately $\epsilon_{\text{off}} = 1/[1 + (2\Delta f/\Gamma)^2] \simeq 1.5 \times 10^{-4}$, or a spectral excitation selectivity of about 99.985%. The separation from the ZZ' transition is even larger: from the experimentally determined level structure we estimate $\Delta f_{ZY} = 14.58$ GHz and $\Delta f_{XZ} = 16.12$ GHz, corresponding to off-resonant excitation probabilities below 2×10^{-6}. Thus, the measured readout contrast is limited by background fluorescence, dark counts and non-perfect spin initialization, and not by optical leakage caused by spectral overlap between the spin-selective optical transitions.

1. Repetitive readout of a subspace of the triplet spin

One property of the molecule prohibiting direct single-shot readout of the electron spin state is the fast depopulation of T_{0x} or T_{0y} to the T_{0z} state via ISC. However, when e.g. the T_{0y} state is optically excited, the ISC is highly selective to the T_{0z} state, and only weakly populates the T_{0x} state (see SI Sec. IV F 8). While not giving the possibility for a quantum non-demolition measurement, this allows for a repetitive readout discriminating two subspaces of the ground state triplet spin, namely the T_{0y}-T_{0z}-subspace from the T_{0x}-subspace or discriminating the T_{0x}-T_{0z}-subspace from the T_{0y}-subspace, depending on the laser and microwaves applied.

The pulse scheme for the repetitive readout is shown in Supplementary Fig. 55a. By applying laser excitation resonant to the YY' transition, simultaneously applying a MW drive to the XY transition, the system is initialized in the T_{0z} state (for a demonstration of Rabi oscillations on the XY transition, see Supplementary Fig. 54. Afterwards, a π-pulse

on either the ZX or ZY transition initializes the system to the T_{0x} or T_{0y} state, respectively. In order to distinguish between the two states, resonant laser excitation on the YY' transition is performed, while continuously applying MW π-pulses on the ZY transition (while a continuous MW drive would work as well, we opted for fast distinct π-pulses to retain low spectral selectivity while reducing heat load on the molecular crystal). For the case of initialization in T_{0y} this results in prolonged fluorescence, by recovering the optically populated T_{0z} state to T_{0y}. When initialized to the T_{0x} state, there is no effect due to optical excitation or MW manipulation. The resulting fluorescence signals

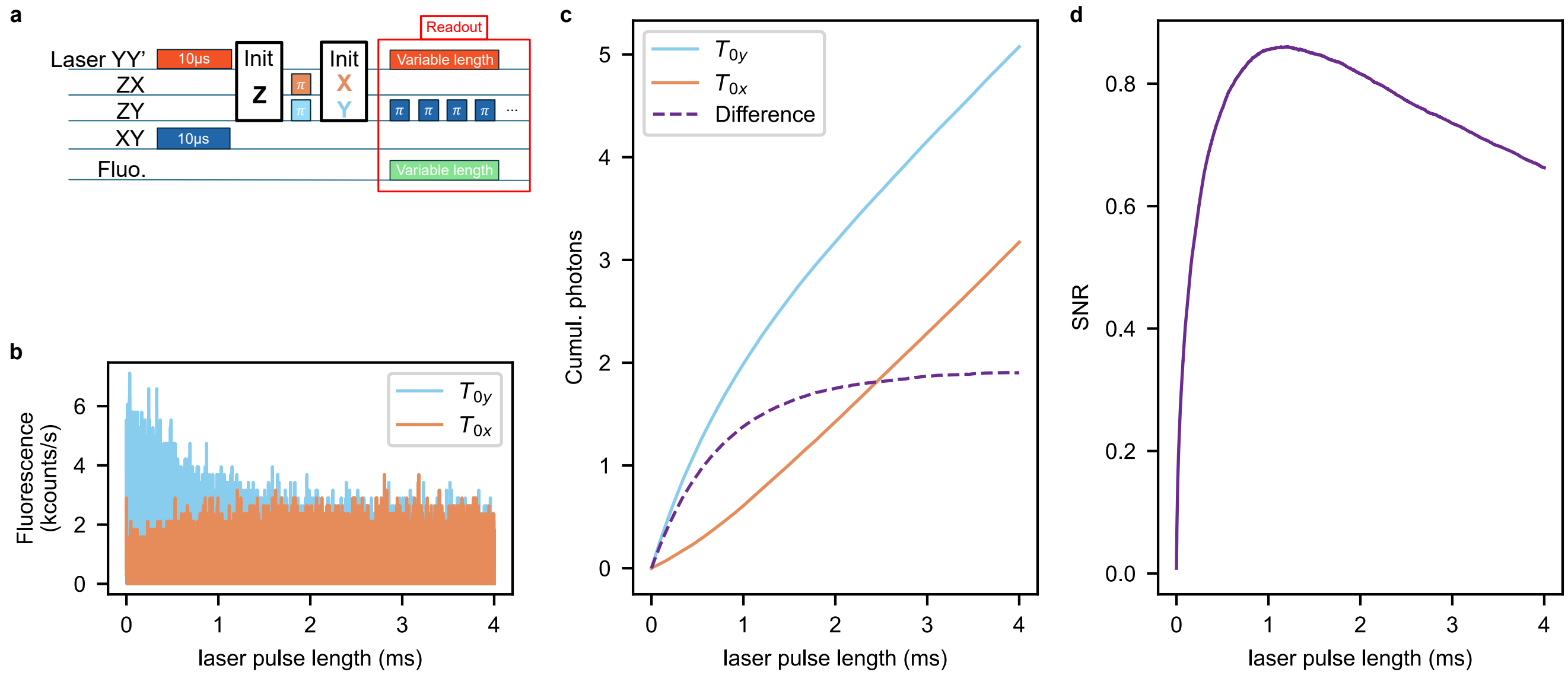

Supplementary Figure 55. Results for repetitive readout of a subspace of the triplet electron spin. **a** shows the pulse scheme for repetitive readout. The triplet spin state is first initialized to the T_{0x} or T_{0y} state. This is achieved by spin pumping to the T_{0z} state, and subsequent application of an ZX or ZY π-pulse. For readout, the molecule is constantly excited on the YY' transition, while applying MW π-pulses on the ZY transition.**b** shows fluorescence timetraces for repetitive readout of the two prepared states T_{0x} and T_{0y}. **c** shows the number of cumulative number of photons for both initialized states, and their difference. **d** shows the SNR of the readout with varying laser pulse length. A maximum SNR of $\mathrm{SNR}_{\mathrm{max,rep}} = 0.86$ is reached for a laser pulse length of 1.2 ms.

are shown in Supplementary Fig. 55b. The signal difference persists for a few milliseconds, which shows, that the initial population difference between the T_{0x} and T_{0y} state can effectively be preserved over multiple excitation and ISC cycles (~ 1000, compare z-selectivity of ground state ISC in SI Sec. IV F 8). Similar to Supplementary Fig. 53, Supplementary Fig. 55c,d show the cumulative number of detected photons for both initialized states, and the resulting SNR of the readout assuming photon shot noise (see above). Remarkably, $\mathrm{SNR}_{\mathrm{max,rep}} = 0.86$ for a laser pulse length of 1.2 ms, almost reaching a value of 1 and thus the regime of a single shot readout for discriminating the T_{0x}-subspace from the T_{0z}-T_{0y} subspace.

VIII. COMPARISON OF SPIN-PHOTON INTERFACES

To systematically assess the performance of the presented qubit molecule against established solid-state qubits, we identify several key figures of merit that dictate their viability as a spin-photon interface. With one exception, we opted to quote numbers that present values of the emitters in bulk materials, without any improvements that can be achieved for example via coupling to optical cavities. The values without an added reference are taken from Atatüre et al..[45] We opted to show five properties overall. The measured optical linewidth gives an insight into residual dephasing components of the optical line. Since slow fluctuations or spectral jumps, e.g. as for NV centers,[46] have the potential to be actively controlled, we do not mention it here. The inverse lifetime, in conjunction with the measured optical linewidth, shows the amplitude of a residual dephasing component. In addition, it gives an estimate of the achievable photon emission rate of the emitter. The radiative emission into the ZPL is the product of PLQY and the Debye-Waller factor, i.e. the amount of photons that are emitted without phononic contribution per excitation. In order to condense the spin properties to one quantity, we chose the T_2 coherence time. The results can be seen in Supplementary Table 13.

Spin-Photon interface	wavelength (nm)	measured optical linewidth (MHz)	transform limited linewidth (MHz)	Radiative emission into ZPL (%)	Spin T_2 (ms)	Temperature (K)
This work	595	38	6.6	2.1	2.2	4.5
Diamond NV	637	14	13	2.4	1 600 [47]	3.7
Diamond SiV	737	100	86	7	13 [48]	0.1
Diamond GeV	602	42	28	35	18 [49]	0.2
SiC Di-Vacancy	1100	80	11	$\ll 7$ [a]	5 000 [50]	5
SiC V_{Si}	900	20	17	2.6 [51,52]	20	5
$^{171}Yb^{3+}$ in YVO [53b]	984	$\lesssim 1.4$	0.07	$\lesssim 100$	30	0.04
Silicon T-center	1326	33 [54]	0.169 [54]	4.2 [55,56c]	2.1 [55]	1.4

[a] PLQY not known, 7% Debye-Waller-Factor.
[b] Measured in photonic crystal cavity.
[c] For deuterated T center the emission percentage to the ZPL is 23%, albeit with 5 times slower emission.

Supplementary Table 13. Comparison of figures of merit for different spin-photon interfaces.

[1] Kalomenopoulos, P. G., Emayavaramban, B. & Johnston, C. P. Enantioselective Synthesis of α-Aryl Ketones by a Cobalt-Catalyzed Semipinacol Rearrangement. *Angewandte Chemie International Edition* **64**, e202414342 (2025).

[2] Schmitt, E. *et al.* A General Approach towards NH-Pyrazoles That Bear Diverse Fluoroalkyl Groups by Means of Fluorinated Iminium Salts. *European Journal of Organic Chemistry* **2015**, 6052–6060 (2015).

[3] Roggors, S. *et al.* Optically Detected Magnetic Resonance on Carbene Molecular Qubits. *Journal of the American Chemical Society* **147**, 36383–36392 (2025).

[4] Groom, C. R., Bruno, I. J., Lightfoot, M. P. & Ward, S. C. The Cambridge Structural Database. *Acta Crystallographica Section B* **72**, 171–179 (2016).

[5] Kratzert, D. FinalCif.

[6] *Crysalispro* **1.171.44.104a** (2025).

[7] Sheldrick, G. M. Crystal structure refinement with *SHELXL*. *Acta Crystallographica Section C* **71**, 3–8 (2015).

[8] Sheldrick, G. M. *SHELXT* – Integrated space-group and crystal-structure determination. *Acta Crystallographica Section A* **71**, 3–8 (2015).

[9] Kleemiss, F. *et al.* Accurate crystal structures and chemical properties from NoSpherA2. *Chemical Science* **12**, 1675–1692 (2021).

[10] Dolomanov, O. V., Bourhis, L. J., Gildea, R. J., Howard, J. A. K. & Puschmann, H. *OLEX2* : A complete structure solution, refinement and analysis program. *Journal of Applied Crystallography* **42**, 339–341 (2009).

[11] Charbonneau, G. P. & Delugeard, Y. Biphenyl: Three-dimensional data and new refinement at 293 K. *Acta Crystallographica Section B Structural Crystallography and Crystal Chemistry* **33**, 1586–1588 (1977).

[12] Spackman, P. R. *et al.* *CrystalExplorer* : A program for Hirshfeld surface analysis, visualization and quantitative analysis of molecular crystals. *Journal of Applied Crystallography* **54**, 1006–1011 (2021).

[13] Karpiuk, J., Majka, A., Karolak, E. & Nowacki, J. Intramolecular Electron Transfer in Frozen Solvents: Charge Transfer and Local Triplet States Population Dynamics Revealed by Dual Phosphorescence. *The Journal of Physical Chemistry Letters* **8**, 4659–4667 (2017).

[14] Kim, H. S., Park, S.-R. & Suh, M. C. Concentration Quenching Behavior of Thermally Activated Delayed Fluorescence in a Solid Film. *The Journal of Physical Chemistry C* **121**, 13986–13997 (2017).

[15] Liu, C.-H., Hamzehpoor, E., Sakai-Otsuka, Y., Jadhav, T. & Perepichka, D. F. A Pure-Red Doublet Emission with 90 % Quantum Yield: Stable, Colorless, Iodinated Triphenylmethane Solid. *Angewandte Chemie International Edition* **59**, 23030–23034 (2020).

[16] Shirazi, R. G., Pantazis, D. A. & Neese, F. Performance of density functional theory and orbital-optimised second-order perturbation theory methods for geometries and singlet–triplet state splittings of aryl-carbenes. *Molecular Physics* **118**, e1764644 (2020).

[17] Kawano, M., Hirai, K., Tomioka, H. & Ohashi, Y. Structure Determination of Triplet Diphenylcarbenes by in Situ X-ray Crystallographic Analysis. *Journal of the American Chemical Society* **129**, 2383–2391 (2007).

[18] Piteša, T., Alešković, M., Becker, K., Basarić, N. & Došlić, N. Photoelimination of Nitrogen from Diazoalkanes: Involvement of Higher Excited Singlet States in the Carbene Formation. *Journal of the American Chemical Society* **142**, 9718–9724 (2020).

[19] Bannwarth, C., Ehlert, S. & Grimme, S. GFN2-xTB—An Accurate and Broadly Parametrized Self-Consistent Tight-Binding Quantum Chemical Method with Multipole Electrostatics and Density-Dependent Dispersion Contributions. *Journal of Chemical Theory and Computation* **15**, 1652–1671 (2019).

[20] Poh, Y. R. *et al.* Alternant Hydrocarbon Diradicals as Optically Addressable Molecular Qubits. *Journal of the American Chemical Society* **146**, 15549–15561 (2024).

[21] Zhurko, G. A. Chemcraft - graphical program for visualization of quantum chemistry computations (Version 1.8) (2005).

[22] Poole, C. P., Jr., Farach, H. A. & Jackson, W. K. Standardization of convention for zero field splitting parameters. *The Journal of Chemical Physics* **61**, 2220–2221 (1974).
[23] Weil, J. A. & Bolton, J. R. *Electron Paramagnetic Resonance: Elementary Theory and Practical Applications* (Wiley, 2006), 1 edn.
[24] Marian, C. M. Understanding and Controlling Intersystem Crossing in Molecules. *Annual Review of Physical Chemistry* **72**, 617–640 (2021).
[25] Poh, Y. R., Chen, X., Cheng, H.-P. & Yuen-Zhou, J. Quantifying the Spin-Optical Properties of Bent π-Carbene Molecular Qubits. (2026). ChemRxiv.10002007 (Preprint).
[26] Poh, Y. R., Chen, X., Cheng, H.-P. & Yuen-Zhou, J. Electron Sextets as Optically Addressable Molecular Qubits: Triplet Carbenes. *Journal of the American Chemical Society* **147**, 22529–22541 (2025).
[27] Stoiber, R. E. & Morse, S. A. *The Immersion Method*, 49–75 (Springer US, Boston, MA, 1994).
[28] Van Horn, B. L. & Winter, H. H. Conoscopic Measurement of Birefringence and Orientation in Biaxially Stretched Polymer Films and Sheets. *Macromolecules* **36**, 8513–8521 (2003).
[29] Sørensen, B. E. A revised Michel-Lévy interference colour chart based on first-principles calculations. *European Journal of Mineralogy* **25**, 5–10 (2013).
[30] Stoll, S. & Schweiger, A. EasySpin, a comprehensive software package for spectral simulation and analysis in EPR. *Journal of Magnetic Resonance* **178**, 42–55 (2006).
[31] Anderson, R. J. M. & Kohler, B. E. Electron nuclear double resonance of triplet diphenylmethylene in *P* 21 benzophenone: Conformation and spin distribution. *The Journal of Chemical Physics* **65**, 2451–2459 (1976).
[32] Graham, D. J. & Wang, C.-L. A magnetic field study of excited triplet diphenylmethylene. *The Journal of Chemical Physics* **85**, 4441–4445 (1986).
[33] Haider, K. W., Platz, M. S., Despres, Alain., Lejeune, Violaine. & Migirdicyan, Eva. Triplet-triplet fluorescence and spin polarization: Diphenylcarbene and dibenzocycloheptadienylidene. *The Journal of Physical Chemistry* **94**, 142–147 (1990).
[34] Wasserman, E. *et al.* Electron paramagnetic resonance of 9,9'-dianthrylmethylene. Linear aromatic ground-state triplet methylene. *Journal of the American Chemical Society* **93**, 6335–6337 (1971).
[35] Graham, D. J. & Lin, T.-S. High-Resolution Absorption Spectra of Diphenylmethylenes. *Chemical Physics* 411–420 (1982).
[36] Kozankiewicz, B., Gudmundsdottir, A., Orrit, M., Platz, M. & Tamarat, Ph. Hole burning on a triplet–triplet transition: 2,2-dinaphthylcarbene in n-hexane at 1.7K. *Journal of Luminescence* **86**, 261–263 (2000).
[37] Kozankiewicz, B. *et al.* A Hole-Burning Study of the Zero-Field Splitting of the Triplet Ground and Excited States of 2,2-Dinaphthylcarbene in n-Heptane and n-Hexane at 1.7 K. *The Journal of Physical Chemistry A* **104**, 7464–7468 (2000).
[38] Kozankiewicz, B. *et al.* Zero-Field Splitting of the Electronic Ground and Lowest Excited Triplet States of 2,2-Dinaphthylcarbene in n -Hexane at 1.7 K. *The Journal of Physical Chemistry A* **103**, 3155–3162 (1999).
[39] Brusar, V. *et al.* Ultrafast Photoelimination of Nitrogen from Upper Excited States of Diazoalkanes and the Fate of Carbenes Formed in the Reaction. *The Journal of Organic Chemistry* **88**, 4286–4300 (2023).
[40] Lambert, N. *et al.* QuTiP 5: The Quantum Toolbox in Python. *Physics Reports* **1153**, 1–62 (2026).
[41] Kummer, S. *et al.* Absorption, excitation, and emission spectroscopy of terrylene in p-terphenyl: Bulk measurements and single molecule studies. *The Journal of Chemical Physics* **107**, 7673–7684 (1997).
[42] Basché, Th., Kummer, S. & Bräuchle, C. Optical spectroscopy of single pentacene molecules in an ordered and a disordered p-terphenyl crystal. *Chemical Physics Letters* **225**, 116–123 (1994).
[43] Brouwer, A. C. J., Köhler, J., Groenen, E. J. J. & Schmidt, J. 13C isotope effects for pentacene in p -terphenyl: High-resolution spectroscopy and single-spin detection. *The Journal of Chemical Physics* **105**, 2212–2222 (1996).
[44] Doberer, U., Port, H., Rund, D. & Tuffentsammer, W. 13C isotope shifts of anthracene and naphthalene isomers in triplet state optical spectra. *Molecular Physics* **49**, 1167–1178 (1983).
[45] Atatüre, M., Englund, D., Vamivakas, N., Lee, S.-Y. & Wrachtrup, J. Material platforms for spin-based photonic quantum technologies. *Nature Reviews Materials* **3**, 38–51 (2018).
[46] Fu, K.-M. C. *et al.* Observation of the Dynamic Jahn-Teller Effect in the Excited States of Nitrogen-Vacancy Centers in Diamond. *Physical Review Letters* **103**, 256404 (2009).
[47] Abobeih, M. H. *et al.* One-second coherence for a single electron spin coupled to a multi-qubit nuclear-spin environment. *Nature Communications* **9**, 2552 (2018).
[48] Sukachev, D. D. *et al.* Silicon-Vacancy Spin Qubit in Diamond: A Quantum Memory Exceeding 10 ms with Single-Shot State Readout. *Physical Review Letters* **119**, 223602 (2017).
[49] Senkalla, K., Genov, G., Metsch, M. H., Siyushev, P. & Jelezko, F. Germanium Vacancy in Diamond Quantum Memory Exceeding 20 ms. *Physical Review Letters* **132**, 026901 (2024).
[50] Anderson, C. P. *et al.* Five-second coherence of a single spin with single-shot readout in silicon carbide. *Science Advances* **8**, eabm5912 (2022).
[51] Son, N. T. *et al.* Developing silicon carbide for quantum spintronics. *Applied Physics Letters* **116**, 190501 (2020).
[52] Morioka, N. *et al.* Spin-Optical Dynamics and Quantum Efficiency of a Single V1 Center in Silicon Carbide. *Physical Review Applied* **17**, 054005 (2022).
[53] Kindem, J. M. *et al.* Control and single-shot readout of an ion embedded in a nanophotonic cavity. *Nature* **580**, 201–204 (2020).
[54] Higginbottom, D. B. *et al.* Optical observation of single spins in silicon. *Nature* **607**, 266–270 (2022).
[55] Bergeron, L. *et al.* Silicon-Integrated Telecommunications Photon-Spin Interface. *PRX Quantum* **1**, 020301 (2020).
[56] Kazemi, M. *et al.* Giant Isotope Effect on the Excited-State Lifetime and Emission Efficiency of the Silicon T Center. *Physical Review Letters* **136**, 053602 (2026).
