# Supplementary material for: A single-molecule spin–photon interface
Source: Nature. 2026 Oct 28;658(8138):1153–9. doi: 10.1038/s41586-026-11147-6 (PMC13716814; doi:10.1038/s41586-026-11147-6)
Supplement: Supplementary file 2 [file 41586_2026_11147_MOESM2_ESM.pdf]

nature portfolio

Peer Review File

A Single-Molecule Spin-Photon Interface

Corresponding Author: Dr Matthias Pfender

Any redactions in this file are there to maintain patient confidentiality, the confidentiality of unpublished data, or to remove third-party material.

This file contains all reviewer reports in order by version, followed by all author rebuttals in order by version.

Attachments originally included by the reviewers as part of their assessment can be found at the end of this file.

Version 1:

Reviewer comments:

Referee #1

(Remarks to the Author)
In this article, the authors introduce a novel molecular system in which single electron spins can be optically read out. It consists of a carbene molecule which is first doped and then photo-activated into a host crystal made from similar molecular species. The energy level structure is theoretically evaluated by quantum chemistry methods and reveals triplet states in the ground and excited levels, spin selective optical transitions, and a spin polarization mechanism through intersystem crossing. These highly uncommon properties for molecules are indeed akin to some of the most promising color centers in diamond.
These predictions are nicely confirmed by low temperature ensemble spectroscopy and then used to measure important parameters on single molecules. First, the optical transitions are remarkably narrow and stable in frequency, which is important for applications in quantum networks. Second, single spin coherent excitation and manipulation, followed by optical read out, show a Hahn T2 of about 10 µs, which can be extended to 20 ms by qubit-relevant dynamical decoupling. This is a value similar to those reported for other solid-state quantum emitters, although the spin T1 is only 10 ms.

This is a well-written paper with convincing and novel results which represent a major milestone for molecular qubits since it is the first demonstration of a single optically addressable spin in this platform. However, the more general question as to whether this is also an important result for solid state quantum emitters should, in my view, be more clearly demonstrated. Indeed, although molecular systems are often rightly advertised as highly tunable, with atomic scale accuracy, integrable in photonic structures, etc.., it does not mean that properties obtained for a given molecule can be improved in order to reach the required level for quantum applications. I suggest the authors consider the comments below and submit a revised version of their manuscript.

Major concerns:
As mentioned above, my main concerns are related to the potential of the system for more advanced demonstrations and possible applications:
- the emission wavelength is in the visible range and mitigation strategies should be mentioned in relation to quantum networks. Could it be possible to design molecules with IR of near-IR emissions?
- T1 appears to be rather short and sets up a strong limitation on achievable T2. What is limiting T1 and could it be extended at e.g. lower temperatures? Although a 21 ms T2 is obtained with dynamical decoupling, the Hahn echo is only 12 µs. This may have important consequences on storage fidelity at long times and should be discussed.
- The qubit is not stable at room temperature but is there evidence that at the operating temperature its stability can extend over e.g. days or longer? It is also not completely clear to me what is creation yield of the qubits - can deterministic creation be envisioned for e.g. integration into photonic structures?
- The emission quantum yield is low. Can this be improved as it could also make potential applications much more difficult to achieve. In particular, taking into consideration the other properties of the qubit (cyclicity, spin T1, etc..), is single-shot spin readout feasible?

Minor comments
- introduction is especially short and should give more context to the study. For example, what are the key

parameters/experiments to consider and how do they look like in competing systems?
- p. 5: what is causing the additional decoherence of the optical transition? The claim 'exceptional stability' should be supported by a comparison to e.g. color centers in diamond. What is the origin of the optical stability?
- Fig. 4d - the meaning of the y axis label is not clear to me. How many repetitions of the sequence b are performed to yield Fig. 2c?
- p. 8: 'more than an order of magnitude': maybe add Vasilenko et al., Nat. Materials, 2026 which reports T2 DD = 2 ms and T2 Hahn = 0.6 ms for an ensemble of nuclear spins.
- p. 8 'significantly lower noise' - In this case why is the Hahn echo T2 much shorter than e.g. for the NV centers?

Referee #2

(Remarks to the Author)
The authors demonstrate optical spin detection and coherent manipulation of an individual ground-state molecular spin for the first time. This presents an important advance for the application of molecular qubits. The demonstrated combination of properties (~38 MHz optical linewidths, ~24 ns optical lifetimes, long-term spectral stability, and ~ms coherence under dynamical decoupling) are attractive, even when compared to much more established solid-state defect systems. The paper is very clearly written, well presented, and the work is thorough. The detailed methods and supporting information are valuable. I believe the importance of this demonstration, combined with the quality of the manuscript warrant publication in Nature. I do have some comments which the authors should address before publication, however.

In the abstract, the authors state “however, the lack of a robust spin-photon interface combining bright fluorescence, high spectral stability, and the persistent spin lifetimes inherent to ground-state systems has prohibited the detection of individual molecular qubits” They should rephrase this statement as it sounds like there has never been a detection of an individual molecular spin, and that all detection methods for an individual molecular qubit would require all of the above features (which is not the case).

The paper should include citations to relevant earlier work on hole-burning spectroscopy of carbenes performed on similar compounds as these relate to the authors’ work. For example, there are several papers by Kozankiewicz et al. from the 1990s which would be relevant to cite.

To assess the brightness of this system, the authors should clarify their single-molecule saturation count rate, including a saturation curve.

The authors use photoactivation at 580 nm. However, most previous work on carbenes used UV excitation wavelengths: the authors should include a comment on the process at play and why they are able to use longer wavelengths than in previous studies.

The authors state “This level of control, achieved via confocal microscopy, allows the qubit molecule density to be regulated independently of the overall doping level.” Given the similarity between photoactivation wavelength (580nm) and resonant ZPL excitation wavelength (~595 nm), the authors should clarify to what extent this holds. In other words, to what extent does resonant ZPL excitation cause photoactivation?

The authors should clarify whether they observed any photostability issues with the samples. To what extent are the generated carbenes robust to higher laser power, for example, and do they observe stability over long (~week) timescales?

The authors should include a comment on the role of the liberated nearby nitrogen: do they expect this to be detrimental, e.g., for inducing disorder?

In Fig. 1b, the authors display a calculated transition-dipole moment which appears to have ‘chosen’ one of the two biphenyl units’ directions. It would be helpful to further clarify why this arises.

In Fig. 2b: it would be helpful to include a diagram of which transitions result in the substructure for clarity.

The authors state “we find this transition to involve a formal redistribution of spin density from the central carbene p-orbital to an adjacent phenyl ring (Fig. 1b).” however this does not seem to be clear from Fig. 1b. The authors should update the manuscript accordingly.

In Fig 1b, the authors display calculated ISC rates. However, it is only made clear later in the text how these have been determined. (A brief update to the caption would be sufficient to fix this.)

In the outlook, the authors should include a more critical discussion of the opportunities the work opens up. For example, solid-state defects can be coupled to photonic cavities so the authors should clarify the real opportunity for added-value.

The authors should clarify whether the diarylketone sublimes under vacuum at room temperature (since the sample is under vacuum at the start of the cool down).

The authors show microwave transitions involving T0z. It would be helpful to clarify if they can observe transitions between

T0x and T0y (e.g., under off-resonant excitation). This would be helpful in clarifying the selectivity between T0x and T0y from the ISC.

In the supporting information, the authors present a PLQY of 10%. They should clarify why this is relatively low and how it might be improved.

It would be valuable for the authors to present single-spin T2* ODMR data to complement the Hahn-echo/dynamical decoupling data.

There is a typo on line 81: 'qubit molecules fluoresce'

Referee #3

(Remarks to the Author)

Referee #4

(Remarks to the Author)
I co-reviewed this manuscript with one of the reviewers who provided the listed reports.

Version 2:

Reviewer comments:

Referee #1

(Remarks to the Author)
In the revised manuscript and their response letter, the authors have addressed my concerns satisfactorily. The manuscript has been substantially improved, and I am happy to recommend it for publication in Nature.

Referee #2

(Remarks to the Author)
The authors have satisfactorily addressed my comments and revised the manuscript and supplementary information appropriately. I recommend the article to be published in Nature.

Referee #3

(Remarks to the Author)
I am satisfied with the provided answers and with the additional details provided in the manuscript and SM.

Referee #4

(Remarks to the Author)
I co-reviewed this manuscript with one of the reviewers who provided the listed reports.

Open Access This Peer Review File is licensed under a Creative Commons Attribution 4.0 International License, which permits use, sharing, adaptation, distribution and reproduction in any medium or format, as long as you give appropriate credit to the original author(s) and the source, provide a link to the Creative Commons license, and indicate if changes were made.
In cases where reviewers are anonymous, credit should be given to 'Anonymous Referee' and the source.
The images or other third party material in this Peer Review File are included in the article's Creative Commons license, unless indicated otherwise in a credit line to the material. If material is not included in the article's Creative Commons license and your intended use is not permitted by statutory regulation or exceeds the permitted use, you will need to obtain permission directly from the copyright holder.
To view a copy of this license, visit https://creativecommons.org/licenses/by/4.0/

Response to Reviewers

General Response

We sincerely thank the editor and the four reviewers for their careful reading of our manuscript and for their constructive feedback. We are particularly encouraged that the reviewers recognize this work as a "major milestone for molecular qubits since it is the first demonstration of a single optically addressable spin in this platform" (Reviewer 1) which constitutes "an important advance for the application of molecular qubits" (Reviewer 2), because we "found a unique system in molecular spectroscopy" (Reviewer 3) highlighting our demonstration of optical spin detection and coherent manipulation of an individual ground-state molecular spin.

In response to the reviewers' comments, we have substantially revised the manuscript and the Supporting Information to clarify the broader impact of our work, particularly in comparison to established solid-state quantum emitters. The key improvements in this revision include:

- **Additional experimental data:** We have included several new measurements, including saturation curves, spatially-selective and wavelength-dependent activation studies, evaluation of the (repetitive) readout efficiency, chemical stability measurements and the temperature dependence of optical resonances. These experiments provide a clearer picture of the system's brightness, the activation yield, the possibility for single-shot spin readout and decoherence mechanisms.
- **Contextualizing the advantage of molecular systems:** We have expanded our discussion (particularly in the Introduction and the Outlook section) to put our findings in a broader context and to explicitly highlight the unique advantages of this molecular platform over traditional semiconductor defects, specifically detailing its potential for heterogeneous integration with photonic chips without etching damage, and its favorable properties as a quantum node.
- **Improved theoretical modeling:** We have re-examined our computational models and updated our CASSCF calculations with a more physically appropriate active space. This successfully corrects the unphysical symmetry breaking in the transition dipole moment predictions and provides a much stronger theoretical foundation for our findings.

Below, we provide detailed point-by-point responses to each reviewer's comments. We believe these revisions have significantly strengthened the manuscript and addressed all concerns, making

it suitable for publication in *Nature*.

Reviewer: 1

In this article, the authors introduce a novel molecular system in which single electron spins can be optically read out. It consists of a carbene molecule which is first doped and then photo-activated into a host crystal made from similar molecular species. The energy level structure is theoretically evaluated by quantum chemistry methods and reveals triplet states in the ground and excited levels, spin selective optical transitions, and a spin polarization mechanism through intersystem crossing. These highly uncommon properties for molecules are indeed akin to some of the most promising color centers in diamond. These predictions are nicely confirmed by low temperature ensemble spectroscopy and then used to measure important parameters on single molecules. First, the optical transitions are remarkably narrow and stable in frequency, which is important for applications in quantum networks. Second, single spin coherent excitation and manipulation, followed by optical read out, show a Hahn T2 of about 10 μs, which can be extended to 20 ms by qubit-relevant dynamical decoupling. This is a value similar to those reported for other solid-state quantum emitters, although the spin T1 is only 10 ms.

This is a well-written paper with convincing and novel results which represent a major milestone for molecular qubits since it is the first demonstration of a single optically addressable spin in this platform. However, the more general question as to whether this is also an important result for solid state quantum emitters should, in my view, be more clearly demonstrated. Indeed, although molecular systems are often rightly advertised as highly tunable, with atomic scale accuracy, integrable in photonic structures, etc.., it does not mean that properties obtained for a given molecule can be improved in order to reach the required level for quantum applications. I suggest the authors consider the comments below and submit a revised version of their manuscript.

Response: We thank the reviewer for this careful and constructive review. Before turning to the individual points, we would like to address directly the central concern raised in the reviewer's overall assessment: that molecular tunability, on its own, does not guarantee that a given molecule's properties can be pushed to the level required for quantum applications. We agree with this note of caution, and in what follows, as well as in our detailed responses below, we have tried to support our view with quantitative benchmarks and with results already demonstrated in closely related molecular systems, rather than with tunability as an abstract argument.

A widely used set of benchmarks for a spin-photon interface intended as a node in a quantum network includes: a spin coherence time sufficient to store quantum information between entanglement attempts, a high probability of emission into the zero-phonon line together with high quantum yield, near-lifetime-limited and spectrally stable optical transitions to enable photon indistinguishability between remote nodes, and spin-selective optical transitions for high-fidelity spin-photon entanglement (Awschalom, D. D. et al. *Nat. Photonics* **2018**, *12*, 516). Measured against these criteria, the carbene qubit reported here already combines several of the required properties in a single system: hour-scale optical spectral stability of 2.4-2.6 MHz, millisecond-scale spin coherence under dynamical decoupling, and spin-selective optical transitions separated by more than 1.5 GHz. The two properties furthest from the values ultimately needed are the

quantum yield (9%) and further improvements of the T_1 relaxation time (21 ms) and T_2^{Hahn}. The reviewer is right to focus on these, and we address both in detail in the responses to the specific concerns.

Our confidence that these properties can be substantially improved rests on concrete precedent within organic molecular qubits, not on the general premise that molecules are tunable. Isotopic engineering (deuteration) has already been shown to extend Hahn-echo coherence times by more than an order of magnitude in a closely related carbene system (Roggors, S. et al. *J. Am. Chem. Soc.*, **2025**, *147*, 36383–36392), where we have additionally shown that temperature reduction can lead to a large increase in T_1 relaxation time. Regarding PLQY, comparable chemical substitution strategies have raised photoluminescence quantum yields from below 15% to above 90% in related molecular radical qubits, as detailed below.

The reviewer also rightly emphasizes the importance of photonic integration. Here we see the molecular platform's principal advantage over semiconductor defects: because the doped molecular crystal can be processed into thin films and deposited onto pre-fabricated photonic circuits, emitter performance is decoupled from the etching and nanofabrication steps that typically degrade the optical stability of solid-state defects (Lombardi, P. et al. *ACS Photonics* **2018**, *5*, 126; Huang, T. et al. *Nat. Nanotechnol.* **2025**, *20*, 1748; Lange, C. M. et al. *ACS Nano* **2026**, *20*, 9867). Separately, as discussed below, coupling to periodically poled lithium niobate (PPLN) waveguides offers a near-term route to convert the visible emission to telecom wavelengths, following a strategy already demonstrated for a solid-state color center (Knaut, C. M. et al. *Nature* **2024**, *629*, 573), extending the reach of this platform toward long-distance quantum communication.

We therefore see the remaining path toward a competitive quantum-network node as primarily one of systematic molecular optimization and photonic integration engineering, building on demonstrated chemistry, rather than an open question of principle. We have in addition revised the Outlook section to provide a more refined discussion of this point. We hope the detailed, quantitative responses below substantiate this view to the reviewer's satisfaction.

Major concerns: As mentioned above, my main concerns are related to the potential of the system for more advanced demonstrations and possible applications:

- The emission wavelength is in the visible range and mitigation strategies should be mentioned in relation to quantum networks. Could it be possible to design molecules with IR of near-IR emissions?

  Response: We thank the reviewer for raising this important point. The present carbene emits in the visible, with ZPLs at 594.18 and 595.04 nm. We agree that direct transmission at this wavelength is not optimal for long-distance fibre-based quantum networking which ideally requires operating at the O-band (around 1350 nm) and the C-band (around 1550 nm). We therefore now distinguish two complementary strategies in the revised Outlook.

  First, the molecular platform provides a chemical route to shift the optical transition. In preliminary work on related carbene derivatives, we have synthesized a library of more than 40 compounds and observed that extended conjugation and electron-accepting substituents

can shift the ZPL into the far-red, up to approximately 720 nm. These results support the feasibility of further red-shifting toward the near-IR. At the same time, we need to be cautious: shifting the direct emission all the way to the primary telecom bands is unlikely to be the optimal near-term route, because large bathochromic shifts can increase charge-transfer character and non-radiative decay, potentially compromising PLQY, ZPL branching, spectral stability and spin-selective optical cycling. We therefore view chemical design as a route toward far-red/near-IR operation, but not as the sole solution for telecom-band quantum networking.

Second, and more immediately, visible-to-telecom quantum frequency conversion provides a realistic mitigation strategy. This approach is already established in solid-state spin-photon-interface platforms. For example, Knaut, C.M. et al. *Nature* **2024**, *629*, 573 demonstrated a SiV-based quantum network in which 737 nm photons were converted to 1350 nm telecom photons using periodically poled lithium niobate (PPLN) waveguides. This strategy is particularly compatible with our molecular platform because molecular crystals can be processed as thin films and deposited onto pre-fabricated photonic structures, including silicon nitride or lithium niobate circuits, without requiring etching of the emitter host. We have revised the Outlook to explicitly discuss chemical red-shifting and quantum frequency conversion as complementary routes toward fibre-compatible molecular spin-photon interfaces.

Action: The Outlook section of our manuscript has been updated to explicitly discuss this strategy to convert to telecom frequencies and reference the relevant implementations to address this valid concern.

- T1 appears to be rather short and sets up a strong limitation on achievable T2. What is limiting T1 and could it be extended at e.g. lower temperatures? Although a 21 ms T2 is obtained with dynamical decoupling, the Hahn echo is only 12 µs. This may have important consequences on storage fidelity at long times and should be discussed.

Response: We appreciate this opportunity to expand on the achievable relaxation and coherence times. In the present single-molecule experiment we measure

$$T_1 = 21(2)\,\mathrm{ms}, \qquad T_2^{\mathrm{Hahn}} = 12.2(6), \mu\mathrm{s}, \qquad T_2^{\mathrm{XY8}} = 2.2(3)\,\mathrm{ms}$$

at $4.5\,\mathrm{K}$. We agree that T_1 ultimately bounds the achievable electron-spin coherence, with $T_2 \leq 2T_1$. However, the present Hahn-echo coherence time is not yet T_1-limited. Rather, the nearly two-orders-of-magnitude extension from Hahn echo to XY8 indicates that the dominant limitation of T_2^{Hahn} is slow dephasing from the proton-rich nuclear-spin bath of the molecule and matrix.

We attribute the current T_1 primarily to phonon-mediated spin-lattice relaxation, most likely through modulation of the zero-field-splitting tensor by low-energy lattice and intramolecular vibrational modes. This assignment is supported by our related work on (2,2')-dinaphthylcarbene in a (2,2')-dinaphthylketone host (Roggors, S. et al. *J. Am. Chem. Soc.*, **2025**, *147*, 36383–36392), where T_1 increased from approximately $5\,\mathrm{ms}$ at $9\,\mathrm{K}$ to approximately $23\,\mathrm{ms}$ at $4\,\mathrm{K}$ without clear saturation. This suggests that operation below $4\,\mathrm{K}$ may further increase the relaxation time. We have not yet performed a full temperature-dependent

single-molecule T_1 study for the present carbene, and we now state this explicitly.

The short Hahn-echo time is primarily a separate, dephasing-related limitation. As shown previously for related molecular qubits, deuteration of the qubit and host matrix can substantially suppress the proton nuclear-spin bath and should therefore increase T_2^{Hahn} and the decoupled T_2. We have revised the manuscript to distinguish these two improvement routes: lower-temperature operation to increase T_1, and isotopic engineering to reduce nuclear-spin-induced dephasing.

We also agree that the consequences for storage fidelity should be stated more explicitly. For an exponentially decaying electron-spin coherence, a storage interval (t) contributes a memory fidelity approximately $F \simeq \frac{1+\exp[-t/T_2]}{2}$. Thus, preserving an electron-spin superposition for $0.1 - 1\mathrm{ms}$ with $F > 0.99$ requires T_2 values of order $5 - 50\,\mathrm{ms}$, depending on the protocol and noise spectrum. The present $T_2^{\mathrm{XY8}} = 2.2(3)\,\mathrm{ms}$ is therefore already sufficient for repeated optical interrogation and proof-of-principle spin-photon-interface experiments, but further improvement will be required for long entanglement-purification or multi-gate memory sequences. For such longer storage times, the molecular platform offers an additional route: chemically engineered nuclear-spin registers introduced by site-selective isotopic labeling, which can act as longer-lived memory qubits coupled to the optically active electron spin.

Action: We have revised the Outlook to clarify that T_1 is most likely limited by phonon-mediated spin-lattice relaxation and may be improved by lower-temperature operation, whereas the present Hahn-echo T_2 is limited mainly by the proton nuclear-spin bath and can be improved by deuteration. We added the estimation of the storage fidelity above. We have also added a short discussion the usage of nuclear-spin registers as a route toward longer-lived quantum memories.

- The qubit is not stable at room temperature but is there evidence that at the operating temperature its stability can extend over e.g. days or longer? It is also not completely clear to me what is creation yield of the qubits - can deterministic creation be envisioned for e.g. integration into photonic structures?

Response: Indeed robust stability is a critical prerequisite for any long-term practical application. For carbenes in general the question is two-fold: First, the molecule needs to be chemically stable when embedded in the matrix without laser illumination, and second, as for many other molecules they should not bleach under illumination. Although we have not performed dedicated molecular lifetime measurements over long periods of time, we do not experience any notable decrease in fluorescence from photoactivated carbenes after annealing (around 200 K for about one hour). Furthermore, EPR measurements show signs of decay of carbenes within several hours only above 280 K. If the decay of carbene molecules obeyed purely the Arrhenius law, the lifetime, at the operating temperature of ca 4.5 K would be practically infinite.

Regarding photo stability we have performed additional illumination studies on carbene ensembles under various illumination conditions and recorded the decay of fluorescence (see

SI, VII. G.). We observe a close to quadratic increase of the decay rate of the fluorescence level with increasing laser power. Therefore it is reasonable to expect a two-photon-process leading to bleaching of molecules. To prevent shelving into an optically inaccessible dark state we had to increase microwave power for the latter experiments. This led to an increase in sample temperature by about 1 K. Nevertheless, the extracted average lifetime of di([1,1'-biphenyl]-4-yl)carbene molecules under continuous 3 nW of resonant laser excitation (including the required microwave fields for repumping) is ≈ 119 hours. Such a two photon-process might be mitigated by coupling of di([1,1'-biphenyl]-4-yl)carbene molecules to photonic cavities and thus effectively reducing excited state lifetime and shelving into metastable singlet states.

To answer the second question on the creation yield we have evaluated data from the manuscript to get an estimate of the conversion efficiency of the precursors to the carbene molecules. Our cw-EPR experiments demonstrate that photoactivation with a relatively high-energy 395 nm UV LED curing lamp (Jupitertech, 12.0 W/cm^2, distance ca. 3 cm) exclusively yields resonances from our triplet ground state carbenes. As shown in the nascent rotation spectra (see SI Fig. S32), no other EPR-active species are generated (at least with a factor of $\sim 1:1000$, according to the experimental SNR). We can therefore conclude that the diazomethane precursors are efficiently converted into the target molecular qubits without forming any paramagnetic decomposition products. This observation is in line with the general perception that, "Carbenes are conveniently generated from nitrogenous precursors such as diazo compounds or diazirines upon irradiation or thermolysis, which takes place very cleanly and efficiently." (Hirai, K. et al. *Chem. Rev.* **2009**, *109*, 3275.) Beyond the EPR data, we have looked at fully activated regions on the crystal spectroscopically (Figure 2a in the manuscript), measured their spatial and spectral extent and compared to those of a single carbene molecule. Taking into account the nominal doping concentration we estimate a creation yield $\eta = \frac{\#\text{carbenes}}{\#\text{precursors}} \approx 1.4(6)$ provided that the distribution of precursors throughout the whole crystal is homogeneous. As 1.4 is larger than the seemingly obvious physical limit of 1 we suspect either a higher local concentration of precursors or a too rough estimate or a combination of both. Indeed, from previous similar studies (see e.g. Roggors, S. et al. *J. Am. Chem. Soc.*, **2025**, *147*, 36383–36392) and the current molecular samples we have reason to believe that highly doped crystals can exhibit a spatially inhomogeneous distribution of dopant molecules. Initial investigations within a targeted 10–1000 ppm doping range reveal a distinct dopant-induced coloration of the otherwise transparent crystal, which can be easily studied under a microscope at room temperature with transmitted white light. In some crystals, we observe spatial variations of this coloration which strongly suggests an inhomogeneous distribution. We suspect this to stem from the nature of substitutional doping in combination with slow crystal growth dynamics. While we have not yet evaluated these findings further, we hypothesize that this inhomogeneity may eventually be relieved at lower dopant concentrations.

We have reasons to believe that deterministic creation of carbenes from precursors for example close to photonic structures is straightforward because photoactivation and fluorescence detection can be done simultaneously. Therefore, one exemplary strategy for deterministic creation would be activation in a confocal microscope with for example 580 nm laser light

such that just-activated carbenes within the confocal volume would lead to increased fluorescence. When the activation of a single carbene has been detected the activation laser would be switched off immediately to avoid activation of further emitters. It is an open challenge to find an appropriate combination of precursor doping density, laser wavelength and intensity to allow for the detection of the activation of a single carbene by photon-counting before a second carbene will be activated. For our current doping density we experience fast and continuous increase of fluorescence during activation, which does not exhibit a step-wise increase expected for number-resolved activation. Therefore, the current doping concentration might be too high for the proposed method.

Action:

- We have added a section to the SI (Sec. VII. B.) where we show new measurements and give more details about the activation/creation of di([1,1'-biphenyl]-4-yl)carbene molecules. We have referenced this part of the SI int the text.
- We have added a section to the SI (Sec. VII. G.) where we present new data about photo-bleaching of di([1,1'-biphenyl]-4-yl)carbene molecules.
- We have added a sentence to the manuscript about chemical and photo-stability including a reference to the SI.

• The emission quantum yield is low. Can this be improved as it could also make potential applications much more difficult to achieve. In particular, taking into consideration the other properties of the qubit (cyclicity, spin T1, etc..), is single-shot spin readout feasible?

Response: We thank the reviewer for this question and agree that a higher PLQY would greatly facilitate potential applications. In the following we decided to elaborate on the topic in more detail due to the shared interest of all reviewer. According to our current understanding of the rates between the triplet and the singlet states, we believe that a full single-shot readout of the triplet spin of the presented molecule is not possible without enhancement of the photon emission (i.e. the PLQY, but also the quantum efficiency of the triplet to triplet deexcitation) and collection efficiency.

Apart from a full single-shot readout, a repetitive readout scheme can be employed to discriminate the $T_{0\mathrm{y}}$-$T_{0\mathrm{z}}$-subspace from the $T_{0\mathrm{x}}$-subspace with a high SNR. This utilizes the fact, that optical excitation on e.g. the YY' transition populates the $T_{0\mathrm{z}}$ state via ISC, but only weakly populates $T_{0\mathrm{x}}$. With this method, a SNR of the spin readout of 0.86 (for a single readout operation) can be achieved in our experimental setting (with a photon detection efficiency of 1.3%), almost reaching the threshold for single-shot readout. Starting with these properties, the SNR of the readout can be improved in multiple ways. From a quantum optics standpoint, both the PLQY and detection efficiency can be increased by coupling the molecules to optical cavities or dielectric antennas (see Wang, D. et al. *Phys. Rev. X*, **2017** *7*, 021014 and Lee, K. G. et al., *Nature Photonics*, **2011** *5*, 166–169).

In addition, chemically altering the presented carbene molecule to increase PLQY or to obtain a higher cyclicity is also feasible. Generally, the photoluminescence quantum yield can be enhanced through two primary strategies: 1) increasing the oscillator strength of the

radiative transition, and 2) suppressing unwanted non-radiative decay pathways. In the following we would like to lay out a clear way forward for a future molecular design:
For the first strategy, the recent comprehensive study by Arnold et al. (*Adv. Opt. Mater.* **2024**, *12*, 2400697) on mono-substituted *N*-donor triarylmethyl (trityl) radicals provided a blueprint for this systematic PLQY tuning optimization. Their work establishes that the spectral properties are linked to the electron-donating strength of the functional group, which can be parameterized by its ionization energy. The authors demonstrate a relationship between a donor's ionization energy and the overall PLQY of the radical follows a distinct bell-shaped curve. Specifically, functionalizing the system with very weak or very strong electron donors results in poor emission due to large energy barriers or competitive non-radiative decay pathways, respectively. Conversely, coupling the radical with medium-strength *N*-heterocyclic donors minimizes non-radiative decay and facilitates fast intramolecular charge transfer. This targeted approach leads to highly efficient emission from the charge-transfer state, allowing the PLQY of the TTM radical to experimentally reach up to 91% when systematically paired with an optimal medium-strength donor such as carbazole.

Ghosh, P. et al. (*Nature* **2024**, *629*, 355) demonstrated that in deep red/ near-infrared emitting organic molecules, the non-radiative decay rate is typically dominated by strong coupling to high-frequency vibrational modes (1,000–1,600 cm^{-1}), such as carbon-carbon stretch modes. It was shwon that excitions could be effectively decoupled from these detrimental high-frequency vibrations by selecting moieties where the participating molecular orbitals possess a symmetry-imposed non-bonding character. Using subtle changes in the connectivity of a carbazole donor, the authors could thereby increase the PLQY of a trityl radical based emitter from 14% to 94%.

To further improve the PLQY, we can exploit kinetic isotope effects as shown for 2,2'-dinapthylcarbene (DNC) in our recent paper (Roggors, S. et al. *J. Am. Chem. Soc.*, **2025**, *147*, 36383–36392) and e.g. Dibenzoterrylene (DBT, Mishra, K. et al. *Chem. Sci.* **2024**, 16, 90). In principle, by substituting hydrogen atoms with heavier deuterium more vibrational quanta are required to bridge the electronic energy gap, which reduces the Franck-Condon overlap factors and suppresses the non-radiative IC rate (V. L. Ermolaev, *Russ. Chem. Rev.* **2001**, *70*, 471). In our previous study on DNC this perdeuteration led to an increase by a factor of ca 1.5 in the measured PLQY (77 K in frozen MeTHF). For DBT, bulk experiments at room temperature revealed that the fluorescence quantum yield in different solvents range from 10–35% (C. Erker, T. Basché, *J. Am. Chem. Soc.* **2022**, *144*, 14053). Upon perdeuteration, it was found that the quantum yield increase up to roughly 60% (Mishra, K. et al. *Chem. Sci.* **2024**, *16*, 90).

It is important to acknowledge that optimizing this property often inherently impacts several other characteristics of engineered molecules. For example, modifying a specific substituent to improve the emission efficiency or shift the emission wavelength can simultaneously alter excited-state lifetimes, spin coherence times, the Debye-Waller factor, and the fidelity of the spin-selective optical transitions. Furthermore, any structural modification to the carbene risks disrupting the precise isosteric matching and the overall packing of the ketone precursor matrix, setting new baseline conditions for the protective environment of

our qubits. However, when we consider the substantial improvements demonstrated in the present work compared to our previous system (Roggors, S. et al. *J. Am. Chem. Soc.*, **2025**, *147*, 36383–36392), we remain confident that with systematic, iterative molecular design, decisive advancements will be made in this field, ultimately bridging the gap to a more robust single-shot spin readout.

Action: Related to the PLQY, we have added a new measurement in the SI, corroborating the PLQY measurement of an ensemble in frozen solution at 77 K for a single molecule in a crystalline matrix at 4.5 K (SI Sec. VII. F.). We have furthermore added a discussion of the prospect for full single shot readout and repetitive readout of a subspace of the triplet spin that almost reaches the SNR for single-shot readout (SI Sec. VII. M.). In the outlook, we now mention the low PLQY as one deficit of the molecule, highlighting both chemical and quantum optical measures for improvement.

Minor comments

- introduction is especially short and should give more context to the study. For example, what are the key parameters/experiments to consider and how do they look like in competing systems?

  Response: We thank the reviewer for this constructive feedback, which has helped us better frame the significance of our work. We agree that a broader context is necessary to highlight the advantages of our approach. In response, we have significantly expanded the Introduction to detail the key parameters of a robust spin-photon interface and directly compare our platform against competing solid-state systems. Specifically, we now explicitly discuss leading semiconductor color centers, such as the nitrogen-vacancy (NV) and silicon-vacancy (SiV) centers in diamond, as well as defects in silicon carbide. We highlight their key performance parameters — such as room-temperature spin lifetimes, while explaining their inherent limitations regarding extraneous electron defects, charge traps, optical spectral diffusion, and phonon-limited spin coherence. By outlining these specific parameters and then transitioning to the perspective of using molecular systems as spin-photon interfaces, we believe the reader is now significantly better introduced to the topic. On a sidenote, we also added a more quantitative comparison of different spin-photon interfaces to the SI, Sec. VIII.

- p. 5: what is causing the additional decoherence of the optical transition? The claim 'exceptional stability' should be supported by a comparison to e.g. color centers in diamond. What is the origin of the optical stability?

  Response: We thank the reviewer for raising this important distinction. We now explicitly distinguish between the linewidth of an individual optical resonance and the long-term stability of its center frequency. For the single molecule shown in Fig. 3(c)–(f), the measured linewidth is $38(2)\,\mathrm{MHz}$, while the lifetime-limited linewidth inferred from $\tau_{\mathrm{exc}} = 24(2)\,\mathrm{ns}$ is $6.6(5)\,\mathrm{MHz}$. Thus, the optical transition is not yet transform limited and exhibits an additional pure-dephasing contribution. However, repeated spectroscopy over more than one hour shows that the center frequencies of two individual molecular resonances fluctuate only by 2.4 and $2.6\,\mathrm{MHz}$, respectively. The "exceptional stability" claim therefore refers specifically to the very small long-term spectral diffusion, not to a transform-limited linewidth.

To investigate the origin of the additional optical broadening, we performed new temperature-dependent single-molecule excitation spectroscopy. As shown in the new Sec. VII. H. of the SI, increasing the temperature from $4.67, \mathrm{K}$ to $9.5, \mathrm{K}$ causes both a systematic shift and broadening of the zero-phonon line (ZPL). Both quantities are well described by an Arrhenius dependence. For the linewidth, the fit gives an extrapolated zero-temperature linewidth of $\Delta\nu_0 = 23(7)\,\mathrm{MHz}$, an infinite-temperature broadening amplitude of $\Delta_\infty = 8(4)\,\mathrm{GHz}$, and an activation energy $E_\mathrm{a}/k_\mathrm{B} = 27(4)\,\mathrm{K}$. We therefore attribute the thermally activated part of the additional decoherence to coupling of the optical transition to low-energy lattice or intramolecular vibrational modes. The extrapolated linewidth remains above the lifetime limit, so a residual low-temperature dephasing contribution remains unresolved and will require measurements at lower temperature and reduced perturbation from optical and microwave driving.

The origin of the high spectral stability is different. The small hour-scale drift indicates that slow electric-field noise and configurational rearrangements are strongly suppressed in the present host. We attribute this to the isosteric and rigid molecular crystal, the locally excited character of the optical transition, the absence of extended semiconductor charge traps, and the post-activation annealing step, which relieves local strain. The trapped nitrogen molecule generated during photoactivation also does not appear to introduce dynamic disorder: as discussed in the revised Supplemental Information, the observed linewidth and long-term stability indicate that it acts as a sterically constrained local neighbor rather than as an active source of spectral diffusion.

To place these values in context, we now compare the measured spectral diffusion to representative color-center platforms. Inversion-symmetric group-IV centers such as SiV centers are protected against first-order Stark shifts and are well known for high optical stability. Non-centrosymmetric defects such as NV centers are more sensitive to local electric-field fluctuations and can exhibit substantially larger spectral wandering in charge-noisy environments. The notable point here is that the carbene achieves only 2.4–$2.6\,\mathrm{MHz}$ hour-scale spectral diffusion despite being a molecular emitter without inversion-symmetry protection. This supports the conclusion that the molecular host provides an unusually quiet local electrostatic environment.

Action: We added temperature-dependent single-molecule spectroscopy to Sec. VII. J. of the SI and revised the main text to clarify that the residual linewidth broadening is attributed mainly to phonon-assisted pure dephasing, whereas the exceptional spectral stability refers to the very small hour-scale drift of the transition frequency (Sec. "Single Molecule Spectroscopy"). We also added a comparison to representative diamond color centers (Sec. "Conclusion and Outlook" and SI, Sec. VIII.).

- Fig. 4d - the meaning of the y axis label is not clear to me. How many repetitions of the sequence b are performed to yield Fig. 2c?

Response/Action: Indeed the initial axis label was not sufficiently clear. We have therefore changed the labels of figure 4c and d. Furthermore, we have changed the presentation of the

data in figure 4d to allow for a straight forward estimate of relevant quantities and a quick relation to figure 4c. Now, the fluorescence response of a single molecule to a laser pulse is expressed as average fluorescence rate in figure 4d for the two cases of a microwave π-pulse or none being applied prior to the laser pulse. It is now easier to estimate the average amount of photons per fluorescence pulse to $\sim 5\,\mathrm{kcounts/s} \cdot 2\,\mu\mathrm{s} = 0.01\,\mathrm{counts}$ for the case of a π-pulse. The latter value is consistent with the data shown in figure 4c where the average number of photons per readout event is in the range of about 0.003 to 0.01. For the acquisition of the data shown in figure 4d about $2 \cdot 10^6$ repetitions of the pulse sequence in figure 4b have been averaged for each of the two cases.

- p. 8: 'more than an order of magnitude': maybe add Vasilenko et al., Nat. Materials, 2026 which reports T2 DD = 2 ms and T2 Hahn = 0.6 ms for an ensemble of nuclear spins.

  Action: We changed the respective sentence to compare our results with molecular electron spins, while highlighting the improvement one can have when utilizing nuclear spins, as in Vasilenko et al. The sentence now reads: "These single-molecule *electron spin* values represent a substantial leap for molecular qubits, extending previously reported values on ensembles by more than one order of magnitude." At a later point, we mention: "...and coherent memory times can be extended significantly further by employing nuclear spins [Vasilenko et al.]".

- p. 8 'significantly lower noise' - In this case why is the Hahn echo T2 much shorter than e.g. for the NV centers?

  Response: We thank the reviewer for pointing out this ambiguity. In the original wording, "significantly lower noise" was intended to refer specifically to extrinsic electronic-defect and charge noise in the host, not to the total magnetic noise experienced by the electron spin. We agree that this distinction should be made explicit.

  The present Hahn-echo T_2 is much shorter than record values for NV centers because the dominant decoherence channel for the spin of our molecule is the dense proton nuclear-spin bath of the protiated qubit and matrix molecules. This is distinct from the electronic- and charge-noise channels that often affect color centers through nearby paramagnetic defects, surfaces, implantation damage, or charge traps. In our system, the excellent optical spectral stability indicates a low level of slow electric-field noise, whereas the short Hahn-echo time reflects magnetic dephasing from surrounding $^1\mathrm{H}$ nuclear spins.

  This assignment is supported by the strong extension of coherence under dynamical decoupling: in the present single-molecule experiment, T_2 increases from $12.2(6)\,\mu\mathrm{s}$ under Hahn echo to $2.2(3)\,\mathrm{ms}$ under XY8. Such a large improvement is characteristic of decoherence dominated by slowly fluctuating nuclear-spin baths rather than by fast electronic spin relaxation. The measured $T_1 = 21(2)\,\mathrm{ms}$ also shows that the Hahn-echo time is not currently relaxation-limited.

  The relevant comparison to diamond is therefore not that the present protiated molecular crystal has a weaker nuclear-spin bath than isotopically engineered diamond. Rather, the advantage of the molecular platform is that the electronic and nuclear environments can be

chemically engineered independently. In the present system, deuteration of both the qubit and the matrix should strongly reduce the proton nuclear-spin bath, analogous in purpose to isotopic purification in diamond. In related carbene systems (see Roggors, S. et al. *J. Am. Chem. Soc.*, **2025**, *147*, 36383–36392), deuteration has already yielded Hahn-echo coherence times in the several-hundred-microsecond range. We therefore expect a substantial improvement of Hahn-echo T_2 upon deuteration, while isotopically purified ^{12}C diamond remains the benchmark for the longest NV electron-spin coherence times.

Action: In the revised manuscript, the sentence was removed completely. In the new introductory paragraph we now explicitly highlight the difference in noise due to charge effects of the matrix / the crystal influencing the optical spectral stability. In the outlook, instead of relying on an ambiguous claim of ”significantly lower noise”, we now discuss the limits for the spin coherence time, and how to improve them.

Reviewer: 2

The authors demonstrate optical spin detection and coherent manipulation of an individual ground-state molecular spin for the first time. This presents an important advance for the application of molecular qubits. The demonstrated combination of properties (∼38 MHz optical linewidths, ∼24 ns optical lifetimes, long-term spectral stability, and ∼ms coherence under dynamical decoupling) are attractive, even when compared to much more established solid-state defect systems. The paper is very clearly written, well presented, and the work is thorough. The detailed methods and supporting information are valuable. I believe the importance of this demonstration, combined with the quality of the manuscript warrant publication in Nature. I do have some comments which the authors should address before publication, however.

1. In the abstract, the authors state “however, the lack of a robust spin-photon interface combining bright fluorescence, high spectral stability, and the persistent spin lifetimes inherent to ground-state systems has prohibited the detection of individual molecular qubits” They should rephrase this statement as it sounds like there has never been a detection of an individual molecular spin, and that all detection methods for an individual molecular qubit would require all of the above features (which is not the case).

   Response: We thank the reviewer for highlighting this point and agree that individual molecular spins and qubits can be detected through alternative modalities. Furthermore, in principle, basic optical detection does not strictly mandate all of the mentioned stringent criteria simultaneously. Our original intention was to highlight that the simultaneous convergence of these specific properties is required to build an optically addressable quantum networking node. To prevent any misunderstanding regarding the broader perspective of single-molecule detection, we have rephrased this statement in the abstract to focus precisely on the demands of the spin-photon interface.

   Action: We rephrased the sentence to ”However, a molecular platform that concurrently satisfies the demands of a robust spin-photon interface, namely the combination of bright fluorescence, high spectral stability and long ground-state spin lifetimes, has yet to be realized at the single-molecule level.”

2. The paper should include citations to relevant earlier work on hole-burning spectroscopy of carbenes performed on similar compounds as these relate to the authors' work. For example, there are several papers by Kozankiewicz et al. from the 1990s which would be relevant to cite.

   Actions: We appreciate the reviewer pointing out this oversight. The missing references have now been incorporated into the manuscript:

   - Anderson, R. J. M. & Kohler, B. E. Electron nuclear double resonance of triplet diphenylmethylene in P 21 benzophenone: Conformation and spin distribution. *The Journal of Chemical Physics* **65**, 2451–2459 (1976).
   - Graham, D. J. & Wang, C.-L. A magnetic field study of excited triplet diphenylmethylene. *The Journal of Chemical Physics* **85**, 4441–4445 (1986).
   - Haider, K. W., Platz, M. S., Despres, A., Lejeune, V. & Migirdicyan, E. Triplet-triplet fluorescence and spin polarization: Diphenylcarbene and dibenzocycloheptadienylidene. *The Journal of Physical Chemistry* **94**, 142–147 (1990).
   - Wasserman, E. et al. Electron paramagnetic resonance of 9,9'-dianthrylmethylene. Linear aromatic ground-state triplet methylene. *Journal of the American Chemical Society* **93**, 6335–6337 (1971).
   - Graham, D. J. & Lin, T.-S. High-Resolution Absorption Spectra of Diphenylmethylenes. *Chemical Physics* 411–420 (1982).
   - Kozankiewicz, B., Gudmundsdottir, A., Orrit, M., Platz, M. & Tamarat, Ph. Hole burning on a triplet-triplet transition: 2,2-dinaphthylcarbene in n-hexane at 1.7K. *Journal of Luminescence* **86**, 261–263 (2000).
   - Kozankiewicz, B. et al. A Hole-Burning Study of the Zero-Field Splitting of the Triplet Ground and Excited States of 2,2-Dinaphthylcarbene in n-Heptane and n-Hexane at 1.7 K. *The Journal of Physical Chemistry A* **104**, 7464–7468 (2000).
   - Kozankiewicz, B. et al. Zero-Field Splitting of the Electronic Ground and Lowest Excited Triplet States of 2,2-Dinaphthylcarbene in n-Hexane at 1.7 K. *The Journal of Physical Chemistry A* **103**, 3155–3162 (1999).

3. To assess the brightness of this system, the authors should clarify their single-molecule saturation count rate, including a saturation curve.

   Response: We thank the reviewer for pointing out the missing data on the optical saturation properties of a single molecule. We have added a microwave-assisted single-molecule saturation measurement to the Supporting Information. Under the resonant detection conditions used for single-molecule spectroscopy, the maximum detected count rate is $I_{max} = 4.58$ kcounts/s at a laser power of $P_{max} = 22.79$ nW. Due to the more complex relationship between laser power and fluorescence count rate, we refrain from quoting saturation values, but the maximum amount of fluorescence and the respective laser power. These values refer to the detected fluorescence count rate in the present confocal setup, using phonon-sideband

detection after spectral filtering of the resonant excitation laser (total photon detection efficiency is estimated as 1.3%), and should therefore not be interpreted as the total photon emission rate of the molecule.

The saturation behavior of this system is less directly accessible than for a closed two-level emitter. Because single-molecule experiments are performed by resonantly addressing one spin-selective optical transition ($T_{0\mathrm{y}}$ in this case), optical excitation pumps the molecule into the $T_{0\mathrm{z}}$ and $T_{0\mathrm{x}}$ spin state, which is dark for the addressed transition. Continuous fluorescence therefore requires microwave repumping through the ZX and ZY transitions. Consequently, the observed steady-state fluorescence rate depends on the applied microwave Rabi frequencies and is limited by the combined optical excitation, intersystem-crossing and microwave-repopulation dynamics, rather than by saturation of the optical transition alone.

We also would like to clarify the distinction between this steady-state photon count rate and the pulsed readout traces in Fig. 4d. The latter show the transient fluorescence immediately after spin preparation; the signal decays as the molecule is optically initialized into $T_{0\mathrm{z}}$. Thus, Fig. 4d is the relevant quantity for the detected photons per spin-readout event, whereas the new SI saturation curve provides the steady-state single-molecule brightness under continuous resonant excitation and microwave repumping.

Action: We added a new SI Sec. VII. F. including the saturation curves and fitted values $I_{\mathrm{max}} = 4.58$ kcounts/s and $P_{\mathrm{max}} = 22.79$ nW. We also changed the axes of Figs. 4c,d to show absolute detected fluorescence quantities and added a short clarification in the main text/Methods that the detected fluorescence rate is microwave-repump limited under resonant excitation.

4. The authors use photoactivation at 580 nm. However, most previous work on carbenes used UV excitation wavelengths: the authors should include a comment on the process at play and why they are able to use longer wavelengths than in previous studies.

Response: We thank the reviewer for bringing up the rather surprising fact that the vast majority of previous photochemical studies on generation of related diphenylcarbenes have utilized light sources that include UV or blue light (see e.g. D. C. Doetschman, et al., *Chem. Phys. Lett.* **1976**, *38*, 18; D. J. Graham, T.-S. Lin, *Chem. Phys.* **1982**, *73*, 411; M. Aloshyna, et al., *J. Phys. Chem. A* **2000**, *104*, 3391).

We hypothesize that the previous usage of UV and blue light was widespread because the activation from higher excited states is considerably more efficient and, hence, the possibility of using longer wavelengths (like 580 nm) was mostly overlooked in the literature.

Indeed, we also commenced our initial experimental efforts by using a high-energy 395 nm UV LED curing lamp (Jupitertech, 12.0 W/cm^2) and only realized in later experiments the potential to also use longer wavelength light. In our case, we find that activation with higher energy blue or green light is much more efficient than using 580 nm. The mechanism governing this wavelength-dependent efficiency has only recently been elucidated (Piteša, T. et al., *J. Am. Chem. Soc.* **2020**, *142*, 9718). In this study the authors could successfully generate diphenylcarbene at 500 nm, but the quantum yields for the conversion were exceptionally low (Φ = 0.020) compared to UV irradiation at 300 nm (Φ = 0.36 to 0.46 de-

pending on the solvent used). According to this and a follow-up study by Brusar, V. et al. (*J. Org. Chem.* **2023**, 88, 4286), excitation to higher-lying singlet states (S_n, such as S_2 via UV/blue light) drives the system toward a non-planar S_1/S_0 conical intersection where outward forces effectively separate the carbene and N_2 fragments, resulting in highly efficient photoelimination. Conversely, excitation to the S_1 state (via lower-energy visible light, e.g., > 500 nm) leads to an in-plane deactivation pathway where the molecular forces actually hinder fragment separation. As a result, direct S_1 excitation is 10 to 50 times less efficient than higher S_n excitation.

In this context, we assume activation at 580 nm to be advantageous for our single-molecule microscopy experiments in that the longer wavelength allows us to more selectively activate the carbene molecules, minimize background autofluorescence, and avoid photodamage to the host matrix and the qubits themselves. The reduced photoelimination efficiency is hereby easily compensated by a higher photon flux in our confocal laser setup.

Action: We have included a short comment in the manuscript with reference to a detailed section in the SI (see Sec. VII. B.) for the interested reader.

5. The authors state "This level of control, achieved via confocal microscopy, allows the qubit molecule density to be regulated independently of the overall doping level." Given the similarity between photoactivation wavelength (580 nm) and resonant ZPL excitation wavelength (~ 595 nm), the authors should clarify to what extent this holds. In other words, to what extent does resonant ZPL excitation cause photoactivation?

Response/Action: This is a very interesting question that has motivated further experiments. Initially, we had neglected photoactivation of carbenes by our resonant excitation laser for three reasons. First, the wavelength is even longer than 580 nm and activation even less likely. Second, the optical intensity under resonant excitation is roughly two orders of magnitude lower than for 580 nm activation studies. Third, activation by the resonant excitation laser would happen unnoticed because carbenes would be activated into their nascent state (site 1 or 2) in which their ZPL is at least 2 nm shifted to the blue with respect to the ZPL of site-1-molecules after the annealing step (see SI Figure 37). Hence, any carbene activated in this manner would only show up in confocal scans or fluorescence excitation spectra after an additional annealing step.

We have now added experimental results to SI Sec. VII. B. where we estimate the photoactivation yield and investigate photo-activation with 594.6 nm laser light. It turns out that prolonged illumination with 594.6 nm laser light indeed activates carbene molecules. For continuous illumination with about 3 nW (common operation condition for single spin experiments) we estimate the full activation of the present precursors within a few days.
We have referenced this new section of the SI in the text.

6. The authors should clarify whether they observed any photostability issues with the samples. To what extent are the generated carbenes robust to higher laser power, for example, and do they observe stability over long (week) timescales?

Response: During our experiments in the course of several months we do have observed

some bleaching events[1] of individual carbene molecules. To shed light on the stability of our carbenes under higher laser illumination we have performed additional experiments:
We have applied resonant laser excitation up to 46 nW to an ensemble of activated and annealed carbene molecules and monitored the decrease of fluorescence. To really increase the effective optical excitation rate and avoid shelving into dark spin states we increased the microwave power on spin transitions ZX and ZY compared to measurements at a few nW of laser power. After analyzing the decay rate of the fluorescence level for several laser powers we find a quadratic dependence on laser power, which hints towards a two-photon bleaching process. For the laser power used in the experiments shown in the paper we have estimated a single molecule lifetime of about 118 hours. Unfortunately, under these burning conditions the temperature of the sample increases due to the higher microwave power and we are unable to disentangle thermal and purely optical effects on burning. A coupling of single molecules to photonic cavities can decrease excited state lifetimes and also shelving into the metastable singlet state. This might reduce two-photon burning processes. Furthermore, reducing temperature might lead to higher stability.

Action: We have added a sentence about photo-stability to the text. Furthermore, we have added a reference to the respective section of the SI including the new measurement data (SI Sec. VII. G.).

7. The authors should include a comment on the role of the liberated nearby nitrogen: do they expect this to be detrimental, e.g., for inducing disorder?

   Response: In our current view, the nitrogen acts merely as a benign, sterically constrained neighbor rather than a significant source of disorder. To shine light on this relevant aspect for the performance of our qubits, we consider long range and local effects in the following.

   As far a long range effects are concerned, we perform thermal annealing to 200 K following photoactivation, which actively relieves local matrix strain induced by the photocleavage of nitrogen and stabilizes the crystalline environment. This is monitored via in-situ EPR spectroscopy at around 120 K to 130 K, demonstrating that the structural relaxation reaches a stable equilibrium with constant observables, e.g. the resonance signal in the EPR and the emission wavelength of the ZPL in the cryo confocal measurements. Importantly, these observables do not shift over the wide range of doping ratios studied so far (0.5%-2.5 ppm), which suggests local matrix rearrangements rather than macroscopic crystal lattice restructuring to relieve strain. To the same effect, for the sub part-per-billion (ppb) levels intended for future quantum network applications, the average distance between individual qubit molecules is on the scale of hundreds of nanometers to micrometer. At these sparse concentrations, we do not expect macroscopic effects or correlated disorder arising from overlapping lattice strain fields between multiple carbene defect centers.

   Regarding the local environment, our calculations indicate i) restructuring of the matrix upon nitrogen cleavage mostly along the y-direction (pointing along the C=O bond), and ii) that the nitrogen molecule remains trapped in the crystal lattice in close vicinity to the

[1] Due to the applied resonant optical excitation, we are unable to distinguish between bleaching and strong spectral drift/jumps. Thus, we conservatively attribute the observed effects to bleaching.

carbene center after photoelimination in accordance with previous experimental studies on congested carbenes (Kawano, M. et al. *J. Am. Chem. Soc.* **2007**, *129*, 2383). The precise molecular dynamics of this nearby nitrogen warrants further investigation, because current data does not allow for a conclusive assessment of a remaining detrimental effect on the qubit's properties.

Action: A brief comment summarizing these points was included in the main text and a more detailed section was added to the SI (see Sec. IV. F. 9.) to clarify the role of the liberated nitrogen for the reader.

8. In Fig. 1b, the authors display a calculated transition-dipole moment which appears to have 'chosen' one of the two biphenyl units' directions. It would be helpful to further clarify why this arises.

Response: In fact, the TDM depicted in the original Figure 1b appears to break C_2 symmetry by pointing toward a specific biphenyl unit. We thank the reviewer for their observation, which motivated us to rigorously re-examine the validity of our complete active space self-consistent field (CASSCF) model used for property prediction.

Upon closer inspection, we realized that our initial choice of a 12-electron, 12-orbital (12,12) active space was physically inappropriate for this system. As now detailed in our revised Supporting Information, the pairing theorem associated with the alternancy symmetry of such diradicals dictates that the active space must comprise $4n + 2$ electrons and an equal number of orbitals (where n is a non-negative integer) to prevent artificial symmetry breaking as also mentioned in Kopp, S. M., et al., *J. Am. Chem. Soc.* **2024**, *146*, 27935, p.5 SI; for the original paper see Poh, Y. R., et al., *J. Am. Chem. Soc.* **2024**, *146*, 15549. Apparently, our previous use of a (12,12) active space violated this $4n + 2$ rule, which led to an unphysical weighting bias toward one of the biphenyl arms and, most likely caused the artifactual tilting of the computed TDM vector.

To rectify this error, we have repeated the SA8-CASSCF/QD-SC-NEVPT2/def2-TZVPP calculations employing a contracted (10,10) active space and find that the balanced model successfully preserves the molecular symmetry and produces a TDM vector that aligns along the long axis of the molecule.

We would like to point out, that alongside the updated active space a more comprehensive model is required to explain the experimentally observed T_{0z}-selectivity in the excited state. Briefly, because direct spin-orbit coupling between the T_1 and S_2 states is unexpectedly weak, the highly efficient intersystem crossing likely proceeds via a second-order spin-vibronic mechanism. In this framework, the T_1 state borrows transition intensity through non-adiabatic vibrational mixing with a closely lying T_2 state that strongly couples to the singlet manifold. For more information we would like to refer the reviewer to Sec. IV. F. 8. in the SI.

Action: We have updated Figure 1b, as well as the corresponding theoretical discussion and results in both the main manuscript and the Supporting Information, to reflect these corrected calculations.

9. In Fig. 2b: it would be helpful to include a diagram of which transitions result in the substructure for clarity.

   Action: We added a diagram showing the triplet ground- and excited state levels (new fig. 2b), as well as the relevant transitions between the energy states. It contains a unified nomenclature for the transitions, which is implemented also in figure 2c. The same nomenclature was also implemented across the manuscript for homogeneity.

10. The authors state "we find this transition to involve a formal redistribution of spin density from the central carbene p-orbital to an adjacent phenyl ring (Fig. 1b)." however this does not seem to be clear from Fig. 1b. The authors should update the manuscript accordingly.

    Response: We thank the reviewer for pointing out this discrepancy that Figure 1b does not visually corroborate the stated redistribution of spin density. The statement is, in fact, supported by our state-specific CASSCF/NEVPT2 calculations detailed in the Supporting Information. Specifically, Supplementary Figure 27c displays the difference spin density between the ground (T_0) and first excited (T_1) states. This plot mathematically subtracts the T_1 spin density from the T_0 spin density, explicitly illustrating the depletion of spin density at the central carbene carbon and its corresponding accumulation on the adjacent phenyl rings upon excitation.

    Action: We apologize for the incorrect figure reference in the main text. We have updated the manuscript accordingly to correctly direct the reader to the relevant Supplementary Figure (Fig. 27c) to support this claim.

11. In Fig 1b, the authors display calculated ISC rates. However, it is only made clear later in the text how these have been determined. (A brief update to the caption would be sufficient to fix this.)

    Response: Following the update to a (10,10) CAS and the adoption of a more comprehensive theoretical framework to explain our experimental observations (detailed in Sec. IV. F. 8. of the SI), the relative ISC rates can no longer be derived purely from ab initio predictions. Consequently, we have instead estimated these ratios based on our experimental data. In SI Sec. H and L we have added estimates about intersystem crossing derived from experimental results. While we cannot clearly determine differences for ISC from $T_{1x'}$ and $T_{1y'}$ into S and similarly from S to T_{0x} and T_{0y}, we conclude that ISC from $T_{1z'}$ to S is at least five times faster than for the other two states and ISC from the singlet manifold into the triplet ground state is faster than $1\,\mu$s and appears predominantly into T_{0z} ($\gamma_{ISC,z}/(\gamma_{ISC,x} + \gamma_{ISC,y} + \gamma_{ISC,z}) \approx 0.99967$).

    Action: We have therefore removed the rates from arrows symbolizing ISC in Figure 1b and have highlighted the different rates by different shades of the arrows along with a description stating z'- and z-selectivity for ISC to and from the singlet manifold, respectively. We have added links to the respective section of the SI (Sec. IV. F. 8, Sec. VII. H. and Sec. VII. L.)

12. In the outlook, the authors should include a more critical discussion of the opportunities the work opens up. For example, solid-state defects can be coupled to photonic cavities so the authors should clarify the real opportunity for added-value.

Response: We appreciate the suggestion to expand on the unique opportunities this platform opens up, particularly concerning cavity coupling and photonic integration. To clarify the added-value over traditional solid-state systems, we updated the Conclusion and Outlook section to address these points directly.

First, we contrast the integration methods of our molecular qubits with those of semiconductor defects. Traditional color centers typically require top-down nanofabrication or etching of the host substrate to couple with photonic structures, a process known to introduce local lattice strain and degrade spectral stability. Molecular systems, by contrast, can be grown as standalone thin films or deposited directly onto pre-fabricated photonic integrated circuits, minimizing substrate-induced degradation.

Second, we address the reviewer's point about cavity coupling by highlighting the robust electronic structure of the carbene. The energy separation between the spin-selective transitions (at least 1.555 GHz) is more than two orders of magnitude greater than the lifetime-limited linewidth (6.6 MHz). This broad splitting ensures that high-fidelity spin addressability is maintained even when excited-state lifetimes are shortened via cavity coupling to improve the photoluminescence quantum yield, Debye-Waller factor, and photon collection efficiency. Finally, we added a brief discussion on using periodically poled lithium niobate within this framework to convert visible photons to the telecom band for long-range fiber networks.

Action: The Outlook has been updated to better highlight the mentioned opportunities.

13. The authors should clarify whether the diarylketone sublimes under vacuum at room temperature (since the sample is under vacuum at the start of the cool down).

Response: In our cryo-confocal setup, we have not observed any evidence of noticable sublimation during operation at cryogenic temperatures and during the intial pumping phase at room temperature. Visual inspection of the bulk crystals before insertion and after removal from the sample stage shows no macroscopic changes that hint at lost material due to sublimation. We agree that the most critical phase for potential sublimation is the initial pump-down of the cryostat chamber at room temperature to approximately 10^{-3} mbar, which typically takes about 10 minutes before the active cooling process begins.

However, considering that the analytical purification of the diarylketone matrix via vacuum sublimation requires significantly harsher thermal conditions (sublimation 3×10^{-2} mbar at 195 °C typically carried out over 16 h), the vapor pressure at room temperature is assumed to be exceedingly low. Consequently, we do not expect any significant material loss or structural impact on our bulk crystal samples, which are approximately 1 mm^3 in size, during the brief room-temperature vacuum phase.

Having said that, while bulk crystals can be considered stable, we are currently developing methods to deposit thin films of these materials (< 100 nm) for future integrated device applications. In these regimes, where the surface-to-volume ratio is substantially higher, we actively ensure the long-term integrity of the materials under vacuum by employing encap-

sulation strategies, such as coating the samples with a thin layer of polyvinyl alcohol (PVA).

14. The authors show microwave transitions involving T0z. It would be helpful to clarify if they can observe transitions between T0x and T0y (e.g., under off-resonant excitation). This would be helpful in clarifying the selectivity between T0x and T0y from the ISC.

    Response/Action: We have performed ODMR measurements using off-resonant laser excitation and have swept the microwave transition across the resonance condition for the $T_{0\mathrm{x}} \leftrightarrow T_{0\mathrm{y}}$ transition. No change in fluorescence intensity was observed. Hence, we conclude that either too small population difference between these states was accomplished by ISC, or differences in population do not lead to detectable differences in fluorescence. For sake of completeness, pulsed ODMR on the XY transition is readily possible, as is now shown in the SI Sec. VII. M.

15. In the supporting information, the authors present a PLQY of 10%. They should clarify why this is relatively low and how it might be improved.

    Response: We thank the reviewer for highlighting the photoluminescence quantum yield (PLQY). This is an important metric, and we agree that it requires clearer contextualization.

    Both environmental rigidification by e.g. embedment in a crystalline matrix and cryogenic cooling are known to enhance photon emission by suppressing active phonon modes and modifying Franck-Condon overlaps (see Mishra, K. et al., *Chem. Sci.* **2024**, *16*, 90; and S. Reineke, M. A. Baldo, *Sci. Rep.* **2014**, *4*, 3797). Taking these effects into account, we reassessed the PLQY of our doped molecular crystals at 4.5 K to better reflect actual operating conditions. Utilizing excitation saturation measurements, we determined the quantum efficiency of the triplet-to-triplet transition to be approximately 10%. While establishing this molecular spin-photon interface represents a fundamental breakthrough, we agree that optimizing the absolute PLQY is a critical next step. We identify two tractable strategies for significantly enhancing the emission yield in future iterations:

    First, molecular design and isotope engineering has recently shown to overcome low emission yields for related systems. For instance, recent work has demonstrated remarkable PLQY improvements—up to near-unity—through the targeted chemical modification of related emitters (Ghosh, P. et al. *Nature* **2024**, *629*, 355; Arnold et al. *Adv. Opt. Mater.* **2024**, *12*, 2400697). Furthermore, exploiting kinetic isotope effects via perdeuteration strongly suppresses non-radiative internal conversion. As we demonstrated for 2,2'-dinaphthylcarbene (Roggors, S. et al. *J. Am. Chem. Soc.*, **2025**, *147*, 36383–36392) and was shown for Dibenzoterrylene (Mishra, K. et al. *Chem. Sci.* **2024**, 16, *90*), substituting hydrogen with heavier deuterium reduces Franck-Condon overlap factors, which routinely increases the PLQY by a factor of 1.5 to 2.

    Second, the intrinsic processability of our molecular thin crystals uniquely enables their direct integration with photonic integrated circuits without destructive ion etching (as demonstrated recently in Huang, T. et al. *Nat. Nanotechnol.* **2025** *20*, 1748–1756 and Lange, C. M. et al. *ACS Nano* **2026** *20*, 9867–9874). Coupling these systems to photonic microcavities

allows for significant Purcell enhancement, which can increase the effective PLQY by an order of magnitude while preserving the emitter's narrow linewidth.

Action: We have now added a mention of the PLQY to the main text. The Conclusion and Outlook section now includes a discussion of the PLQY, as well as a discussion of the chemical, as well as quantum optical improvements that might be achievable.

16. It would be valuable for the authors to present single-spin T2* ODMR data to complement the Hahn-echo/dynamical decoupling data.

    Action: We added a comparative measurement of single spin $T_2^* = 5.3(4)\,\mu$s to the figure.

17. There is a typo on line 81: 'qubit molecules fluoresce'

    Response: We thank the reviewer for their careful reading of the manuscript. However, we respectfully clarify that "fluoresce" is used here as a verb and hence opted to retain the original phrasing in the revised manuscript.

Reviewer: 3

The authors present a novel spin molecular system with a relatively good quantum efficiency of fluorescence, allowing for single molecule addressing due to spectral selectivity at low temperature. Moreover, their single molecule emission lines show excellent stability and linewidths that are near the lifetime limit. They've shown control over the spin state of the molecule, and demonstrate long-lived coherence under dynamical decoupling. With that, they found a unique system in molecular spectroscopy, one that could possibly compete with solid-state systems as the NV center and many other spin defects. In particular, the high level of control on doping and in general, the relative ease of integration of molecular systems into devices, could make the author's system an interesting candidate for novel experiments with spins. However, the authors fail in reaching a broad audience for two main reasons, the first one being a language which often becomes very technical, only for the experts in the field only and, most importantly, because there is no thorough presentation of the figures of merit characterizing spin-photon interfaces. This makes it complicated to compare with the state-of-the-art. Below we provide suggestions for major revisions that should be addressed for the manuscript to be suitable for publication in Nature.

Major comments: Better discuss their results, providing important figures of merit and compare with respect to the state of the art and in particular

1. The number of detected photons:

   - In the SI a quantum yield of about 0.09 is mentioned, but it is not discussed in the main text.

     Response: We thank the reviewer for highlighting the photoluminescence quantum yield (PLQY). As detailed in our response to similar comments from the other reviewers, the baseline PLQY of 9% was measured at 77 K in a frozen solution. At our single-molecule operating temperature of 4.5 K, thermally activated, radiationless relaxation pathways can be further suppressed (see Mishra, K. et al., *Chem. Sci.* **2024**, *16*, 90; and

S. Reineke, M. A. Baldo, *Sci. Rep.* **2014**, *4*, 3797). Taking these effects into account, we reassessed the PLQY of our doped molecular crystals at 4.5 K to better reflect actual operating conditions. Utilizing excitation saturation measurements, we measured a PLQY of around 10%, in good agreement with previously disclosed values (see also the answer to the next reviewer comment).

We agree that improving the absolute PLQY is a critical next step. We identify two tractable strategies for significantly enhancing the emission yield in future iterations:

Targeted molecular design and isotope engineering offer documented pathways to overcome low emission yields. For example, functionalizing related open-shell systems with specific electron-donating groups has been shown to dramatically increase the PLQY, even approaching near-unity efficiency (Ghosh, P. et al., *Nature* **2024**, *629*, 355; Arnold et al., *Adv. Opt. Mater.* **2024**, *12*, 2400697). Additionally, leveraging the kinetic isotope effect through perdeuteration strongly suppresses non-radiative internal conversion. As demonstrated in our work on 2,2'-dinaphthylcarbene (Roggors, S. et al., *J. Am. Chem. Soc.* **2025**, *147*, 36383–36392) and in studies on dibenzoterrylene (Mishra, K. et al., *Chem. Sci.* **2024**, *16*, 90), substituting hydrogen with heavier deuterium lowers Franck-Condon overlap factors, routinely boosting the PLQY by a factor of 1.5 to 2.

Second, the inherent processability of our molecular thin crystals allows for direct integration into photonic integrated circuits without the need for destructive ion etching. By coupling these systems to photonic microcavities, we can take advantage of significant Purcell enhancement. This can increase the effective PLQY by an order of magnitude while preserving the emitter's narrow linewidth.

Action: We agree that this context is important and have now added a discussion to the main text to explicitly address the quantum yield and the straightforward pathways for its improvement.

- Have the authors measured a saturation curve? (All figures are given in arbitrary units, therefore it is difficult to get an estimate of the typical photon emission rate).

Response: We thank the reviewer for this important point. In accordance to a similar point raised by reviewer 2, we altered the y-axis of figure 4 c and d, to better give an inside into the absolute fluorescence intensity. Together with the absolute fluorescence levels in figure 3 a and f, the main text figures should now present a comprehensive picture.

We have added a microwave-assisted single-molecule saturation measurement to the Supporting Information. Under the resonant detection conditions used for single-molecule spectroscopy, the maximum detected count rate is $I_{max} = 4.58$ kcounts/s at a laser power of $P_{max} = 22.79$ nW. Due to the more complex relationship between laser power and fluorescence count rate, we refrain from quoting saturation values, but the maximum amount of fluorescence and the respective laser power. These values refer

to the detected fluorescence count rate in the present confocal setup, using phonon-sideband detection after spectral filtering of the resonant excitation laser (total photon detection efficiency is estimated as 1.3%), and should therefore not be interpreted as the total photon emission rate of the molecule.

The saturation behavior of this system is less directly accessible than for a closed two-level emitter. Because single-molecule experiments are performed by resonantly addressing one spin-selective optical transition, optical excitation pumps the molecule into the $T_{0\mathrm{z}}$ and $T_{0\mathrm{x}}$ spin state, which is dark for the addressed transition. Continuous fluorescence therefore requires microwave repumping through the ZX and ZY transitions. Consequently, the observed steady-state fluorescence rate depends on the applied microwave Rabi frequencies and is limited by the combined optical excitation, intersystem-crossing and microwave-repopulation dynamics, rather than by saturation of the optical transition alone.

We also would like to clarify the distinction between this steady-state photon count rate and the pulsed readout traces in Fig. 4d. The latter show the transient fluorescence immediately after spin preparation; the signal decays as the molecule is optically initialized into $T_{0\mathrm{z}}$. Thus, Fig. 4d is the relevant quantity for the detected photons per spin-readout event, whereas the new SI saturation curve provides the steady-state single-molecule brightness under continuous resonant excitation and microwave repumping.

Action: We added a new section to the SI (Sec. VII. F.) including the saturation curves and fitted values $I_{max} = 4.58$ kcounts/s and $P_{max} = 22.79$ nW. Together with the new estimation of the experimental collection efficiency (SI, Sec. VII.A.), this allows for the deduction of the PLQY of a single molecule (as was mentioned in the answer to the previous question). We also changed the axes of Figs. 4c,d to show absolute detected fluorescence quantities and added a short clarification in the main text/Methods that the detected fluorescence rate is microwave-repump limited under resonant excitation.

- Could the authors comment on the strong bunching observed in the g(2) measurement? Do the authors have more data that can be used to extract an intersystem crossing/blinking rate?

  Response: For the single molecule experiments, we utilized spectrally selective excitation, to separate a single molecule from the spatial ensemble. Consequently, since the spectral separation of the spin-selective excitation lines is larger than their respective inhomogeneous broadening, only the $T_{0\mathrm{y}}$ to $T_{1\mathrm{y}'}$ transition is excited. Due to the non-zero ISC branching ratio for the $T_{1\mathrm{y}'}$ state, and subsequent decay predominantly to the $T_{0\mathrm{z}}$ state, continuous excitation leads to a rapid pumping to a dark state. This is the same effect that can be seen in the blue curve in Fig. 4d (in fact, ignoring some normalization, the experiment from Fig. 4d would show the same functional behavior, as the $g^{(2)}(\tau)$ function for $\tau \geq 0$). Thus, the decay time of the bunching is a convolution of both the optical excitation rate, as well as the ISC rate. In addition, as mentioned in the Methods section under "Cryogenic Microscopy and Spectroscopy Experiment",

we employ a MW drive between the T_{0z} and T_{0y} transition, in order to recover the molecule from its dark spin state. This drive defines the baseline of the $g^{(2)}(\tau)$ function, and consequently shortens the decay time of the observed bunching. While the ISC rate cannot be directly deduced from the measured decay of the bunching, in the SI Sec. VII. F. we fit a model of the electronic system of the molecule to the optical saturation measurements, allowing us to deduce an ISC rate.

Action: We added a discussion of the (anti-)bunching rate in the Methods section of the manuscript. We added a section to the SI showing saturation measurements, deducing an ISC rate (Sec. VII. F.).

- What is the impact of such low QY with respect e.g. to NVs in performing as spin-photon interface?

  Response: We thank the reviewer for raising this important point. The photoluminescence quantum yield (PLQY) is a critical metric for any spin-photon interface, and we agree that a discussion of its impact relative to established benchmarks like the NV center is warranted. For the presented work, as well as future experiments in similar experimental setups, the direct impact of a PLQY of 10% for resonant excitation, when compared to the highly efficient NV center, is a significantly lower steady-state photon count rate (currently on the order of a few kcounts/s). Especially when performing experiments that are dependent on a high photon flux (i.e. measurements where averaging over many repetitions is not feasible, for example single-shot spin readout), a low quantum yield directly influences the achievable SNR of the readout. However, for applications where spin-photon entanglement is leveraged, the Debye-Waller factor of the emitter plays an equal role in determining the ability to emit not only a photon, but a photon capable of carrying quantum information. In fact, the product of PLQY and Debye Waller factor, i.e. the overall probability to emit a photon without phononic influence, can be found in Atatüre, M.et al., *Nat Rev Mater* **2018** *3*, 38–51 for several spin-photon interfaces. The NV center lands at $0.8 \cdot 0.03 = 0.024$, and our presented molecule at $0.108 \cdot 0.1947 = 0.021$. The SiV defect in diamond, known for its good optical properties, outperforms the presented carbene molecule by a factor of 3.3. Given our discussion on the reviewer's previous question, we are confident that the PLQY can be further improved by chemical means, in addition to coupling emitters to cavities.

  Action: We added a comparison of different spin-photon interfaces, SI, VIII. We also mention the product of PLQY and the Debye-Waller factor in the "Conclusion and Outlook" section of the manuscript.

2. What is the typical contrast of the optical readout of the spin levels? How does this compare to existing platforms?

   Response: We thank the reviewer for asking us to clarify this important figure of merit. In general, one can distinguish two different mechanisms to achieve contrast in optical spin readout:
   i) Under off-resonant optical excitation an emitter is excited independent of its spin state and ISC leads to spin polarization in the ground state and to spin-dependent fluorescence levels for excited states. The contrast for optical readout is mainly limited by the ISC rates and can

be adjusted by excitation rate or temporal filtering of fluorescence response. The contrast ranges from minuscule values to several tens of percent.
ii) Under spin-selective, resonant optical excitation fluorescence will only be excited once the spin is in the matching state until any spin-non-conserving process (e.g. T_1, ISC, or "forbidden" optical transitions) switches the spin state. Spin state initialization is achieved via optical pumping or measurement. The contrast of optical readout is typically close to unity. There are several factors preventing unity contrast. For example inefficient optical pumping, imperfect spin-selectivity of the optical excitation or background fluorescence.

We now distinguish between three different quantities: the spectral spin selectivity of the optical transition, the spin initialization by optical pumping and the experimentally detected fluorescence contrast in a pulsed readout experiment.

The spectral spin selectivity is very high. The narrowest relevant separation between spin-selective optical transitions is the single-molecule XX'–YY' splitting of $\Delta f_{XY} \approx 1.56\,\mathrm{GHz}$, while the measured single-molecule optical linewidth is $38(2)\,\mathrm{MHz}$. Assuming a Lorentzian line shape, this corresponds to an off-resonant excitation probability of approximately $\epsilon_{\mathrm{off}} = 1/[1 + (2\Delta f/\Gamma)^2] \simeq 1.5 \times 10^{-4}$, or a spectral excitation selectivity of about 99.985%. The separation from the ZZ' transition is even larger: from the experimentally determined level structure we estimate $\Delta f_{ZY} = 14.58\,\mathrm{GHz}$ and $\Delta f_{XZ} = 16.12\,\mathrm{GHz}$, corresponding to off-resonant excitation probabilities below 2×10^{-6}. Thus, optical leakage caused by spectral overlap of spin-selective transitions is negligible under our resonant readout conditions.

Spin initialization by optical pumping in our case happens by excitation on the YY' optical transition, which eventually after a few cycles leads to ISC and transition into $T_{0\mathrm{z}}$. This process takes a few microseconds (see Figure 4d of the manuscript) and has to be compared to $T_1 = 21(2)$ ms in the ground state, the time scale on which population is equilibrated. As these time scales differ by about 4 orders of magnitude optically depopulating $T_{0\mathrm{y}}$ is highly efficient. The ground state ISC favors $T_{0\mathrm{z}}$ over $T_{0\mathrm{x}}$ and $T_{0\mathrm{y}}$ by a factor on the order of 1000 and therefore limits the degree of polarization due to optical pumping (see SI Sec IV. F. 8. and VII. M. 1.). In SI Sec VII. M. 8. it is also shown how this limit can be overcome by simultaneous optical pumping on transition YY' and microwave radiation on transition XY. Eventually, we deduce a degree of optical polarization of about $\sim 999 : 1$.

The experimentally detected fluorescence contrast is quantified separately from spectral selectivity and optical initialization. In the pulsed readout experiment of Fig. 4(d), resonant excitation of the YY' transition yields a bright transient when the molecule is prepared in T_{0y}, whereas the T_{0z} state remains dark apart from background counts. Using the same integration window as in Fig. 4(c), we obtain $N_{\mathrm{bright}} = 0.0103$ detected photons and $N_{\mathrm{dark}} = 0.0022$ detected photons per readout pulse, corresponding to a relative readout contrast $C_{\mathrm{rel}} = (N_{\mathrm{bright}} - N_{\mathrm{dark}})/N_{\mathrm{bright}} = 78.8\%$, or, equivalently, a Michelson contrast $C_{\mathrm{M}} = (N_{\mathrm{bright}} - N_{\mathrm{dark}})/(N_{\mathrm{bright}} + N_{\mathrm{dark}}) = 65\%$. The contrast can be improved to a relative contrast of 95% by choosing shorter readout windows (see SI Figure 53). Eventually, the contrast in optical spin readout is limited here by the count rate of a single emitter compared to the background count rate.

This contrast is high enough to enable the averaged single-spin Rabi, Hahn-echo, and dynamical-decoupling measurements shown in Fig. 4. However, the detected photon number per readout event is still well below one in the present confocal implementation, so the current experiment does not yet provide full single-shot spin readout. Compared with existing solid-state spin-photon platforms, the molecular carbene is highly favorable in terms of intrinsic spectral spin selectivity at zero applied magnetic and strain field, but the detected photon budget and hence the single-shot readout fidelity are not yet competitive with mature defect-center systems that benefit from optimized cycling transitions, photonic extraction, or cavity enhancement. We furthermore want to highlight, that repetitive readout distinguishing subspaces $T_{0\mathrm{x}}$ and $T_{0\mathrm{y}}/T_{0\mathrm{z}}$ is feasible, and almost reaches the SNR necessary for single-shot readout.

Action: We have revised the manuscript mentioning that the experimentally measured fluorescence readout contrast from Fig. 4(d) is limited by signal background and we have added SI Sec. VII. M. to give more details about the spin readout. We also clarify that single-shot readout will require increased photon collection, improved PLQY, and/or photonic enhancement. For the results on repetitive readout see also SI Sec. VII. M.

3. Which scheme could be employed to realize spin-photon entanglement and what are the estimated entanglement generation efficiency and fidelity based on the available experimental results?

   Response: We thank the reviewer for asking us to make this point more quantitative. A natural spin–photon-entanglement protocol for the present molecule is a time-bin scheme based on the spin-selective optical transitions. After optical initialization into T_{0z}, a microwave $\pi/2$ pulse prepares a superposition of T_{0z} and T_{0y}. A spectrally selective resonant optical pulse on the YY' transition then excites only the T_{0y} component. Radiative decay back to T_{0y} emits an "early" photon. A subsequent microwave π pulse swaps the spin populations, followed by a second identical optical pulse, which emits a "late" photon from the other spin component. Conditioned on detecting exactly one ZPL photon, this generates a spin–time-bin entangled state of the form $|\Psi\rangle = 2^{-1/2}(|T_{0y}\rangle|E\rangle + e^{i\phi}|T_{0z}\rangle|L\rangle)$, up to known microwave and optical phases. This is closely analogous to time-bin spin–photon-entanglement protocols used in solid-state spin defects and can, in principle, be converted into polarization encoding using an unbalanced interferometer.

   Based on the currently measured properties, the intrinsic spin selectivity of such a protocol is very favorable. The smallest measured separation between spin-selective optical transitions is the XX'–YY' splitting of $1.555(1)$ GHz, while the measured single-molecule linewidth is $38(2)$ MHz. Assuming a Lorentzian line shape, this gives an off-resonant excitation probability of approximately $\epsilon_{\mathrm{off}} = 1/[1 + (2\Delta/\Gamma)^2] \simeq 1.5 \times 10^{-4}$.

   The calculated spin-state overlap between ground- and excited-state triplet sublevels is > 0.998, implying that spin-nonconserving optical excitation contributes at most a few 10^{-3} to the intrinsic error budget. Thus, from spectral selectivity and spin conservation alone, the intrinsic state-selectivity-limited fidelity is expected to be $F_{\mathrm{selectivity}} \gtrsim 0.997$.

The overall entanglement fidelity, however, also depends on quantities that are not yet fully optimized or directly measured in the present work: spin-initialization fidelity, microwave π-pulse fidelity, residual background photons, pulsed multi-photon probability, and the optical coherence of the emitted photons. The measured $g^{(2)}(0) = 0.142$ confirms single-emitter operation, but a dedicated pulsed $g^{(2)}$ measurement under the proposed entanglement sequence would be needed to assign a full multi-photon-error contribution. In addition, the measured linewidth of $38(2)\,\mathrm{MHz}$ is broader than the lifetime limit of $6.6(5)\,\mathrm{MHz}$, so photon indistinguishability and remote two-photon-interference protocols will benefit from further line narrowing, lower-temperature operation, or Purcell enhancement. We therefore now report a selectivity-limited fidelity estimate rather than claiming a measured end-to-end spin–photon-entanglement fidelity.

The generation efficiency can be estimated from the measured photon budget. For a successful spin-selective excitation, the internal probability to emit into the ZPL is approximately $p_{\mathrm{ZPL,int}} \simeq \Phi_{\mathrm{PL}}\beta_{\mathrm{ZPL}}$. Using the experimentally measured PLQY of 10.8% and the Debye–Waller factor of 19.47%, we obtain $p_{\mathrm{ZPL,int}} \approx 2.1\%$ before collection and optical losses. The detected success probability is therefore $p_{\mathrm{det}} \simeq 0.021\eta_{\mathrm{sys}}$, where η_{sys} denotes the total collection, routing, and detector efficiency for ZPL photons. This value should be regarded as a conservative estimate based on the currently measured molecule. Future molecular optimization of PLQY, improved photon extraction, and cavity-enhanced ZPL emission could increase this probability substantially.

Finally, we note that the pulse sequence should use spectrally selective nanosecond-scale or shaped resonant pulses, not picosecond pulses, because the optical bandwidth must remain well below the GHz-scale spin-selective transition separation. The present excited-state lifetime of $24(2)\,\mathrm{ns}$ is compatible with high repetition rates in principle, but in the current experiment the practical rate is limited by spin shelving, microwave repumping, and photon collection rather than by the excited-state lifetime alone.

Action: We added references to future applications for spin-photon entanglement in the Outlook, see also the response to the reviewer's remark below. While above answer shows the foundation for future experiments, we refrain from putting this level of detail in the manuscript.

4. Have the authors measured behaviour with respect to a magnetic field? Is there prospect for magnetometry?

   Response: We have not performed a dedicated single-molecule magnetic-field-dependence study in this work. The zero-field splitting is large enough that small magnetic fields perturb the m_S-mixed eigenstates rather than defining a simple Zeeman basis. A quantitative magnetometry analysis therefore requires a separate orientation-dependent study, and might not be as straightforward as e.g. for NV centers in diamond.

Based on the answers to the previous questions the authors could propose some outlook of what the system can be used for:

- For instance what kind of protocol you could envisage with this new system and which most sound applications for spin-photon interfaces?

  Response: We thank the reviewer for giving us the opportunity to clarify the broader outlook of this platform. The most sound applications for this molecular spin-photon interface are as quantum nodes for distributed fault-tolerant quantum computing (FTQC) (Nickerson, N. H. et al., *Phys. Rev. X* **2014** *4*, 041041 and Nemoto, K. et al. *Phys. Rev. X* **2014** *4*, 031022) and as quantum repeater links for long-distance quantum communication networks (Wehner, S. et al. *Science* **2018** *362*, eaam9288 and Bhaskar, M. K. et al. *Nature* **2020** *580*, 60–64).

  The high-fidelity spin-photon entanglement detailed in the question above can be utilized to entangle distant carbene molecular nodes for these applications. This remote entanglement can be heralded either by a single-photon detection scheme or by a highly robust two-photon interference measurement, such as the Barrett-Kok protocol (Barrett, S. D. & Kok, P. *Phys. Rev. A* **2005** *71*). In these protocols, the highly spin-selective optical transitions of the carbene molecule would be utilized to entangle its ground-state electron spin with the emission of a photon. Utilizing this remote entanglement, high rate quantum repeaters or distributed quantum computing can be potentially made feasible.

  A key challenge in the scalability of such networked systems, is the integration with photonic integrated circuits (PICs). The intrinsic processability of molecular systems facilitates their direct integration with PIC processing technologies, such as silicon nitride or lithium niobate. This capability builds on recent advancements in integrating organic molecules for photonic quantum technologies (Toninelli, C. et al. *Nat. Mater.* **2021** *20*, 1615–1628.), with recent results demonstrating high indistinguishability from different molecular emitters on chip (Huang, T. et al. *Nat. Nanotechnol.* **2025** *20*, 1748–1756). While significant work is required to demonstrate the integration of carbene molecules with PICs, the ability to grow very thin plates of the undoped crystals presents a promising direction for future development of microtransfer printing of carbene-doped crystals onto PICs.

  Action: We added the above citations to the Outlook section of the manuscript.

- depending on the envisaged experiment, could the authors comment on the actual advantage of addressing a single molecule?

  Response: We thank the reviewer for asking us to clarify this point. The advantage of addressing a single molecule depends on the target experiment, and we agree that ensembles remain highly valuable for other tasks, such as absorption-based quantum memories or collectively enhanced light–matter interfaces. Our claim is not that single molecules are universally superior to ensembles. Rather, single-molecule addressability enables a complementary class of experiments in which the stationary qubit is an individually controlled spin with a one-to-one optical interface.

  For spin-photon-interface and quantum-network experiments, the main advantage is that the emitted photon is correlated with a single, well-defined spin state rather than with an inhomogeneous ensemble. In our system this distinction is especially important because

the ensemble optical excitation line is broadened to approximately 3 GHz, whereas an individual molecule exhibits a linewidth of 38(2) MHz and hour-scale spectral diffusion of only 2.4–2.6 MHz. Thus, single-molecule isolation removes the dominant ensemble inhomogeneity and allows one to select and stabilize a specific optical transition. This is a prerequisite for generating photons suitable for high-visibility two-photon interference between remote nodes, although additional spectral tuning and photonic mode matching will still be required.

A second advantage is that the quantum information is stored in a single, localized Hilbert space. In the present work, resonant excitation of one spin-selective optical transition gives fluorescence only when the molecule occupies the addressed spin state, while microwave pulses coherently manipulate that same individual ground-state triplet spin. This direct mapping between one spin and one optical transition is the relevant resource for protocols based on spin-photon entanglement, heralded remote entanglement, local quantum logic with nuclear registers, and repeated single-qubit readout. Ensemble protocols can generate collective spin-wave excitations, but those are not equivalent to deterministic control of an individually addressable stationary qubit.

Single-molecule addressability is also important for photonic integration. Coupling a single emitter to a cavity or waveguide avoids ambiguity in emitter number, multi-emitter spectral congestion and collective averaging over different local environments. This is particularly relevant for our molecular platform because the emitter density can be adjusted by a combination of precursor doping and local photoactivation, while the molecular crystal can in principle be processed as a thin film on pre-fabricated photonic structures.

Finally, for quantum-sensing and molecular-spin-interaction experiments, a single molecule provides spatially local information and avoids ensemble averaging over orientations, strain fields and microscopic nuclear-spin environments. Conversely, deliberately increasing the activated molecule density would allow future studies of controlled dipolar spin networks. We have therefore revised the Outlook to state more explicitly which applications benefit from single-molecule addressability and where ensemble operation remains complementary.

Action: We added a short paragraph at the beginning of to the Outlook clarifying that single-molecule addressing is advantageous specifically for one-to-one spin-photon interfaces, while ensembles remain useful for collective-memory-type protocols.

Language: sometimes a bit technical for a broad audience, see for instance on line 46 ”it diminishes wavefunction overlap of the qubit with the surrounding matrix via a dominant edge-to-face packing motif”.

Action: We agree with the reviewer that the language here is dense and revised this paragraph while avoiding overly technical jargon

- The authors claim that the molecules were engineered to possess the found properties, such as the difference in the ZFS of ground and excited states that facilitates the spin selective optical transitions, but it seems hard to believe that it was actually so. In that regard, the authors could substantiate their claim by commenting a bit on the outlook of chemical tunability. What properties would you change or improve and what would be possible pathways

to achieve it?

Response: We apologize for conveying the impression that the specific ZFS properties and intersystem crossing ISC dynamics were *a priori* engineered by rational design. We agree that the substantial difference in the ZFS between the ground and excited states which facilitates our spin-selective optical transitions is a beneficial intrinsic byproduct of diaryl-carbenes, rather than a deliberately engineered feature.

Predicting excited-state ISC rates *ab initio* using e.g. multiconfigurational methods such as CASSCF, remains a delicate endeavor. As discussed in the SI (Sec. IV. F. 8), the ISC rate is likely dominated by a second-order spin-vibronic mechanism. In this framework, the $T_1 \sim S_2$ transition borrows intensity via non-adiabatic vibrational mixing between the closely spaced T_1 and T_2 states. Currently, further theoretical studies are needed to establish a reliable predictive framework for these specific line-borrowing mechanisms in carbenes. Having said that, we would like to highlight several principles of chemical tunability offered by molecular design building upon recent theoretical studies by Y. R. Poh, et al. (*J. Am. Chem. Soc.* **2024**, *146*, 15549; *J. Am. Chem. Soc.* **2025**, *147*, 22529.).

The sign change of the axial (*D*) and rhombic (*E*) ZFS parameters when transitioning from the ground state to the excited state is a finding that results from the formal redistribution of spin density. Upon electronic excitation, spin density shifts from the central p- and σ-orbitals to the attached phenyl rings. This spatial redistribution changes the shape of the ZFS tensor from oblate to prolate (Richert, S. et al. *J. Magn. Res.* **2017**, *280*, 103.). In that, we find that by using electron donating or accepting groups attached to the central carbene can modify *D* within a limited range of ca. 8 - 12 GHz. More drastic changes can be achieved by enforcing changes in the carbene angle e.g. via steric contraint or tethering into closed rings ca. 4 - 15 GHz (see for example the comprehensive review by Hirai, K., et al. (*Chem. Rev.* **2009**, *109*, 3275.). The rhombic ZFS parameter on the other hand is highly sensitive to the internal carbene angle. Specifically, *E* decreases and ultimately vanishes upon linearization of the carbene backbone (K. Yoshida et al., *BCSJ* **2004**, *77*, 1509; E. Iwamoto et al., *J. Am. Chem. Soc.* **2003**, *125*, 14664.). This offers a direct pathway to chemically tune the transverse ZFS by synthetically constraining the steric bulk around the carbene center. Accordingly, our current design strategy aims at more bent carbenes to increase *E* and, hence, the protection of our qubit from the nuclear spin bath by clock transitions.

Beyond that, utilizing a triplet carbene with a comparatively high singly occupied molecular orbital (SOMO) energy at the carbene carbon strongly favors the population of the carbene non-bonding orbital (Y. R. Poh, et al., *J. Am. Chem. Soc.* **2025**, *147*, 22529.). This electronic configuration facilitates ΔMS = 0 selective ISC in the ground state. This feature was found to be inherent to bent carbenes, providing a robust structural foundation that can be fine-tuned to maximize spin initialization fidelity.

Action: We have rephrased the respective section in the manuscript to correct this misconception. We furthermore added a discussion explicitly on the low PLQY, and how to improve it.

Correctness: Lack of evidence on specific cases

- The photoactivation method offers a powerful feature in that it enables the activation of carbenes with sub-micrometer precision; this needs to be shown and explained in more detail in the main text, as the authors present it in the introduction as an important property of their system. In the fluorescence images for instance, it is not clear that these emitters are photoactivated. A grid/drawing of emitters or a before+after image would be substantial proof. Would it be possible to deterministically activate them for instance in an array geometry?

  Response and Action: Indeed, the localized photoactivation of carbenes is a very useful feature and we agree that it is not quite clear for example from figure 2a that these fluorescent spots are artificially created. Compared to figure 2a the single emitters in figure 3a are randomly distributed because we scanned a mild activation laser across the whole field of view as mentioned in the text and methods. We like to emphasize that this photoactivation process is not a unique feature of di([1,1'-biphenyl]-4-yl)carbene. In (Roggors, S. et al. *J. Am. Chem. Soc.*, **2025**, *147*, 36383–36392) we have demonstrated the activation of DNC ensembles in a regular pattern. We have now added a new section to the SI (Sec.VII. B.) where we provide details about the photoactivation method and creation yield. Along with that we have also created a regular patterns of di([1,1'-biphenyl]-4-yl)carbene the confocal image of which is shown in SI Figure 37.

Minor comments:

- What is the stability of the molecules at room temperature?

  Response: The activated carbenes are highly stable at cryogenic conditions and maintain thermal stability up to at least 250 K, showing no signal loss in EPR measurements within measurement error over several hours. At around room temperature (approximately 300 K), the carbenes become unstable, exhibiting a slow signal decrease over the course of several hours. This is mentioned in the SI Sec. VI. C.

- There is a factor 6 difference of the found wavelength with respect to the Fourier limit. Is this correct? What does this imply?

  Response: This is correct, the measured linewidth of the optical excitation is broadened to 38 MHz, compared to the Fourier limited linewidth of potentially 6.6 MHz. In order to elaborate on the additional optical decoherence, we have further investigated the temperature dependence of optical resonances in excitation spectroscopy. When raising the temperature we see an increase in linewidth and transition frequency that obeys an Arrhenius law, a very similar dependence as for Terrylene in p-terphenyl Kummer, S., et al. *J. Chem. Phys.* **1997**, *107*, 7673–7684). The results suggest that with increasing temperature phonons are more likely excited that finally lead to increased decoherence of optical transitions, and further cooling our molecules might further reduce the linewidth.

  Action: We added Sec. VII. J. to the SI, showing measurements regarding the temperature dependence of the transition frequency and linewidth.

- Why is there a change in fluorescence intensity over time in Figure 3f?

  Response: At the beginning of the measurement, the microscope position was fixed to the molecule at the relative laser frequency of -3.4 GHz. During the measurement, the microscope focus slightly drifted relative to the molecule at -3.4 GHz, reducing the obtained fluorescence rate. At the same time, the molecule at -3.9 GHz moved into the focus, increasing its fluorescence rate over time.

  Action: We now mention this effect in the text.

- Displaying double bonds in Figure 1a shows directly that there are two bonds missing in the middle of the carbene. Maybe this could be highlighted.

  Response: We thank the reviewer for the suggestion regarding the structural representation of the carbene center. While drawing localized double bonds would visually emphasize the divalent nature of the central carbon, we decided to employ the delocalized aromatic ring notation for the biphenyl substituents to avoid giving the misleading impression that the system contains localized double bonds.

  Action: We have updated the sketch of the molecule Figure 1a,d in the revised manuscript.

- Figure 4d could use some clarification: is this a measurement of how fast the spin is reset optically?

  Response: In addition to showing the fluorescence contrast of the optical spin readout, figure 4d also shows the spin polarization effect to the T_{0z} state due to optical excitation.

  Action: We added a small sentence to the caption, and a reference to the figure at the respective position in the main text.

- Add a drawing of the optical setup

  Action: We added a sketch of the optical setup to the SI Sec. VII. A. along with an estimate of the photon collection and detection efficiencies.

References: In the introduction the authors should cite J. Wrachtrup et al., Optical detection of Magnetic resonance in a single molecule, Nature 1993. This would also allow commenting on the difference of addressing ground state vs excited state spin levels.

Action: We acknowledge the oversight, and added a part in the Introduction. It now leads from excited state triplet systems (Wrachtrup et al.) towards the several implementations of molecular ground state electron spins.

A Single-Molecule Spin-Photon Interface

The authors present a novel spin molecular system with a relatively good quantum efficiency of fluorescence, allowing for single molecule addressing due to spectral selectivity at low temperature. Moreover, their single molecule emission lines show excellent stability and linewidths that are near the lifetime limit. They've shown control over the spin state of the molecule, and demonstrate long lived coherence under dynamical decoupling. With that, they found a unique system in molecular spectroscopy, one that could possibly compete with solid-state systems as the NV center and many other spin defects. In particular, the high level of control on doping and in general, the relative ease of integration of molecular systems into devices, could make the author's system an interesting candidate for novel experiments with spins. However, the authors fail in reaching a broad audience for two main reasons, the first one being a language which often becomes very technical, only for the experts in the field only and, most importantly, because there is no thorough presentation of the figures of merit characterizing spin-photon interfaces. This makes it complicated to compare with the state-of-the-art .
Below we provide suggestions for major revisions that should be addressed for the manuscript to be suitable for publication in Nature.

Major comments:

Better discuss their results, providing important figures of merit and compare with respect to the state of the art and in particular

1. The number of detected photons:
- In the SI a quantum yield of about 0.09 is mentioned, but it is not discussed in the main text.
- Have the authors measured a saturation curve? (All figures are given in arbitrary units, therefore it is difficult to get an estimate of the typical photon emission rate).
- Could the authors comment on the strong bunching observed in the $g^{(2)}$ measurement? Do the authors have more data that can be used to extract an intersystem crossing/blinking rate?
- What is the impact of such low QY with respect e.g. to NVs in performing as spin-photon interface?

2. What is the typical contrast of the optical readout of the spin levels? How does this compare to exsisting platforms?

3. Which scheme could be employed to realize spin-photon entanglement and what are the estimated entanglement generation efficiency and fidelity based on the available experimental results?

3. Have the authors measured behaviour with respect to a magnetic field? Is there prospect for magnetometry?

based on the answers to the previous questions the authors could propose some outlook of what the system can be used for:
- For instance what kind of protocol you could envisage with this new system and which most sound applications for spin-photon interfaces?
- depending on the envisaged experiment, could the authors comment on the actual advantage of addressing a single molecule?

Language: sometimes a bit technical for a broad audience, see for instance on line 46 "it diminishes wavefunction overlap of the qubit with the surrounding matrix via a dominant edge-to-face packing motif".

- The authors claim that the molecules were engineered to possess the found properties, such as the difference in the ZFS of ground and excited states that facilitates the spin selective optical transitions, but it seems hard to believe that it was actually so. In that regard, the authors could substantiate their claim by commenting a bit on the outlook of chemical tunability. What properties would you change or improve and what would be possible pathways to achieve it?

Correctness: Lack of evidence on specific cases

- The photoactivation method offers a powerful feature in that it enables the activation of carbenes with sub-micrometer precision; this needs to be shown and explained in more detail in the main text, as the authors present it in the introduction as an important property of their system. In the fluorescence images for instance, it is not clear that these emitters are photoactivated. A grid/drawing of emitters or a before+after image would be substantial proof.

Would it be possible to deterministically activate them for instance in an array geometry?

Minor comments:
- What is the stability of the molecules at room temperature?
- There is a factor 6 difference of the found wavelength with respect to the Fourier limit. Is this correct? What does this imply?
- Why is there a change in fluorescence intensity over time in Figure 3f?
- Displaying double bonds in Figure 1a shows directly that there are two bonds missing in the middle of the carbene. Maybe this could be highlighted.
- Figure 4d could use some clarification: is this a measurement of how fast the spin is reset optically?
- Add a drawing of the optical setup

References:
In the introduction the authors should cite J. Wrachtrup et al., Optical detection of Magnetic resonance in a single molecule, Nature 1993. This would also allow commenting on the difference of addressing ground state vs excited state spin levels.
